# Identification of 2R-ohnologue gene families displaying the same mutation-load skew in multiple cancers

**DOI:** 10.1098/rsob.140029

**Published:** 2014-05-07

**Authors:** Michele Tinti, Kumara Dissanayake, Silvia Synowsky, Luca Albergante, Carol MacKintosh

**Affiliations:** 1 Division of Cell and Developmental Biology, College of Life Sciences, University of Dundee, Dundee DD1 5EH, UK; 2 MRC Protein Phosphorylation and Ubiquitylation Unit, University of Dundee, Dundee DD1 5EH, UK; 3 Division of Computational Biology, College of Life Sciences, University of Dundee, Dundee DD1 5EH, UK

**Keywords:** cancer, mutations, 2R-ohnologue families, signal multiplexing, vertebrates

## Abstract

The complexity of signalling pathways was boosted at the origin of the vertebrates, when two rounds of whole genome duplication (2R-WGD) occurred. Those genes and proteins that have survived from the 2R-WGD—termed 2R-ohnologues—belong to families of two to four members, and are enriched in signalling components relevant to cancer. Here, we find that while only approximately 30% of human transcript-coding genes are 2R-ohnologues, they carry 42–60% of the gene mutations in 30 different cancer types. Across a subset of cancer datasets, including melanoma, breast, lung adenocarcinoma, liver and medulloblastoma, we identified 673 2R-ohnologue families in which one gene carries mutations at multiple positions, while sister genes in the same family are relatively mutation free. Strikingly, in 315 of the 322 2R-ohnologue families displaying such a skew in multiple cancers, the same gene carries the heaviest mutation load in each cancer, and usually the second-ranked gene is also the same in each cancer. Our findings inspire the hypothesis that in certain cancers, heterogeneous combinations of genetic changes impair parts of the 2R-WGD signalling networks and force information flow through a limited set of oncogenic pathways in which specific non-mutated 2R-ohnologues serve as effectors. The non-mutated 2R-ohnologues are therefore potential therapeutic targets. These include proteins linked to growth factor signalling, neurotransmission and ion channels.

## 2. Introduction

Over 500 million years ago, the vertebrates emerged from their invertebrate ancestor via an evolutionary leap involving two rounds of whole genome duplication (2R-WGD) [1–5]. Most of the resulting quadruplicated genes and proteins, termed 2R-ohnologues, were lost. However, the few thousand families of two to four 2R-ohnologues that still survive in modern humans are remarkably enriched in signalling molecules. These include families of growth factors, receptors, protein kinases, GTPases and their regulators, ion channels, transcription factors, developmental regulators and proteins that interact transiently with multi-protein complexes, and many of these are also 14-3-3-binding phosphoproteins [6,7]. By contrast, core components of stable multi-protein complexes such as RNA polymerases and the proteasome, and genes that were duplicated by small-scale genomic events after the 2R-WGD, tend not to be 2R-ohnologues [8,9]. These findings indicate that the 2R-WGD provided a selective boost to regulatory systems, which enabled vertebrate life to evolve. However, there appears to be a downside to the vertebrate style of signalling complexity: several studies have highlighted that 2R-ohnologue dysregulations are highly linked to neurodevelopmental and metabolic disorders, and to cancers [7,10,11].

Most cancers rely on a restricted number of ‘driver’ mutations, which confer cells with selective advantages that promote cancer initiation, progression and metastasis. Some driver mutations inactivate tumour suppressors such as p53, which normally induces growth arrest or apoptosis in stressed cells. Other drivers involve specific gene fusions, or point mutations such as B-Raf^V600E^, K-Ras^G12D^ and N-Ras^Q61R^, which activate oncogenic signalling pathways [12–14]. At the biochemical level, oncogenic signalling stimulates multiple intracellular changes that together promote the cancer phenotype. Interestingly, many tumour suppressors and oncogenic drivers, including p53, B-Raf and Ras proteins, belong to 2R-ohnologue families [11].

In addition to drivers, cancers can accumulate heavy loads of somatic mutations [15]. Most are considered to be ‘passengers’ that do not contribute to cancer progression [16]. However, we reasoned that there must be constraints on which mutations are compatible with any given oncogenic driver. If too many of the proteins that enact the downstream functions of an oncogenic driver were to suffer deleterious passenger mutations, the driver would lose its efficacy. Then, the cancer cell lineage would die out or become quiescent unless it acquired a different driver mutation that operates via a different mechanism, which might help explain cancer heterogeneity [17–19].

This study investigates how somatic mutations in cancers are distributed within families of 2R-ohnologue signalling genes. A general characteristic of 2R-ohnologues is that the domain architectures of the encoded proteins are conserved across each family. Therefore, the broad expectation is for members of a given family to share a high degree of overlap in their core functions. On the other hand, owing to localized sequence changes, family members may differ in their expression patterns, post-translational modifications, regulation by protein kinases, kinetic rates, binding affinities and substrate selectivities [8,20–23]. We therefore conceptualize each 2R-ohnologue protein family as a multiple-input multiple-output (MIMO) system that integrates different input signals to produce a combined functional output [7,24]. The phenotype of a cell will depend on which combinations of 2R-ohnologues from each family are expressed and engaged by the signalling pathways that operate in any physiological context [7,24].

Stemming from the MIMO signal-multiplexing concept, we hypothesized that 2R-ohnologues that are positive effectors of an active oncogenic driver should be kept free of loss-of-function mutations in a cancer. By contrast, mutations may accumulate in sister 2R-ohnologues that are components of signalling networks that are irrelevant or inhibitory to the cancer. Finding the putative non-mutated 2R-ohnologues would need cumulative data from multiple samples, because each cancer cell lineage might have only a few of the allowed mutations spread among many 2R-ohnologue families. The recent availability of large datasets of mutations in a variety of cancer types [15,25] therefore provided the first opportunity to explore this hypothesis. We reveal striking hierarchical patterns in the distributions of mutations within 2R-ohnologue families in cancers. Our findings support the concept of selected 2R-ohnologues being maintained free of mutations as ‘effectors’ in certain cancers.

## 3. Results

### 3.1. 2R-ohnologues carry a higher relative mutation load than non-ohnologues in every cancer type examined

**Figure 1.**
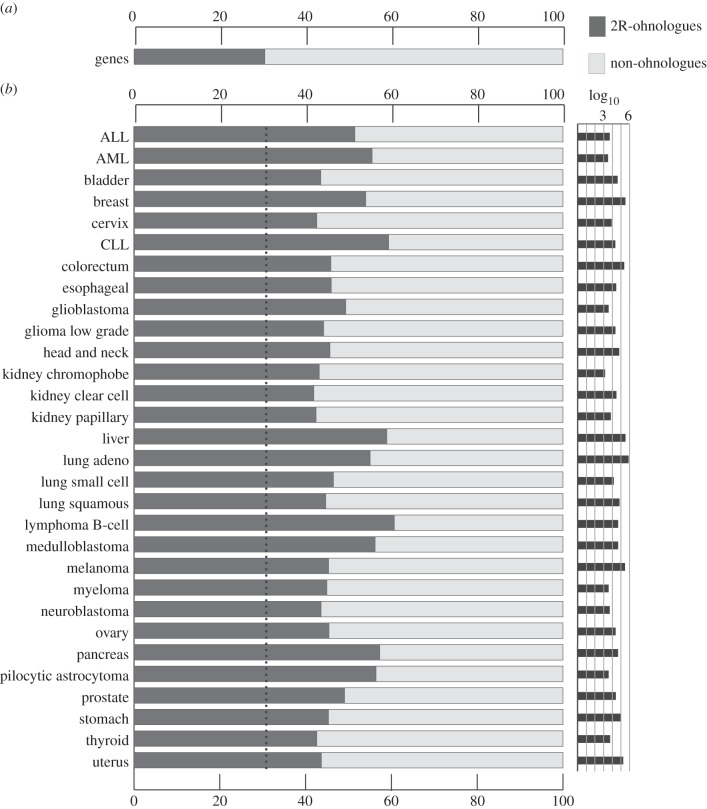
Distributions of cancer mutations between 2R-ohnologue and non-ohnologue transcript-coding genes in 30 different cancers. (*a*) The bar shows the percentage of 2R-ohnologue and non-ohnologue transcript-coding genes in the Ensembl 72 dataset, based on the provisional 2R-ohnologue list compiled by Makino & McLysaght [10]. Note that assignment of which human genes are 2R-ohnologues is still undergoing revision. (*b*) For each cancer type analysed [15], the graph on the left side reports the percentage of mutations that map on 2R-ohnologue and non-ohnologue genes. The right side reports the log_10_ value of the total number of mutations identified in each cancer type in this dataset.

Alexandrov *et al*. [15] validated almost 5 million somatic mutations identified in the genome sequences of 7042 samples from 30 different cancer types, but not in matched DNA from normal cells. We used the Alexandrov data to examine the distribution of mutations in 2R-ohnologue versus non-ohnologue genes (electronic supplementary material, table S1). A striking imbalance was revealed (figure 1). Although 2R-ohnologues comprise only approximately 30% of protein-coding genes in the human genome (figure 1*a*), they carry a higher proportion of the somatic mutations in transcript-coding genes in every cancer examined (figure 1*b*; statistics in the electronic supplementary material, figure S1). The proportions range from 42% of gene mutations located in 2R-ohnologues in kidney clear cell carcinoma to 60% in liver cancer and in B-cell lymphoma. The greater prevalence of mutations among 2R-ohnologues is not due to differences in gene sizes: 2R-ohnologue genes average 2400 nucleotides in length and non-ohnologues 2200 nucleotides (electronic supplementary material, figure S2).

### 3.2. Identification of 2R-ohnologue families in which one gene carries most of the mutations

We next investigated how the somatic mutations are distributed among members of each 2R-ohnologue family, analysing each cancer type separately. To this aim, each 2R-ohnologue gene was given a mutation load (ML) score (electronic supplementary material, table S2). A gene that accumulates most of the mutations for its 2R-ohnologue family scores close to 1, while genes that are clear of mutations relative to their sister genes score close to 0. Repeats of mutations at the same position were scored as a single mutation because we did not wish to rediscover, nor have the analysis dominated by, recurrent driver mutations. Also, we included only families for which at least one gene carried 10 or more different mutations in the cancer type. This method therefore captured genes with broad patterns of mutations at multiple positions.

**Figure 2.**
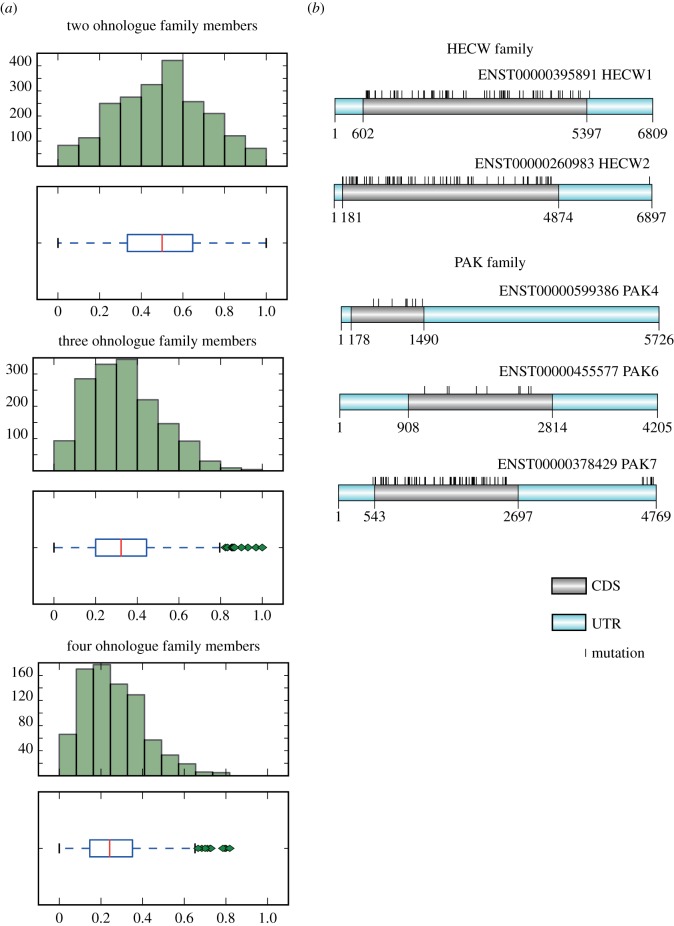
MLs of 2R-ohnologue genes in melanoma. (*a*) For the melanoma dataset [15], the figure plots the ML distribution for 2R-ohnologues within families of 2, 3 and 4 members. Only families in which at least one gene carries carried 10 or more different mutations are included. The ML is computed by summing the total number of mutations identified for a gene divided by the total number of mutations in all members of the same 2R-ohnologue family. The *y*-axes give the number of genes, with the ML scores indicated on the *x*-axes. Each histogram set indicates the medians (red lines), interquartile ranges (rectangular boxes) and outliers (green diamonds) for the ML distributions. Note that for families of 2 members, the median will always be 0.5 by construction, regardless of the ML distribution profile. Data file S1 in the electronic supplementary material presents the corresponding histograms for 30 cancer types, and its legend contains a discussion note about statistics. (*b*) The distribution of mutations in melanoma is given for the HECW E3 ubiquitin ligase family (as an example of even ML distribution) and the type II PAK family (an example with a skewed ML where the PAK7 gene accumulates most of the family mutations). Illustrations were created with Domain Graph, v. 1.0.5 [26]. CDS is coding sequence, UTR refers to 3′ and 5′ UTRs, and mutations are indicated by vertical black lines. Data file S2 in the electronic supplementary material gives similar diagrams for the distributions of mutations in other 2R-ohnologue families in melanoma.

The ML distributions for the melanoma dataset are graphed in figure 2*a* and for other cancers in the electronic supplementary material, data file S1 (with statistical note in legend). In melanoma and other tumour types, there were 2R-ohnologue families in which the MLs were evenly distributed among family members, such as the HECW family of two E3 ubiquitin ligases (figure 2*b*). However, in most cancers, including melanoma, there were also 2R-ohnologue families with a ‘skewed ML’, meaning that one gene carried most of the ML. For example, in the melanoma dataset PAK7 carries 95 of the 114 mutated sites in the family of three type II PAK protein kinases (figure 2*b*).

By contrast, there were no 2R-ohnologue families with skewed MLs in cervical, thyroid, myeloma and kidney papillary cancers (electronic supplementary material, tables S1 and S2). This was likely to be due to insufficient data for these cancer types; there were 10 or more mutations in only 23 2R-ohnologue genes in the cervical cancer samples, seven in thyroid, one in myeloma and nine in kidney papillary cancer samples. This may also be indicative of particular tumourigenesis mechanisms acting in these cancers.

### 3.3. In 315 2R-ohnologue families, the same gene carries the highest mutation load in multiple cancers

**Figure 3.**
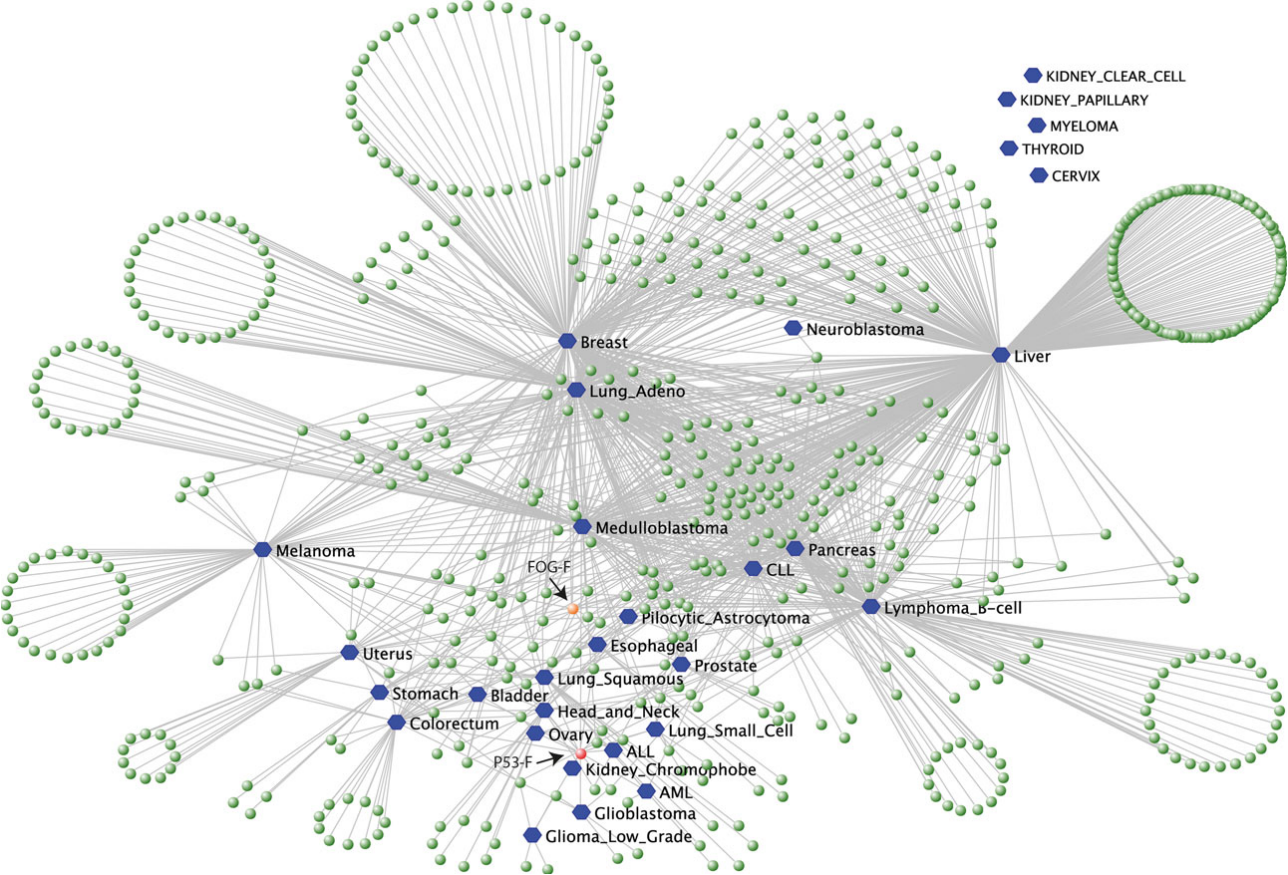
VisANT map of 2R-ohnologue families that display skewed MLs in different cancer types; graph created using VisANT (visant.bu.edu [27]) from the data in the electronic supplementary material, table S3. Each cancer was assigned a node in blue and lines connect these cancers to the 2R-ohnologue families (in green, red or orange) that display a skewed ML in that cancer. For a line to join a cancer and a 2R-ohnologue family, at least one gene in the family had to carry at least 10 different mutations in that cancer. Also, we plotted only those families with ML skew above the thresholds of at least 0.9 for families containing two genes (that is one gene carried more than or equal to 90% of the mutated positions for its family), at least 0.8 for families with three genes and at least 0.7 for families of four genes. The red node labelled ‘P53-F’ is the p53/p63/p73 family, and the orange node marked ‘FOG-F’ represents FOG1/FOG2.

A total of 673 2R-ohnologue families displayed highly skewed MLs in one or more cancers (electronic supplementary material, table S3). This dataset was visualized in a VisANT network graph in which each cancer type is assigned a hexagonal blue node, connected to circular nodes (green, red or orange) that each represent a 2R-ohnologue family with a skewed ML in that cancer (figure 3). The ‘elegant relaxing’ VisANT rule was applied [27], which means that 2R-ohnologue families with a skewed ML in only one cancer fan to the outside of the graph, while those with a skewed ML in multiple cancers are pulled towards the centre (figure 3). The layout of the graph therefore reflects patterns in the data.

Cancers around the top and sides of the graph, namely melanoma, breast, lung adenocarcinoma, liver, pancreatic, B-cell lymphoma and chronic lymphocytic leukaemia (CLL), are characterized by relatively high numbers of ML-skewed families (figure 3). Each of these cancers has 2R-ohnologue families for which ML skews were identified only in the individual cancer, which may be ‘signatures’ reflecting the tissues of origin of these cancers. However, these cancers are also highly interlinked by the many other ML-skewed 2R-ohnologue families that they have in common. Paediatric medulloblastoma is also highly interlinked, being central on the graph because it shares 120 ML-skewed families with other cancers, while only 20 ML-skewed families are unique to medulloblastoma (figure 3; electronic supplementary material, table S3).

Overall, 322 2R-ohnologue families displayed a ML skew in two or more cancers (electronic supplementary material, table S3). Remarkably, in 315 of these 322 families, the same gene carried the highest ML in each of the linked cancer types (figure 3; electronic supplementary material, table S3 with statistical note in legend, and data file S2). Furthermore, in most of these 322 families, the same gene carried the second highest ML for each family in each of the linked cancer types (table 1; electronic supplementary material, table S3, and statistical note in legend). For example, in the cumulative data for nine cancer types, the protein receptor tyrosine phosphatase PTPRD has many more mutated sites than PTPRS, while PTPRF is rarely mutated; in seven cancers, the Discs large homologue DLG2 is by far the most often mutated, DLG1 second, and DLG3 and DLG4 rarely mutated; the EGF receptor family member ERBB4 carries far more mutations than EGFR, while ERBB2 (HER2) and ERBB3 have relatively few nucleotides mutated in seven cancers (table 1; electronic supplementary material, table S3). Thus, the relative MLs in such 2R-ohnologue families are under selection pressures that act in a quantitative manner.

**Table 1.** Rank orders of ML scores within ML-skewed families. Electronic supplementary material, table S3, and figure 3 contain data for all the 2R-ohnologue families that have extreme ML skews. This table shows only those families of three and four members with high ML skews in at least four cancers. The table shows which 2R-ohnologues carry the highest number and second highest number of different mutations for its family. The last column indicates how many cancers have that same rank order of MLs for each family. The ML scores for the 2R-ohnologue families of two members can be viewed in the electronic supplementary material, table S3. The family identifier is an arbitrary number assigned to each 2R-ohnologue family in [7].

2R-ohnologue family identifier number and family description		most mutated member of the 2R-ohnologue family	second most mutated family member	least mutated family member(s)	no. cancers where the proteins indicated to the left are ranked first and second most mutated in the family
60	P53 tumour suppressor family	P53	P63	P73	13 of 14
177	cyclic nucleotide-gated ion channels	HCN1	HCN4	HCN2, HCN3	10 of 10
309	receptor tyr phosphatases	PTPRD	PTPRS	PTPRF	nine of nine
904	low-density lipoprotein receptors	LRP1B	LRP2	LRP1	nine of nine
724	N-acetylglucosaminyltransferases	MGT4C	MGT4A	MGT4B	eight of eight
1621	bHLH transcription factors	NPAS3	SIM1	NPAS1, SIM2	seven of eight
66	Ca^2+^-binding cadherin-like	CSTN2	CSTN1	CSTN3	seven of seven
170	very long chain acyl-CoA synthetases	S27A6	S27A2, S27A3		seven of seven for first; others equal
219	phosphatidylserine receptors	BAI3	BAI1	BAI2	seven of seven
289	RNA-binding proteins	RALYL	RALY	HNRPC, HNRCL	seven of seven
689	Kv channel-interacting proteins	KCIP4	KCIP1	CSEN, KCIP2	seven of seven
785	EGF receptor family	ERBB4	EGFR	ERBB2, ERBB3	seven of seven
1117	Discs large homologues	DLG2	DLG1	DLG3, DLG4	seven of seven
1547	transmembrane proteins	TM14B	TM14C	TM14A	seven of seven for first; five of seven for second
1706	autism susceptibility	AUTS2	FBSL	FBRS	six of seven
1727	engulfment and cell motility proteins	ELMO1	ELMO2	ELMO3	seven of seven for first; six of seven for second
61	Kv channel subunits	KCAB1	KCAB2	KCAB3	six of six
89	synaptophysin-like proteins	SYNPR	SYPL1, SYPL2, SYPH		six of six for first, others equal
157	leucine-rich repeat proteins	LRRC7	LAP2	SCRIB, LRRC1	six of six for first, five of six for second
246	choline transporter-like	CTL5	CTL2	CTL4	six of six for first, five of six for second
277	Kv channel subunits	KCND2	KCND3	KCND1	six of six
482	liprin family	LIPA2	LIPA1	LIPA3, LIPA4	six of six
725	guanine exchange factors for ARF GTPases	PSD3	PSD2	PSD1, PSD4	six of six for first, three of six for second
948	protein kinase D family	KPCD1	KPCD3	KPCD2	six of six
1327	Dickkopf-related, Wnt antagonists	DKK2	DKK4, DKK1		six of six for first, others equal
2105	C2-containing calcium sensors	RP3A	DOC2A	DOC2B	six of six
2215	type II cdc42-interacting protein kinases	PAK7	PAK4, PAK6		six of six for first, others equal
2275	RNA-binding splicing regulators	RFOX1	RFOX3	RFOX2	six of six for first, five of six for second
28	zinc transporters	ZNT8	ZNT4	ZNT2, ZNT3	five of five for first, four of five for second
47	histone lysine N-methyltransferases	SMYD3	SMYD1	SMYD2	five of five for first, three of five for second
139	PI 3-kinase regulatory subunits	P85A	P85B	P55G	five of five for first, four of five for second
287	muscarinic acetylcholine receptors	ACM2	ACM3	ACM1, ACM5	four of five for first, four of five for second
479	a synaptotagmin family	SYT1	SYT2	SYT5	five of five
515	hypoxia-inducible prolyl hydroxylases	EGLN3	EGLN1	EGLN2	five of five
525	protein kinase B family	AKT3	AKT2	AKT1	five of five for first, four of five for second
649	single-stranded DNA/RNA interacting	RBMS3	RBMS1	RBMS2	five of five
806	type II histone deacetylases	HDAC9	HDAC4	HDAC5, HDAC7	five of five
872	integrin alpha family	ITA8	ITAV	ITA2B, ITA5	five of five for first, four of five for second
882	accessory to TGFbeta assembly	LTBP1	LTBP2	LTBP3, LTBP4	five of five
941	serotonin receptors	5HT2C	5HT2A	5HT2B	five of five
1085	diacylglycerol kinases	DGKB	DGKG	DGKA	five of five
1443	zinc-finger DNA-binding proteins	ZMAT4	ZMAT1	ZN346	five of five for first, three of five for second
1996	localization of receptors and ion channels	LIN7A	LIN7C	LIN7B	four of four for first, three of four for second
106	voltage-gated calcium channel subunits	CAC1C	CAC1D	CAC1S, CAC1F	four of four
140	Rab3 GTPase in exocytotic vesicle fusion	RAB3C	RAB3B	RAB3D, RAB3A	four of four for first, three of four for second
193	transcription factors	RUNX1	RUNX2	RUNX3	four of four
196	regulator in Ras signalling pathway	CNKR2	CNKR3	CNKR1	four of four for first, three of four for second
254	deubiquitylating enzymes	OTU7A	OTU7B	TNAP3	four of four
386	dynamin vesicle trafficking proteins	DYN3	DYN1, DYN2		four of four for first, others equal
541	RNA-splicing protein	CELF4	CELF5	CELF3, CELF6	four of four for first, three of four for second
632	poly(rC)-binding proteins	PCBP3	PCBP2	PCBP1, PCBP4	four of four
915	RNA-binding zinc-finger proteins	Z385D	Z385B	Z385A	four of four
1103	cell adhesion molecules	CD166	MUC18	BCAM	four of four for first, two of four for second
1909	exocytosis, regulated by diacylglycerol	UN13C	UN13B	UN13A	four of four for first, two of four for second
2301	vesicle regulators alcohol dehydrogenase family	VAT1L	ZADH2	VAT1	four of four
2377	histone demethylases	KDM6A	UTY	KDM6B	four of four for first, three of four for second

We checked whether any of the genes with low ML scores in these 322 families had recurrent mutations in the same position, indicative of potential drivers. However, there were few repeated mutations in these low-ML genes.

### 3.4. A distinct group of cancers in which p53 is the most mutated member of the p53/63/73 family has low connectivity to other mutation load-skewed families

The ML skew characteristics of cancers in different regions of figure 3 are summarized in table 2. Interestingly, a distinct cluster of cancers at the bottom of the graph is characterized by a high ML skew in the p53/63/73 family (red node on figure 3). Green nodes are relatively sparse in this region of the graph, meaning that these cancers have ML skews in relatively few other 2R-ohnologue families. Furthermore, the ML-skewed families associated with this cancer cluster are generally not shared by cancers in the upper region of the graph.

**Table 2.** Characteristics of cancers in different regions of the VisANT graph in figure 3. In this table, cancers are loosely clustered according to the indicated characteristics. The data for mutations in p53, p63 and p73 for all cancers (from electronic supplementary material, table S2) are summarized in the electronic supplementary material, figure S5. Note that for all cancers with at least 10 mutations in at least one member of the p53/p63/p73 family, p73 always carries the lowest number of mutations in this dataset (electronic supplementary material, figure S3).

position on graph in figure 3	cancers in cluster	connected to ML-skewed families	above-threshold ML skew within p53/63/73 family?
top and sides	melanoma, B-cell lymphoma, breast, CLL, liver, lung adenocarcinoma, pancreatic, stomach, uterus	highly connected. Each cancer has both unique and shared ML-skewed families	no: melanoma, liver and B-cell lymphoma have most mutations in p63, while the other cancers have p53 as the most mutated protein, but these trends are below the skew thresholds for these cancers to be linked to the p53/p63/p73 in figure 3
centre	medulloblastoma	highly connected, with 120 ML-skewed families shared with other cancers and 20 ML-skewed families unique to medulloblastoma	yes: p63 is the most mutated member of this family
bottom	ALL, AML, bladder, colorectal, esophageal, glioblastoma, glioma (low grade), head-and-neck, lung squamous, ovary, prostate, kidney chromophobe	relatively few ML-skewed families are linked to each cancer in this cluster	yes: p53 is the most mutated member of this family
unconnected	cervical, kidney papillary, kidney clear cell carcinoma, myeloma, thyroid	unconnected to any ML-skewed families	no: cancers have fewer than nine mutations in p53/p63/p73 family in this dataset
	neuroblastoma	only one ML-skewed family, namely ALK/LTK (ALK most mutated, including well-known mutations)	no: only one p53 mutation and two p63 mutations in dataset
	pilocytic astrocytoma	connected to only three ML-skewed families, two of which are also highly connected to other cancers (LRP1B-most mutated, PTPRD-most mutated), the third being the Raf family (B-Raf most mutated)	no: only two mutations in the p53/p63/p73 family in dataset; one in p63 and one in p73

The p53 gene has a higher ML than p63 and p73 in most of the cancers in this lower cluster, namely acute myeloid leukaemia (AML), acute lymphoblastic leukaemia (ALL), bladder, colorectal, esophageal, glioblastoma, low-grade glioma, head-and-neck, chromophobe renal cell carcinoma, small cell lung carcinoma and lung squamous cancers (electronic supplementary material, figure S3; figure 3). However, the p53/63/73 family was one of only seven 2R-ohnologue families whose ML skew switched according to cancer type, and p63 has a far higher ML than p53 and p73 in medulloblastoma (electronic supplementary material, table S3 and figure S3). Medulloblastoma has a low incidence of p53 mutations associated with poor prognosis in subtypes with activated Wnt signalling and polyploid cases with activated hedgehog signalling, but p53 mutations are generally not in Group 3 and 4 subtypes [28–30]. However, p63 mutations were found in every medulloblastoma subtype (electronic supplementary material, table S4).

Cancers in the upper region of figure 3, such as melanoma, breast and liver cancer, are not linked to the p53/63/73 family node because, while these cancers can carry various mutations in p53 and p63, they do not display a strong ML skew in this gene family (table 2; electronic supplementary material, figure S3).

### 3.5. The p53/63/73 and FOG1/2 mutation load-skewed families together link to 22 of the 30 cancers examined

**Figure 4.**
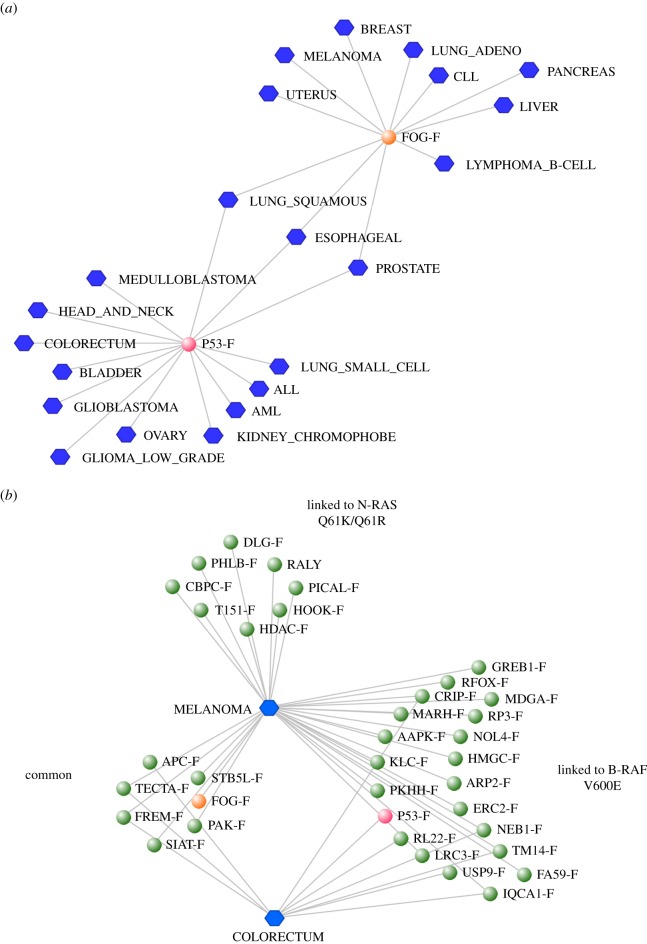
Associations between specific cancers and specific mutation-load-skewed 2R-ohnologue families. (*a*) Data extracted from figure 3, showing the cancers for which there are ML skews in the p53/p63/p73 and FOG1/FOG2 protein families. (*b*) A graph showing those 2R-ohnologue families that display a skewed ML in the cumulative data from those melanoma and colorectal cancer samples that have either a B-Raf^V600E^ or N-Ras^Q61K/R^ mutation (data in the electronic supplementary material, table S5). ‘Common’ indicates 2R-ohnologue families that have a skewed ML in the data from both the B-Raf^V600E^-mutated and the N-Ras^Q61K/R^-mutated samples.

The p53/63/73 family has an ML skew in 14 cancer types (red nodes in figures 3 and 4*a*). Also linked to many cancers is the FOG1/2 (Friend of GATA 1/2) family in which FOG2 is more commonly mutated than FOG1 (orange node on figures 3 and 4*a*; electronic supplementary material, table S3). Between them, the p53/63/73 and FOG1/2 ML-skewed families link to 22 of the 30 cancers examined, with an overlap of only three cancer types (figure 4*a*). Other 2R-ohnologue families displaying high ML skews in 10 or more cancers are those with the following proteins most mutated: potassium/sodium hyperpolarization-activated cyclic nucleotide-gated channel 1 (HCN1), tectorin alpha (TECTA), receptor-type tyrosine-protein phosphatase delta (PTPRD), low-density lipoprotein receptor-related protein 1B (LRP1B), MAM domain-containing glycosylphosphatidylinositol anchor protein 2 (MDGA2) and protein phosphatase 1 regulatory subunit 9A (neurabin-1) (electronic supplementary material, table S3).

### 3.6. 2R-ohnologue families with skewed mutation loads in cancer samples that carry activating driver mutations in B-Raf and N-Ras

We hypothesized that cancers with drivers that activate different signalling pathways may depend on different subsets of non-mutated 2R-ohnologue effectors. Therefore, from the 7042 samples in the dataset [15], we selected those with activating mutations in either B-Raf or N-Ras (electronic supplementary material, table S5). Generally, these two mutations are mutually exclusive. A subset of skewed-ML families co-occurred with either B-Raf^V600E^ or N-Ras^Q61K/R^ in melanoma (figure 4*b*). Of these, a smaller subset of families had ML skews in both the Raf- and Ras-mutated melanoma samples; these included the type II PAK kinases (PAK7 most mutated) and FOG1/2 family (FOG2 most mutated) (figure 4*b*; electronic supplementary material, table S5).

By contrast, in colorectal cancer the p53 family was one of seven families whose ML skew (p53 most mutated) coincided with B-Raf^V600E^ mutations, while the adenomatous polyposis coli family (APC more mutated than APC2) had ML skews in colorectal cancers with either B-Raf^V600E^ or N-Ras^Q61K/R^ (figure 4*b*). Thus, our unbiased analysis rediscovered the well-known dominance of APC driver mutations in these tumours, which can also acquire Ras and p53 driver mutations as they progress [31]. We note that as well as the V600E driver mutation, B-Raf acquired 107 different mutations in the colorectal cancer samples (electronic supplementary material, table S3). Finding that many different B-Raf mutations are allowed to occur in colorectal cancer (as well as the fact that APC mutation dominates) is consistent with findings that B-Raf^V600E^ inhibitors are generally not therapeutically useful in this tumour type [31–33].

### 3.7. The relative mutation load of a 2R-ohnologue is moderately predictive of its altered mRNA expression in melanoma

**Figure 5.**
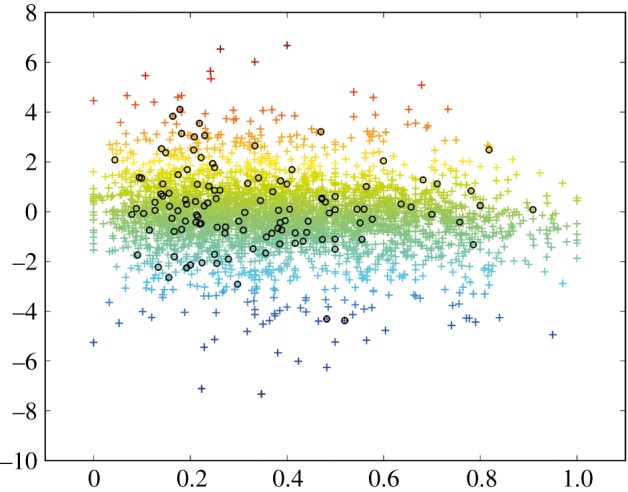
Relationships among ML of 2R-ohnologues in melanoma, cancer/control mRNA expression in melanoma and proteins identified in 14-3-3-affinity capture experiments using melanoma cell lysates. Each cross represents a gene in the E-GEOD-3189 transcription profiling dataset [34]. The log_2_ ratio of mRNA expression in malignant melanoma versus benign melanocytic lesions in the E-GEOD-3189 dataset is plotted on the *y*-axis against the ML score of the gene calculated from the Alexandrov *et al.* [15] data on the *x*-axis. The genes whose mRNA levels are most strongly up- or downregulated in melanoma are in red and blue, respectively. Also plotted (circles) are the proteins that were isolated by 14-3-3-affinity capture of cell lysates from both SKMEL13 and SBCL2 melanoma cells, and identified by mass spectrometric analyses. The data used for this figure are in the electronic supplementary material, table S6.

An important question is whether the 2R-ohnologues that are relatively free of mutations are expressed in the cancers. While we cannot answer this question for the Alexandrov *et al.* [15] dataset, other available data record differences in mRNA levels between samples of malignant cancers compared with benign or normal controls. We found a moderate, but statistically significant tendency for 2R-ohnologues with low ML scores (mutation-free relative to sister 2R-ohnologues) to have their mRNA levels strongly upregulated in melanoma in the E-GEOD-3189 dataset [34] (figure 5; electronic supplementary material, figure S4 and table S6). A similar trend was observed in the E-GEOD-32867 dataset, which reports gene expression levels in lung adenocarcinoma relative to adjacent non-tumour tissue (electronic supplementary material, figure S5 and table S7). These results indicate the existence of selection pressures that affect the expression levels of certain 2R-ohnologues in cancer cells. The 2R-ohnologues whose expression is altered in the cancer have a greater tendency to be maintained with a low ML.

### 3.8. 2R-ohnologues with low mutation loads can be isolated by 14-3-3-affinity capture from lysates of melanoma cells

We also wished to assess whether 2R-ohnologues with low MLs are expressed as proteins in the relevant cancers. To this end, we took advantage of the fact that Ras–Raf and certain other oncogenic signalling pathways stimulate the phosphorylation of many proteins that consequently dock onto the phosphoprotein-binding 14-3-3 proteins [35–37]. Moreover, the 14-3-3-binding phosphoproteome is enriched in 2R-ohnologues [7,38–41].

14-3-3-affinity capture was therefore used to isolate (phospho)proteins from lysates of SKMEL13 melanoma cells that carry B-Raf^V600E^ and also SBCL2 N-Ras^Q61K^ melanoma cells (electronic supplementary material, figure S6, and tables S8 and S9). Of the 1007 proteins that were 14-3-3-affinity captured and identified in a total of two experiments from both cell lysates, 68 were previously reported 14-3-3-binding phosphoproteins (gold standards), and 480 proteins were 2R-ohnologues. Of these, 62 had ML scores of less than 0.2 in the melanoma dataset of 2R families in which at least one member had 10 or more mutations (electronic supplementary material, table S10). These results indicate that at least some 2R-ohnologues with low ML scores are expressed as proteins in relevant cancer cells.

Further analysis identified candidate ‘lynchpin’ 14-3-3-binding phosphosites in 286 of the melanoma 14-3-3-affinity captured 2R-ohnologues [42]. Lynchpins are 14-3-3-binding phosphosites whose positions are conserved across members of a given 2R-ohnologue family [7]. From the overall analysis, we assembled a stringent list of 235 known and candidate 14-3-3 binding partners (electronic supplementary material, table S10). Interestingly, 143 proteins from this stringent list could be mapped to the E-GEOD-3189 melanoma transcriptome dataset and were moderately overrepresented among 2R-ohnologues with low ML scores (figure 5). Specifically, 111 of these 143 (78%) 14-3-3-affinity captured proteins were among the 66% of this dataset that had ML scores of less than 0.5 (electronic supplementary material, table S6). In summary, these pilot experiments indicate the potential of 14-3-3-affinity capture for isolating 2R-ohnologue proteins to dissect their biochemical regulation in cancers.

### 3.9. Overexpression of protein kinase 2R-ohnologues with low mutation load scores decreased the sensitivity of B-Raf^V600E^-mutant melanoma cells to PLX4720

One further observation is consistent with the notion that 2R-ohnologues with low ML scores are functionally relevant for cancer: Wood *et al.* [43] screened for protein kinases whose overexpression rendered B-Raf^V600E^-melanoma cells resistant to the B-Raf^V600E^ inhibitor, PLX4720. We noted that 2R-ohnologue protein kinases with low ML scores in melanoma (this study) tended to be better at rendering cells resistant to PLX4720 [43], compared with their sisters that have high ML scores (table 3). The trend of ‘highest viability score = lowest ML score’ was observed even within protein kinase families that display moderate ML skews in melanoma (table 3). This finding suggests that the protein kinases with low ML scores may contribute to melanoma progression by a mechanism that is linked to B-Raf^V600E^.

**Table 3.** Overexpression of protein kinase 2R-ohnologues with low mutation scores decreased the sensitivity of B-Raf^V600E^-mutant melanoma cells to PLX4720. The viability score assigned by Wood *et al.* [43] refers to the ability of the protein kinase, when overexpressed, to enhance the viability of B-Raf^V600E^-mutant melanoma (A375) cells exposed to the B-Raf^V600E^ inhibitor, PLX4720. Seven 2R-ohnologue families of protein kinases had least one member among the top hits of the Wood dataset [43] and at least one member with at least 10 mutations in melanoma in the Alexandrov dataset [15]. The cell viability scores [42] and ML scores (this study) are shown for each member of these seven families. The family Id is an arbitrary number assigned to identify each 2R-ohnologue family in [7]. n.a., data not available.

2R-ohnologue family Id	protein name	UniProt Id	viability score [42]	ML score in melanoma (electronic supplementary material, table S2)	is ML skew in melanoma above threshold for inclusion in figure 3 and the electronic supplementary material, table S3?
378	NTRK2	Q16620	1.23	0.0777	no
378	NTRK1	P04629	1.07	0.3932	no
378	NTRK3	Q16288	n.a.	0.2621	no
378	MUSK	O15146	0.76	0.2670	no
1058	MST1R	Q04912	1.13	0.2923	no
1058	MET	P08581	1.03	0.7077	no
1085	MAPK8	P45983	1.18	0.1563	no
1085	MAPK9	P45984	0.93	0.3438	no
1085	MAPK10	P53779	0.79	0.5000	no
1548	SRPK3	Q9UPE1	1.16	0.2195	no
1548	SRPK1	Q96SB4	0.95	0.3415	no
1548	SRPK2	P78362	0.86	0.4390	no
1666	PIM2	Q9P1W9	1.15	0.3182	no
1666	PIM1	P11309	1.11	0.5000	no
1666	PIM3	Q86V86	n.a.	0.1818	no
1780	LIMK1	P53667	1.17	0.3103	no
1780	LIMK2	P53671	0.94	0.6897	no
2215	PAK6	Q9NQU5	1.21	0.0877	yes
2215	PAK4	O96013	0.97	0.0789	yes
2215	PAK7	Q9P286	0.86	0.8333	yes

## 4. Discussion

Here, knowledge of the evolutionary history of the human genome was used to unlock patterns in a heterogeneous dataset of somatic mutations from many cancers. Our findings cannot be accommodated within the conventional binary driver/passenger model [16,32].

In every cancer type examined, somatic mutations are more prevalent in 2R-ohnologues than in non-ohnologue genes. Because 2R-ohnologues are enriched in signalling proteins [6,7], this finding is consistent with cancer being a disease in which regulatory processes go awry.

A second finding was that in a subset of cancers—particularly melanoma, lung adenocarcinoma, breast and liver cancers, B-cell lymphoma and medulloblastoma—there are 2R-ohnologue families in which one gene carries multiple mutations in the cumulative data, while sister genes in the same family are relatively mutation-free. Most notably, in 315 out of the 322 2R-ohnologue families displaying a high skew in multiple cancers the *same gene* carries the heaviest ML in each cancer, and for families of more than two members, usually the second-ranked gene is also the same in each cancer.

Generally, each gene in a 2R-ohnologue family is on a different chromosome, and the non-mutated 2R genes in the ML-skewed families are widely distributed in the genome. It therefore seems unlikely that all of these are in genomic regions that are protected from mutation, though this possibility must be formally tested.

**Figure 6.**
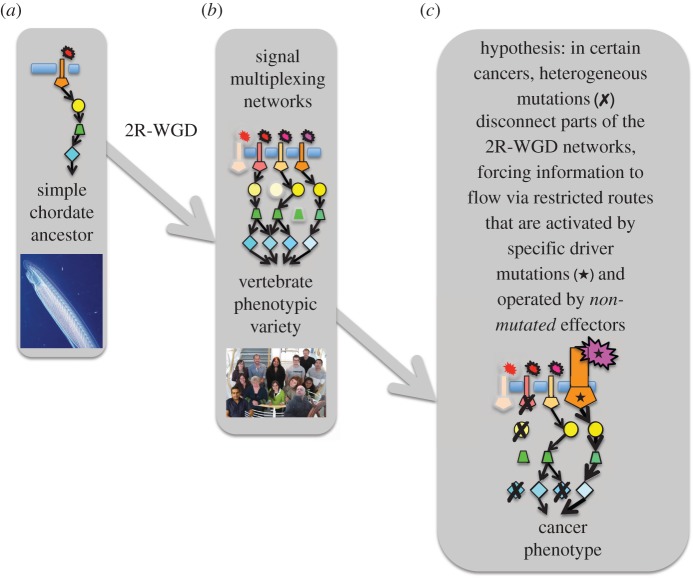
Simplified model that depicts cancer as a disorder of signal multiplexing in the cellular 2R-WGD networks of vertebrate animals. (*a*) The ancestor of all the vertebrates was an invertebrate chordate whose cells are depicted as being under the control of simple linear regulatory pathways. The image is of amphioxus (*Branchiostoma*), regarded as the best modern-day proxy for the ancestor. (*b*) 2R-WGD at the evolutionary origins of the vertebrate animals boosted communication networks inside our cells. Variations in these networks may underpin variety of vertebrate cell types, species and behaviours. (*c*) We hypothesize that certain cancers arise when different heterogeneous combinations of mutations (crosses) disconnect certain parts of the 2R-WGD regulatory networks and force too much communication flow via a restricted number of oncogenic pathways. These ‘open’ oncogenic pathways are activated by specific driver mutations (stars) and also require effector proteins that must remain mutation-free. If these effectors acquire too many deleterious mutations, the cancer cell will be lost. Though the model only depicts 2R families with high ML skews, it could be extended to include other patterns. For example, 2R families whose members carry an even ML may be in parts of the network where any member can perform the family function for the cancer, or represent functions whose total elimination gives a selective advantage to the cancer. In its simple form, the model assumes that when genes are hit by a number of different mutations (crosses) these will include loss-of-function mutations. However, it is appreciated that this may not always be so, in which case the rules of the model would change. The model highlights that the contribution of both mutated and non-mutated 2R-ohnologues to the overall function of each family in the cancer should be considered.

Rather, we favour a conceptually simple working model (figure 6). The invertebrate ancestor of the vertebrates is depicted as having cells controlled by linear regulatory pathways. Via the 2R-WGD leap, these pathways were quadruplicated, generating the complex networks that transmit multiple regulatory signals in vertebrate cells. In certain cancers such as melanoma and breast cancer, heterogeneous patterns of multiple mutations (black crosses in figure 6) result in the shutdown of certain routes through these communication networks. Information flow is therefore forced through a limited number of ‘open’ network pathways, which are driven by activating oncogenic driver mutations (star symbols in figure 6) and also depend on specific non-mutated 2R-ohnologues as effectors.

In this model, driver mutations that activate the ‘open’ oncogenic pathways will be under positive selection, whereas essential effectors of these drivers will be under purifying selection to be maintained mutation-free. By contrast, 2R-ohnologues in the ‘shut down’ part of the signalling network will experience different selection pressures that vary with time. Our findings indicate quantitative hierarchies in how mutation burdens are distributed across 2R-ohnologue families in cancers that conform to our model. We therefore suggest that mutations that block tumour-suppressing pathways through the network will be under positive selection until these routes are closed, at which time these pathways will join the ones that are irrelevant to the cancer, where further mutations will be under neutral selection.

The heterogeneity of so-called passenger mutations is generally taken to imply that these represent clinically inconsequential ‘noise’. However, in our model such heterogeneity reflects the possibility that there are multiple ways to disable parts of the 2R-WGD networks, while still leaving the oncogenic pathways open. These findings raise challenging questions of redundancy and essentiality of family members at the biochemical level. Why are certain 2R genes quantitatively more important to a cancer than their sisters? As more cancer mutation data are collected, it will be fascinating to see how the overall ML rankings resolve.

Through this study, we realize that recurrent oncogenic driver mutations, genes with multiple mutations and non-mutated genes can each occur within the same 2R-ohnologue family, or even in the same gene, in different cancer contexts. For example, neuroblastoma presented with only one ML-skewed gene family (the ALK/LTK receptor tyrosine kinases), which includes driver mutations known to activate ALK in this disease [44]. However, the cumulative data for lung adenocarcinoma, breast and liver cancers contain a few hundred different mutations in ALK, but few in its sister LTK, an imbalance that is inconsistent with ALK being an activated driver in these cancers.

An important question arising from our model is whether the mutation-free 2R-ohnologues that are putative effectors of oncogenic drivers will be good targets for therapeutic intervention. While a logical proposition, unfortunately these mutation-free genes are largely understudied. For example, there is intense focus of research on the inactivating mutations in APC in colorectal cancers [31]. However, it is conceivable that the loss of APC renders these cancers dependent on APC2, and to our knowledge this hypothesis has not been tested. Similarly, many FOG2 mutations, but few in FOG1, were found in 11 cancer types. Therefore, targeting FOG1 for inactivation seems logical. Furthermore, the FOG family (FOG2 most mutated) was among several 2R families whose skewed MLs coincided with activating B-Raf^V600E^ and N-Ras driver mutations in melanoma. Targeting aberrant Raf signalling is therapeutic in melanoma [31,33], so it will be important to define the biochemical relationships between Ras–Raf signalling and FOG proteins in melanoma. Interestingly, FOG1 interacts with the GATA transcription factors, which include GATA2, which is essential for proliferation of cancer cells carrying oncogenic K-Ras [45]. The type II PAK kinases (PAK7 most mutated) also showed a ML skew in melanomas with B-Raf^V600E^ and N-Ras driver mutations. Furthermore, PAK4 and PAK6 have much lower MLs than PAK7 in six cancers; PAK4, PAK6 and PAK7 are 14-3-3-binding phosphoproteins [42,46]; PAK4 was 14-3-3-affinity captured here from melanoma cell lysates; and overexpressed PAK4 and PAK6 were more effective than PAK7 in enhancing the viability of B-Raf^V600E^-mutant melanoma cells exposed to PLX4720 [43]. These findings suggest that, for unknown reasons, the PAK4/PAK6 kinases are more important than PAK7 in melanoma.

STRING analysis [47] revealed that the protein families with ML skews in multiple cancers include regulatory proteins in growth factor signalling (families whose most multiply-mutated members are ERBB4, AKT3, GSK3B, ALK, KPCD1, PAK7, KCC1D, AAKG2, KPCT, FER, PTPRD, LIPA2, PTPRR, P2R3A, P85A, P3C2G, INP4B, PPM1H, IRS1, GRB2, RHEB, OTU7A), chromatin function (FOG2, RUNX1, ANM8, TREF1, SMYD3, HDAC9), membrane and cytoskeletal dynamics (UN13C, ELMO1, RAB3C, RB11A, TSNA1, RBG1L, MTUS2, RASF9, GAS2, RHG06, RRAS2, EXT1, SYT1), neurotransmission (AUTS2, DLG2, AMMR1, HIP1, CBLN2, NPAS3, CABP8, BAI3, DAB1, SYNPR, DCC, 5HT2C, LIN7A, CRUM1, ACM3, neurabin-1), ion channels (HCN1, KCIP4, KCAB1, KCND2), protein glycosylation (XYLT1, MDGA2, MGT4C, LARGE), extracellular matrix (TECTA, FREM2), angiogenesis and responses to hypoxia (BAI3, EDIL3, EGLN3). While the most mutated family members are listed here for linguistic ease, we propose the least-mutated members for attention as potential therapeutic targets. Many of these protein families have known functional links with cancer and metastases, but this dataset is also rich in ion channels and neurotransmitter systems; this is intriguing, since most of the analysed cancers are not in excitable nervous tissues. Interestingly, tumours exhibit bioelectric changes and ion channel modulation is being explored for cancer therapy [48,49].

A distinct subset of cancers (ALL, AML, bladder, colorectal, glioblastoma, glioma, head-and-neck, ovary, kidney chromophobe) was characterized by an ML skew towards mutations in p53, and not p63 or p73. These cancers have relatively few other 2R-ohnologue families with high ML skews, and the ones that they did have generally differed from the ML-skewed families in breast, melanoma, lung adenocarcinoma and liver cancers. These ‘p53-mutated, but not p63/p73-mutated’ cancers may therefore require a tailored version of the model (figure 6) to be developed.

Finally, it has been considered paradoxical that ‘dangerous’ cancer genes from the 2R-WGD were selectively retained during vertebrate evolution [11]. An alternative view is that the robustness of 2R-ohnologue signal-multiplexing networks enables vertebrates to survive long enough to develop cancer. Mutating one 2R-ohnologue may not be lethal if other members of the family can at least partially compensate. However, if such mutations disconnect that protein family from certain signalling networks, cells will get stuck in one signalling mode, generating the cancer phenotype. It is fascinating to discover how an ancient evolutionary leap left its mark on the genomes of vertebrate animals, on signalling complexity and on the diseases of modern humans.

## 5. Material and methods

### 5.1. Mapping mutations to genes and computing mutation load scores of 2R-ohnologues

The chromosomal locations of single and double nucleotide substitutions, small insertions and deletions within the Alexandrov *et al.* [15] dataset were mapped onto nucleotide positions within protein-coding and pseudogene-RNA genes in the Ensembl 72 dataset. Briefly, the BioMart service of Ensembl (www.ensembl.org) was used to retrieve the gene positions, and a Python script was compiled to identify the mutations that map within a given gene. Mutations involving more than one nucleotide were mapped to the first nucleotide change. Each mutation that maps onto the same starting nucleotide was considered only once. For example, for the B-Raf gene, the V600L and V600K amino acid substitutions start on the same nucleotide (1798G > C and 1798_1799GT > AA, respectively) and would count as one, whereas the V600E substitution starts one base after (1799T > A) and would be counted separately. Gene copy number changes and changes involving more than one gene were not considered. Genes annotated as pseudogenes were included in our analysis, because functions have been identified for many such genes.

The ML for each gene was computed by summing the total number of different mutations identified for a gene (PROT ML) divided by the sum of all mutations identified in the 2R-ohnologue family components of the gene under analysis. This number scores from 1 to 0 and identifies the genes in any 2R-ohnologue family that are prone to accumulate mutations (score close to 1) or are clear of mutations (score close to 0).

The genomic nucleotide positions of mutations were translated with a Python script into the transcript nucleotide position of a protein-coding gene for making pictures of the MLs of the different family members. The BioMart service of Ensembl was used to retrieve the transcript genomic coordinates, and the longest transcript for each gene was used for this analysis.

### 5.2. Lack of mutations: biological reality or missing data?

The mutation data were derived from 507 whole genome and 6535 exome sequencing studies, and further sequencing validation experiments performed by Alexandrov *et al.* [15], the Cancer Genome Atlas, International Cancer Genome Consortium and other laboratories [15]. For our study, it was critical that genes that record few mutations represent biological reality and not false negatives due to lack of sequence coverage. There is unevenness in the dataset because, while the exome enrichment platforms that were used (Agilent, Nimblegen and Illumina) capture mainly protein-coding regions of genes, the Illumina platform gives reads in 3′ and 5′ untranslated regions (UTRs) [50] (figure 2*b*; electronic supplementary material, data file S2). However, we did not exclude UTRs from our analysis, after checking that this would not have a general effect on which 2R-ohnologue families were designated to display an ML skew. Each exome sequencing platform misses certain ‘difficult’ regions [50]. However, with the possible exception of pseudogenes, the raw data in the primary studies cited in [15] give no indication of technical biases that would result in specific genes being systematically missed in the cumulative data.

### 5.3. Melanoma cell culture, 14-3-3-affinity chromatography and mass spectrometric identification of proteins

SKMEL13 (B-RAF^V600E^) and Sbcl2 (N-RAS^Q61K^) melanoma cells were cultured in RPMI and Dulbecco's modified eagle medium, respectively. Media were supplemented with 10% (v/v) fetal calf serum (Thermo Scientific), 2 mM l-glutamine, 50 units ml^−1^ penicillin G and 50 μg ml^−1^ streptomycin (Life Technologies), and cells cultured under 5% CO_2_ at 37°C. Lysates were prepared as in [51] and subject to 14-3-3-affinity chromatography. Briefly, proteins were bound to 14-3-3-Sepharose, and specifically bound proteins eluted by competition with the 14-3-3-binding synthetic phosphopeptide, ARAApSAPA, as in [35] except that the high salt wash was only 500 ml and the mock peptide elution was omitted. The eluted proteins were denatured in 4× LDS sample buffer (Life Technologies) containing 10% sample reducing agent at 70°C for 10 min, cooled and alkylated with 50 mM iodoacetamide for 30 min in the dark at room temperature. Proteins were separated on NuPAGE 4–12% gradient gels and stained with colloidal Coomassie Blue (Life Technologies). Gel lanes were cut into 20 sections (electronic supplementary material, figure S6), which were washed successively with 50 mM triethylammonium bicarbonate; 50% acetonitrile, 50 mM triethylammonium bicarbonate (twice); and acetonitrile (15 min each wash), before drying in a SpeedVac (Eppendorf). Trypsin (5 μg ml^−1^ trypsin gold; Promega) in sufficient 25 mM triethylammonium bicarbonate to cover the gel pieces was added for 12 h at 30°C. Supernatant was transferred to a fresh tube, to which two 50% acetonitrile washes of the gel pieces were also added. The digested samples were dried, and each digest was redissolved in 2 μl of 25 mM sodium acetate buffer, pH 5.5, 30 mM sodium cyanoborohydride containing 0.2% (v/v) formaldehyde and incubated at room temperature for 15 min. Tryptic digests were analysed using Ultimate 3200 nanoflow chromatography (LC Packings) coupled to an LTQ-Orbitrap (Thermo Finnigan) mass spectrometer equipped with a dynamic NanoSpray source (Optron). For protein identification, mass spectra were acquired using the LTQ-Orbitrap programmed to perform two FT scans (60 000 resolution) on 300–800 and 800–1800 amu mass ranges with the top five ions from each scan selected for LTQ-MS/MS. FT spectra were internally calibrated using a single lock mass (445.1200 atomic mass units). Raw files were converted to peak lists in Mascot generic format (MGF) files using raw2msm v. 1.7 software (Matthias Mann) using default parameters and without any filtering, charge state deconvolution or deisotoping. MGF files were searched using a Mascot 2.2 in-house server against the International Protein Index human 3.26 database (57 846 sequences; 26 015 783 residues).

## Supplementary Material

List of supplementary materials; supplementary figures S1 to S6; legends for supplementary tables and data files

## Supplementary Material

Data file S1

## Supplementary Material

Data file S2

## Supplementary Material

Supplementary tables S1 to S10
