# Supplementary material for: Identification of 2R-ohnologue gene families displaying the same mutation-load skew in multiple cancers
Source: Open Biol. 2014 May 7;4(5):140029. doi: 10.1098/rsob.140029 (PMC4042849; doi:10.1098/rsob.140029)
Supplement: List of supplementary materials; supplementary figures S1 to S6; legends for supplementary tables and data files [file rsob140029supp1.pdf]

**List of the Supplementary Materials**

**Supplementary Figures S1-S6 (All supplementary figures are within this document)**

Fig. S1. Statistical analysis for the mutation distributions in 2R-ohnologues versus non-ohnologues in Fig 1A.

Fig. S2. Comparison of the lengths of 2R-ohnologue and non-ohnologue genes.

Fig. S3. Distributions of mutations in p53, p63 and p73 genes respectively, for cancers depicted in Fig 3A and described in table 2.

Fig. S4. Statistical plot for the relationship between mutation loads (MLs) of 2R-ohnologues (this study) and expression levels of their transcripts in melanoma compared with non-malignant control samples (E-GEOD-3189).

Fig. S5. Relationships among mutation load of 2R-ohnologues, ratio of mRNA expression in lung adenocarcinoma compared with control samples.

Fig. S6. SDS polyacrylamide gels of proteins isolated by 14-3-3-affinity capture from lysates of SKMEL13 and SBCL2 melanoma cells.

**Supplementary Tables S1-S10 (All supplementary tables are within a separate single Excel file)**

Table S1. Numbers of mutations in 2R-ohnologue and non-ohnologue genes in 30 cancer types.

Table S2. Mutation scores and mutation loads (MLs) for all 2R-ohnologues in families of 2, 3 and 4 members.

Table S3. Mutation scores and mutation loads (MLs) for 2R-ohnologues in families that display ML skews above the specified thresholds.

Table S4. Medulloblastoma samples in the Alexandrov et al (2013) dataset are annotated according to their subtype (where known) and according to whether they carry mutations in p53 or p63.

Table S5. Mutation scores and mutation loads (MLs) for those 2R-ohnologue families that display ML skews in melanoma and colorectal cancers with B-RafV600E or N-RasQ61K/R mutations.

Table S6. 2R-ohnologues whose mRNAs are included in the E-GEOD-3189 melanoma transcription profiling dataset and whose proteins were captured by 14-3-3 in melanoma cell lysates.

Table S7. 2R-ohnologues whose mRNAs are included in the E-GEOD-32867 lung adenocarcinoma profiling dataset.

Table S8. Proteins captured by 14-3-3-affinity chromatography from lysates of SBCL2 (N-Ras$^{Q61K}$) melanoma cells.

Table S9. Proteins captured by 14-3-3-affinity chromatography from lysates of SKMEL13 (B-Raf$^{V600E}$) melanoma cells.

Table S10. 14-3-3-affinity captured proteins analysed in ANIA.

**Supplementary data files**

Data file S1. Mutation loads of 2R-ohnologue genes in different cancers.

Data file S2. Distributions of mutations within 2R-ohnologue genes in melanoma.

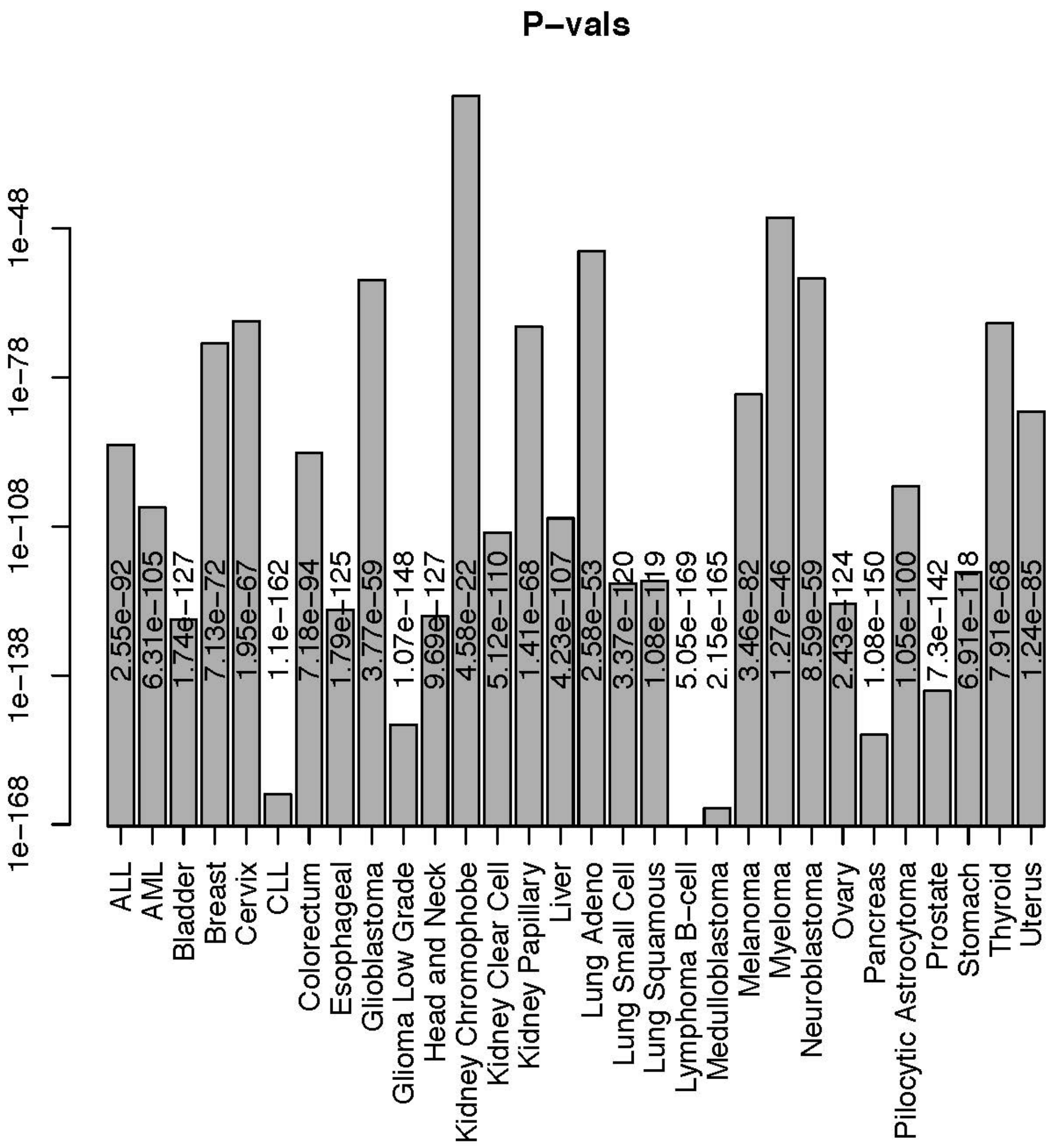


**Figure S1 - Statistical analysis for the mutation distributions in 2R-ohnologues versus non-ohnologues in Fig 1A.** The graph shows the p-values relative to the proportion of mutations identified in 2R-ohnologues versus non-ohnologues under a binomial model. The low p-values indicate that the preponderance of mutations in the 2R-ohnologues has high statistical significance.

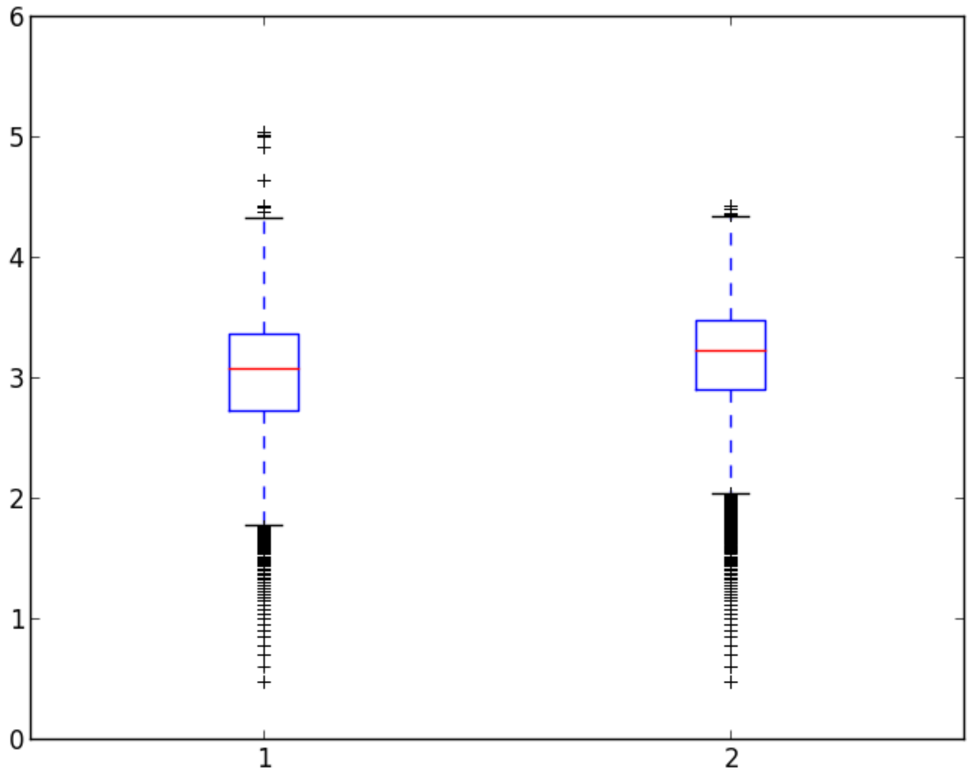


**Figure S2 - Gene length comparison between 2R-ohnologue and non-ohnologue.** The picture show the distributions of lengths of the 2R-ohnologue (1) and non-ohnologue (2) transcript coding genes. A gene length is defined by the log10 of the base pair length of the longest transcript. Red lines indicate the medians and blue boxes the interquartile ranges.

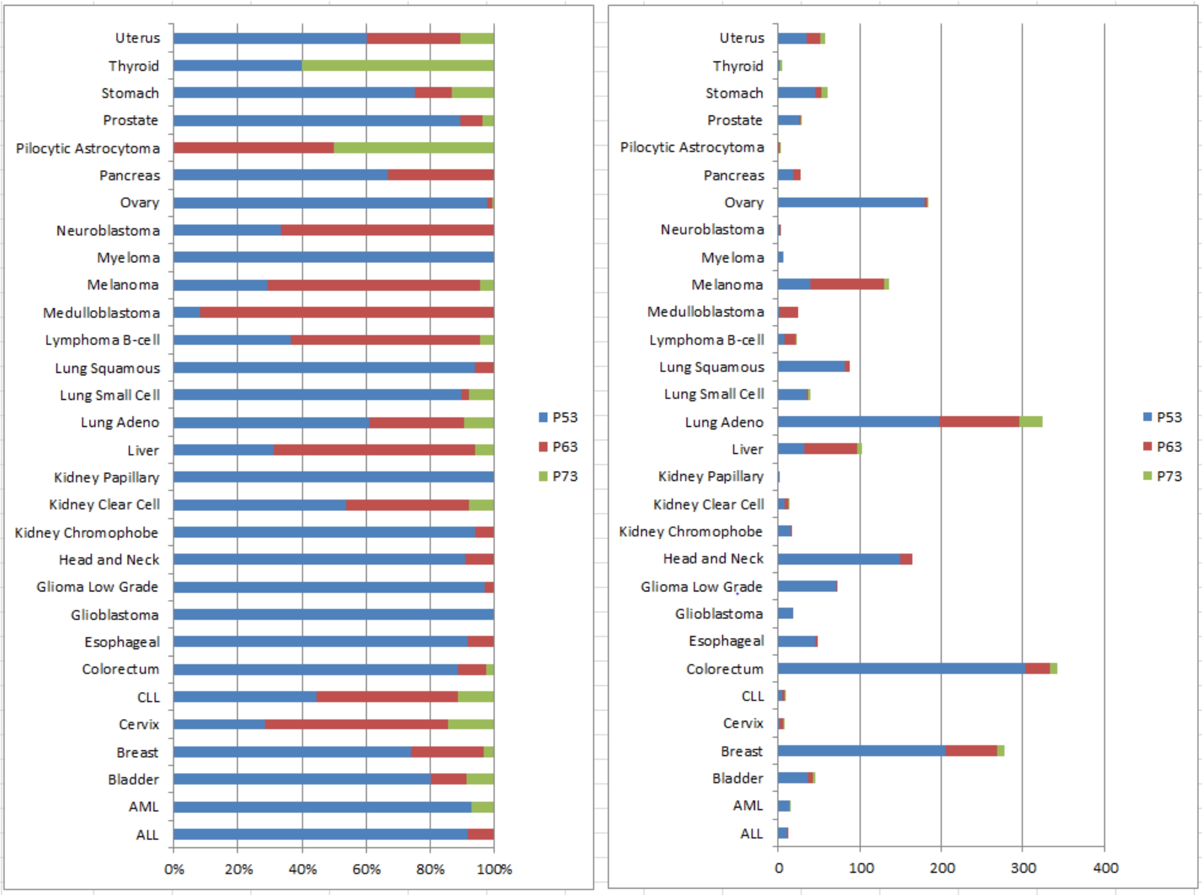


**Figure S3 - Distributions of mutations in p53, p63 and p73 genes.** The picture shows the percentage of mutations (left) and the absolute number (right) of mutations that map onto the p53 (blue), p63 (red) and p73 (green) genes for the cancers depicted in Fig 3A and described in Table 2.

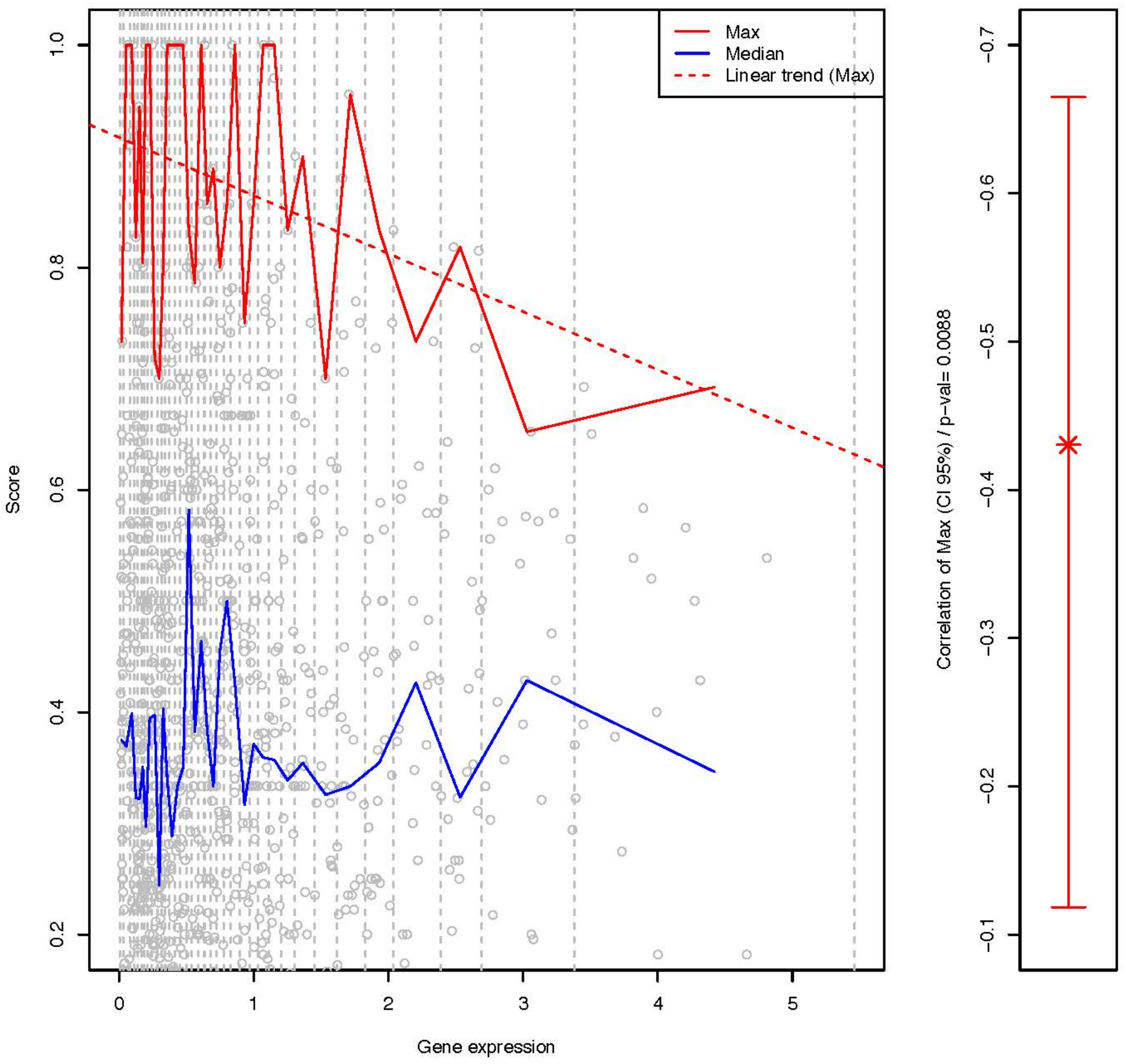


**Figure S4 - Statistical plot for the relationship between mutation load scores (score, y-axis) of 2R-ohnologues (this study) and expression levels of their transcripts in melanoma compared with non-malignant control samples (E-GEOD-3189) (gene expression, X-axis).**

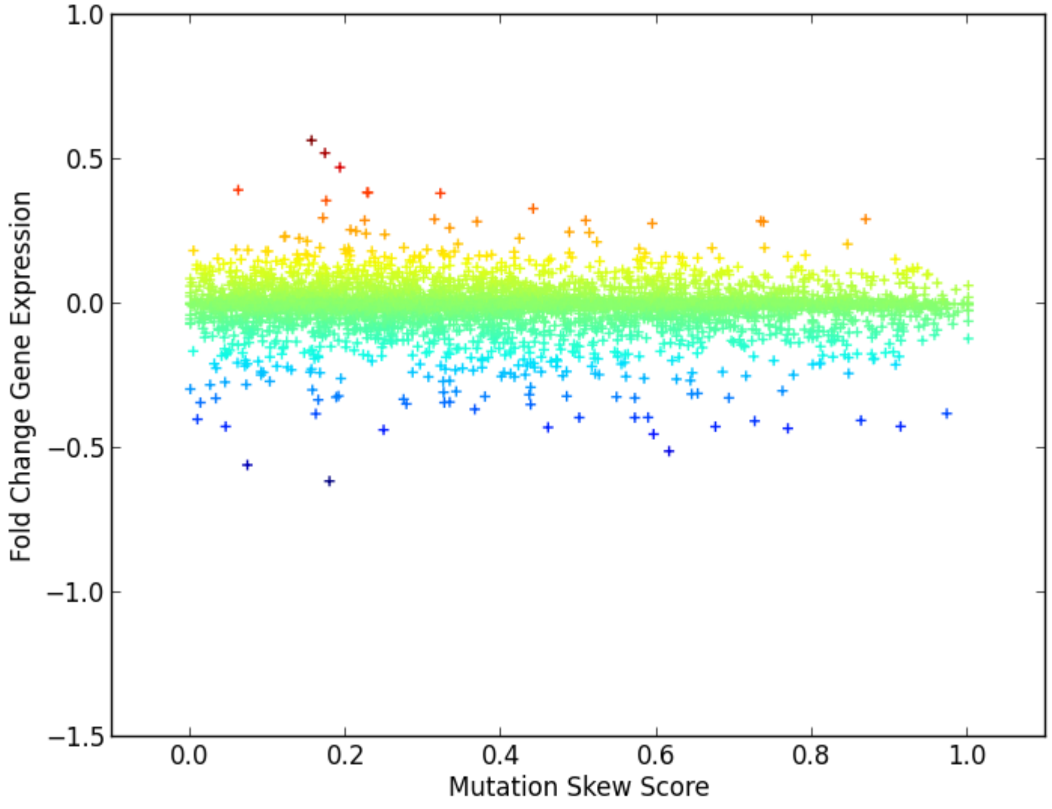


**Figure S5 - Relationships among mutation load of 2R-ohnologues, ratio of mRNA expression in lung adenocarcinoma compared with control samples.** Each cross represents a gene in the E-GEOD-32867 dataset transcription profiling dataset (www.ebi.ac.uk/arrayexpress/experiments/E-GEOD-32867). The log2 ratio of mRNA expression in lung adenocarcinoma versus adjacent normal tissue in the E-GEOD-32867 dataset is plotted on the y-axis against the ML score of the gene calculated from the Alexandrov data (2013) on the x-axis. The genes whose mRNA levels are most strongly up or down regulated in lung adenocarcinoma are in red and blue, respectively.

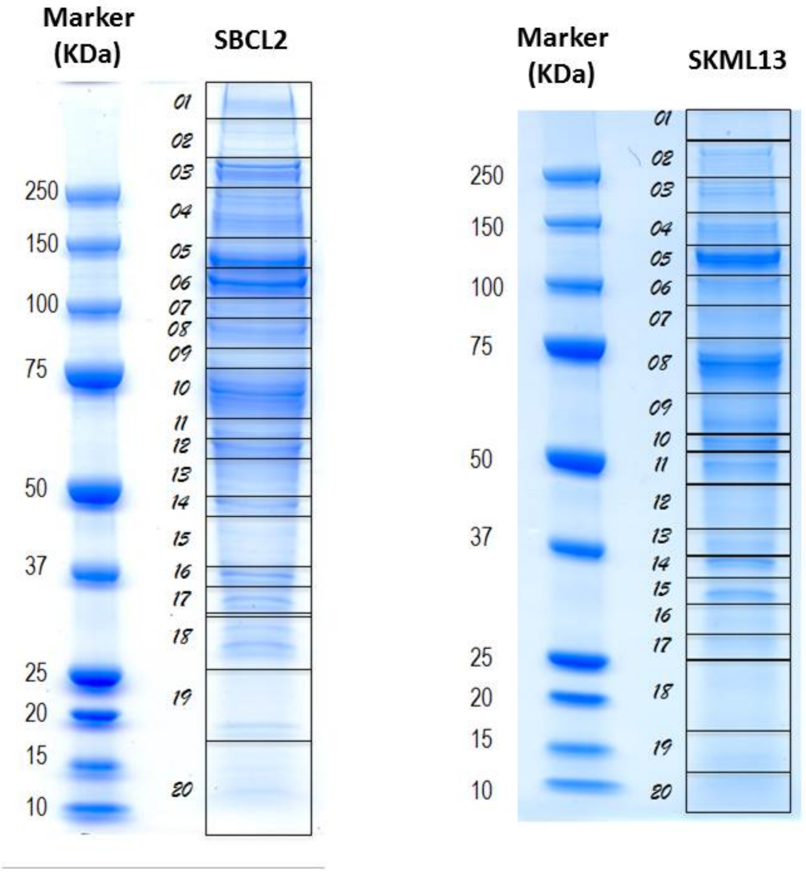


**Figure S6 - SDS polyacrylamide gels of proteins isolated by 14-3-3-affinity capture from lysates of SBCL2 (left) and SKMEL13 (right) melanoma cells.** Gels shown represent one experiment of two that performed for each cell type.

**Supplementary table legends (all supplementary tables are within a single Excel file)**

**Table S1 - Numbers of mutations in 2R-ohnologue and non-ohnologue genes in 30 cancer types.** The first column lists the Ensembl 72 gene identifiers. The second column contains a binary tag: 1 if the gene is annotated as a 2R-ohnologue, 0 if not. The columns C to AF report the numbers of mutations in each gene in each cancer type, as identified in Alexandrov et al (2013). Here, 'genes' include protein-coding genes and pseudogenes that are expressed as transcripts, and their length is defined as the length of the longest transcript.

**Table S2 - Mutation scores and mutation loads (MLs) for all 2R-ohnologues in families of 2, 3 and 4 members.** The first column (A) is the family ID, which is an arbitrary number assigned to identify each 2R-ohnologue family as in Tinti et al (2012). Column B is the Ensembl gene identifier and Column C the UniProt protein identifier. Columns from D to BK are in pairs: each pair represents a different cancer type with the number of mutations in the gene in that cancer in the first column and the mutation load skew in the second column and 'none' indicates no mutations in the gene in that cancer.

**Table S3 - Mutation scores and mutation loads (MLs) for 2R-ohnologues in families that display ML skews above the specified thresholds.** The table contains four columns. The first column is the cancer type. The second is the family ID (an arbitrary number assigned to identify each 2R-ohnologue family in Tinti et al (2012)). Column C contains information on the members of each family (geneID|UniProtID|UniProtName|number of mutations in each family member|skew score for the family for the cancer type named in column A) calculated from the data in Alexandrov et al (2013). In each box in column C, genes are listed in rank order according to their ML scores: the gene carrying the highest number of mutations for its family in the given cancer is listed first. Column D reports how many times the family is present in the dataset (that is, the number of cancer types in which the family displays a mutation load (ML) skew over the threshold values). To be included in this table, 2R-ohnologue protein families must present with ML skew threshold values of $\geq 0.9$ for families containing 2 genes (that is, one family member carries at least 90% of the mutated positions for that family), $\geq 0.8$ for families with 3 genes (one family members carries at least 80% of the mutated positions) and $\geq 0.7$ for families of 4 genes (one family member carries at least 70% of the mutated positions).

**Statistical note for Table S3:** A complete statistical analysis of the extent of skewedness of each family of 2R-ohnologues is beyond the scope of the current article, given the variable incidences of mutations in different cancers. However, we can state that: (a) the data in this table show that in 315 out of 322 families with an above-threshold skew in multiple cancers, the same gene carries the heaviest ML in each cancer, which is unlikely to happen by chance. For example, for each 2-gene family, one can assume a simple binomial model and say that if the family displays a skew in N cancers, the probability of the same gene having the highest ML in all N cancers by random chance will be $(1/2)^N$; for 3-gene families it will be $(1/3)^N$ and for 4-gene families $(1/4)^N$. These values decrease quickly as N increases. In this table, 96% of gene families with a skewed ML in 4 or more cancers present the most extreme case, that is a single gene carries the heaviest ML in all of the relevant cancers. The probability of

obtaining even more extreme results is 0 in all of these instances. Also, the probability of these observed results occurring by chance is lower than 0.01 in 57% of such families, and lower than 0.001 in 21% of such families.

(b) For the ML-skewed families of more than two members, that the gene ranked most-mutated *and* that ranked second-most mutated should be the same in multiple cancers (as occurs often, see Table 1) is even more unlikely by chance, for similar reasons to those outlined in (a) above.

**Table S4 - Medulloblastoma samples in the Alexandrov dataset are annotated according to their subtype (where known) and according to whether they carry mutations in p53 or p63.** The table lists the medullobalstoma subtype (SubType, column C) and mutated gene (GeneMutated, column B) of the p53 family for the medulloblastoma samples (TumorID, column A) analysed in Alexandrov et al (2013). Medulloblastoma subtypes are characterized by WNT signaling (WNT), SHH signaling (SHH), as Group 3 and as Group 4, or as not known (N/A).

**Table S5 - Mutation scores and mutation loads (MLs) for those 2R-ohnologue families that display ML skews in melanoma and colorectal cancers with B-Raf$^{V600E}$ or N-Ras$^{Q61K/R}$ mutations.** Column A indicates the cancer types, which are melanoma and colorectal cancers with N-Ras$^{Q61K/R}$ (upper set of rows) and melanoma and colorectal cancers with B-Raf$^{V600E}$ (lower set of rows) selected from the data in Alexandrov et al (2013). Column B gives the identifier number for the 2R-ohnologue protein family. Column C contains information on the members of each family (geneID|UniProtID|UniProtName|number of mutations in each family member|skew score for the family) for the cancer type and for the Ras or Raf mutation named in column A. Column D indicates whether the 2R-ohnologue family displays a ML skew in the samples with N-Ras$^{Q61K/R}$ and/or with B-Raf$^{V600E}$ in one cancer type (NofFamilyInstance =1), or in both melanoma and colorectal cancer (NofFamilyInstance = 2).

**Table S6 - 2R-ohnologues whose mRNAs are included in the E-GEOD-3189 melanoma transcription profiling dataset and whose proteins were captured by 14-3-3 in melanoma cell lysates.** The table gives UniProt protein identifiers (column A), Ensembl gene identifiers (column B), and 2R-ohnologue family identifiers (column C). Column D is a binary tag: YES if the protein is in the list of 14-3-3-affinity captured proteins from SKMEL13 (B-Raf$^{V600E}$) and SBCL2 (N-Ras$^{Q61K}$) melanoma cell lysates, and NO if not (see main text). The GeneScore (column E) shows the ML score of the gene calculated from the Alexandrov et al (2013) data. Column F (ExpValue) shows the log2 ratio of mRNA expression in malignant melanoma versus benign melanocytic lesions in the E-GEOD-3189 dataset (www.ebi.ac.uk/arrayexpress/experiments/E-GEOD-3189) (Talantov et al 2005).

**Table S7 - 2R-ohnologues whose mRNAs are included in the E-GEOD-32867 lung adenocarcinoma profiling dataset.** The table gives UniProt protein identifiers (column A), Ensembl gene identifiers (column B). The GeneScore (column C) shows the ML score of the gene calculated from the Alexandrov et al (2013) data. Column D (ExpValue) shows the log2

ratio of mRNA expression in lung adenocarcinoma versus adjacent non-tumor lung tissue (ExpValue) in the E-GEOD-32867 dataset (www.ebi.ac.uk/arrayexpress/experiments/E-GEOD-32867). Column E is the identifier for the 2R-ohnologue protein family.

**Table S8 - Proteins captured by 14-3-3-affinity chromatography from lysates of SBCL2 (N-Ras$^{Q61K}$) melanoma cells.** The table reports the MASCOT score (ProteinScore), the protein rank (ProteinRank) the UniProt id (ProteinId) and the UniProt description (ProteinDescription) for the proteins identified in each gel band (GelBand SbCl2) for the 14-3-3 affinity capture in SBCL2 (N-Ras$^{Q61K}$) melanoma cell lysates (see Supplementary Fig S6).

**Table S9 - Proteins captured by 14-3-3-affinity chromatography from lysates of SKMEL13 (B-Raf$^{V600E}$) melanoma cells.** The table reports the MASCOT score (ProteinScore), the protein rank (ProteinRank) the UniProt id (ProteinId) and the UniProt description (ProteinDescription) for the proteins identified in each gel band (GelBand SKM13) for the 14-3-3 affinity capture in SKMEL13 (B-Raf$^{V600E}$) melanoma cell lysates (see Supplementary Fig S6).

**Table S10 - 14-3-3-affinity captured proteins analysed in ANIA.** This table includes information downloaded from the ANIA database https://ania-1433.lifesci.dundee.ac.uk/prediction/webserver/index.py (Tinti et al, Database (Oxford) 2014 in press) for the 235 proteins that were 14-3-3-affinity captured from melanoma cells in the present study, and which were either well-characterized 14-3-3-binding phosphoproteins (Golden Standards) or which passed stringent filters as candidate 14-3-3-binding phosphoproteins. The columns of the table report (A) UniProt Ids for human protein (UniProt), (B) UniProt name (Name), (C) UniProt description (Description), (D) under 'Gold standard' is given the residue number(s) for identified 14-3-3-binding phosphosites, (E) PubMed PMID number for article characterising the protein as a 14-3-3-binding phosphoprotein, (F and G) UniProt Ids for corresponding mouse and rat proteins, (H) number of high-throughput 14-3-3-affinity capture experiments that have previously isolated the protein, (I) PubMed PMID number for articles on the high-throughput experiments, (J) number of times the protein has been identified as a 'contaminant' isolated in in-house control experiments using Sepharose or agarose beads with no 14-3-3 protein attached, (K) number of 2R-ohnologues in the family (N-Ohnologs), (L) members of the 2R-ohnologue family given by UniProt Id, (M) phosphorylated sites that conform to the sequences of candidate 14-3-3 binding sites, (N) GO gives the Gene Ontology classification, (O) 'GAD' indicates proteins with entries in the human Genetic Association Database (GAD) (60), (P) gives number of mutations listed in the COSMIC database, (Q) family ID (an arbitrary number assigned to identify each 2R-ohnologue family as in Tinti et al (2012).

## Legends for supplementary data files

### Data file S1 - Mutation loads of 2R-ohnologue genes in different cancers.

Each file contains data for a different cancer. Similar to Fig 2A for melanoma, the histograms plot the Mutation Load (ML) distribution for 2R-ohnologues within families of 2, 3 and 4 members. The ML is computed by summing the total number of mutations identified for a gene divided by the total number of mutations in all members of the same 2R-ohnologue family. Only families in which at least one gene carried 10 or more different mutations are included. Each histogram set indicates the medians (red lines), interquartile ranges (rectangular boxes) and outliers (green diamonds) for the ML distributions. Note that for families of 2 members, the median will always be 0.5 by construction, regardless of the ML distribution profile.

**Statistical note:** The numbers of 2R-ohnologue families with one gene carrying 10 or more different mutations differs in different cancers, and so too do the numbers of families with ML skews, making it difficult to perform a systematic statistical analysis to assess the role of randomness. Nevertheless, we can make a more qualitative statement, as follows: Under a purely random model, the ML distributions of 2R ohnologues in families of 2 members is expected to be approximated by a normal distribution centered on 0.5. Therefore, in such cases, a possible approach to define any deviation from randomness of the ML distribution is to measure the normality of the sample distribution (for example, using the Kolmogorov-Smirnov test). For families of 3 members, random distribution will have an expected value of 0.33, and for families of 4 it will be 0.25. Even without formal testing, the histograms here provide visual information that allows the degrees of normality of each distribution to be gauged. For example, the ML distribution for 2R ohnologues in 2-gene families in colorectal cancer is unimodal and short-tailed, and thus does not provide enough statistical support to exclude randomness as a possible generating mechanism. In contrast, the ML distribution for 2R ohnologues in pancreatic cancer appears multimodal and not bell-shaped for the 2-member families, advocating against a purely random process and suggesting selection in favour of extreme MLs. In future, further data will resolve these statistical issues.

### Data file S2 - Distributions of mutations within 2R-ohnologue genes in melanoma.

Each image shows the distribution of mutations in the melanoma datasets from Alexandrov et al (2013) in genes from a 2R-ohnologue family. Genes are identified by their Ensembl identifiers. Mutations are indicated by vertical black lines, and turquoise regions are 3’ and 5’ UTR regions.
