# Supplementary figures and images for: Identification of 2R-ohnologue gene families displaying the same mutation-load skew in multiple cancers (part 1 of 3)
Source: Open Biol. 2014 May 7;4(5):140029. doi: 10.1098/rsob.140029 (PMC4042849; doi:10.1098/rsob.140029)

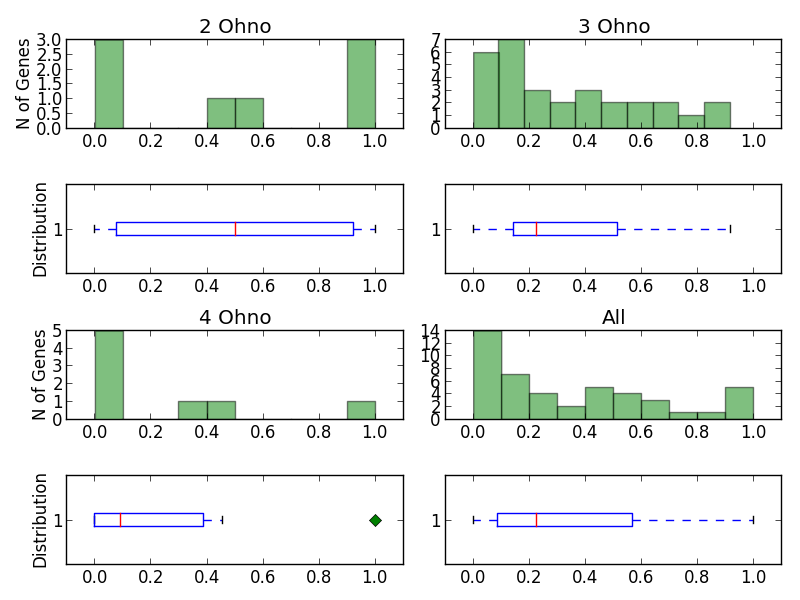

Supplement: Data file S1 [file rsob140029supp2.zip › rsob-14-0029-File009/data file S1/ALL.png]

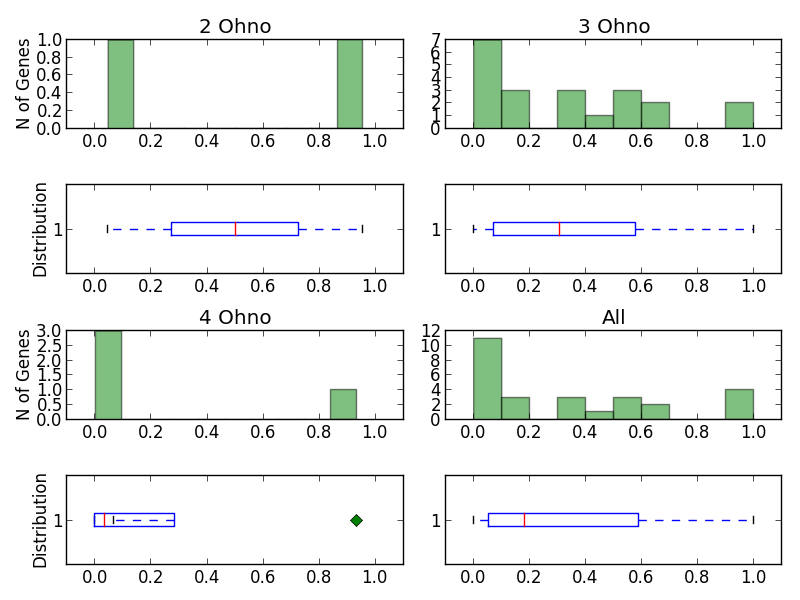

Supplement: Data file S1 [file rsob140029supp2.zip › rsob-14-0029-File009/data file S1/AML.png]

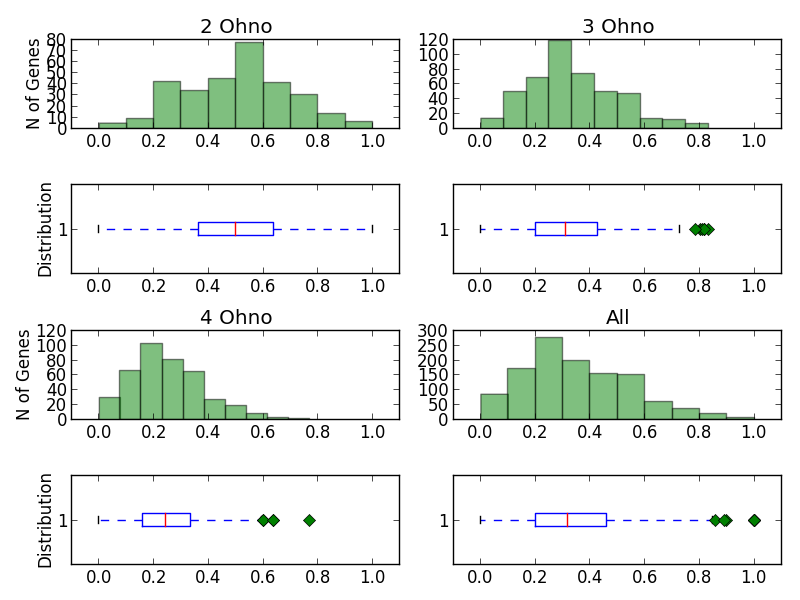

Supplement: Data file S1 [file rsob140029supp2.zip › rsob-14-0029-File009/data file S1/Bladder.png]

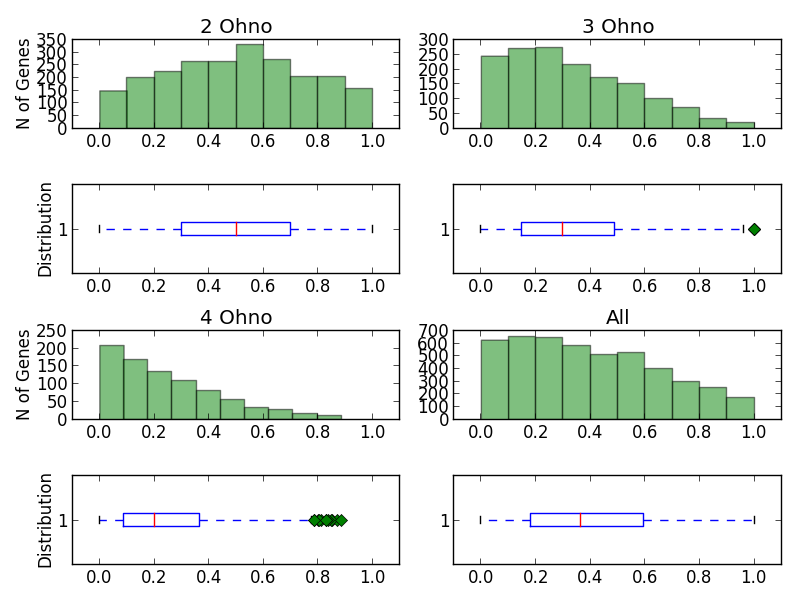

Supplement: Data file S1 [file rsob140029supp2.zip › rsob-14-0029-File009/data file S1/Breast.png]

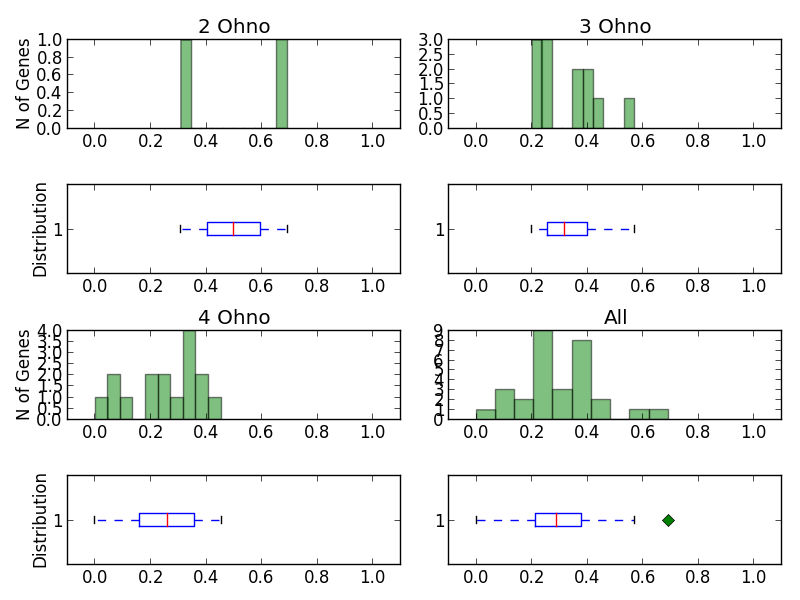

Supplement: Data file S1 [file rsob140029supp2.zip › rsob-14-0029-File009/data file S1/Cervix.png]

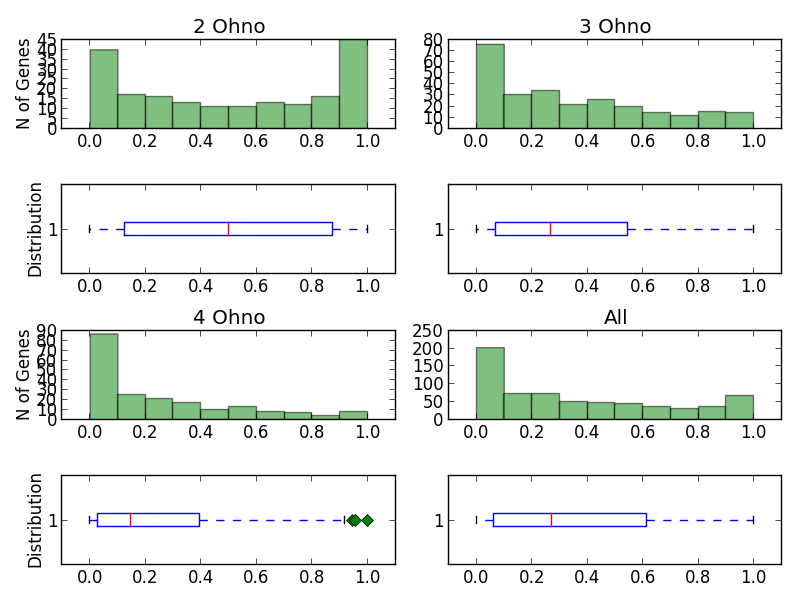

Supplement: Data file S1 [file rsob140029supp2.zip › rsob-14-0029-File009/data file S1/CLL.png]

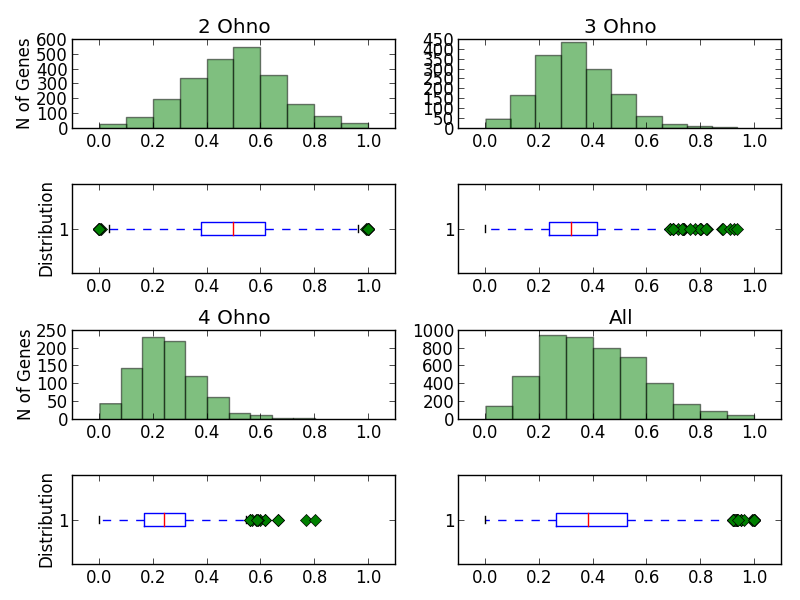

Supplement: Data file S1 [file rsob140029supp2.zip › rsob-14-0029-File009/data file S1/Colorectum.png]

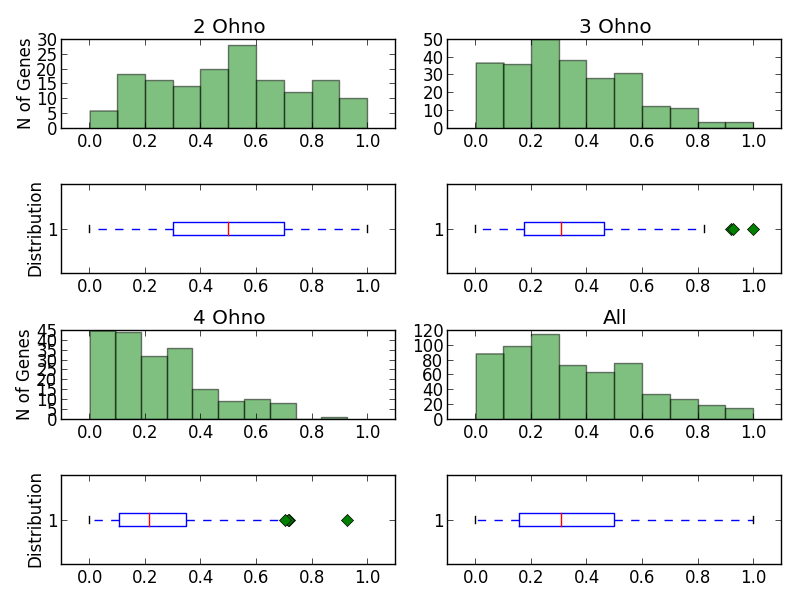

Supplement: Data file S1 [file rsob140029supp2.zip › rsob-14-0029-File009/data file S1/Esophageal.png]

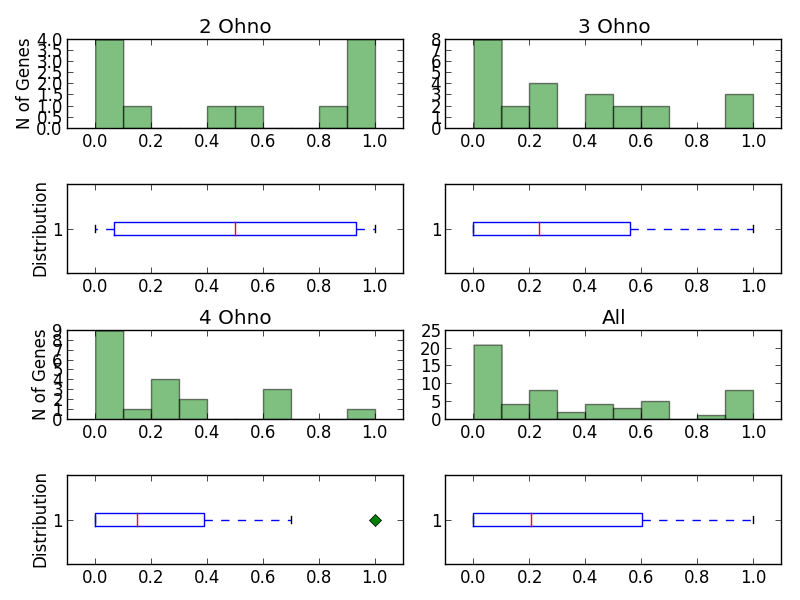

Supplement: Data file S1 [file rsob140029supp2.zip › rsob-14-0029-File009/data file S1/Glioblastoma.png]

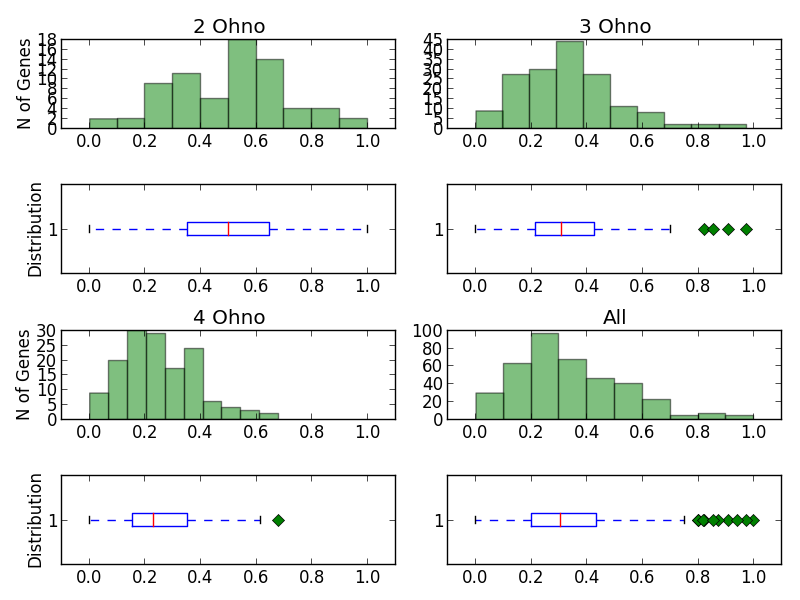

Supplement: Data file S1 [file rsob140029supp2.zip › rsob-14-0029-File009/data file S1/Glioma Low Grade.png]

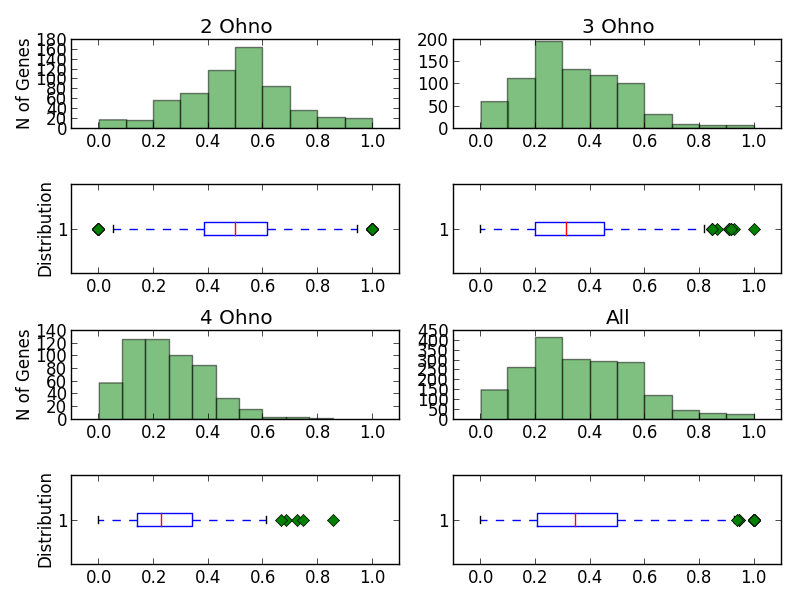

Supplement: Data file S1 [file rsob140029supp2.zip › rsob-14-0029-File009/data file S1/Head and Neck.png]

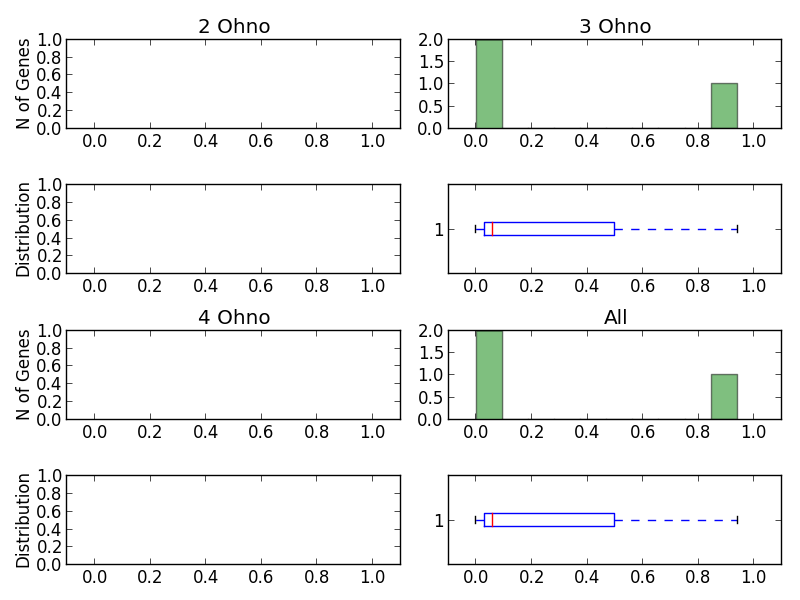

Supplement: Data file S1 [file rsob140029supp2.zip › rsob-14-0029-File009/data file S1/Kidney Chromophobe.png]

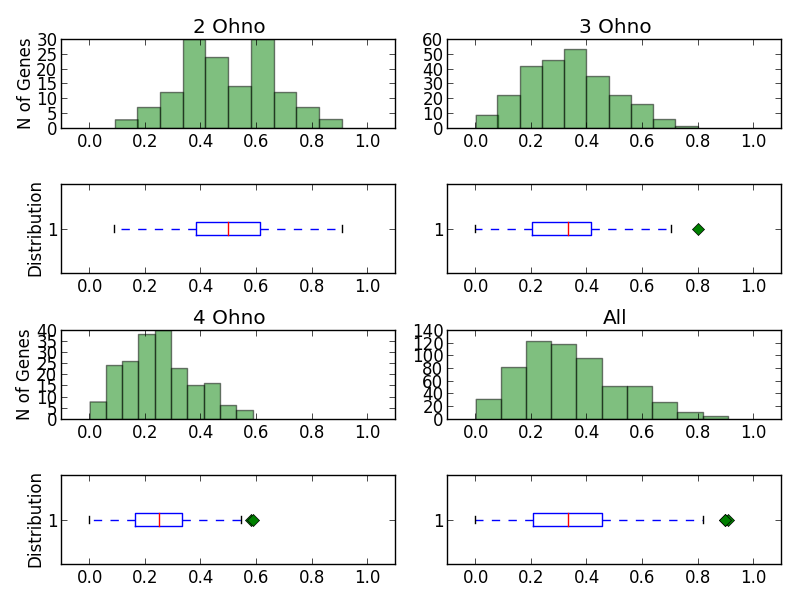

Supplement: Data file S1 [file rsob140029supp2.zip › rsob-14-0029-File009/data file S1/Kidney Clear Cell.png]

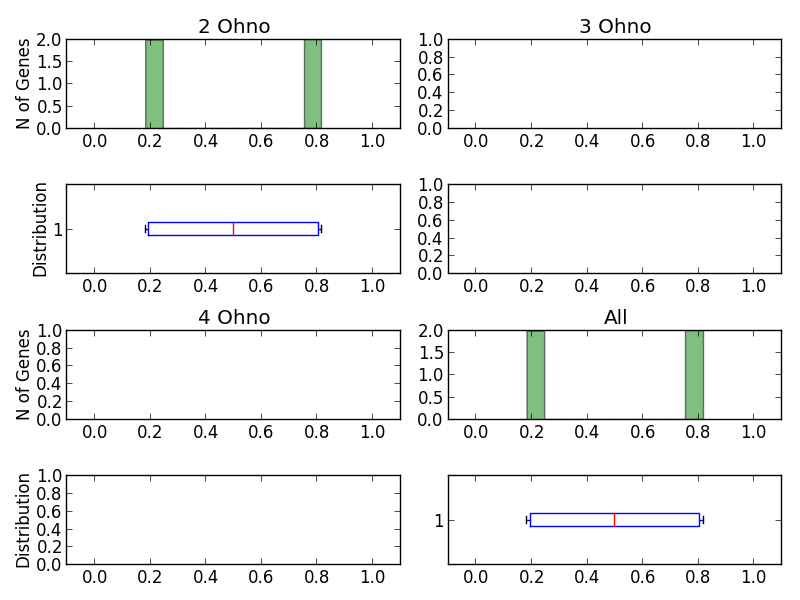

Supplement: Data file S1 [file rsob140029supp2.zip › rsob-14-0029-File009/data file S1/Kidney Papillary.png]

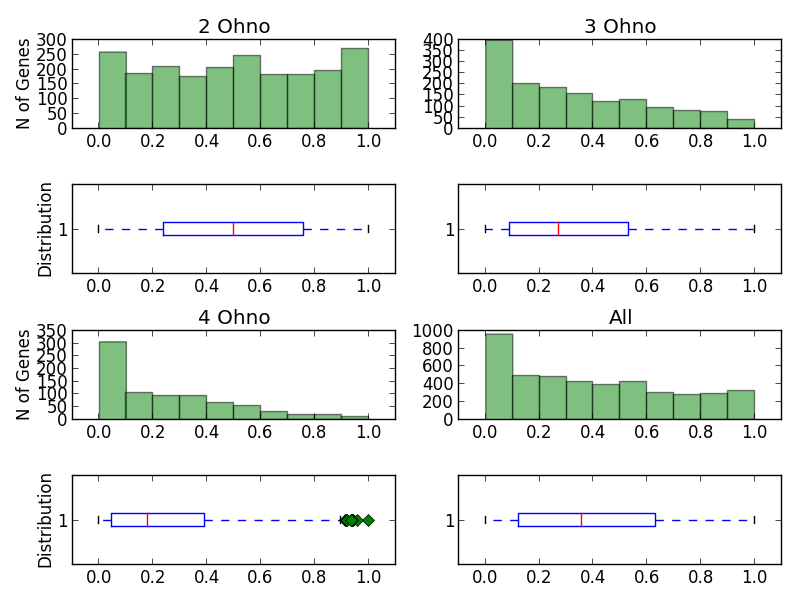

Supplement: Data file S1 [file rsob140029supp2.zip › rsob-14-0029-File009/data file S1/Liver.png]

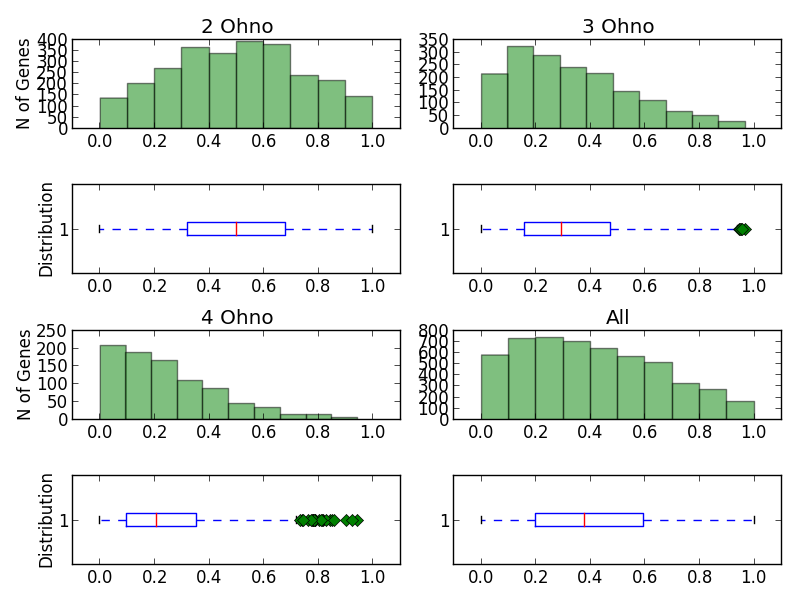

Supplement: Data file S1 [file rsob140029supp2.zip › rsob-14-0029-File009/data file S1/Lung Adeno.png]

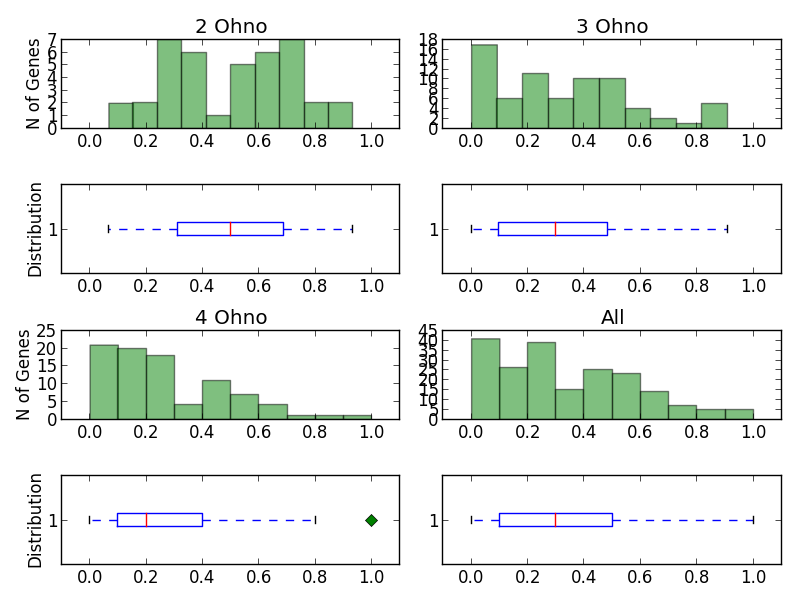

Supplement: Data file S1 [file rsob140029supp2.zip › rsob-14-0029-File009/data file S1/Lung Small Cell.png]

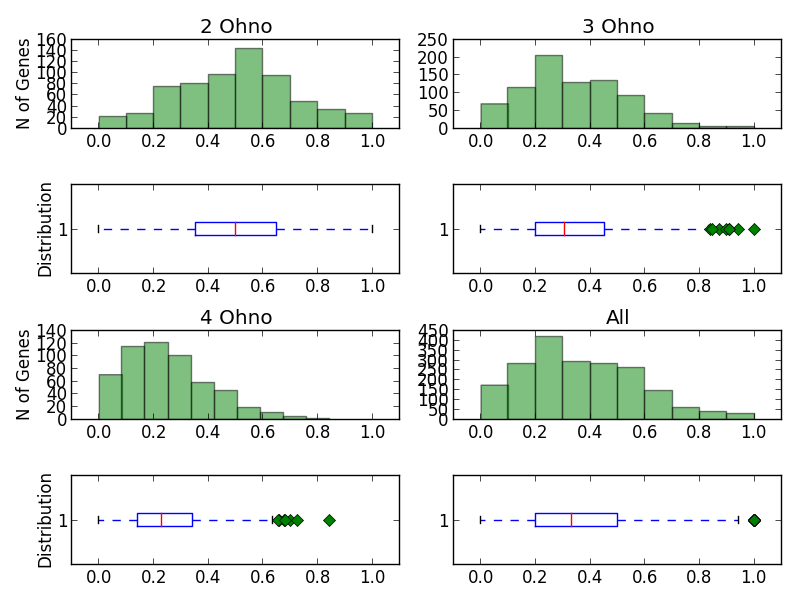

Supplement: Data file S1 [file rsob140029supp2.zip › rsob-14-0029-File009/data file S1/Lung Squamous.png]

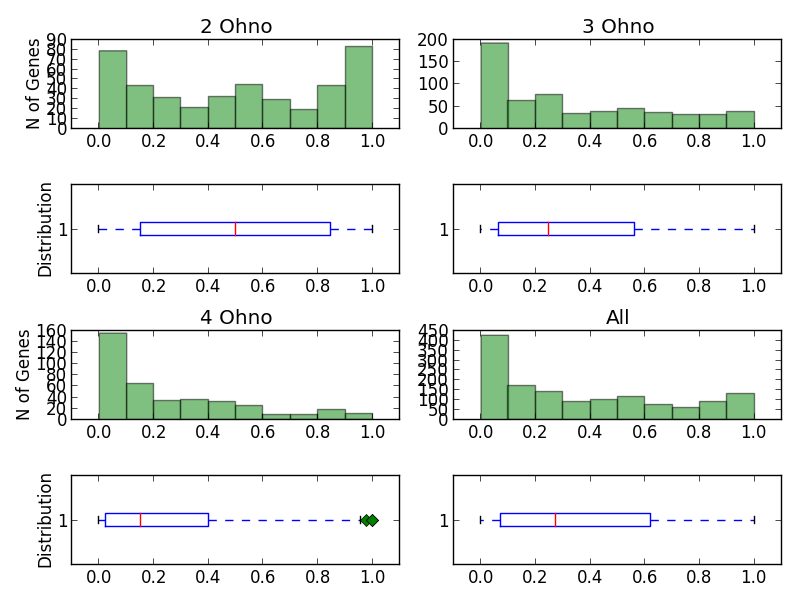

Supplement: Data file S1 [file rsob140029supp2.zip › rsob-14-0029-File009/data file S1/Lymphoma B-cell.png]

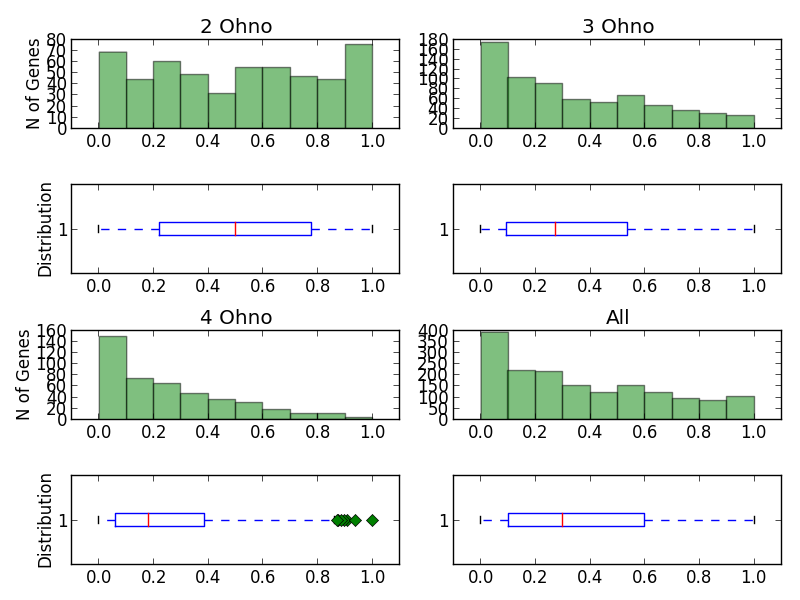

Supplement: Data file S1 [file rsob140029supp2.zip › rsob-14-0029-File009/data file S1/Medulloblastoma.png]

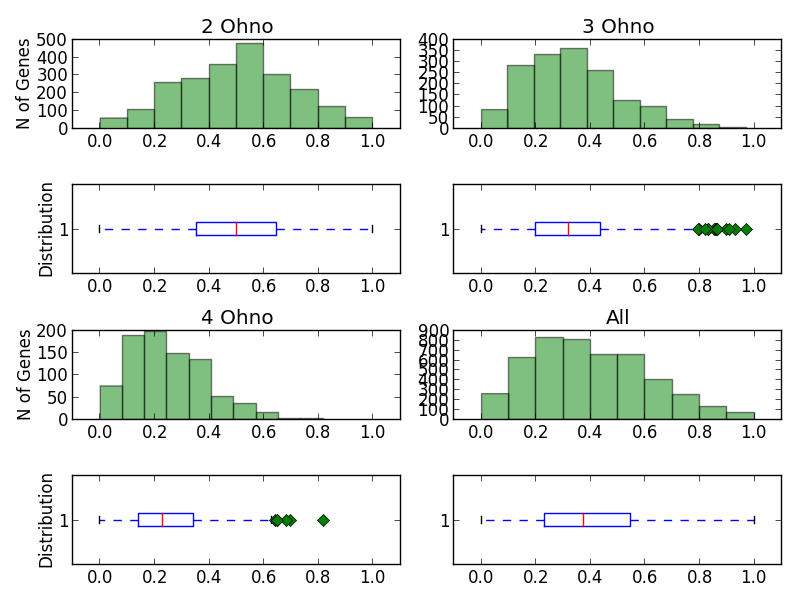

Supplement: Data file S1 [file rsob140029supp2.zip › rsob-14-0029-File009/data file S1/Melanoma.png]

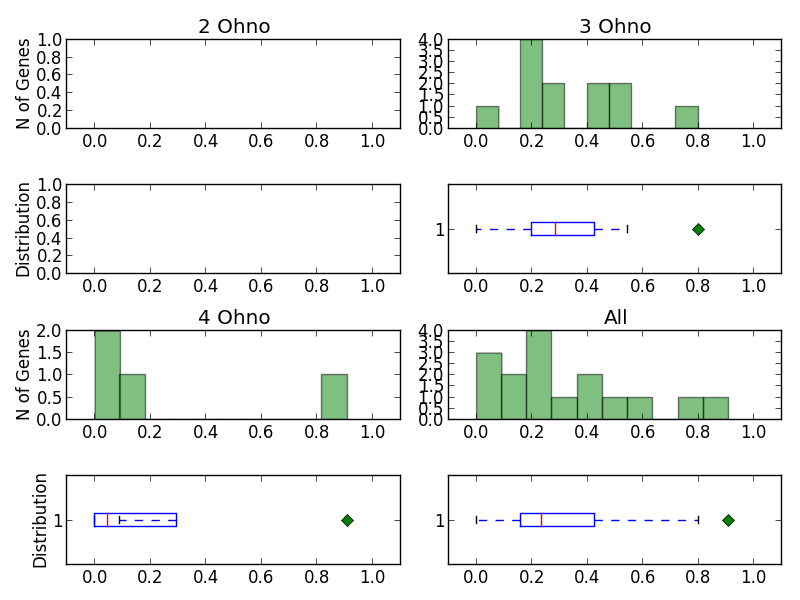

Supplement: Data file S1 [file rsob140029supp2.zip › rsob-14-0029-File009/data file S1/Myeloma.png]

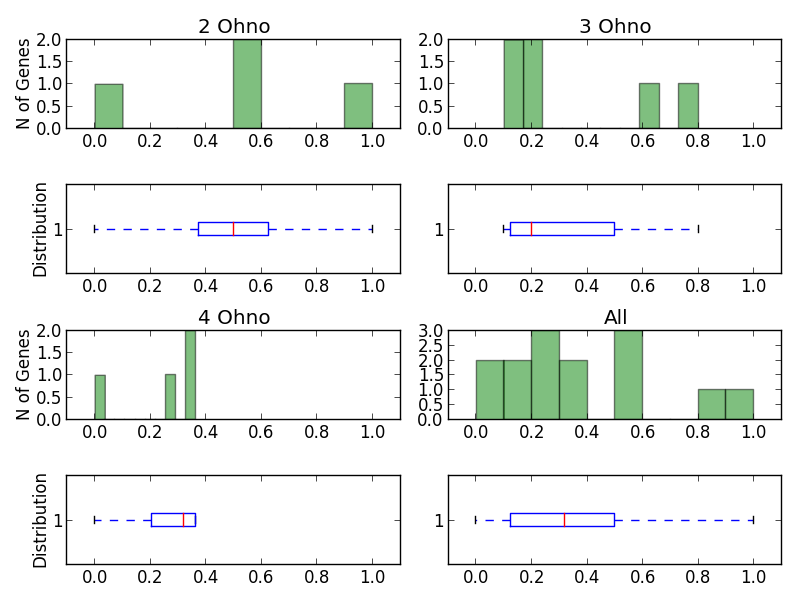

Supplement: Data file S1 [file rsob140029supp2.zip › rsob-14-0029-File009/data file S1/Neuroblastoma.png]

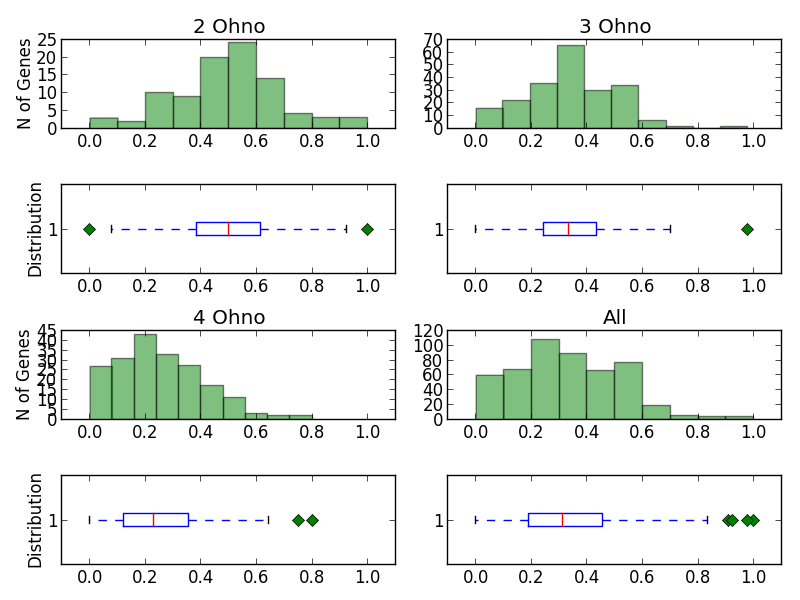

Supplement: Data file S1 [file rsob140029supp2.zip › rsob-14-0029-File009/data file S1/Ovary.png]

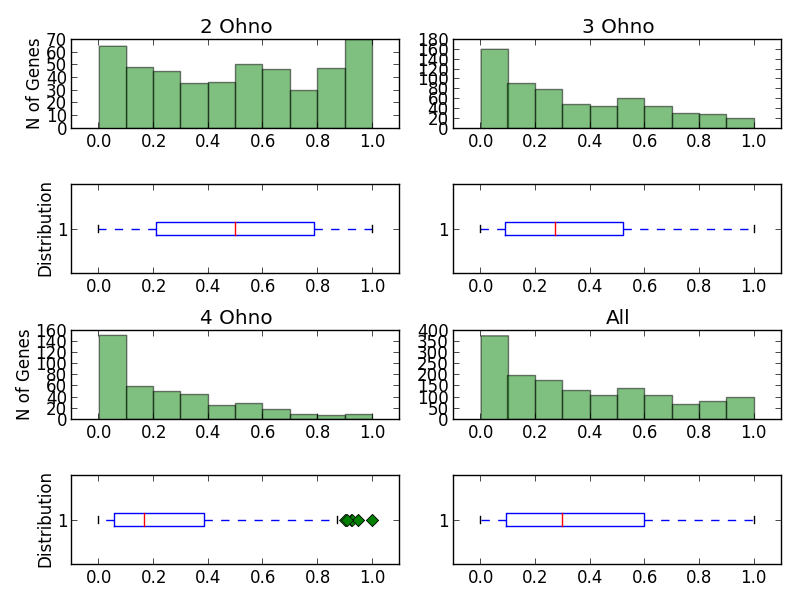

Supplement: Data file S1 [file rsob140029supp2.zip › rsob-14-0029-File009/data file S1/Pancreas.png]

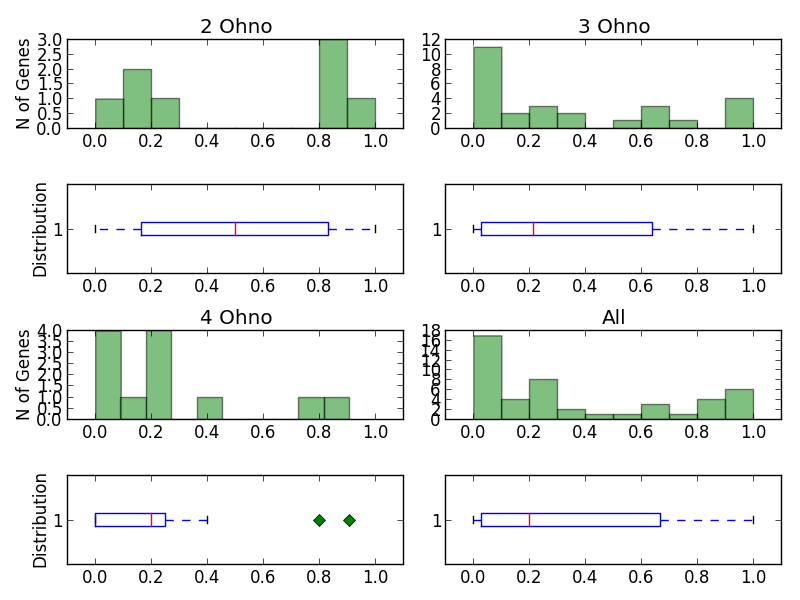

Supplement: Data file S1 [file rsob140029supp2.zip › rsob-14-0029-File009/data file S1/Pilocytic Astrocytoma.png]

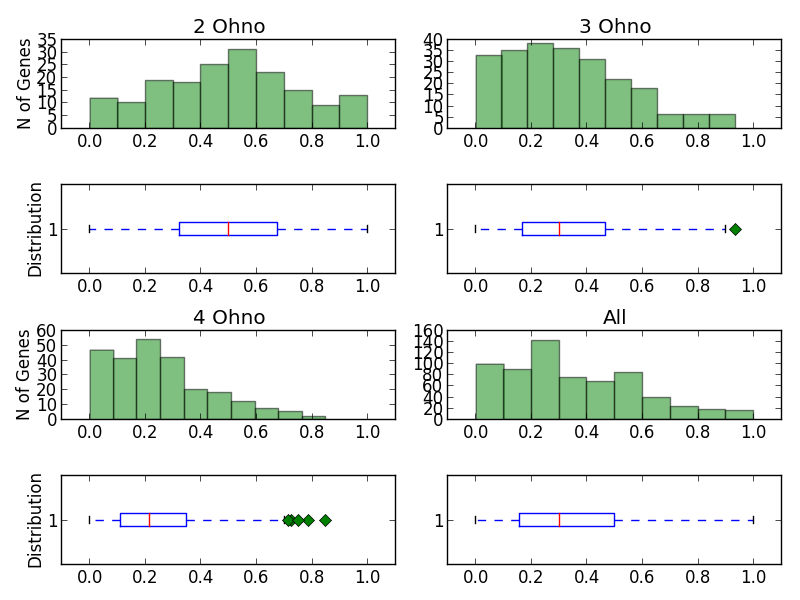

Supplement: Data file S1 [file rsob140029supp2.zip › rsob-14-0029-File009/data file S1/Prostate.png]

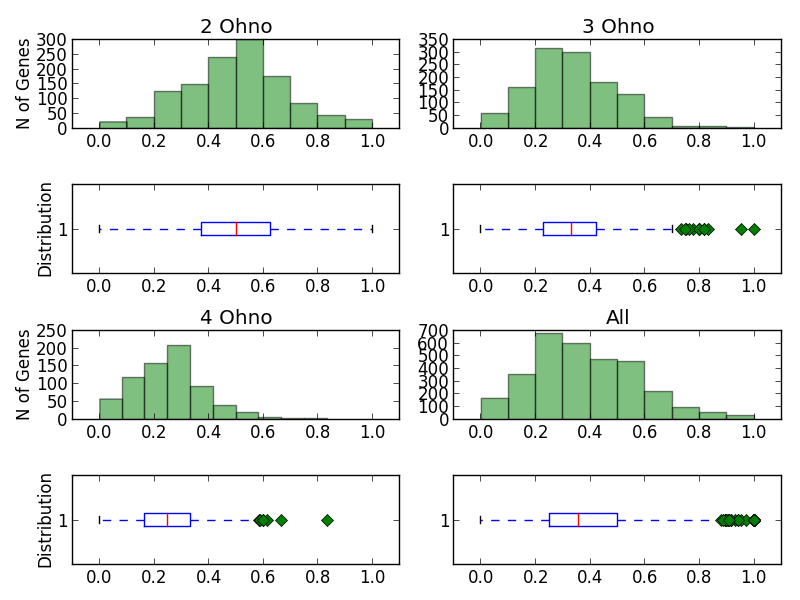

Supplement: Data file S1 [file rsob140029supp2.zip › rsob-14-0029-File009/data file S1/Stomach.png]

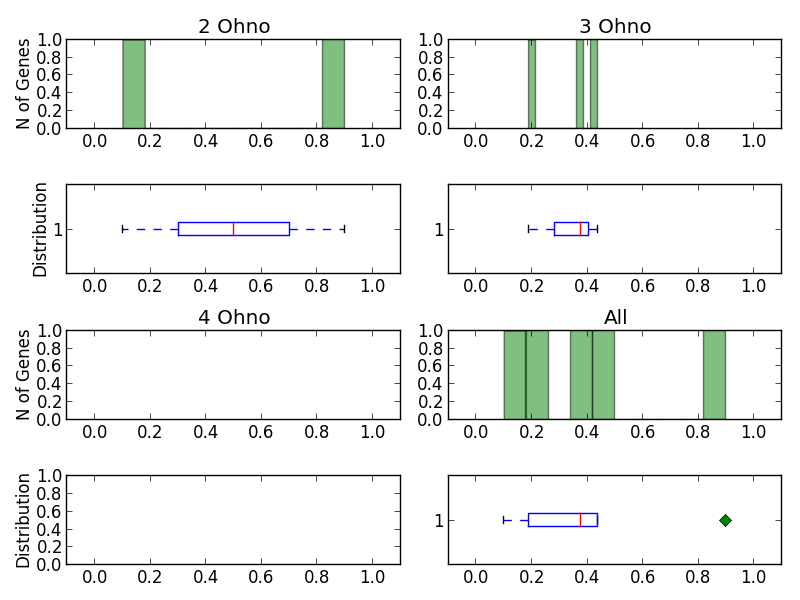

Supplement: Data file S1 [file rsob140029supp2.zip › rsob-14-0029-File009/data file S1/Thyroid.png]

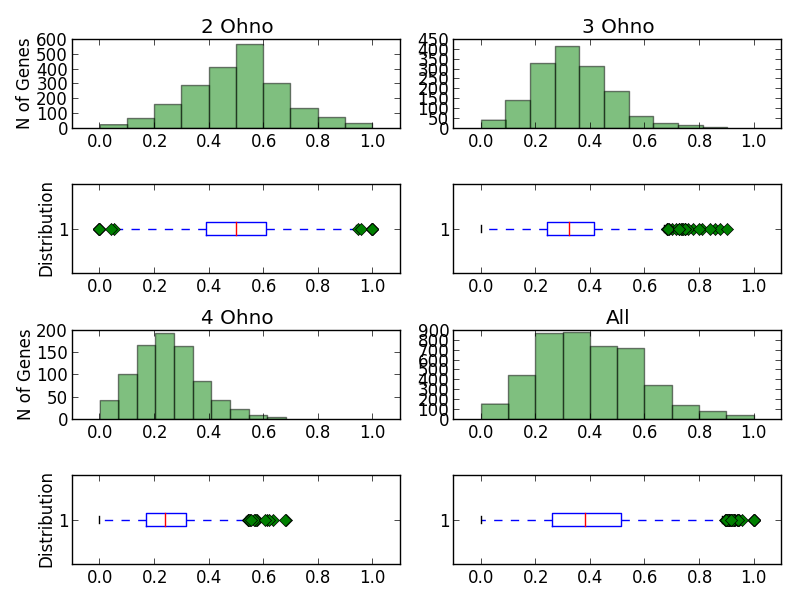

Supplement: Data file S1 [file rsob140029supp2.zip › rsob-14-0029-File009/data file S1/Uterus.png]

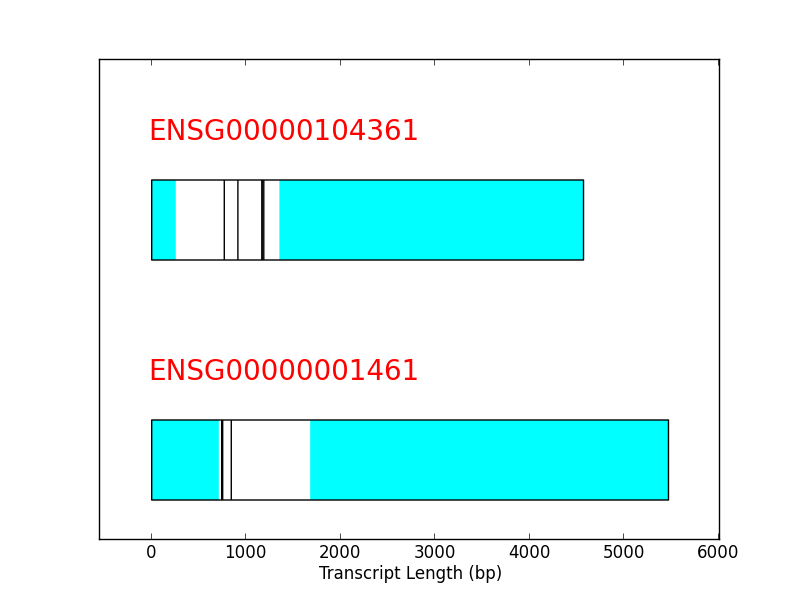

Supplement: Data file S2 [file rsob140029supp3.zip › rsob-14-0029-File010/Melanoma/ENSG00000001461_ENSG00000104361.png]

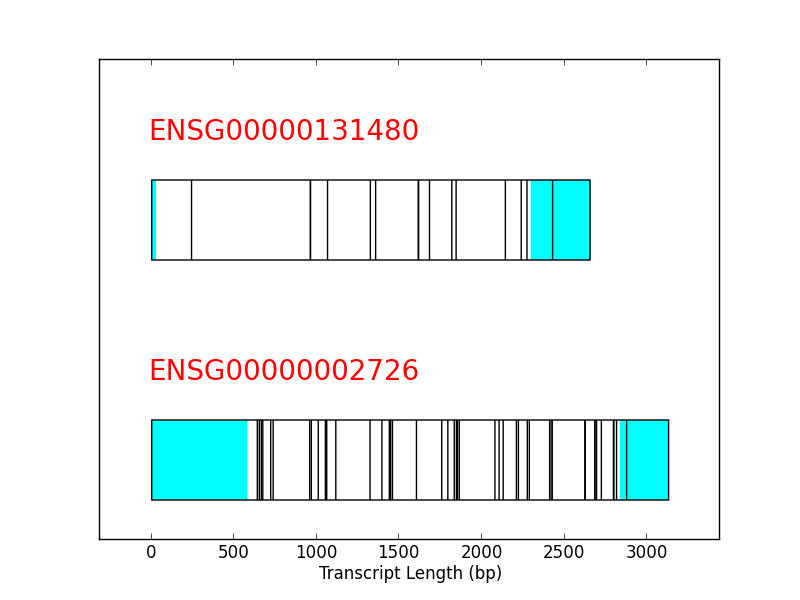

Supplement: Data file S2 [file rsob140029supp3.zip › rsob-14-0029-File010/Melanoma/ENSG00000002726_ENSG00000131480.png]

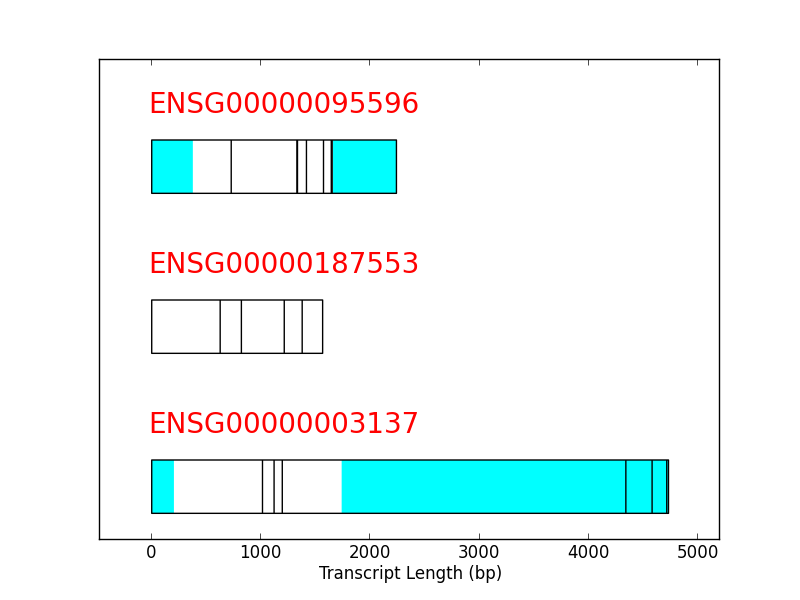

Supplement: Data file S2 [file rsob140029supp3.zip › rsob-14-0029-File010/Melanoma/ENSG00000003137_ENSG00000187553_ENSG00000095596.png]

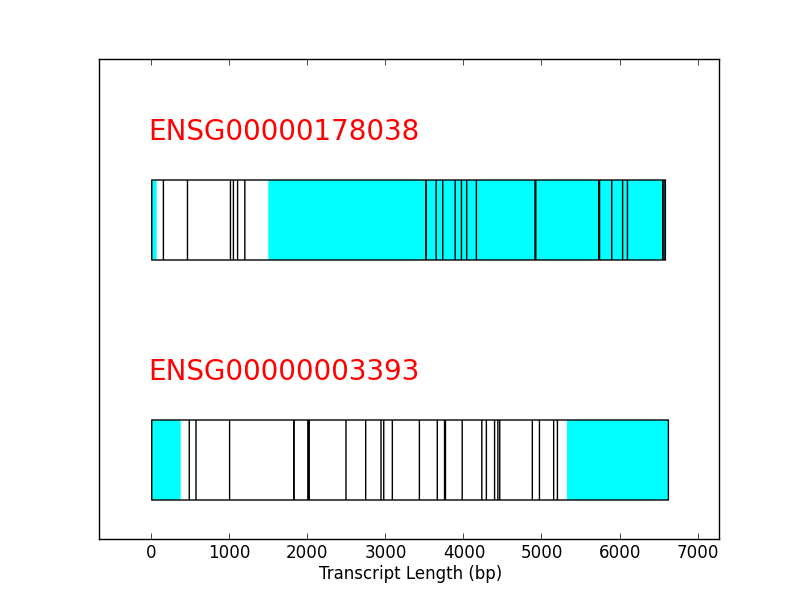

Supplement: Data file S2 [file rsob140029supp3.zip › rsob-14-0029-File010/Melanoma/ENSG00000003393_ENSG00000178038.png]

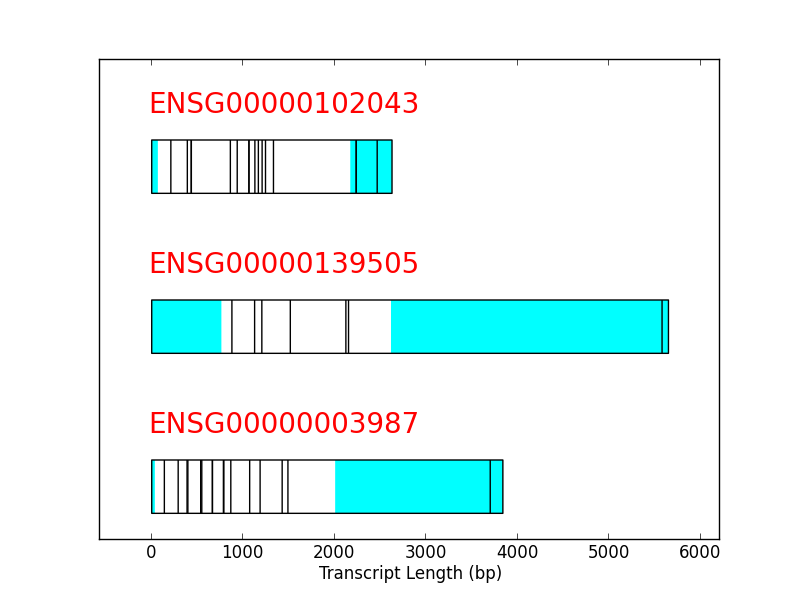

Supplement: Data file S2 [file rsob140029supp3.zip › rsob-14-0029-File010/Melanoma/ENSG00000003987_ENSG00000139505_ENSG00000102043.png]

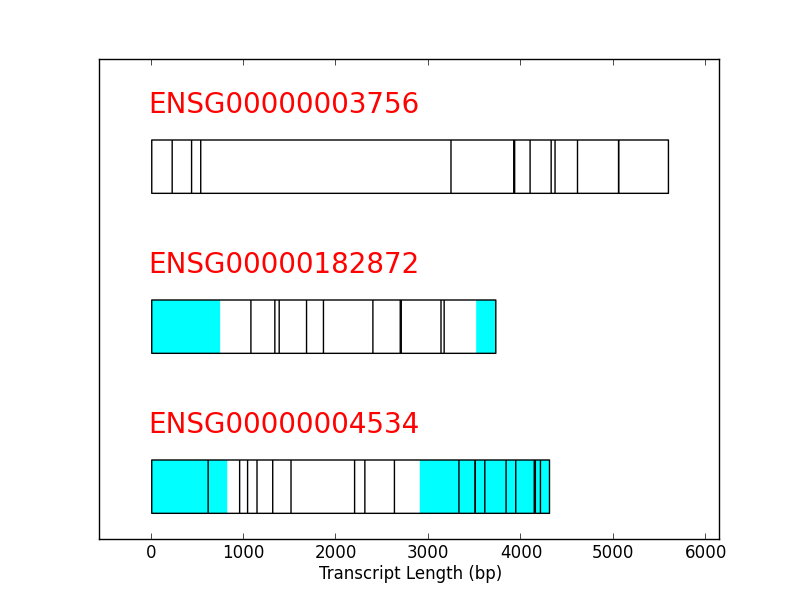

Supplement: Data file S2 [file rsob140029supp3.zip › rsob-14-0029-File010/Melanoma/ENSG00000004534_ENSG00000182872_ENSG00000003756.png]

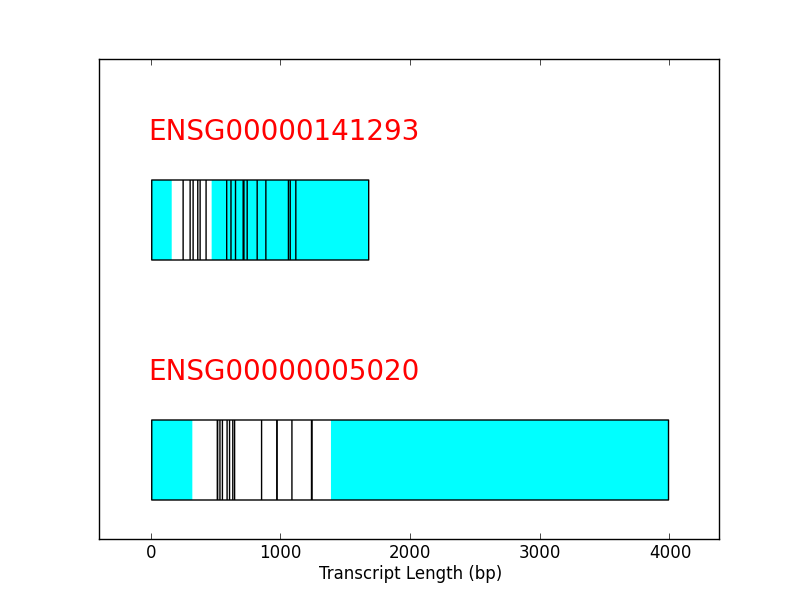

Supplement: Data file S2 [file rsob140029supp3.zip › rsob-14-0029-File010/Melanoma/ENSG00000005020_ENSG00000141293.png]

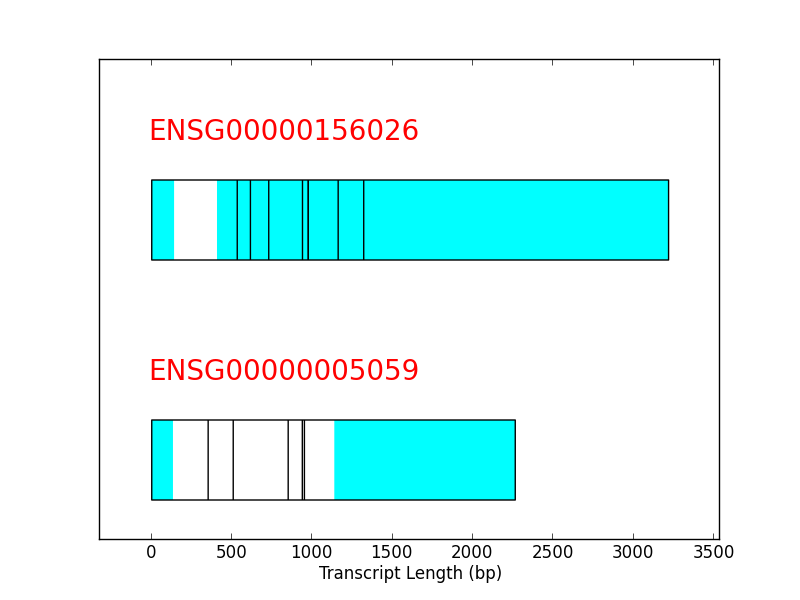

Supplement: Data file S2 [file rsob140029supp3.zip › rsob-14-0029-File010/Melanoma/ENSG00000005059_ENSG00000156026.png]

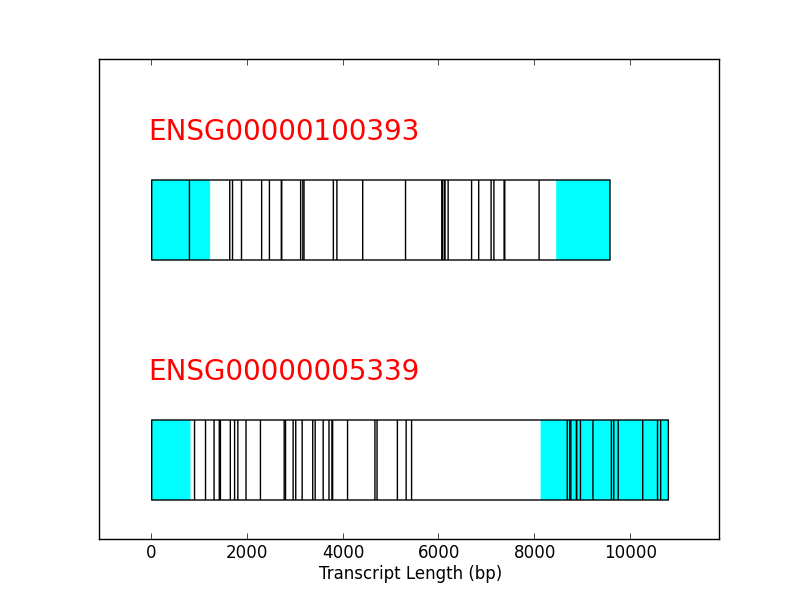

Supplement: Data file S2 [file rsob140029supp3.zip › rsob-14-0029-File010/Melanoma/ENSG00000005339_ENSG00000100393.png]

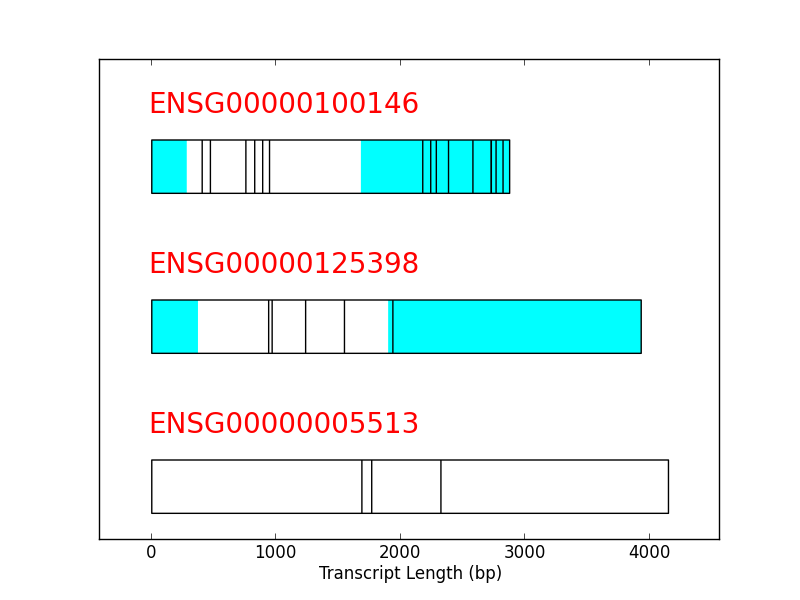

Supplement: Data file S2 [file rsob140029supp3.zip › rsob-14-0029-File010/Melanoma/ENSG00000005513_ENSG00000125398_ENSG00000100146.png]

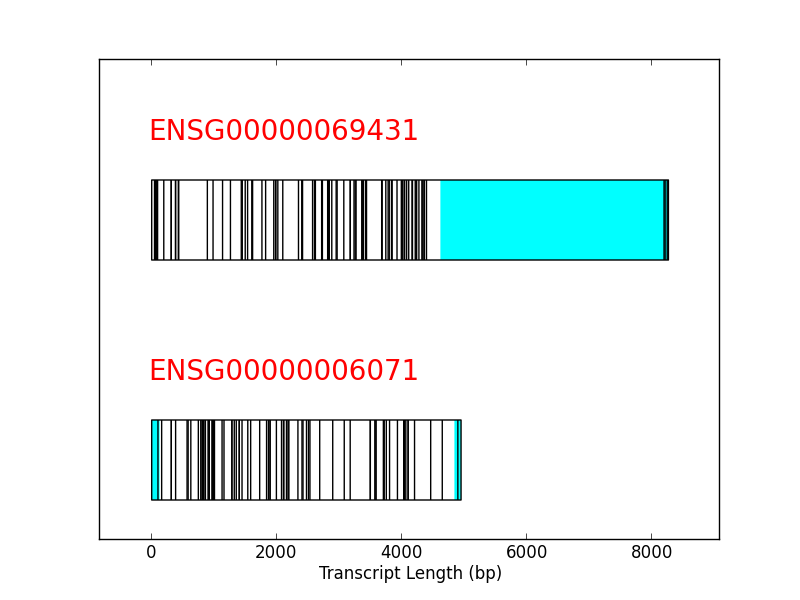

Supplement: Data file S2 [file rsob140029supp3.zip › rsob-14-0029-File010/Melanoma/ENSG00000006071_ENSG00000069431.png]

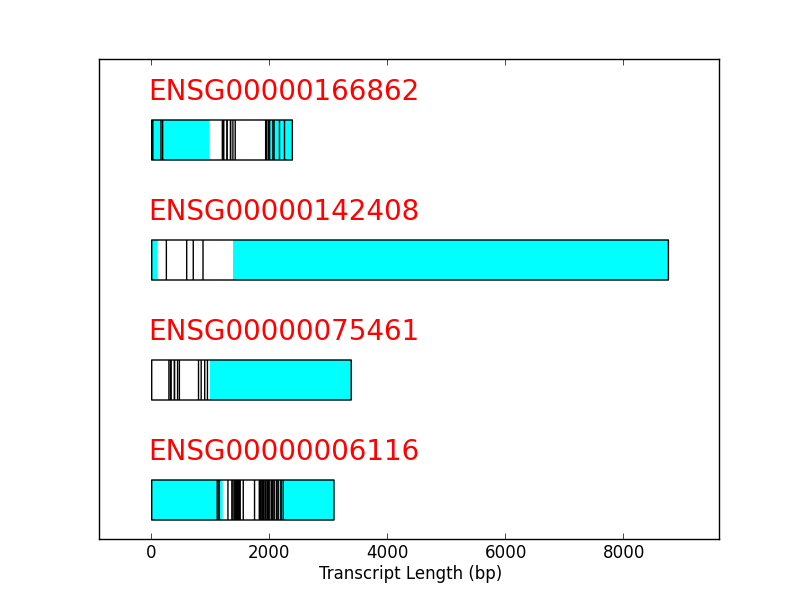

Supplement: Data file S2 [file rsob140029supp3.zip › rsob-14-0029-File010/Melanoma/ENSG00000006116_ENSG00000075461_ENSG00000142408_ENSG00000166862.png]

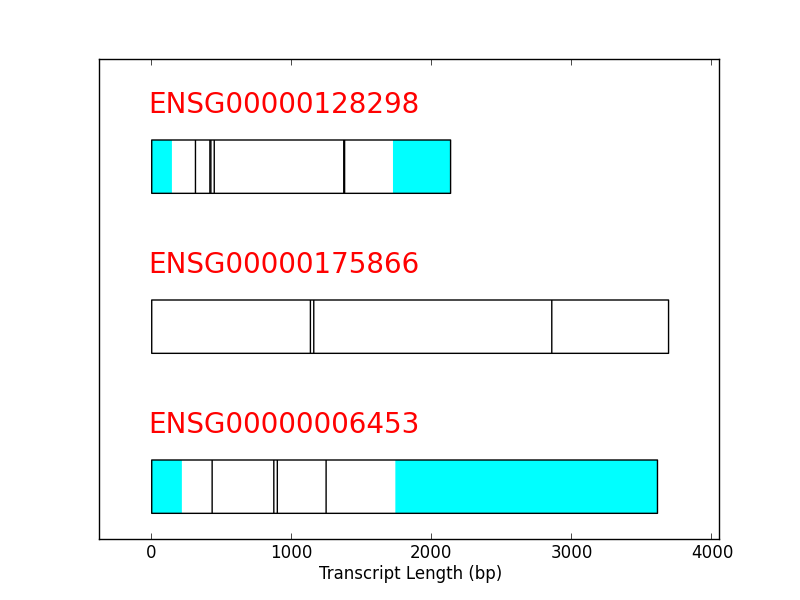

Supplement: Data file S2 [file rsob140029supp3.zip › rsob-14-0029-File010/Melanoma/ENSG00000006453_ENSG00000175866_ENSG00000128298.png]

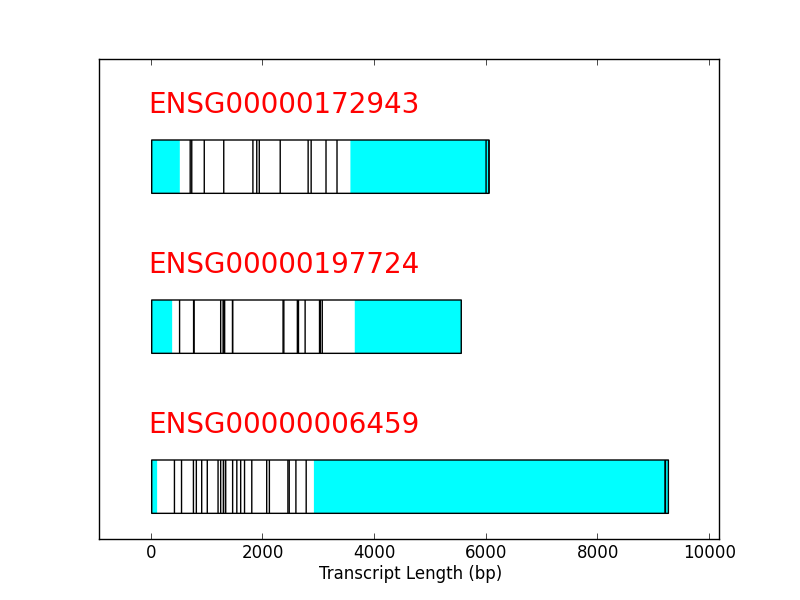

Supplement: Data file S2 [file rsob140029supp3.zip › rsob-14-0029-File010/Melanoma/ENSG00000006459_ENSG00000197724_ENSG00000172943.png]

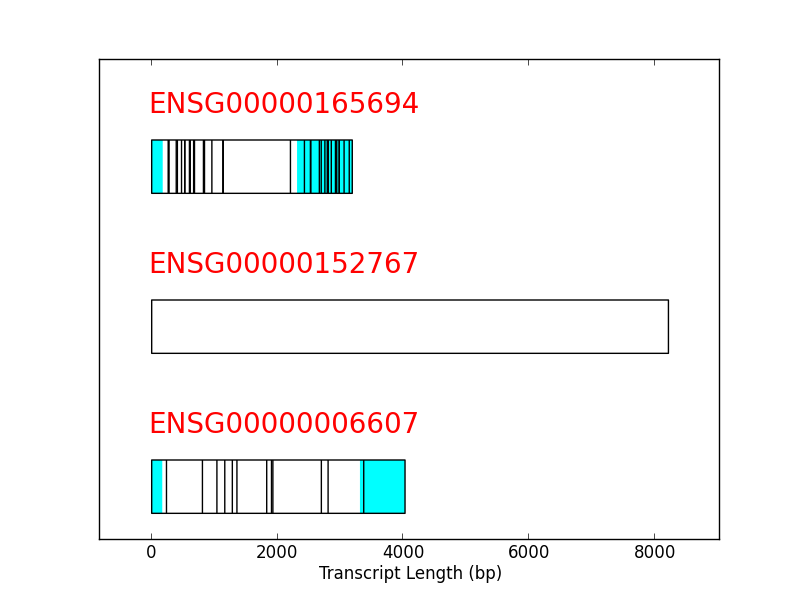

Supplement: Data file S2 [file rsob140029supp3.zip › rsob-14-0029-File010/Melanoma/ENSG00000006607_ENSG00000152767_ENSG00000165694.png]

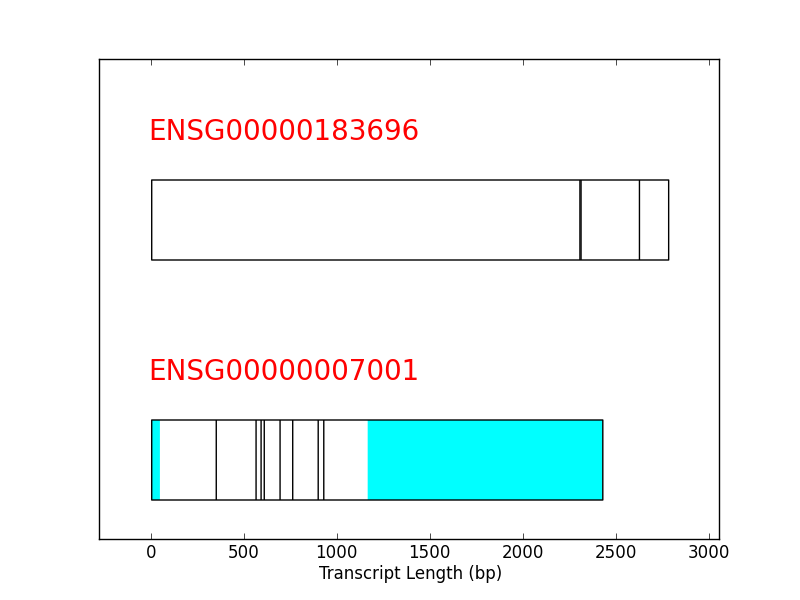

Supplement: Data file S2 [file rsob140029supp3.zip › rsob-14-0029-File010/Melanoma/ENSG00000007001_ENSG00000183696.png]

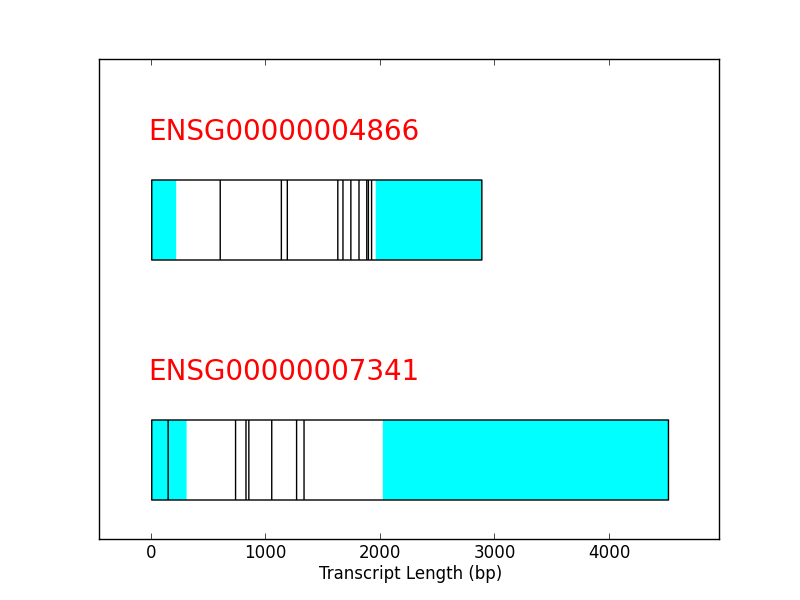

Supplement: Data file S2 [file rsob140029supp3.zip › rsob-14-0029-File010/Melanoma/ENSG00000007341_ENSG00000004866.png]

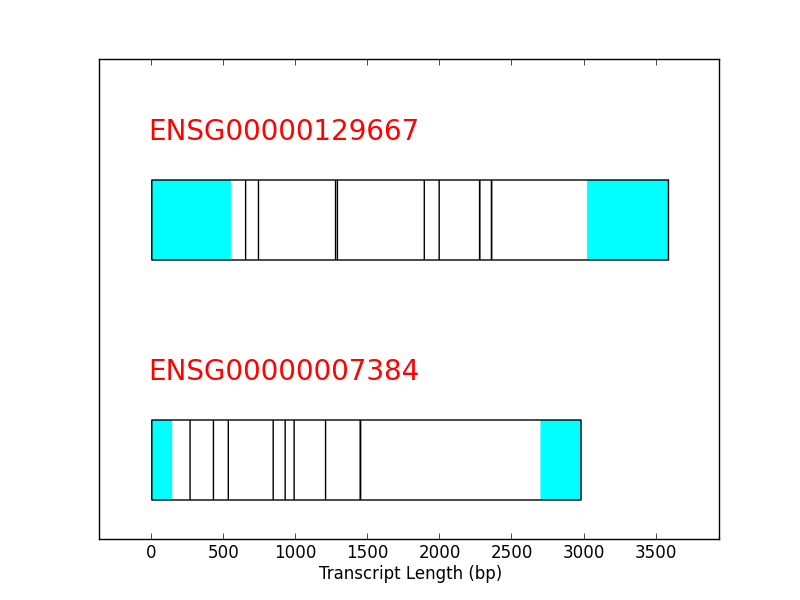

Supplement: Data file S2 [file rsob140029supp3.zip › rsob-14-0029-File010/Melanoma/ENSG00000007384_ENSG00000129667.png]

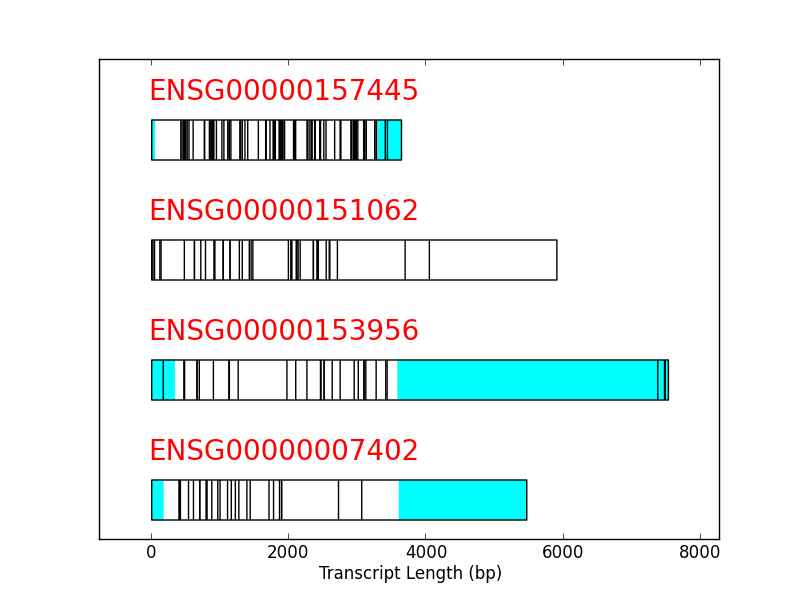

Supplement: Data file S2 [file rsob140029supp3.zip › rsob-14-0029-File010/Melanoma/ENSG00000007402_ENSG00000153956_ENSG00000151062_ENSG00000157445.png]

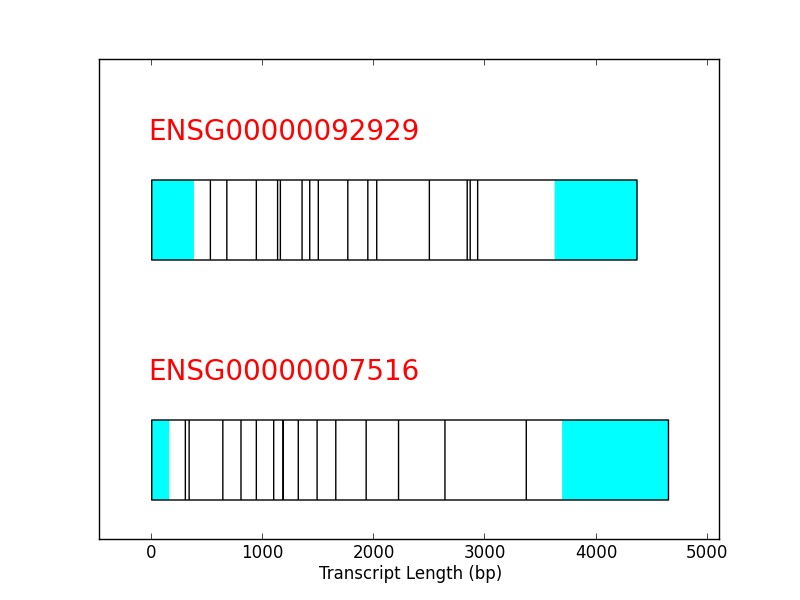

Supplement: Data file S2 [file rsob140029supp3.zip › rsob-14-0029-File010/Melanoma/ENSG00000007516_ENSG00000092929.png]

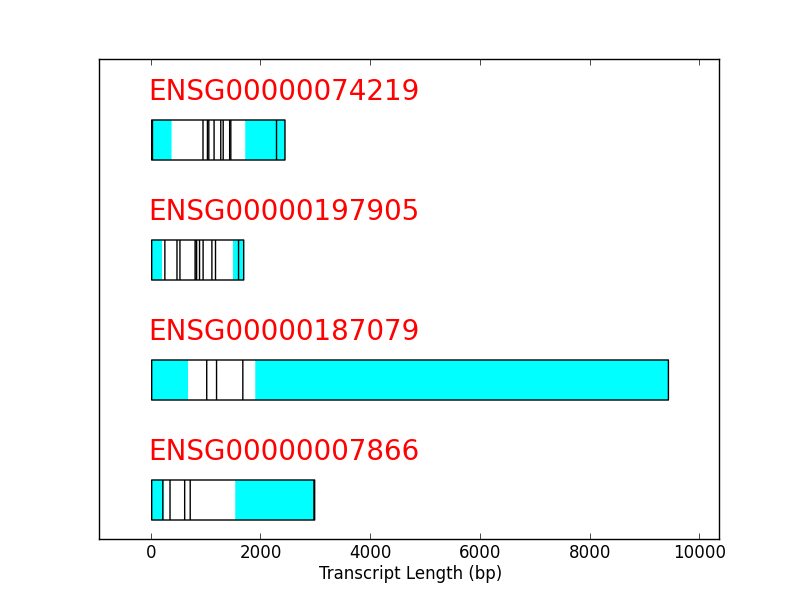

Supplement: Data file S2 [file rsob140029supp3.zip › rsob-14-0029-File010/Melanoma/ENSG00000007866_ENSG00000187079_ENSG00000197905_ENSG00000074219.png]

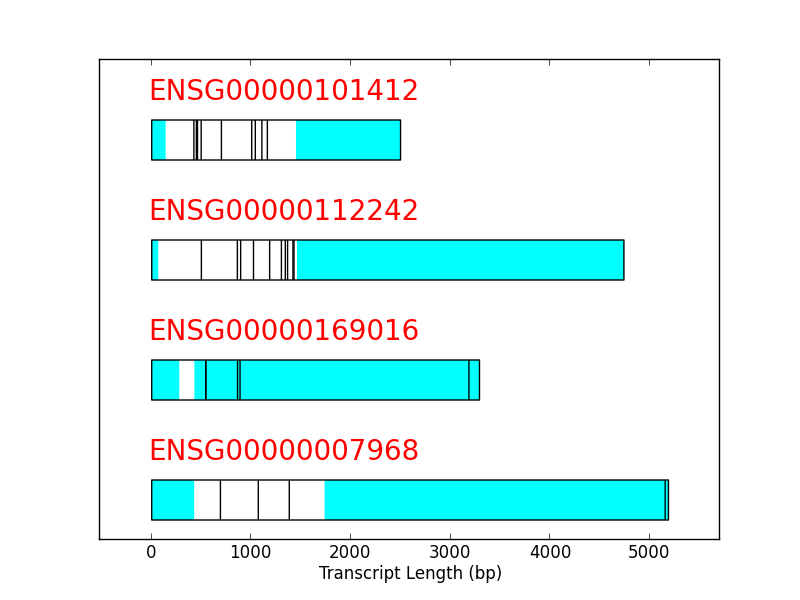

Supplement: Data file S2 [file rsob140029supp3.zip › rsob-14-0029-File010/Melanoma/ENSG00000007968_ENSG00000169016_ENSG00000112242_ENSG00000101412.png]

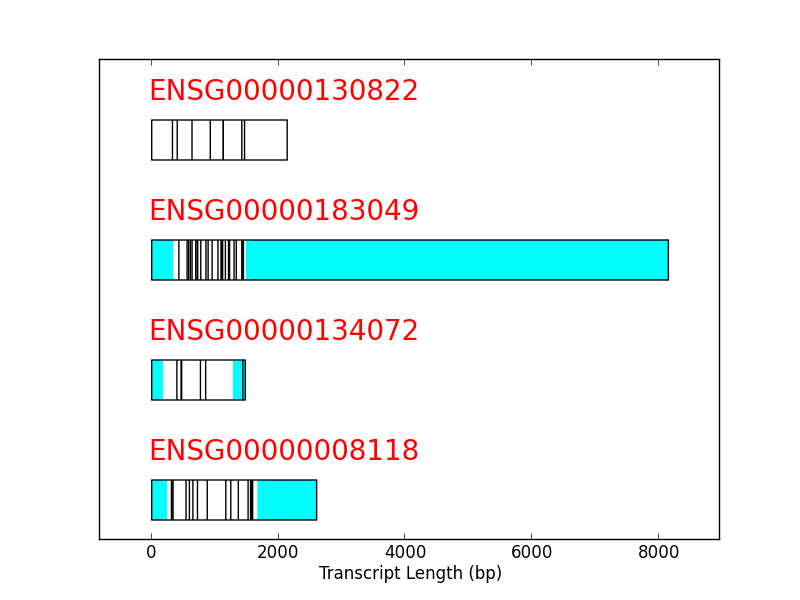

Supplement: Data file S2 [file rsob140029supp3.zip › rsob-14-0029-File010/Melanoma/ENSG00000008118_ENSG00000134072_ENSG00000183049_ENSG00000130822.png]

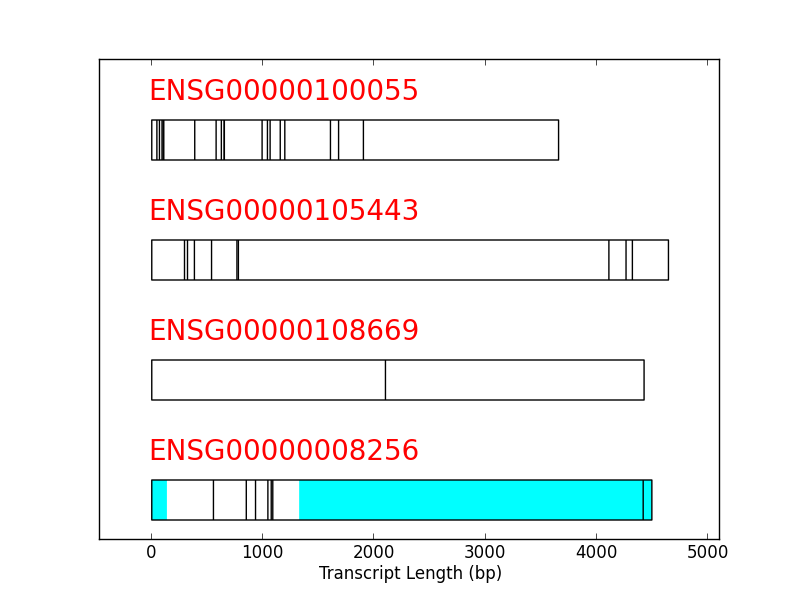

Supplement: Data file S2 [file rsob140029supp3.zip › rsob-14-0029-File010/Melanoma/ENSG00000008256_ENSG00000108669_ENSG00000105443_ENSG00000100055.png]

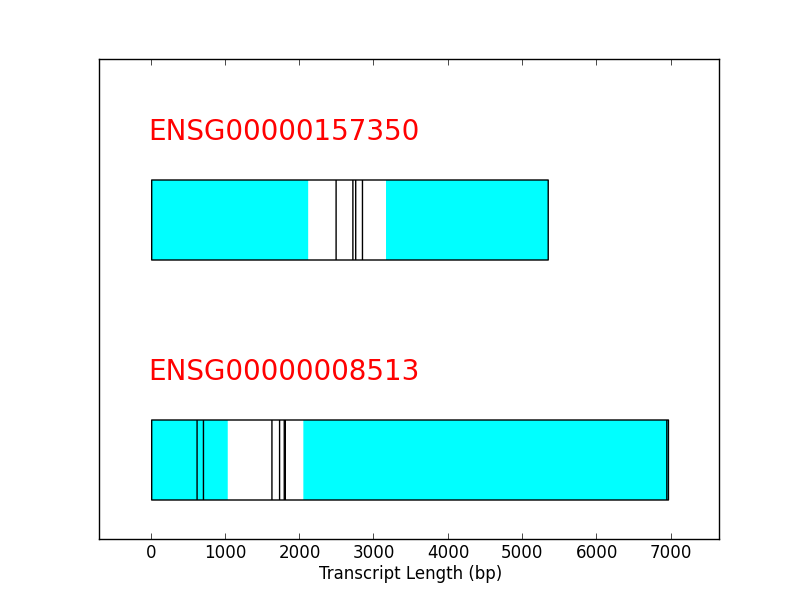

Supplement: Data file S2 [file rsob140029supp3.zip › rsob-14-0029-File010/Melanoma/ENSG00000008513_ENSG00000157350.png]

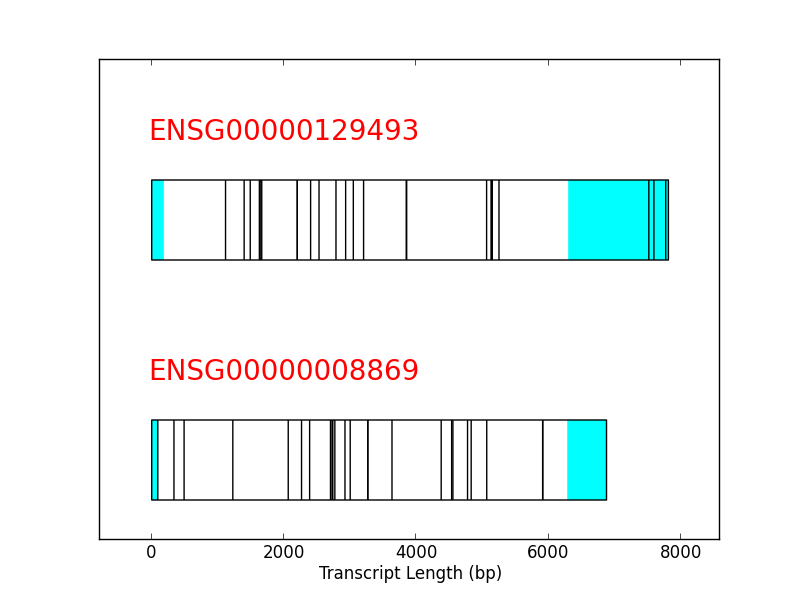

Supplement: Data file S2 [file rsob140029supp3.zip › rsob-14-0029-File010/Melanoma/ENSG00000008869_ENSG00000129493.png]

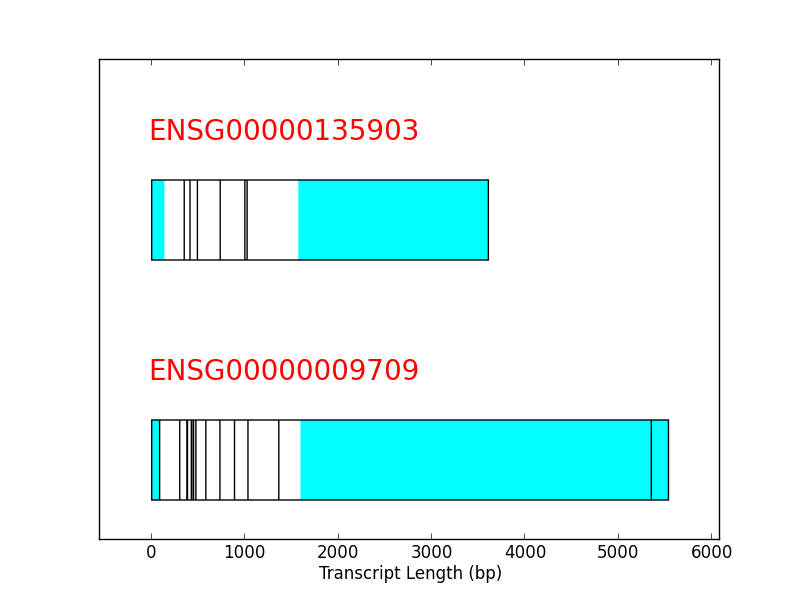

Supplement: Data file S2 [file rsob140029supp3.zip › rsob-14-0029-File010/Melanoma/ENSG00000009709_ENSG00000135903.png]

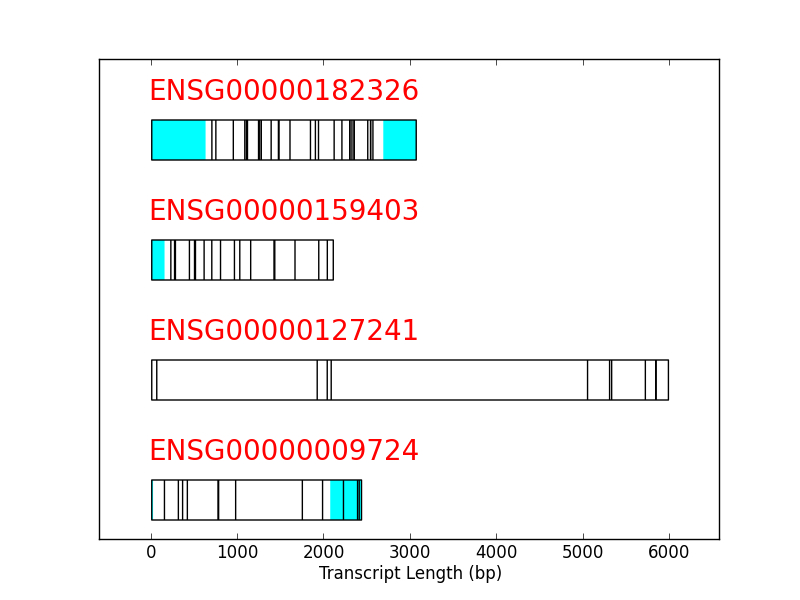

Supplement: Data file S2 [file rsob140029supp3.zip › rsob-14-0029-File010/Melanoma/ENSG00000009724_ENSG00000127241_ENSG00000159403_ENSG00000182326.png]

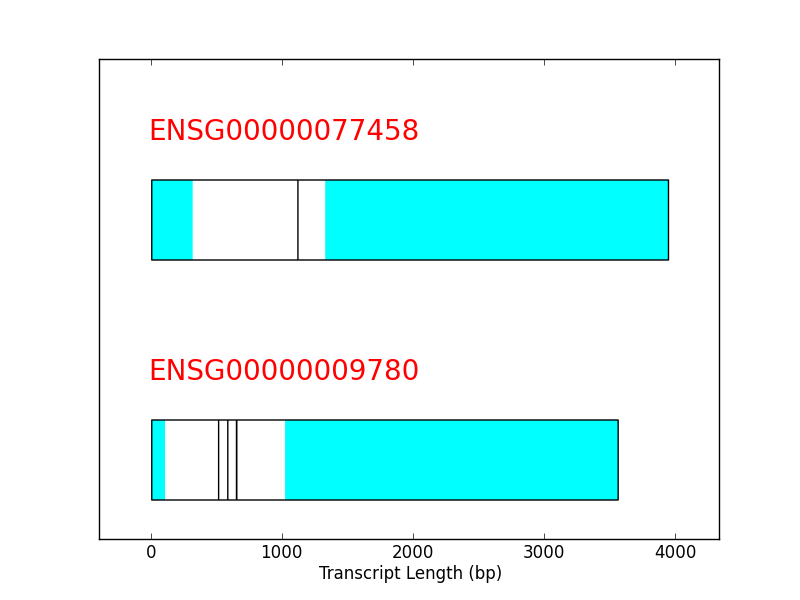

Supplement: Data file S2 [file rsob140029supp3.zip › rsob-14-0029-File010/Melanoma/ENSG00000009780_ENSG00000077458.png]

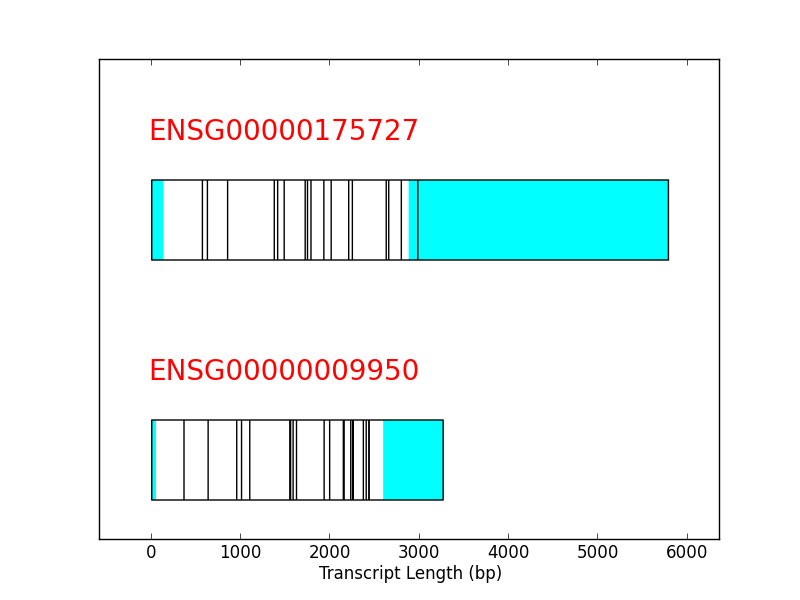

Supplement: Data file S2 [file rsob140029supp3.zip › rsob-14-0029-File010/Melanoma/ENSG00000009950_ENSG00000175727.png]

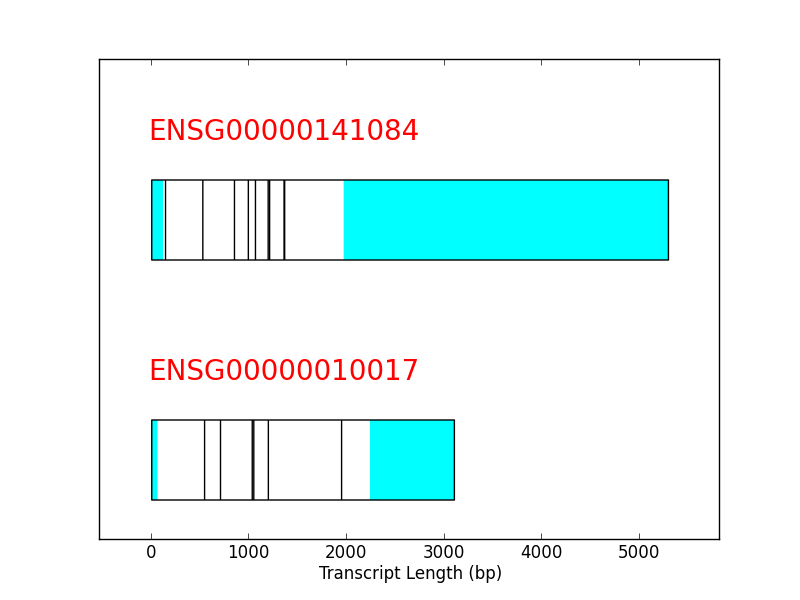

Supplement: Data file S2 [file rsob140029supp3.zip › rsob-14-0029-File010/Melanoma/ENSG00000010017_ENSG00000141084.png]

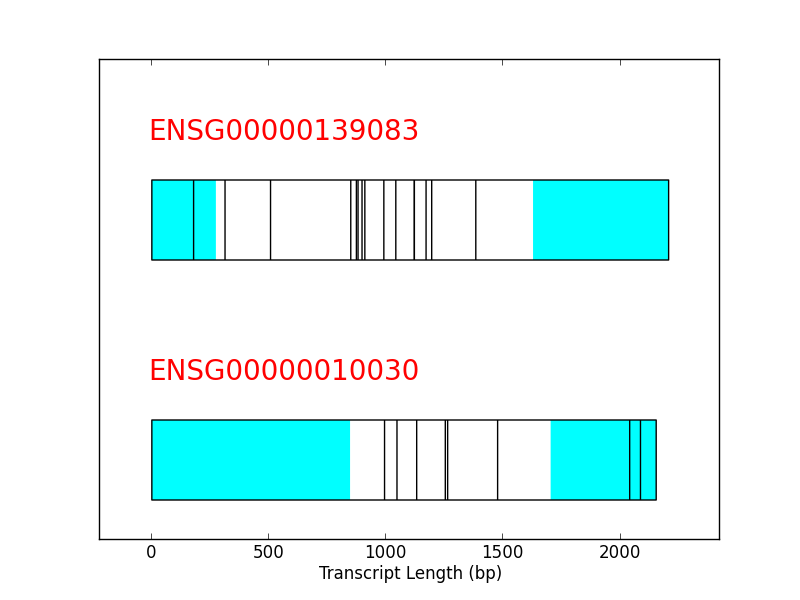

Supplement: Data file S2 [file rsob140029supp3.zip › rsob-14-0029-File010/Melanoma/ENSG00000010030_ENSG00000139083.png]

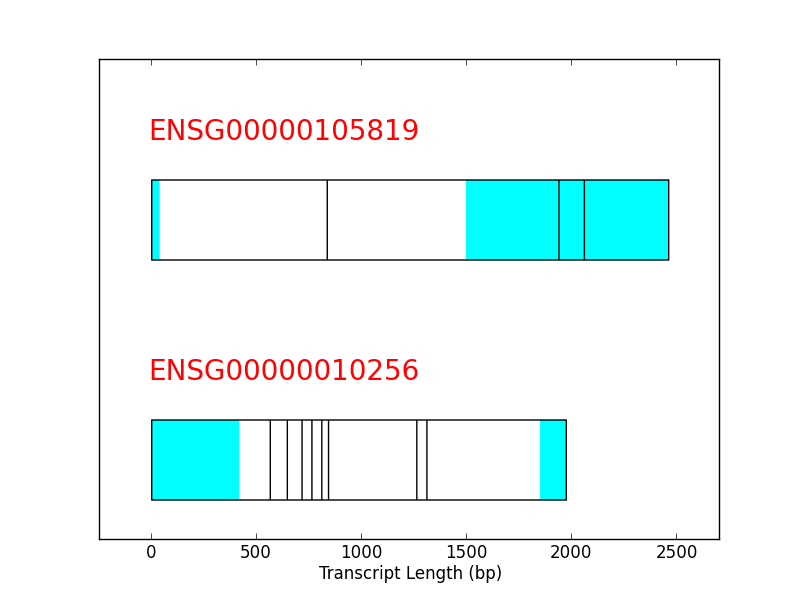

Supplement: Data file S2 [file rsob140029supp3.zip › rsob-14-0029-File010/Melanoma/ENSG00000010256_ENSG00000105819.png]

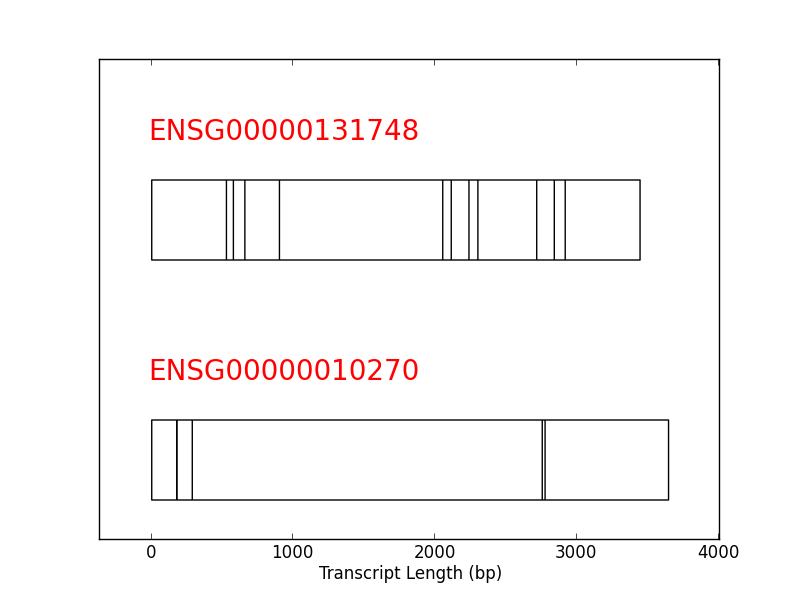

Supplement: Data file S2 [file rsob140029supp3.zip › rsob-14-0029-File010/Melanoma/ENSG00000010270_ENSG00000131748.png]

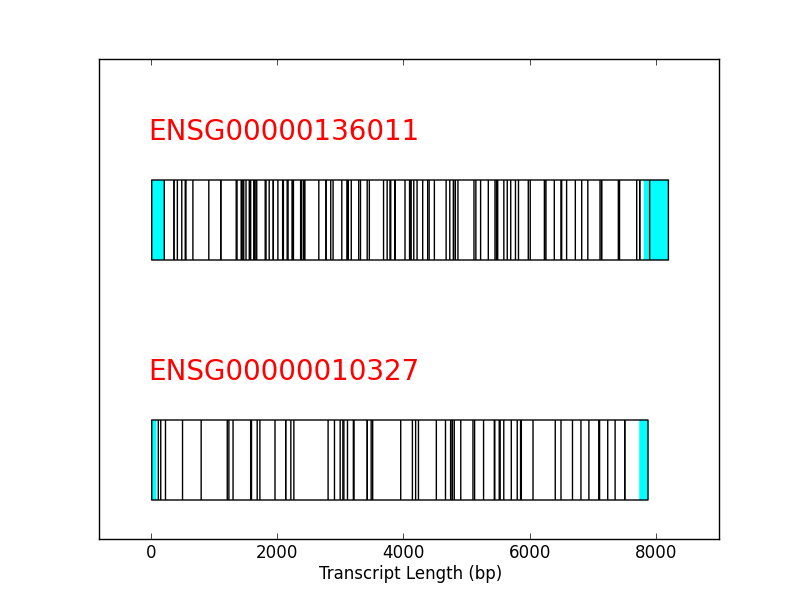

Supplement: Data file S2 [file rsob140029supp3.zip › rsob-14-0029-File010/Melanoma/ENSG00000010327_ENSG00000136011.png]

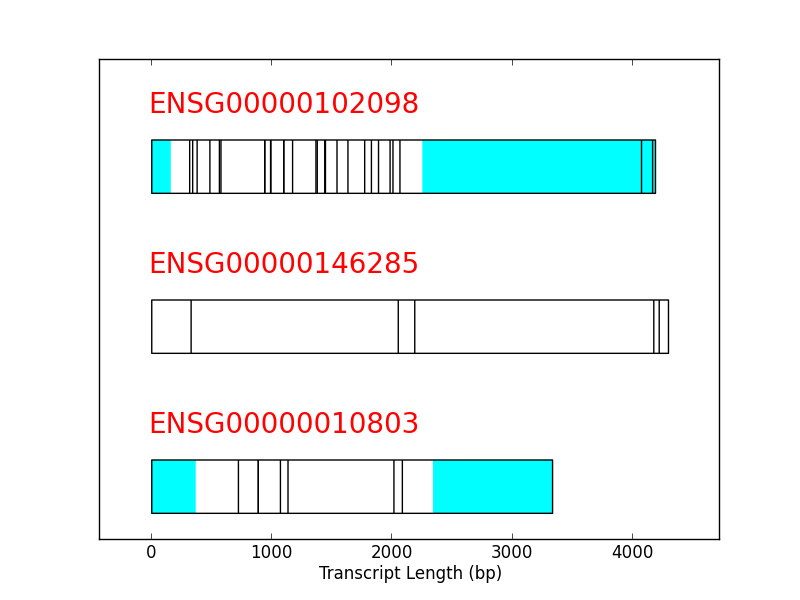

Supplement: Data file S2 [file rsob140029supp3.zip › rsob-14-0029-File010/Melanoma/ENSG00000010803_ENSG00000146285_ENSG00000102098.png]

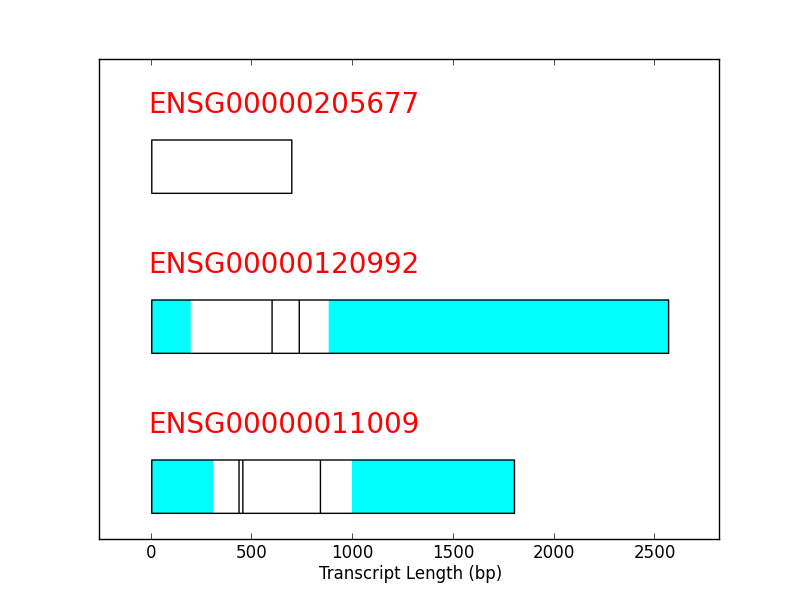

Supplement: Data file S2 [file rsob140029supp3.zip › rsob-14-0029-File010/Melanoma/ENSG00000011009_ENSG00000120992_ENSG00000205677.png]

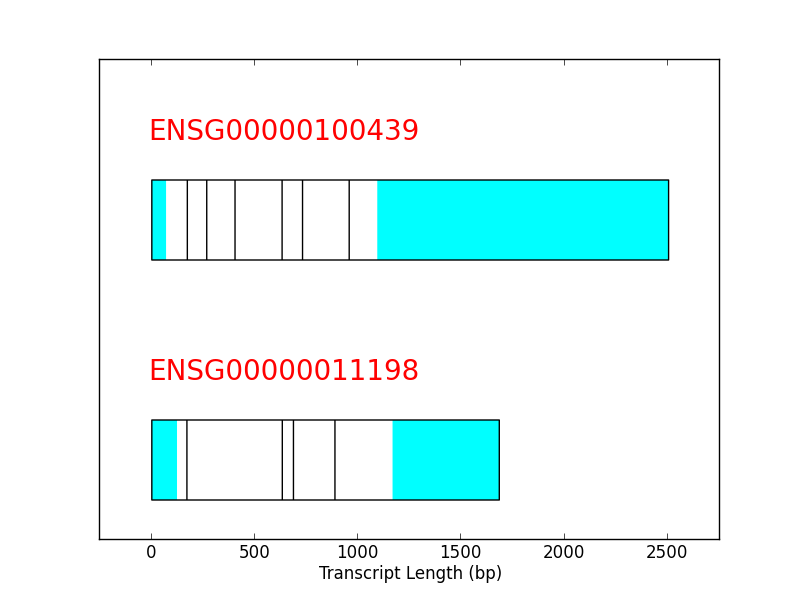

Supplement: Data file S2 [file rsob140029supp3.zip › rsob-14-0029-File010/Melanoma/ENSG00000011198_ENSG00000100439.png]

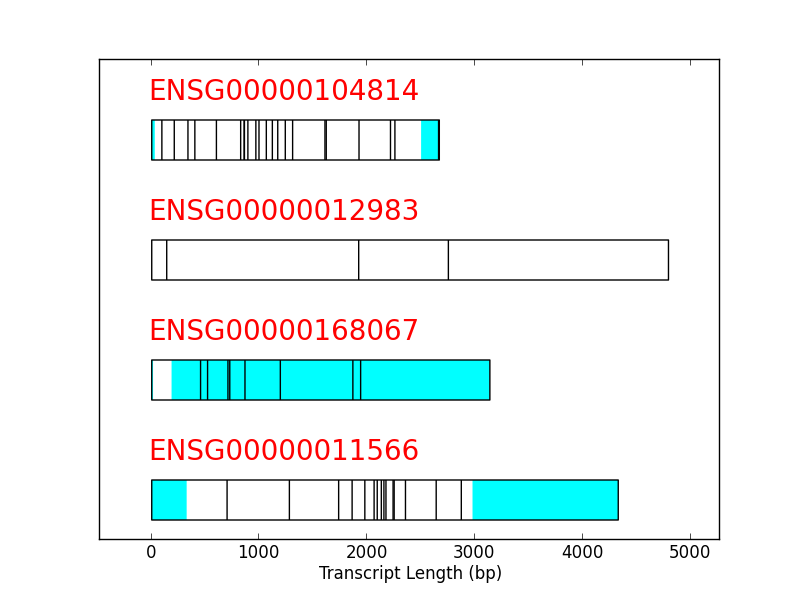

Supplement: Data file S2 [file rsob140029supp3.zip › rsob-14-0029-File010/Melanoma/ENSG00000011566_ENSG00000168067_ENSG00000012983_ENSG00000104814.png]

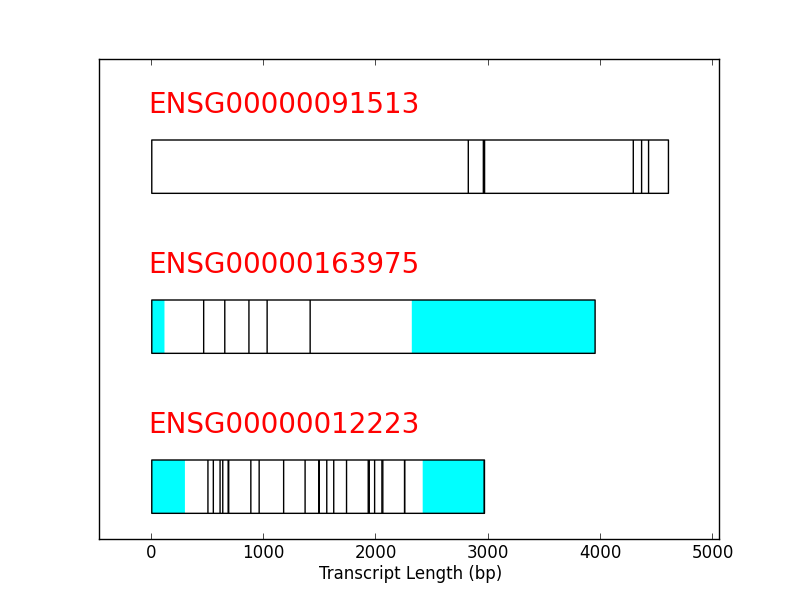

Supplement: Data file S2 [file rsob140029supp3.zip › rsob-14-0029-File010/Melanoma/ENSG00000012223_ENSG00000163975_ENSG00000091513.png]

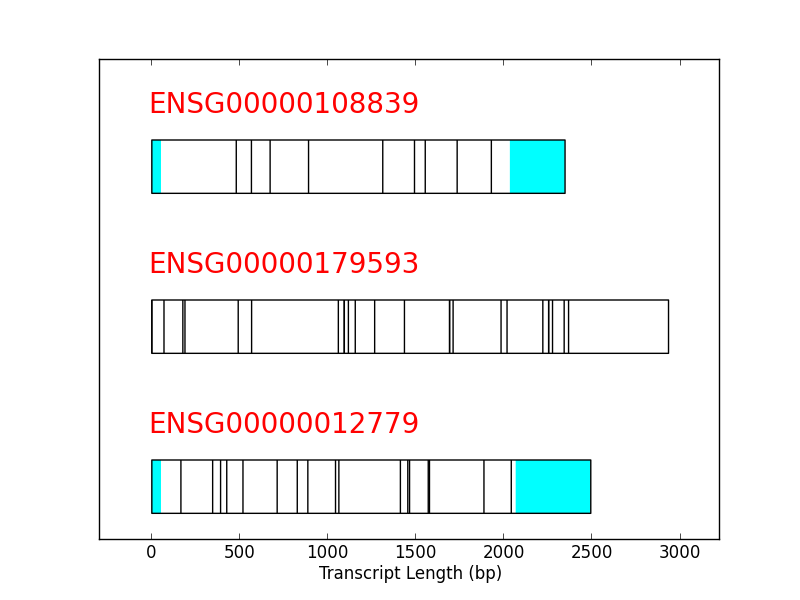

Supplement: Data file S2 [file rsob140029supp3.zip › rsob-14-0029-File010/Melanoma/ENSG00000012779_ENSG00000179593_ENSG00000108839.png]

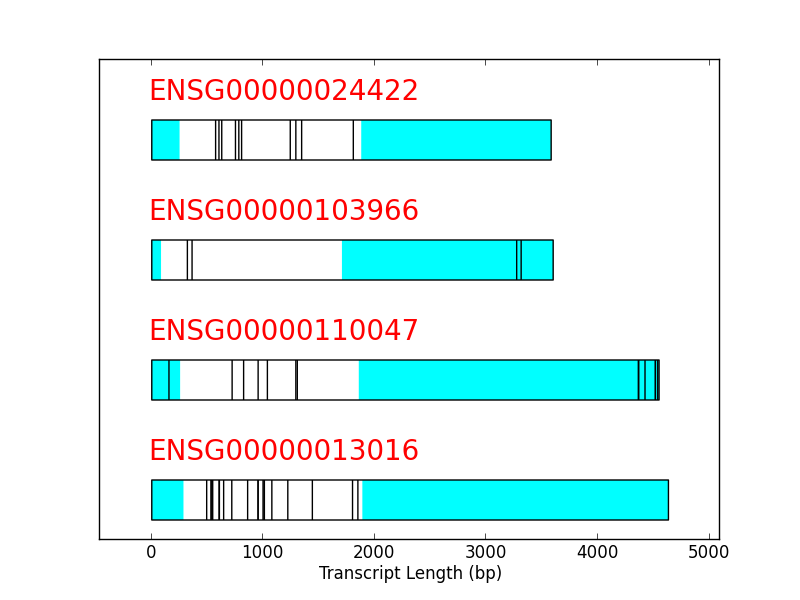

Supplement: Data file S2 [file rsob140029supp3.zip › rsob-14-0029-File010/Melanoma/ENSG00000013016_ENSG00000110047_ENSG00000103966_ENSG00000024422.png]

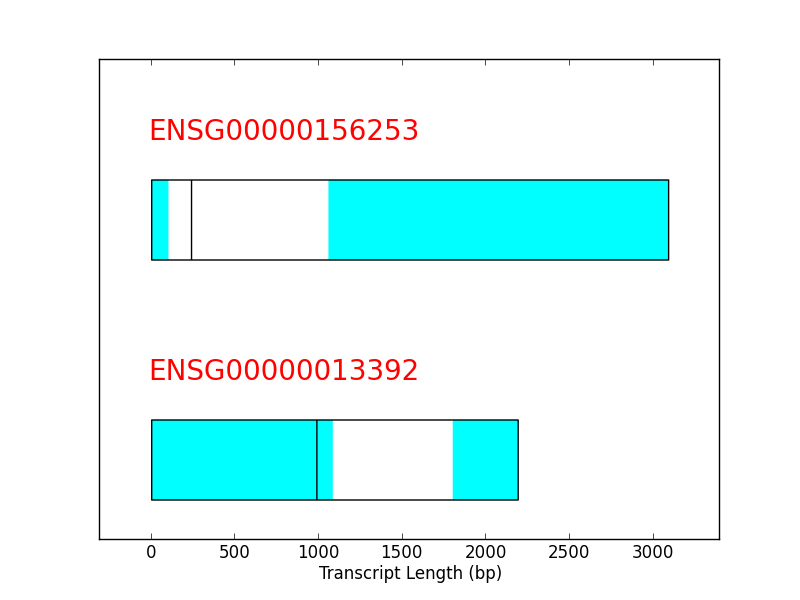

Supplement: Data file S2 [file rsob140029supp3.zip › rsob-14-0029-File010/Melanoma/ENSG00000013392_ENSG00000156253.png]

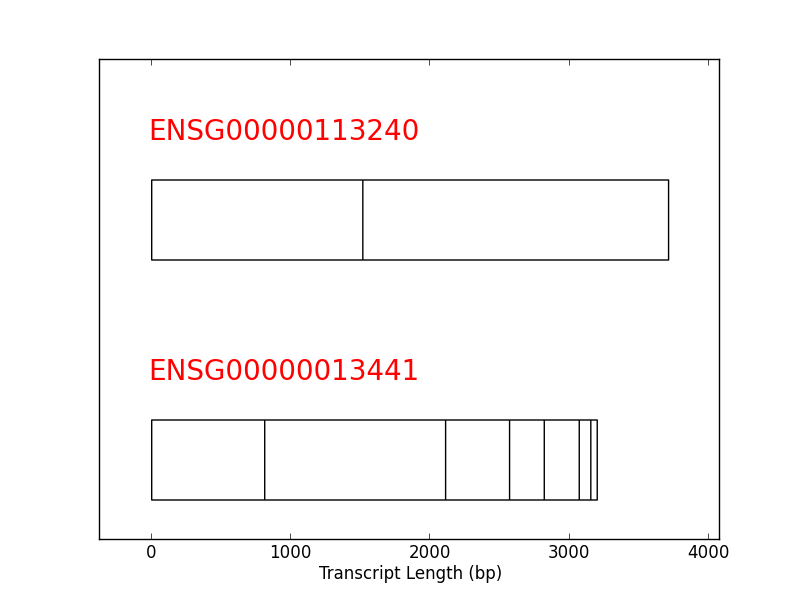

Supplement: Data file S2 [file rsob140029supp3.zip › rsob-14-0029-File010/Melanoma/ENSG00000013441_ENSG00000113240.png]

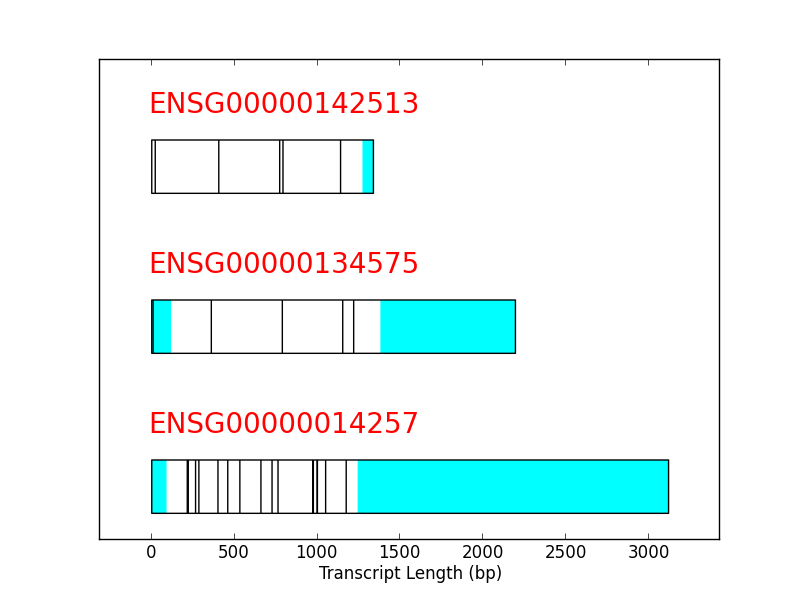

Supplement: Data file S2 [file rsob140029supp3.zip › rsob-14-0029-File010/Melanoma/ENSG00000014257_ENSG00000134575_ENSG00000142513.png]

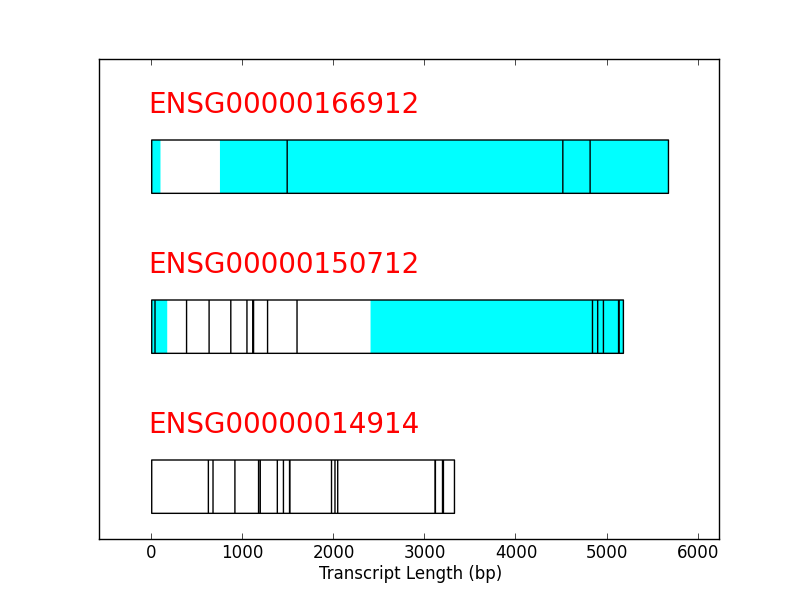

Supplement: Data file S2 [file rsob140029supp3.zip › rsob-14-0029-File010/Melanoma/ENSG00000014914_ENSG00000150712_ENSG00000166912.png]

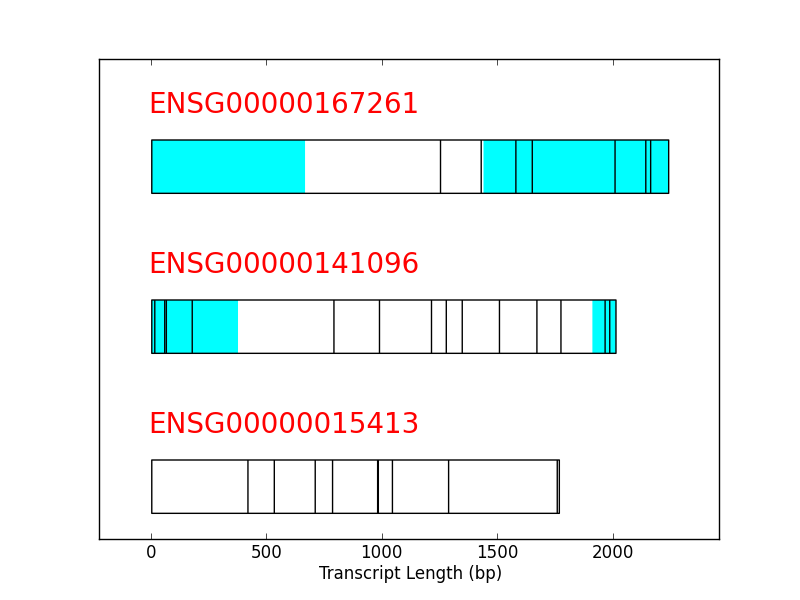

Supplement: Data file S2 [file rsob140029supp3.zip › rsob-14-0029-File010/Melanoma/ENSG00000015413_ENSG00000141096_ENSG00000167261.png]

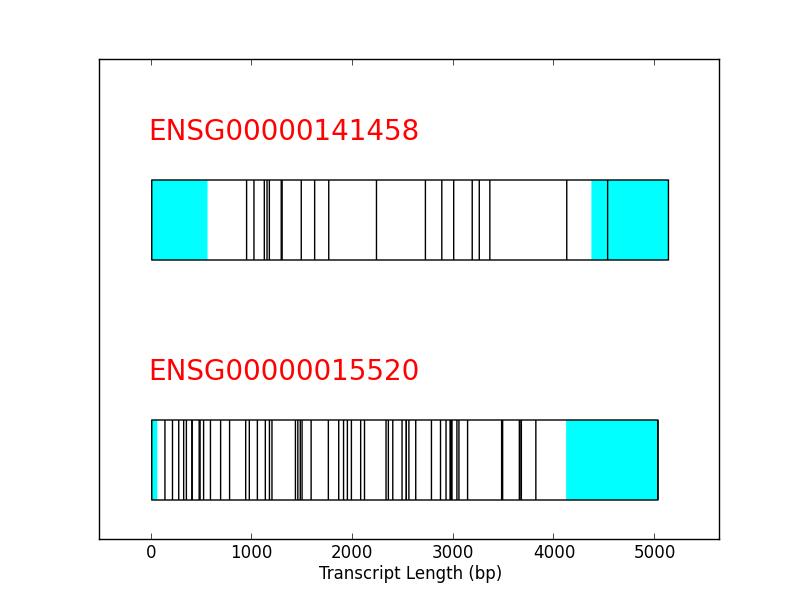

Supplement: Data file S2 [file rsob140029supp3.zip › rsob-14-0029-File010/Melanoma/ENSG00000015520_ENSG00000141458.png]

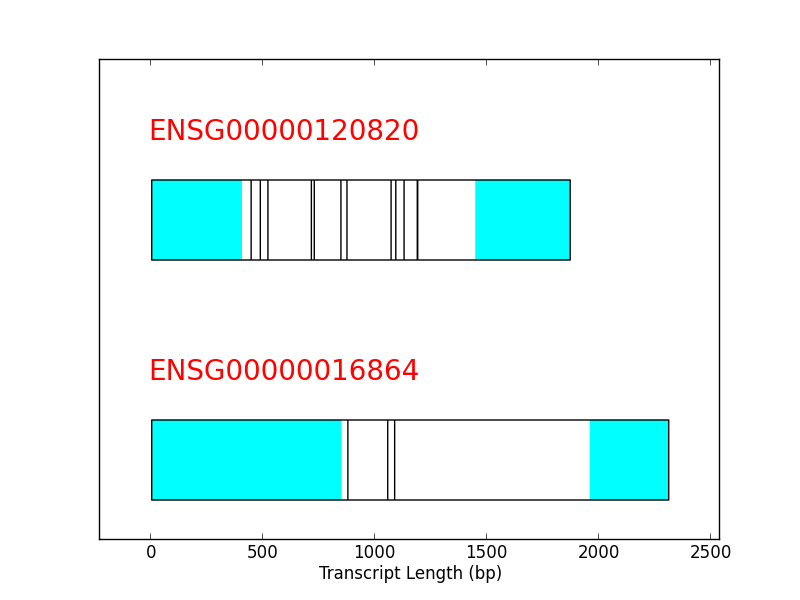

Supplement: Data file S2 [file rsob140029supp3.zip › rsob-14-0029-File010/Melanoma/ENSG00000016864_ENSG00000120820.png]

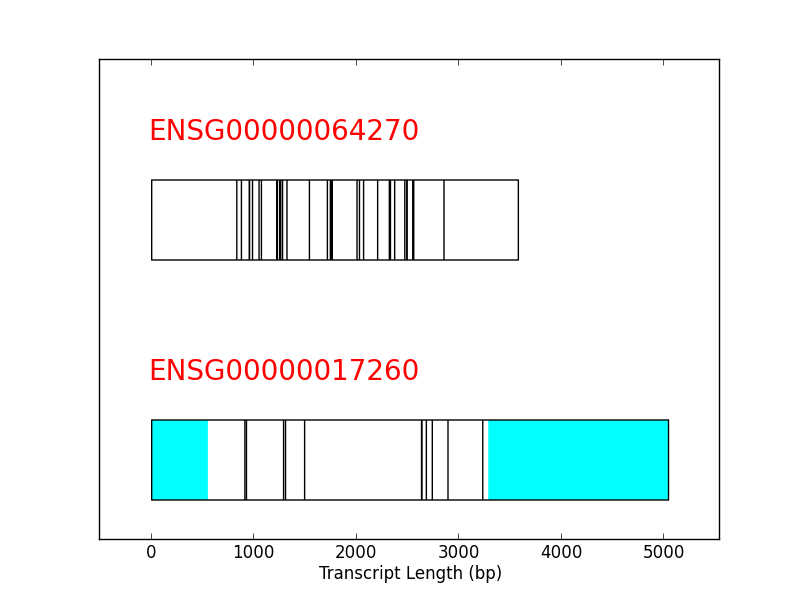

Supplement: Data file S2 [file rsob140029supp3.zip › rsob-14-0029-File010/Melanoma/ENSG00000017260_ENSG00000064270.png]

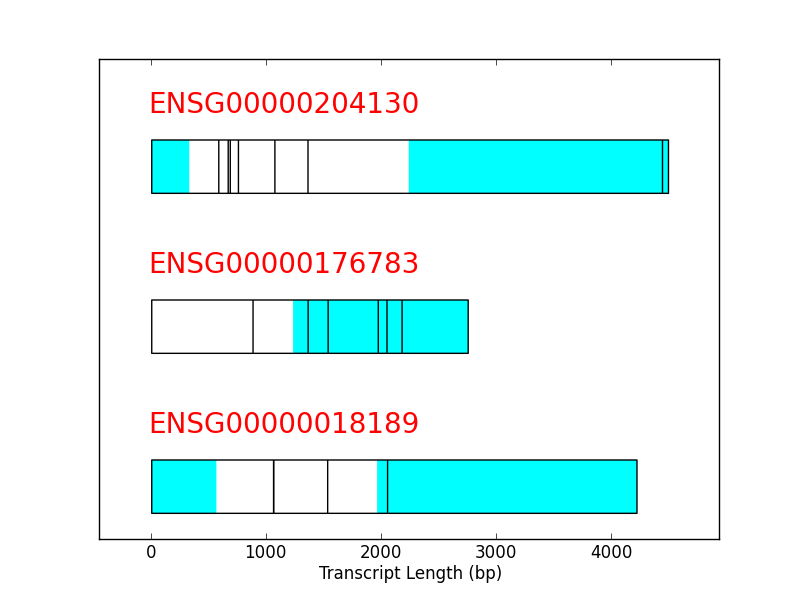

Supplement: Data file S2 [file rsob140029supp3.zip › rsob-14-0029-File010/Melanoma/ENSG00000018189_ENSG00000176783_ENSG00000204130.png]

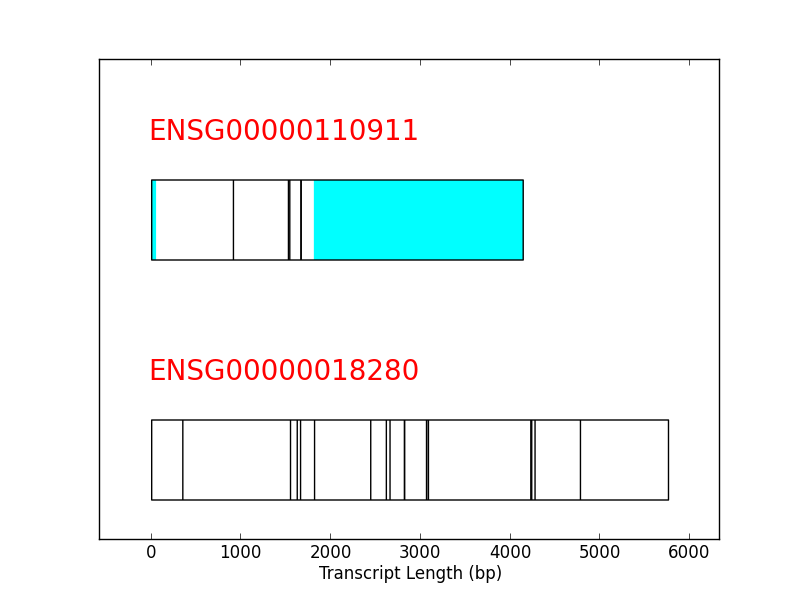

Supplement: Data file S2 [file rsob140029supp3.zip › rsob-14-0029-File010/Melanoma/ENSG00000018280_ENSG00000110911.png]

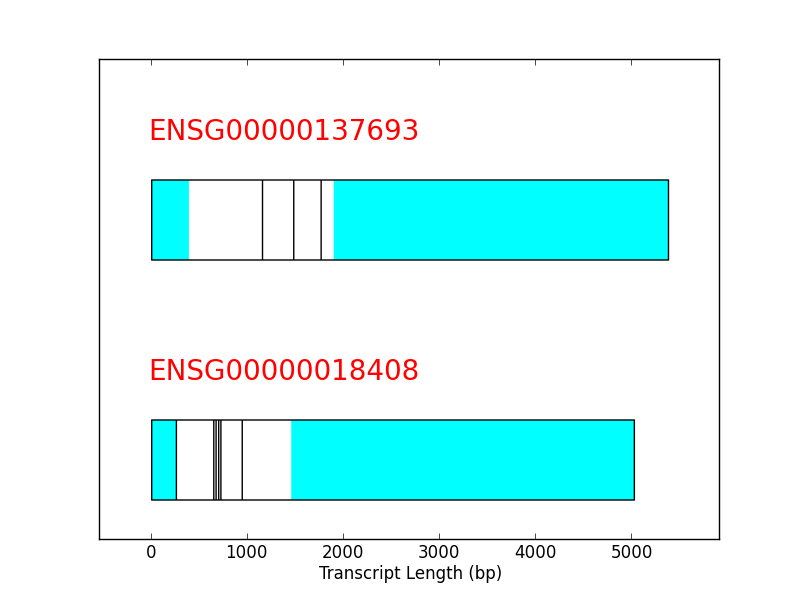

Supplement: Data file S2 [file rsob140029supp3.zip › rsob-14-0029-File010/Melanoma/ENSG00000018408_ENSG00000137693.png]

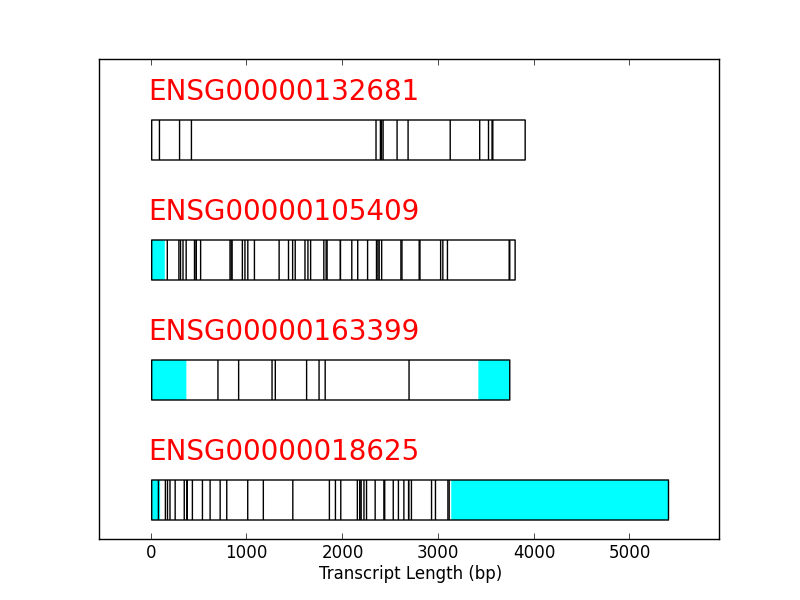

Supplement: Data file S2 [file rsob140029supp3.zip › rsob-14-0029-File010/Melanoma/ENSG00000018625_ENSG00000163399_ENSG00000105409_ENSG00000132681.png]

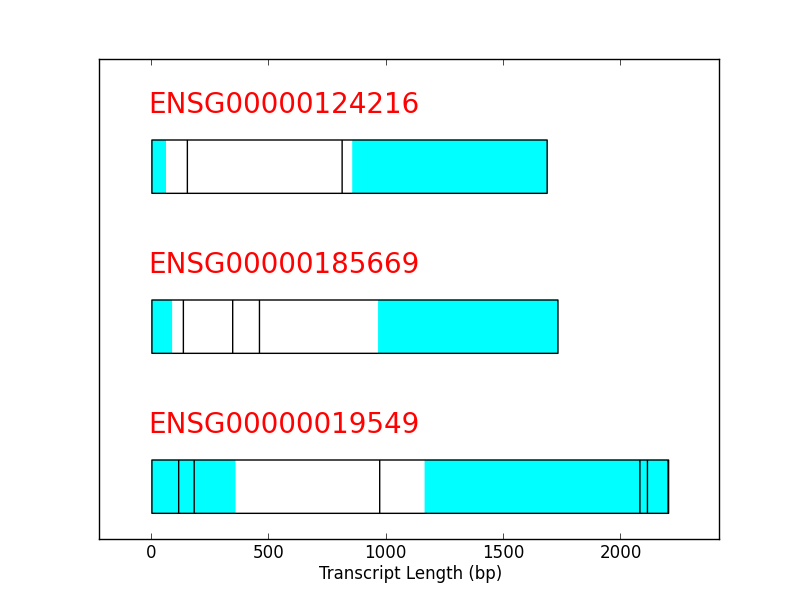

Supplement: Data file S2 [file rsob140029supp3.zip › rsob-14-0029-File010/Melanoma/ENSG00000019549_ENSG00000185669_ENSG00000124216.png]

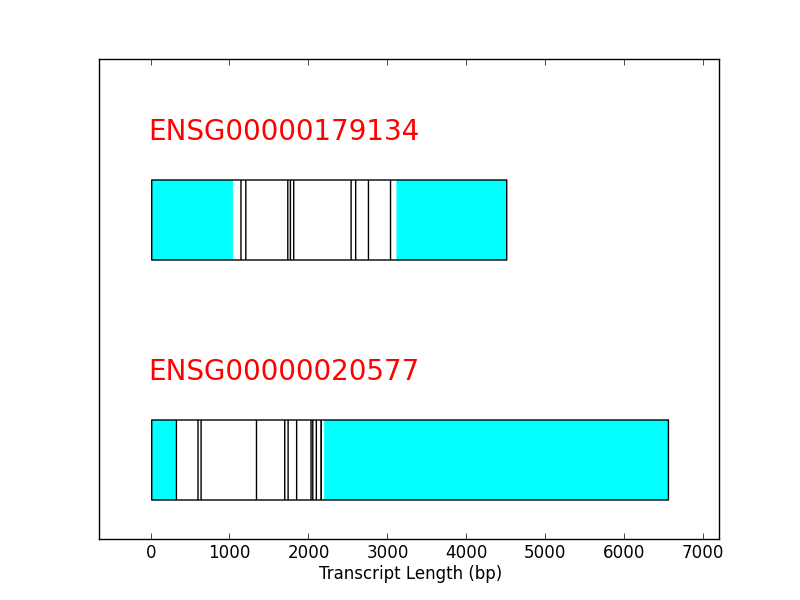

Supplement: Data file S2 [file rsob140029supp3.zip › rsob-14-0029-File010/Melanoma/ENSG00000020577_ENSG00000179134.png]

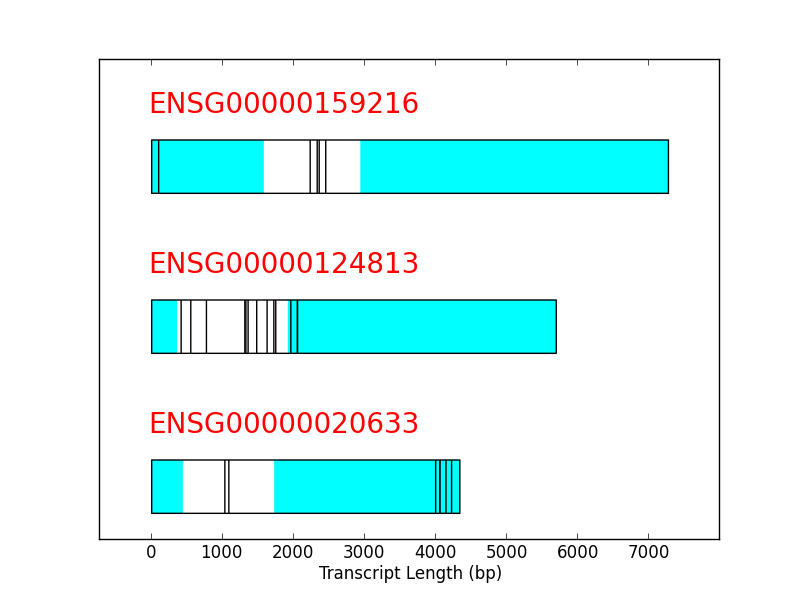

Supplement: Data file S2 [file rsob140029supp3.zip › rsob-14-0029-File010/Melanoma/ENSG00000020633_ENSG00000124813_ENSG00000159216.png]

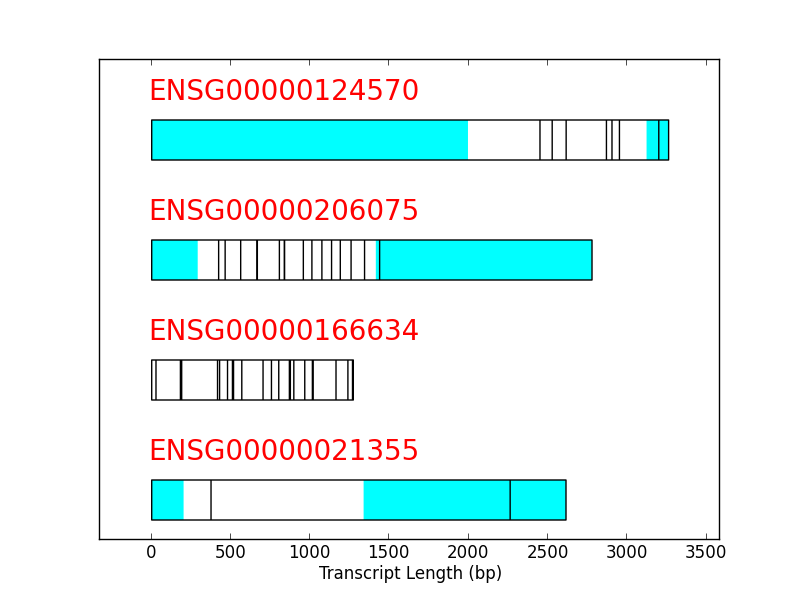

Supplement: Data file S2 [file rsob140029supp3.zip › rsob-14-0029-File010/Melanoma/ENSG00000021355_ENSG00000166634_ENSG00000206075_ENSG00000124570.png]

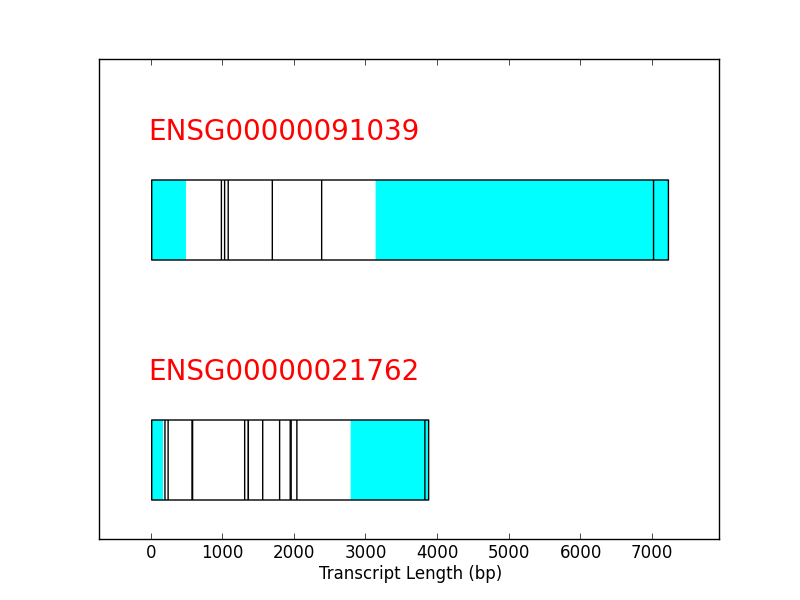

Supplement: Data file S2 [file rsob140029supp3.zip › rsob-14-0029-File010/Melanoma/ENSG00000021762_ENSG00000091039.png]

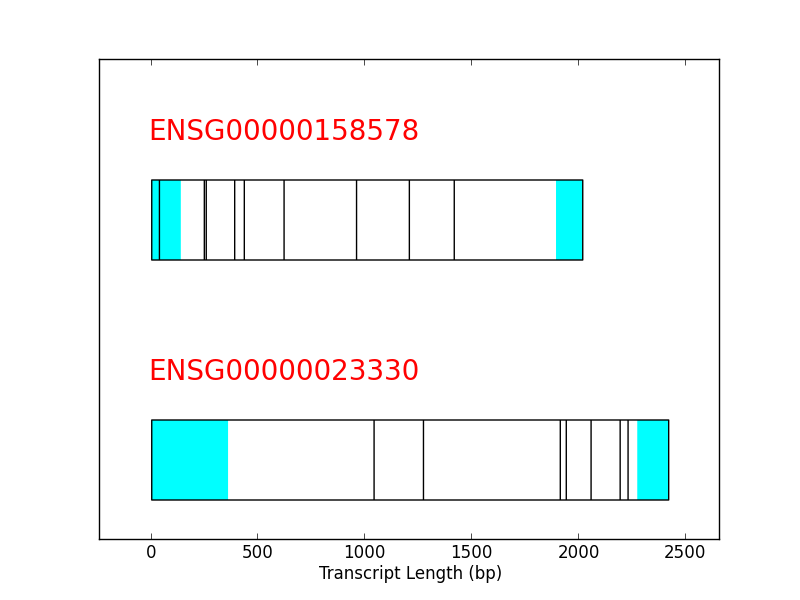

Supplement: Data file S2 [file rsob140029supp3.zip › rsob-14-0029-File010/Melanoma/ENSG00000023330_ENSG00000158578.png]

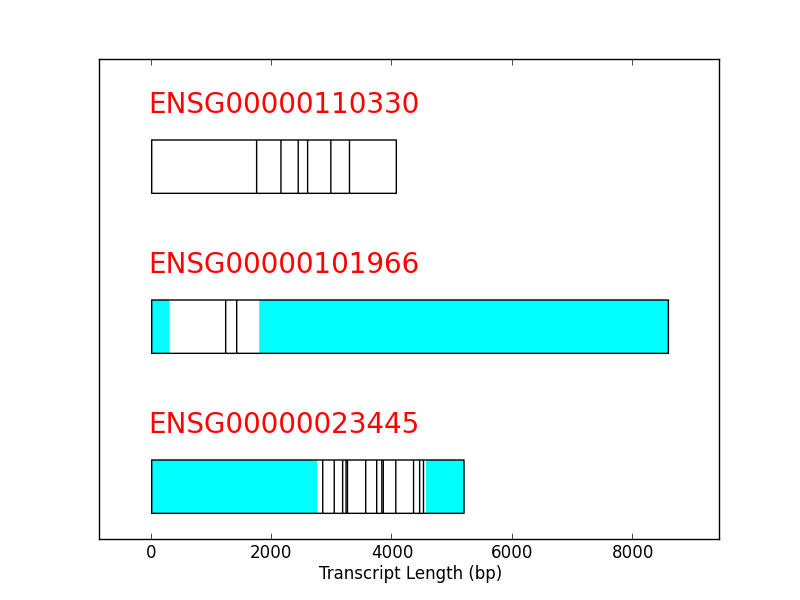

Supplement: Data file S2 [file rsob140029supp3.zip › rsob-14-0029-File010/Melanoma/ENSG00000023445_ENSG00000101966_ENSG00000110330.png]

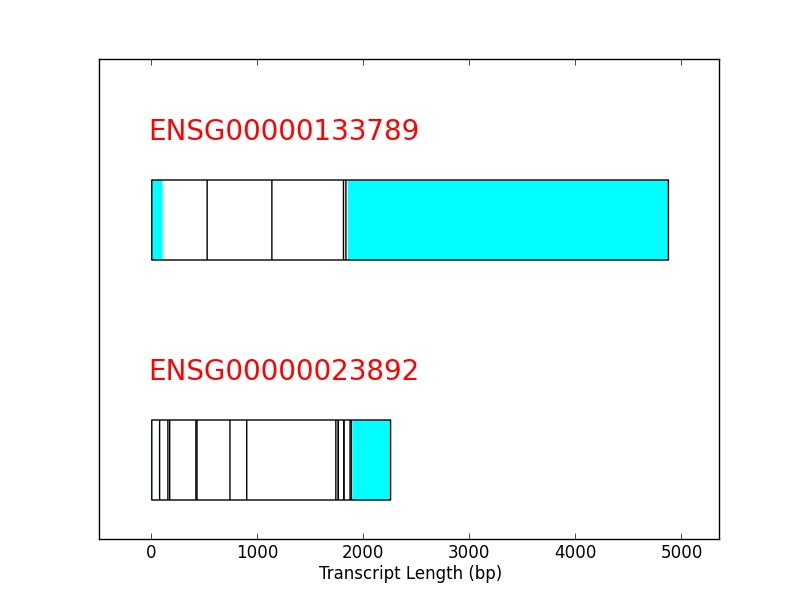

Supplement: Data file S2 [file rsob140029supp3.zip › rsob-14-0029-File010/Melanoma/ENSG00000023892_ENSG00000133789.png]

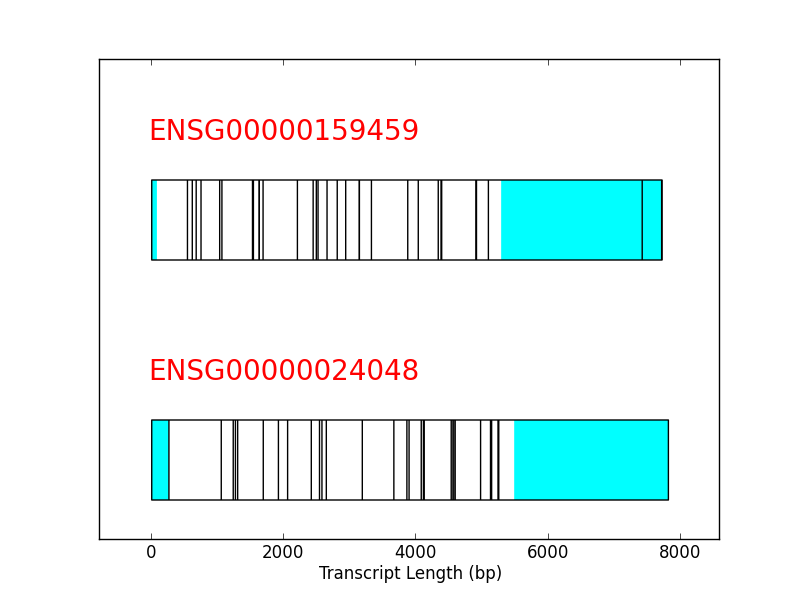

Supplement: Data file S2 [file rsob140029supp3.zip › rsob-14-0029-File010/Melanoma/ENSG00000024048_ENSG00000159459.png]

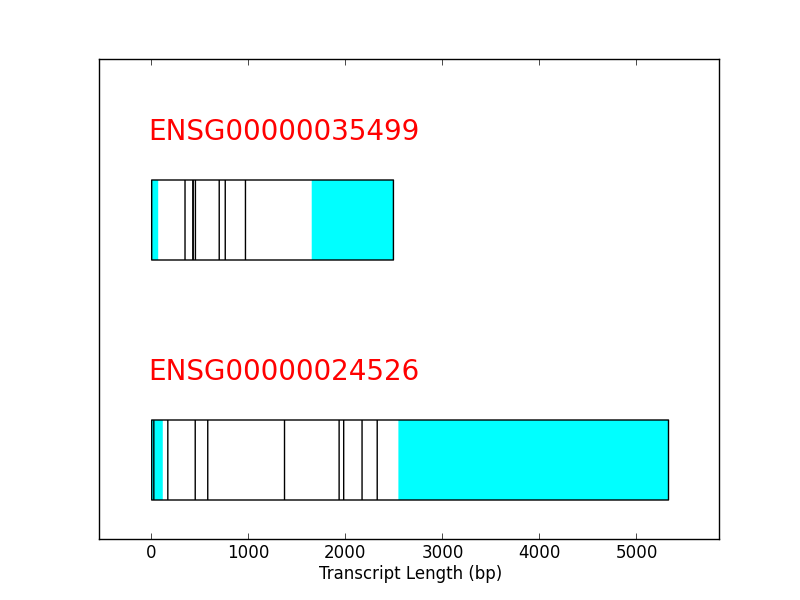

Supplement: Data file S2 [file rsob140029supp3.zip › rsob-14-0029-File010/Melanoma/ENSG00000024526_ENSG00000035499.png]

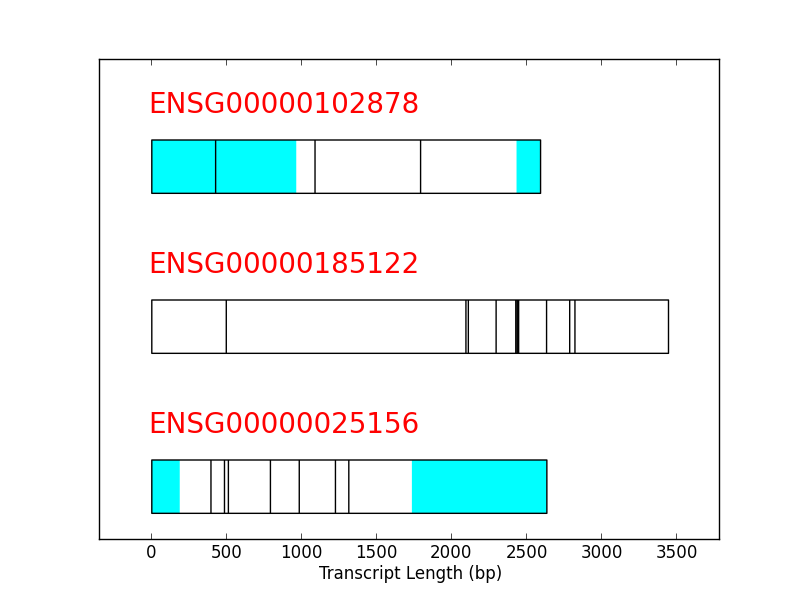

Supplement: Data file S2 [file rsob140029supp3.zip › rsob-14-0029-File010/Melanoma/ENSG00000025156_ENSG00000185122_ENSG00000102878.png]

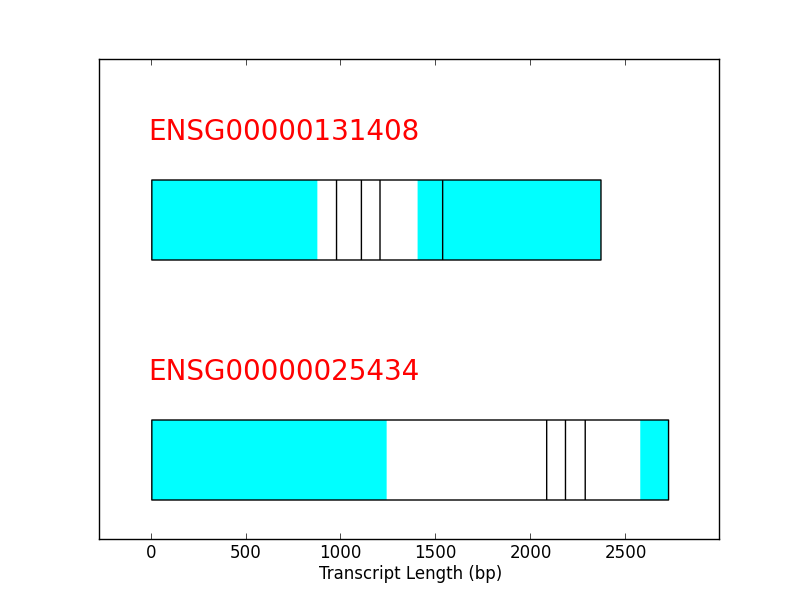

Supplement: Data file S2 [file rsob140029supp3.zip › rsob-14-0029-File010/Melanoma/ENSG00000025434_ENSG00000131408.png]

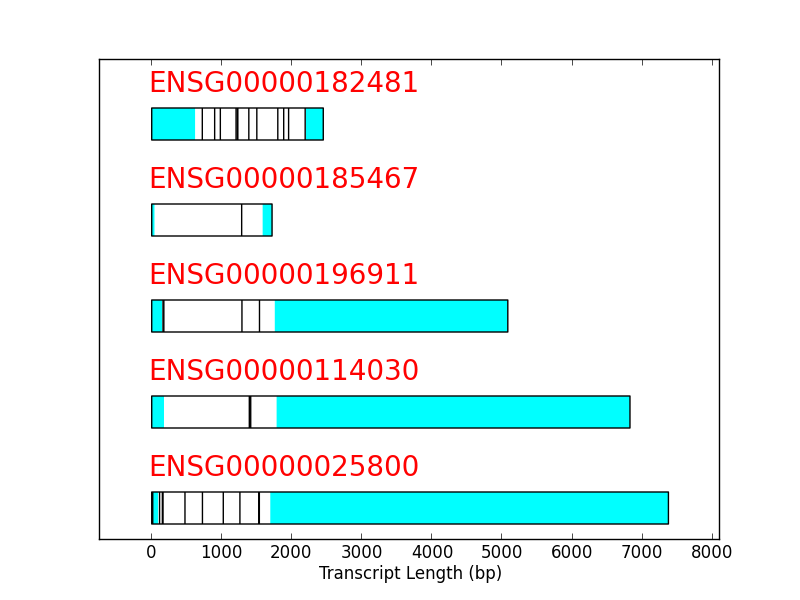

Supplement: Data file S2 [file rsob140029supp3.zip › rsob-14-0029-File010/Melanoma/ENSG00000025800_ENSG00000114030_ENSG00000196911_ENSG00000185467_ENSG00000182481.png]

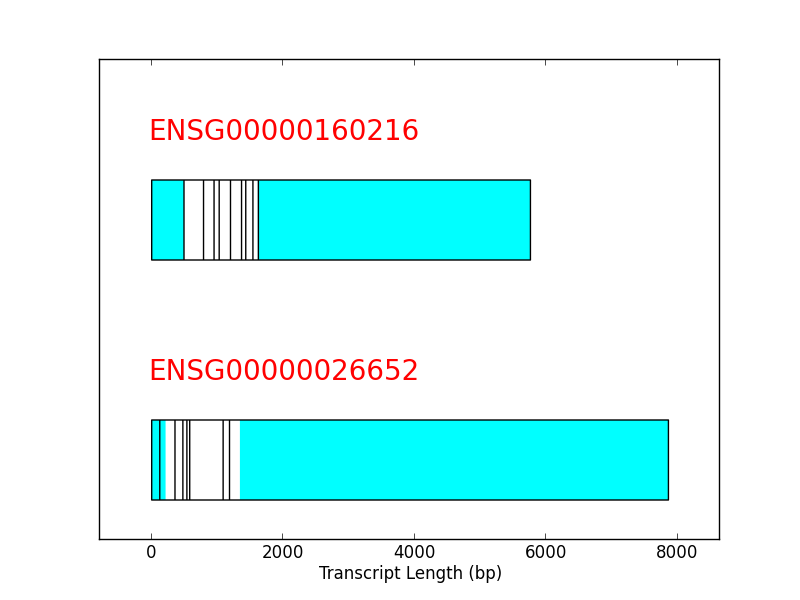

Supplement: Data file S2 [file rsob140029supp3.zip › rsob-14-0029-File010/Melanoma/ENSG00000026652_ENSG00000160216.png]

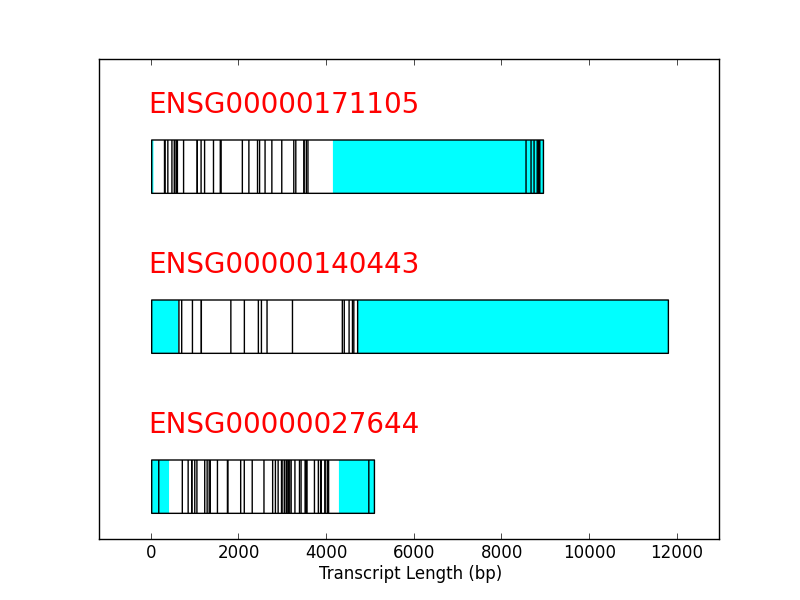

Supplement: Data file S2 [file rsob140029supp3.zip › rsob-14-0029-File010/Melanoma/ENSG00000027644_ENSG00000140443_ENSG00000171105.png]

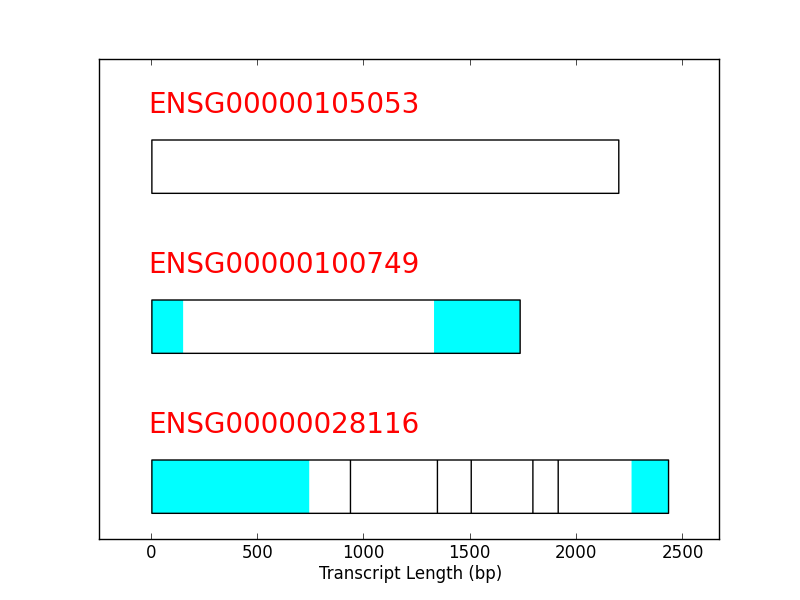

Supplement: Data file S2 [file rsob140029supp3.zip › rsob-14-0029-File010/Melanoma/ENSG00000028116_ENSG00000100749_ENSG00000105053.png]
