# Supplementary figures and images for: Identification of 2R-ohnologue gene families displaying the same mutation-load skew in multiple cancers (part 3 of 3)
Source: Open Biol. 2014 May 7;4(5):140029. doi: 10.1098/rsob.140029 (PMC4042849; doi:10.1098/rsob.140029)

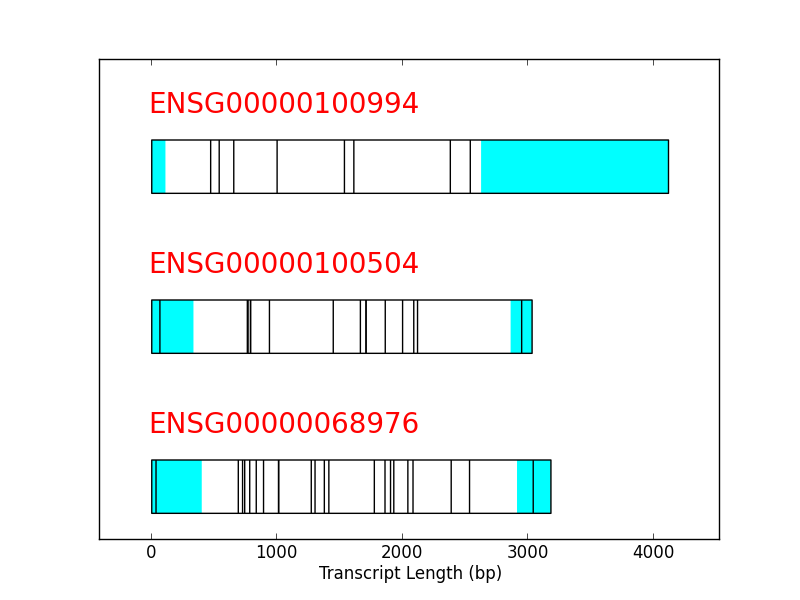

Supplement: Data file S2 [file rsob140029supp3.zip › rsob-14-0029-File010/Melanoma/ENSG00000068976_ENSG00000100504_ENSG00000100994.png]

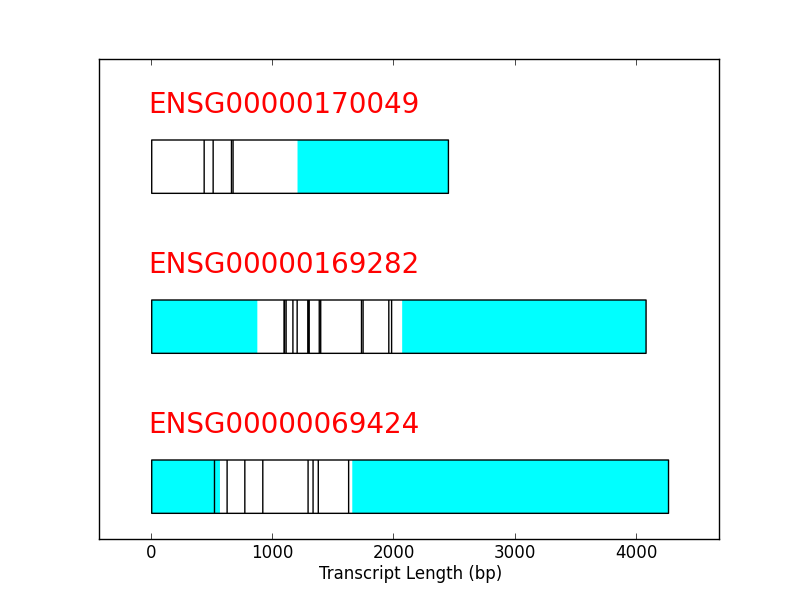

Supplement: Data file S2 [file rsob140029supp3.zip › rsob-14-0029-File010/Melanoma/ENSG00000069424_ENSG00000169282_ENSG00000170049.png]

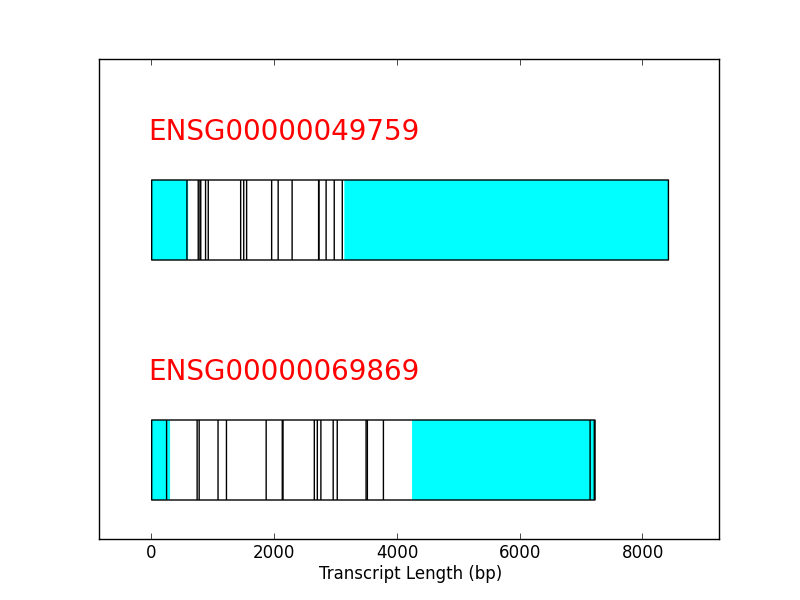

Supplement: Data file S2 [file rsob140029supp3.zip › rsob-14-0029-File010/Melanoma/ENSG00000069869_ENSG00000049759.png]

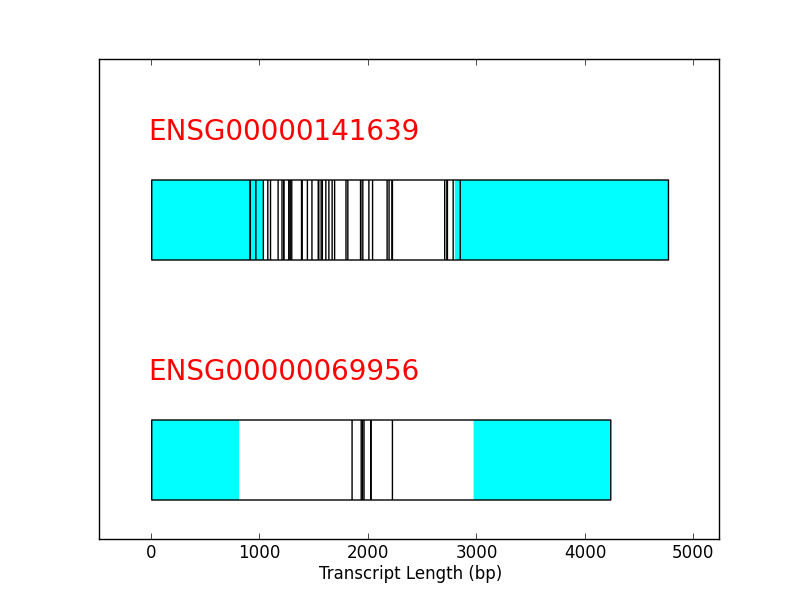

Supplement: Data file S2 [file rsob140029supp3.zip › rsob-14-0029-File010/Melanoma/ENSG00000069956_ENSG00000141639.png]

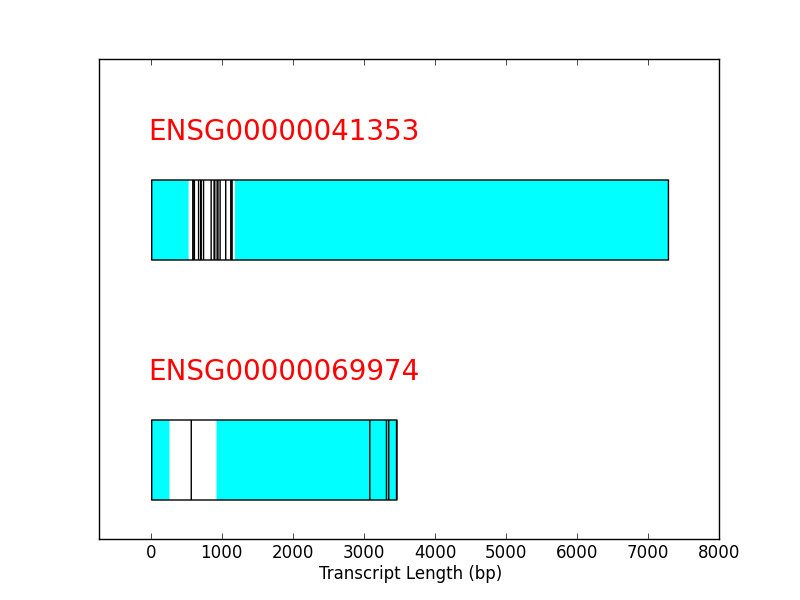

Supplement: Data file S2 [file rsob140029supp3.zip › rsob-14-0029-File010/Melanoma/ENSG00000069974_ENSG00000041353.png]

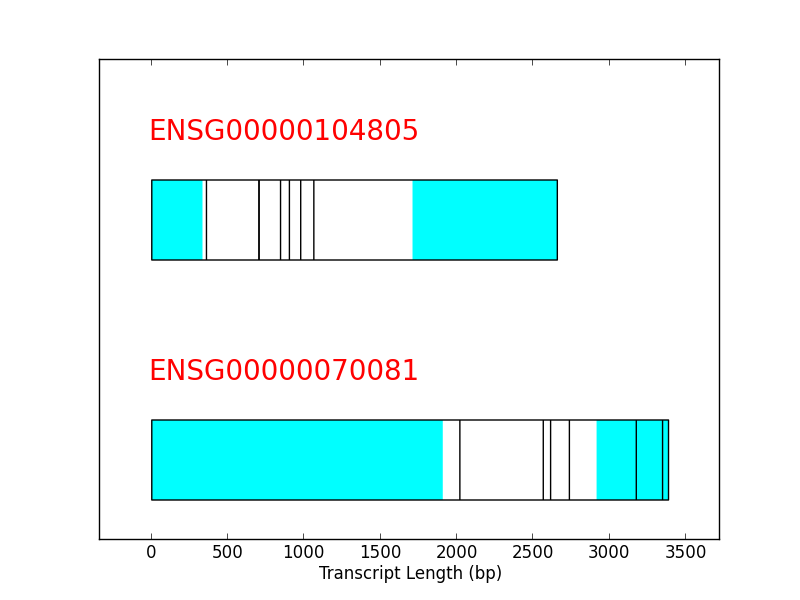

Supplement: Data file S2 [file rsob140029supp3.zip › rsob-14-0029-File010/Melanoma/ENSG00000070081_ENSG00000104805.png]

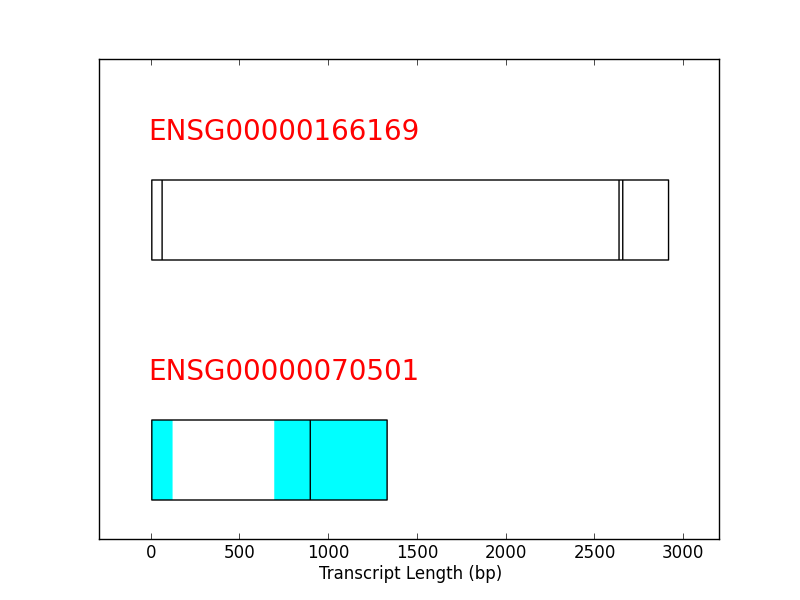

Supplement: Data file S2 [file rsob140029supp3.zip › rsob-14-0029-File010/Melanoma/ENSG00000070501_ENSG00000166169.png]

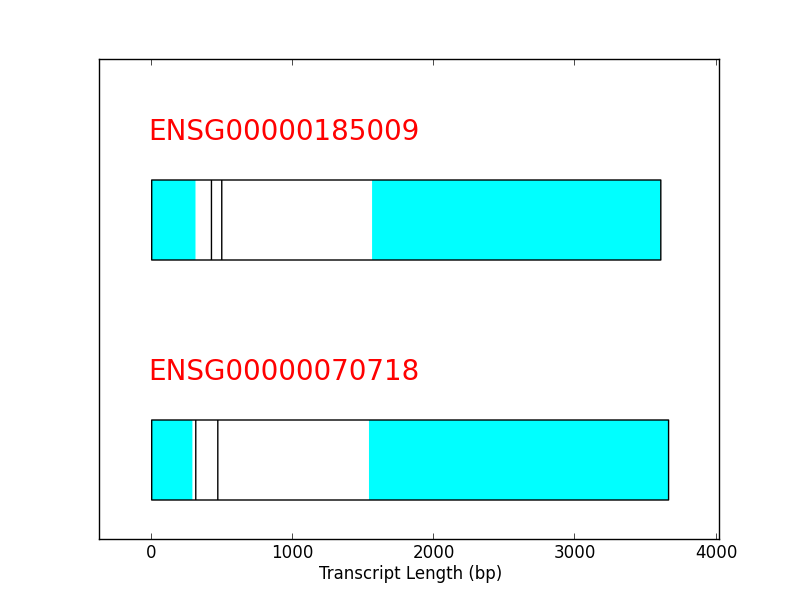

Supplement: Data file S2 [file rsob140029supp3.zip › rsob-14-0029-File010/Melanoma/ENSG00000070718_ENSG00000185009.png]

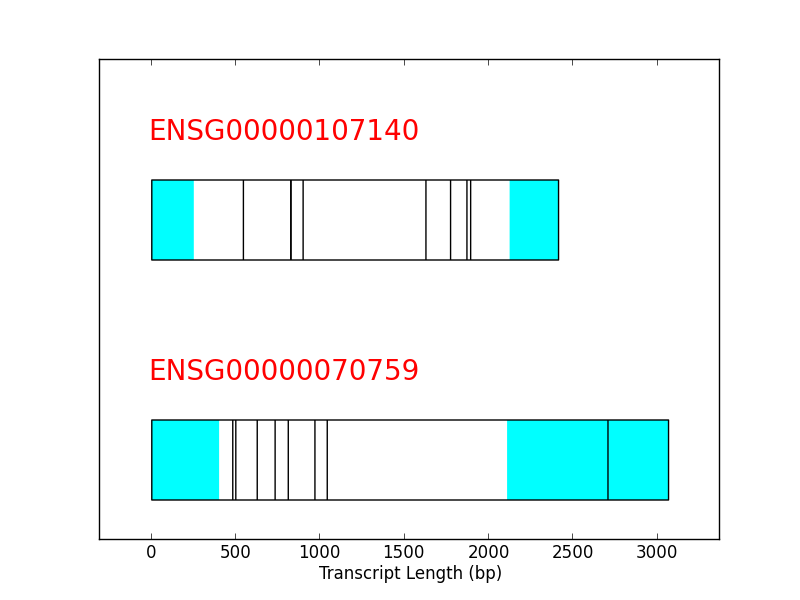

Supplement: Data file S2 [file rsob140029supp3.zip › rsob-14-0029-File010/Melanoma/ENSG00000070759_ENSG00000107140.png]

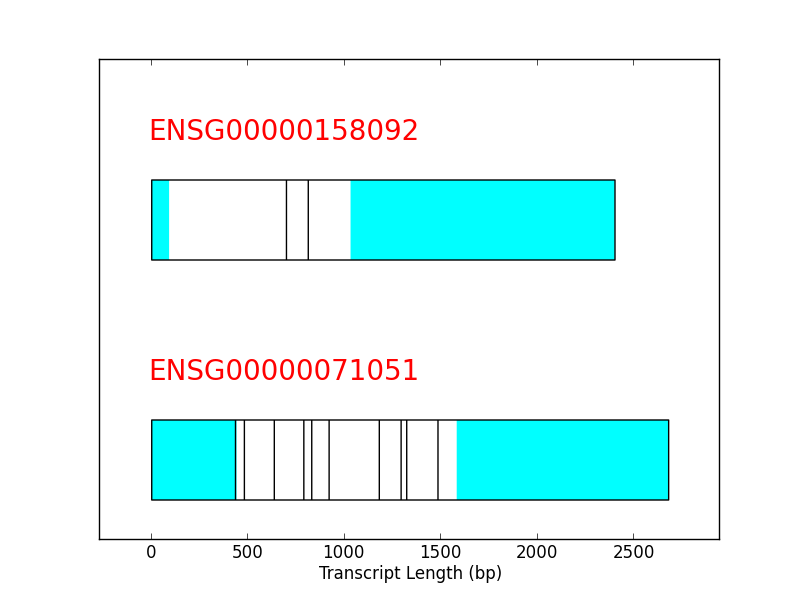

Supplement: Data file S2 [file rsob140029supp3.zip › rsob-14-0029-File010/Melanoma/ENSG00000071051_ENSG00000158092.png]

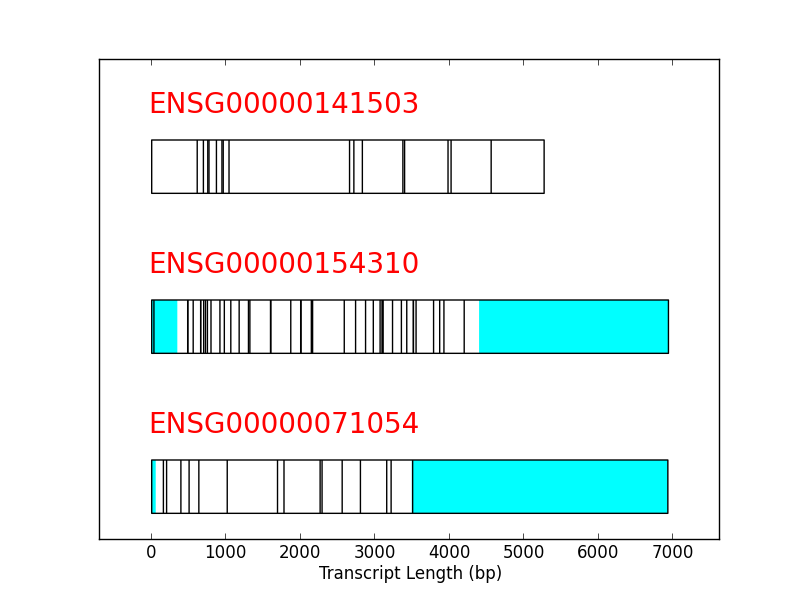

Supplement: Data file S2 [file rsob140029supp3.zip › rsob-14-0029-File010/Melanoma/ENSG00000071054_ENSG00000154310_ENSG00000141503.png]

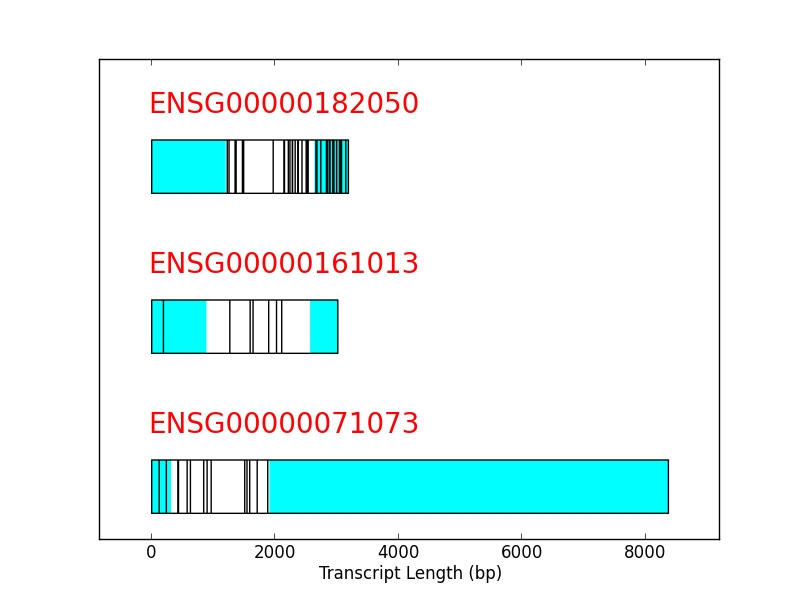

Supplement: Data file S2 [file rsob140029supp3.zip › rsob-14-0029-File010/Melanoma/ENSG00000071073_ENSG00000161013_ENSG00000182050.png]

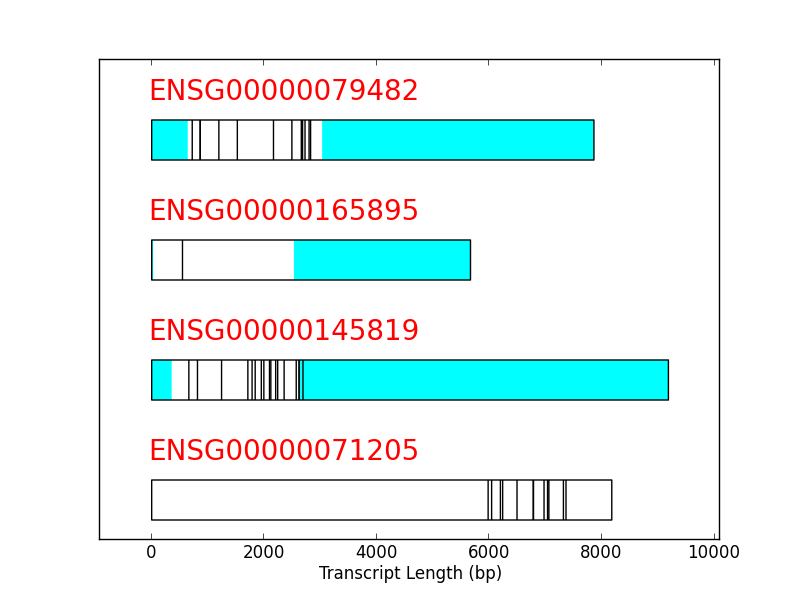

Supplement: Data file S2 [file rsob140029supp3.zip › rsob-14-0029-File010/Melanoma/ENSG00000071205_ENSG00000145819_ENSG00000165895_ENSG00000079482.png]

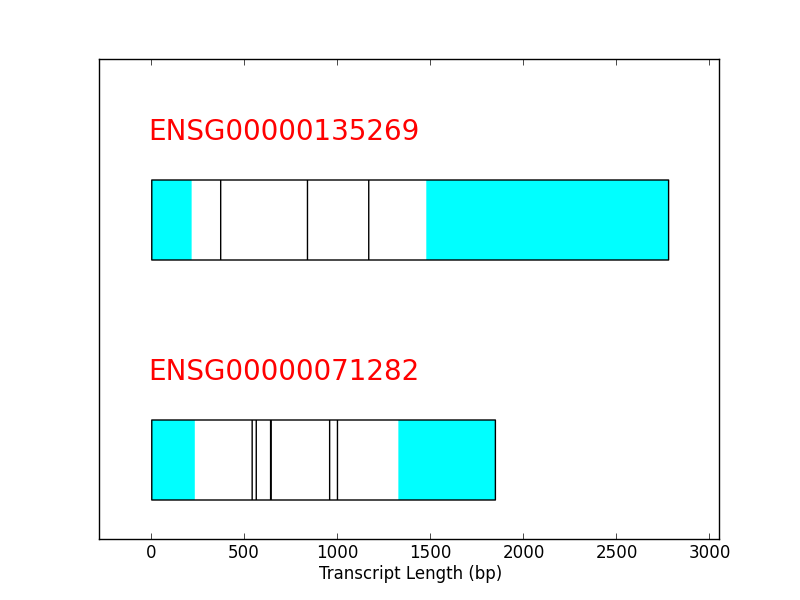

Supplement: Data file S2 [file rsob140029supp3.zip › rsob-14-0029-File010/Melanoma/ENSG00000071282_ENSG00000135269.png]

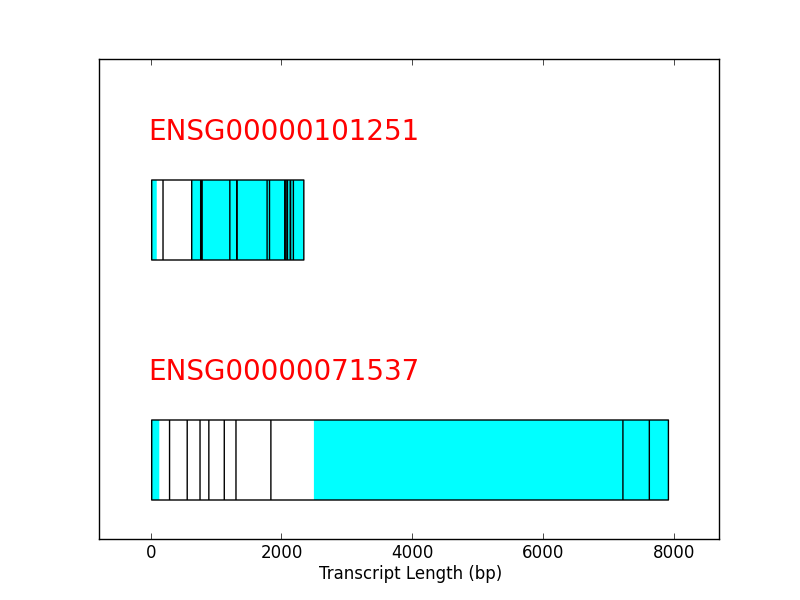

Supplement: Data file S2 [file rsob140029supp3.zip › rsob-14-0029-File010/Melanoma/ENSG00000071537_ENSG00000101251.png]

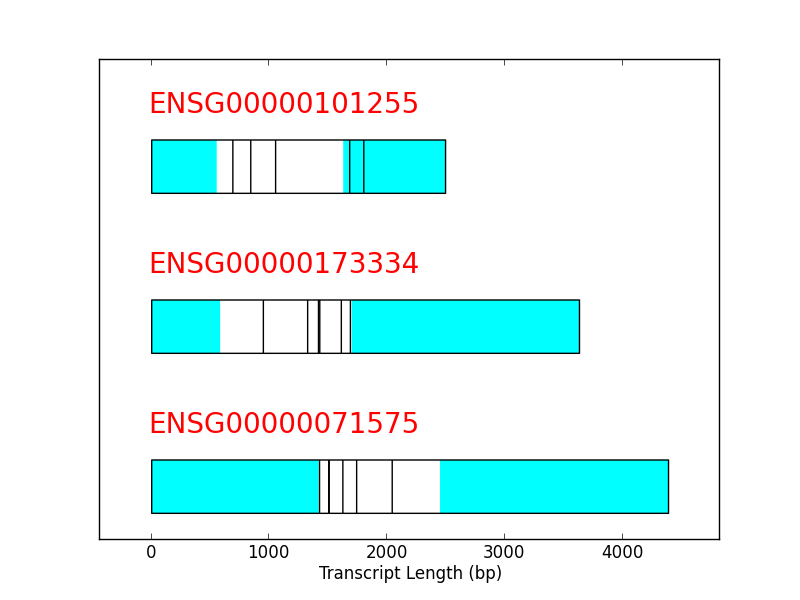

Supplement: Data file S2 [file rsob140029supp3.zip › rsob-14-0029-File010/Melanoma/ENSG00000071575_ENSG00000173334_ENSG00000101255.png]

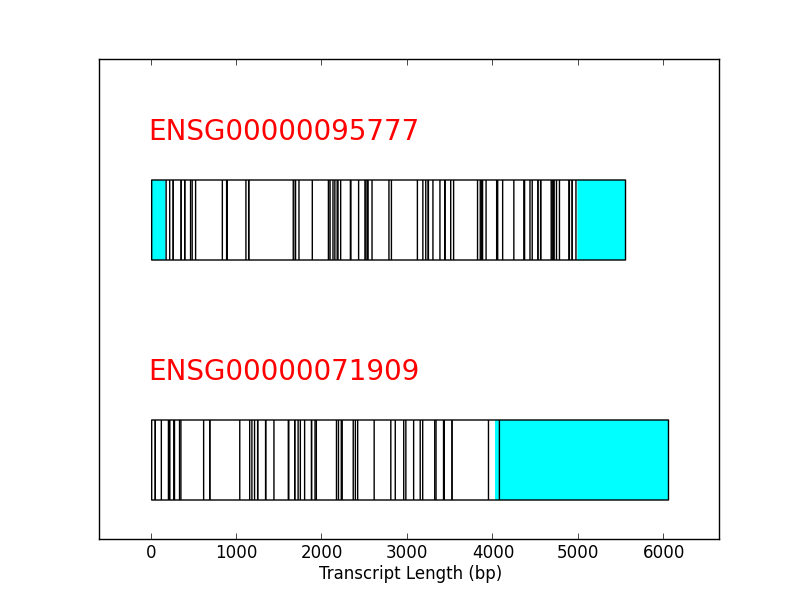

Supplement: Data file S2 [file rsob140029supp3.zip › rsob-14-0029-File010/Melanoma/ENSG00000071909_ENSG00000095777.png]

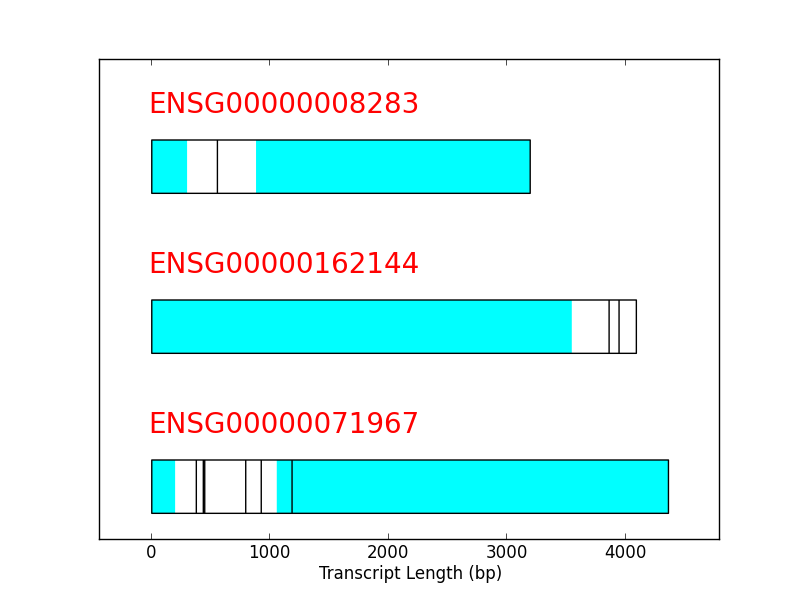

Supplement: Data file S2 [file rsob140029supp3.zip › rsob-14-0029-File010/Melanoma/ENSG00000071967_ENSG00000162144_ENSG00000008283.png]

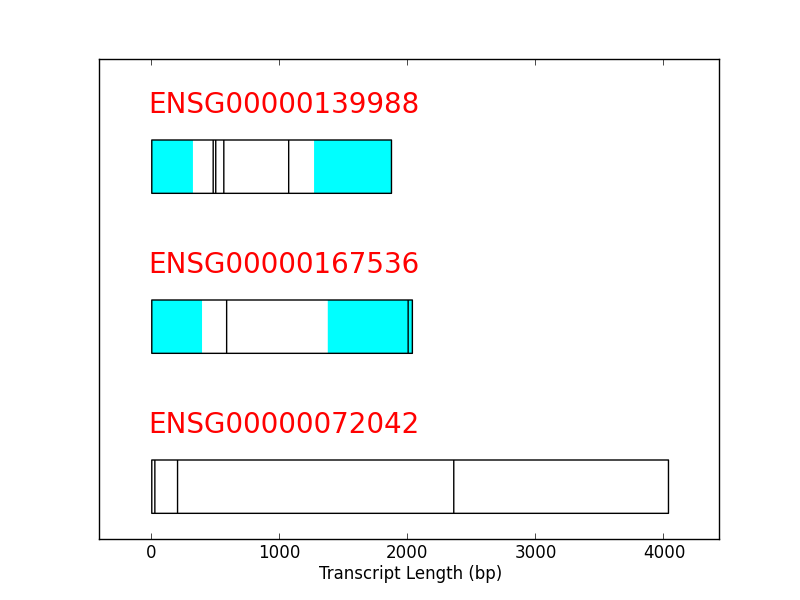

Supplement: Data file S2 [file rsob140029supp3.zip › rsob-14-0029-File010/Melanoma/ENSG00000072042_ENSG00000167536_ENSG00000139988.png]

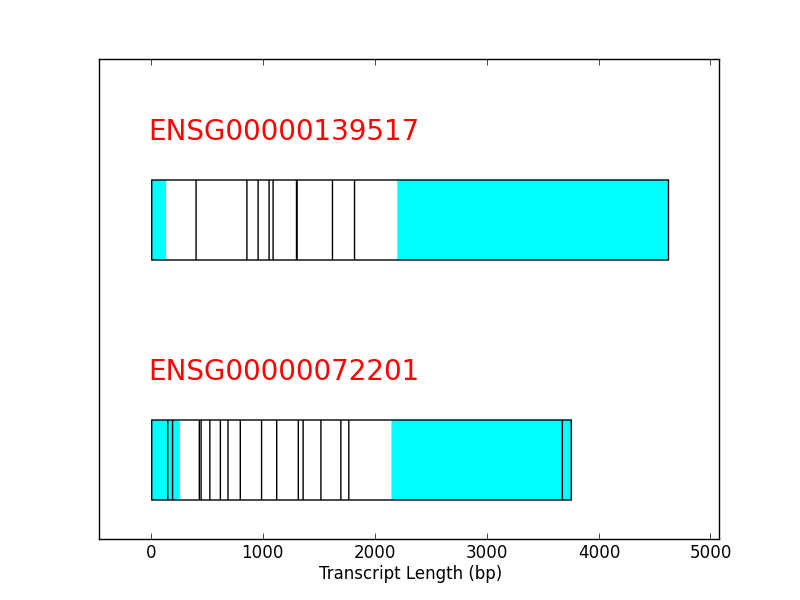

Supplement: Data file S2 [file rsob140029supp3.zip › rsob-14-0029-File010/Melanoma/ENSG00000072201_ENSG00000139517.png]

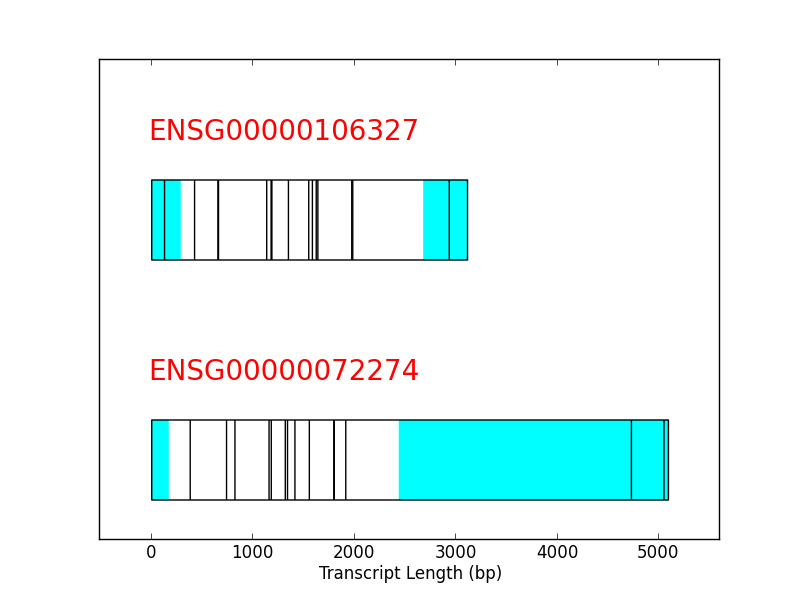

Supplement: Data file S2 [file rsob140029supp3.zip › rsob-14-0029-File010/Melanoma/ENSG00000072274_ENSG00000106327.png]

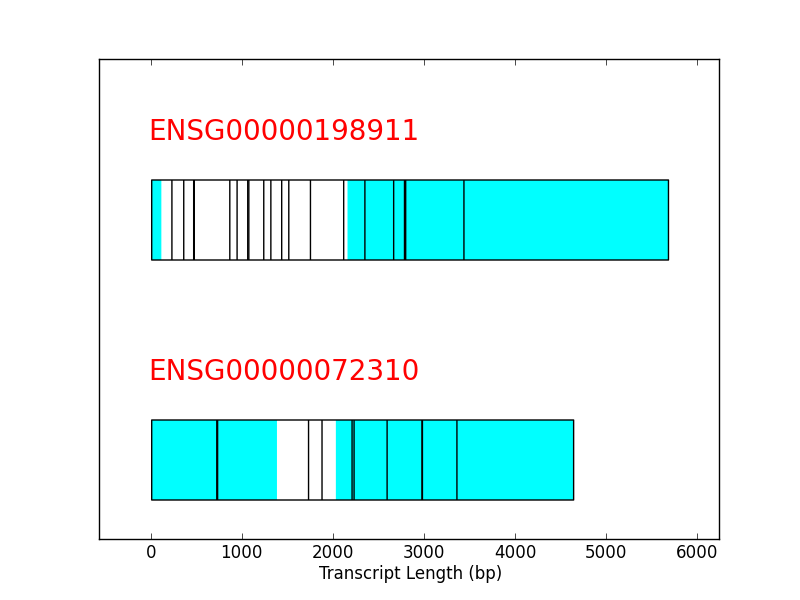

Supplement: Data file S2 [file rsob140029supp3.zip › rsob-14-0029-File010/Melanoma/ENSG00000072310_ENSG00000198911.png]

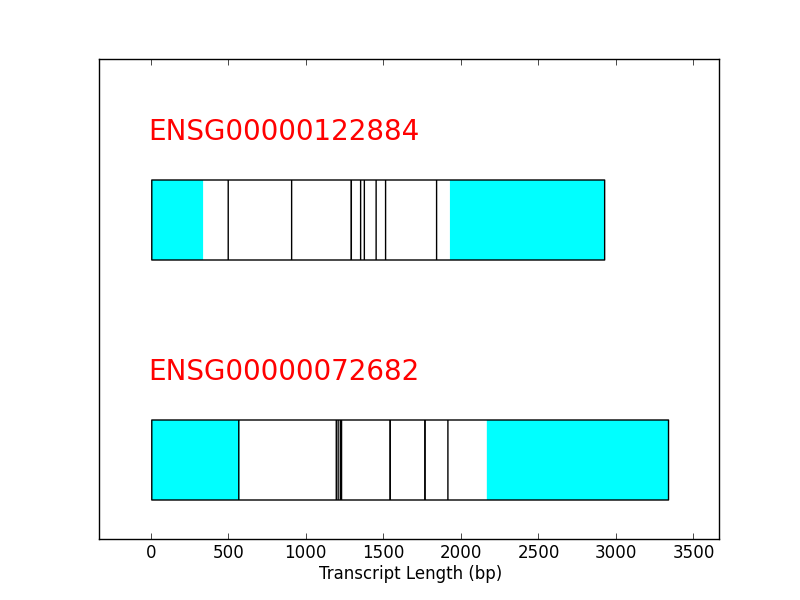

Supplement: Data file S2 [file rsob140029supp3.zip › rsob-14-0029-File010/Melanoma/ENSG00000072682_ENSG00000122884.png]

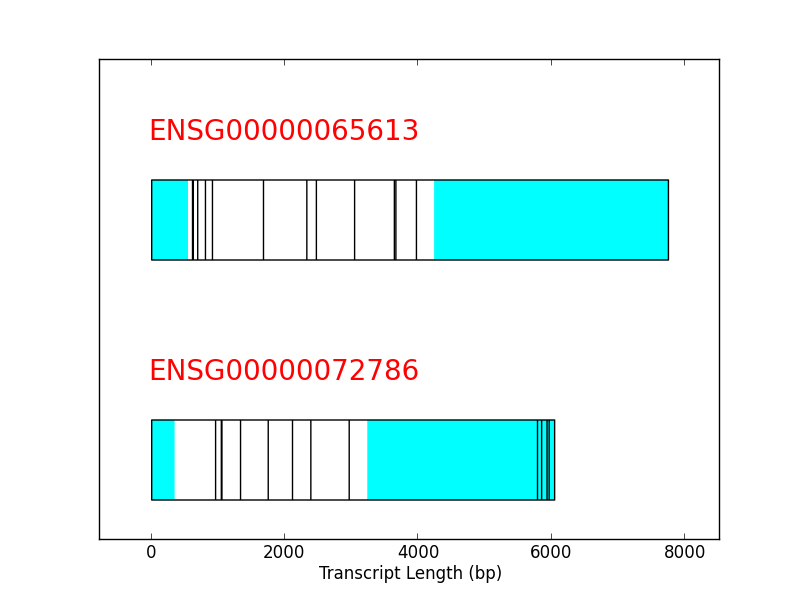

Supplement: Data file S2 [file rsob140029supp3.zip › rsob-14-0029-File010/Melanoma/ENSG00000072786_ENSG00000065613.png]

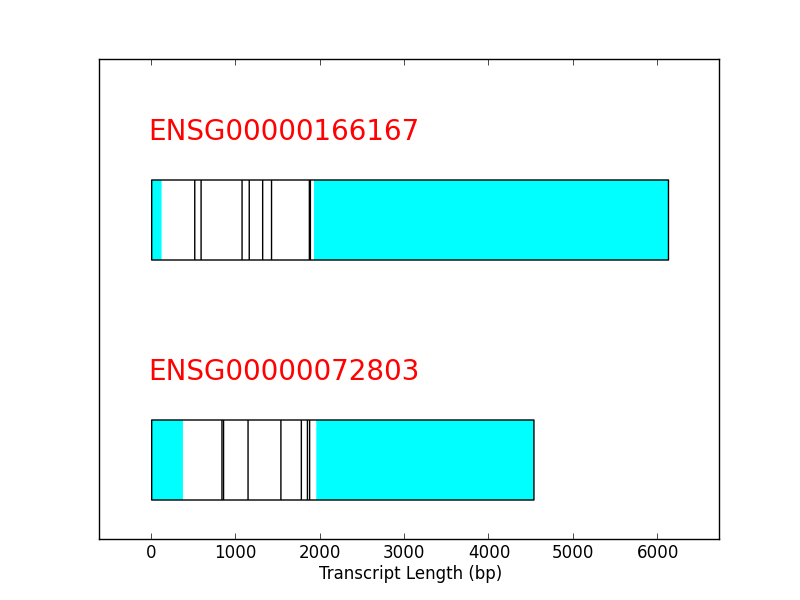

Supplement: Data file S2 [file rsob140029supp3.zip › rsob-14-0029-File010/Melanoma/ENSG00000072803_ENSG00000166167.png]

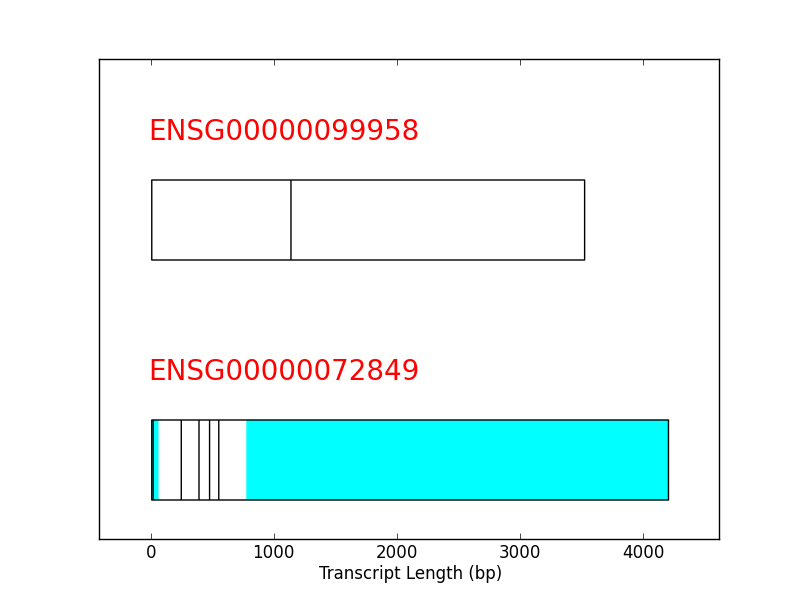

Supplement: Data file S2 [file rsob140029supp3.zip › rsob-14-0029-File010/Melanoma/ENSG00000072849_ENSG00000099958.png]

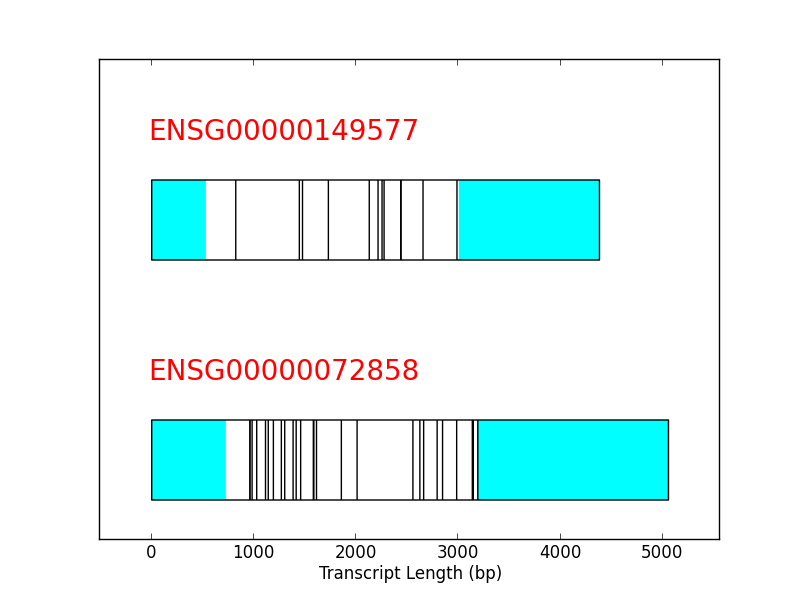

Supplement: Data file S2 [file rsob140029supp3.zip › rsob-14-0029-File010/Melanoma/ENSG00000072858_ENSG00000149577.png]

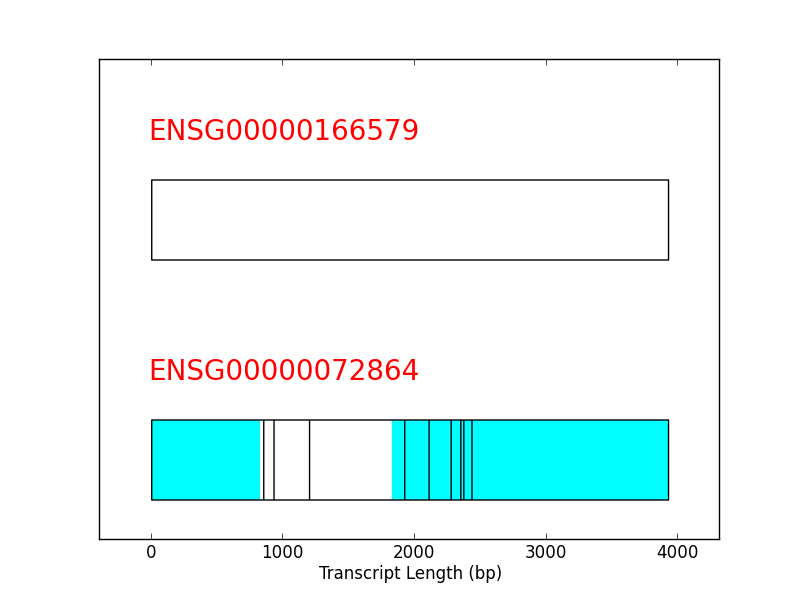

Supplement: Data file S2 [file rsob140029supp3.zip › rsob-14-0029-File010/Melanoma/ENSG00000072864_ENSG00000166579.png]

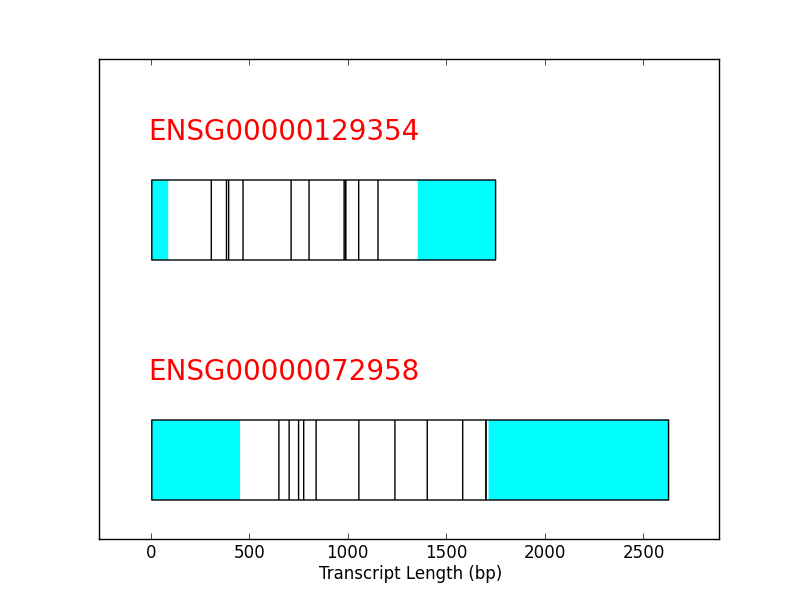

Supplement: Data file S2 [file rsob140029supp3.zip › rsob-14-0029-File010/Melanoma/ENSG00000072958_ENSG00000129354.png]

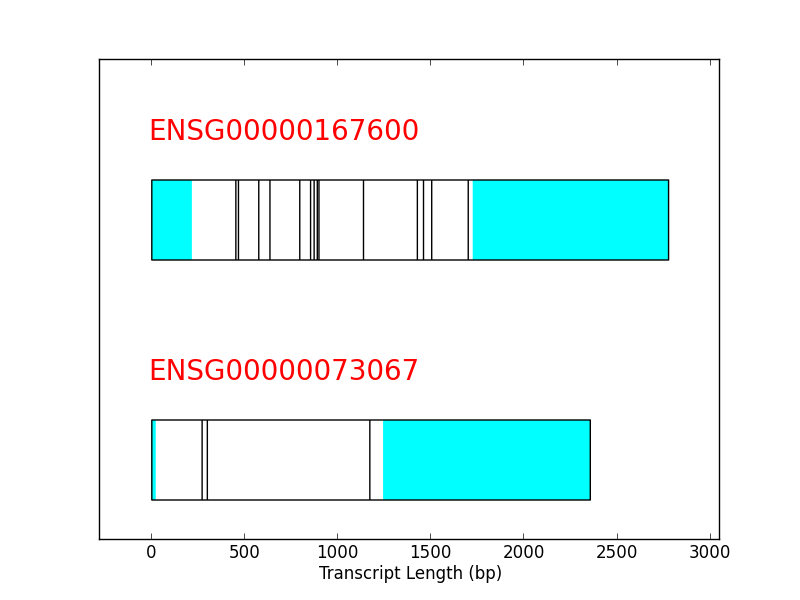

Supplement: Data file S2 [file rsob140029supp3.zip › rsob-14-0029-File010/Melanoma/ENSG00000073067_ENSG00000167600.png]
