# Supplementary figures and images for: Identification of 2R-ohnologue gene families displaying the same mutation-load skew in multiple cancers (part 2 of 3)
Source: Open Biol. 2014 May 7;4(5):140029. doi: 10.1098/rsob.140029 (PMC4042849; doi:10.1098/rsob.140029)

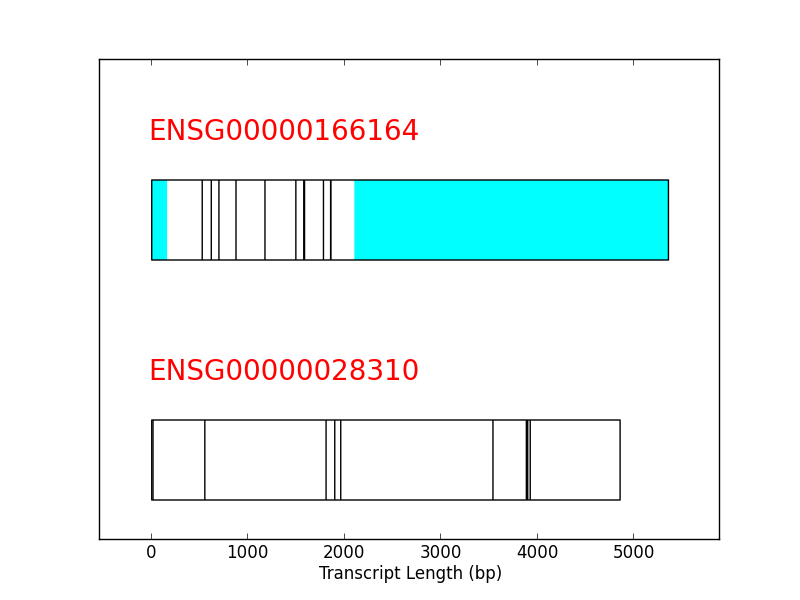

Supplement: Data file S2 [file rsob140029supp3.zip › rsob-14-0029-File010/Melanoma/ENSG00000028310_ENSG00000166164.png]

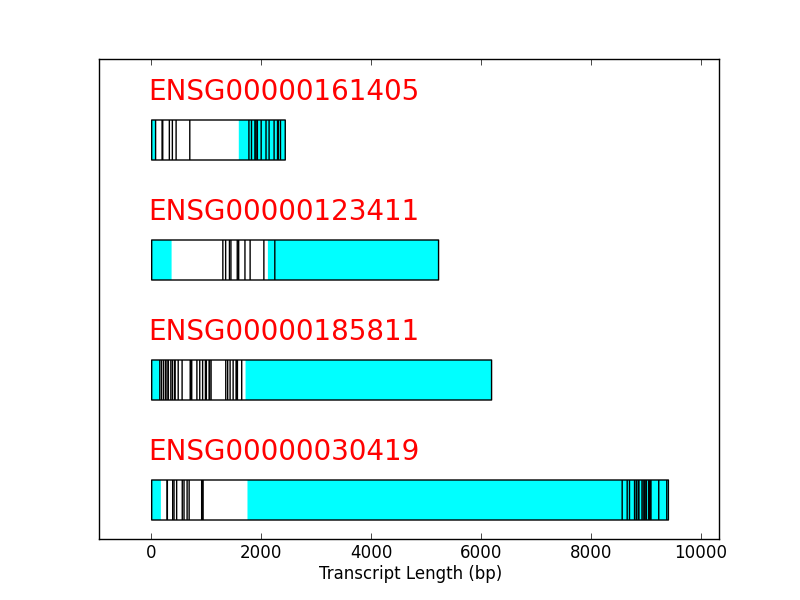

Supplement: Data file S2 [file rsob140029supp3.zip › rsob-14-0029-File010/Melanoma/ENSG00000030419_ENSG00000185811_ENSG00000123411_ENSG00000161405.png]

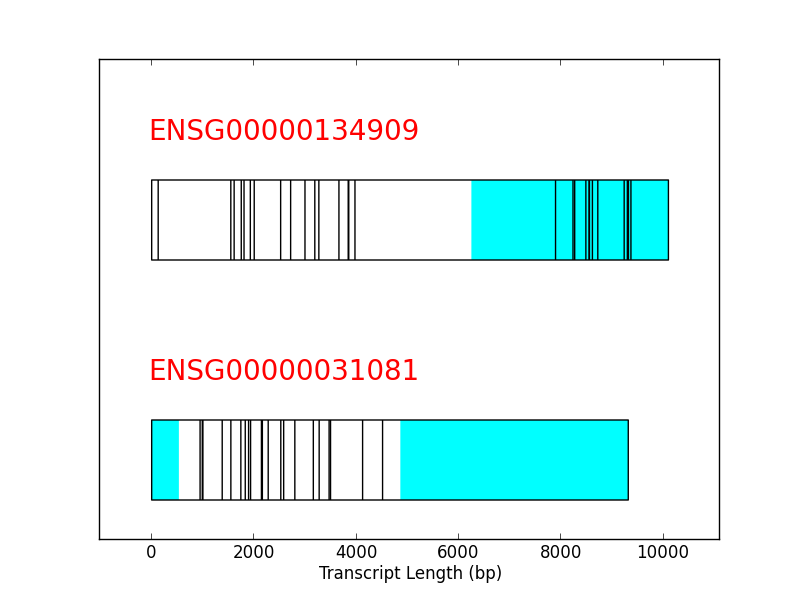

Supplement: Data file S2 [file rsob140029supp3.zip › rsob-14-0029-File010/Melanoma/ENSG00000031081_ENSG00000134909.png]

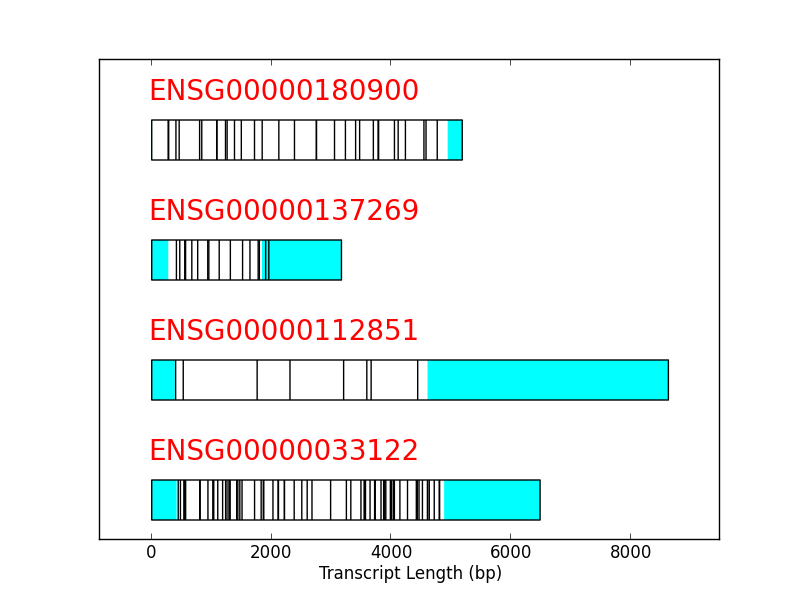

Supplement: Data file S2 [file rsob140029supp3.zip › rsob-14-0029-File010/Melanoma/ENSG00000033122_ENSG00000112851_ENSG00000137269_ENSG00000180900.png]

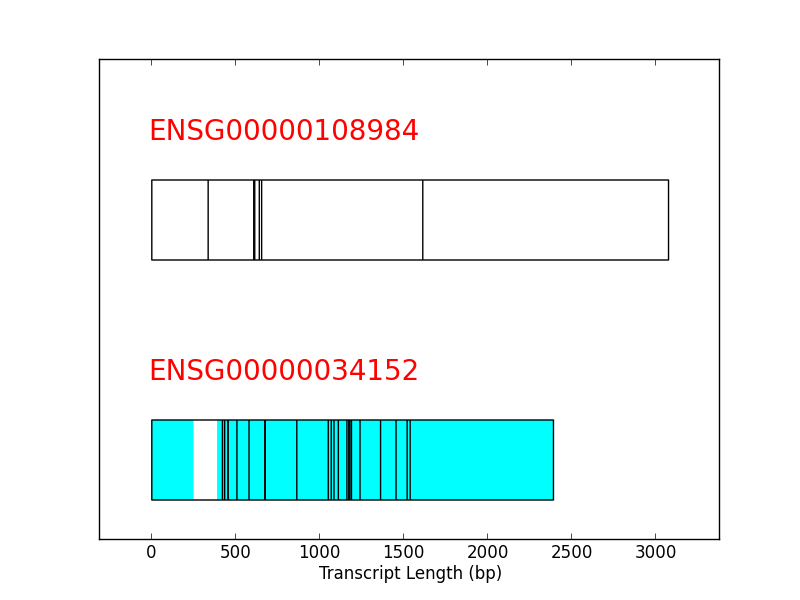

Supplement: Data file S2 [file rsob140029supp3.zip › rsob-14-0029-File010/Melanoma/ENSG00000034152_ENSG00000108984.png]

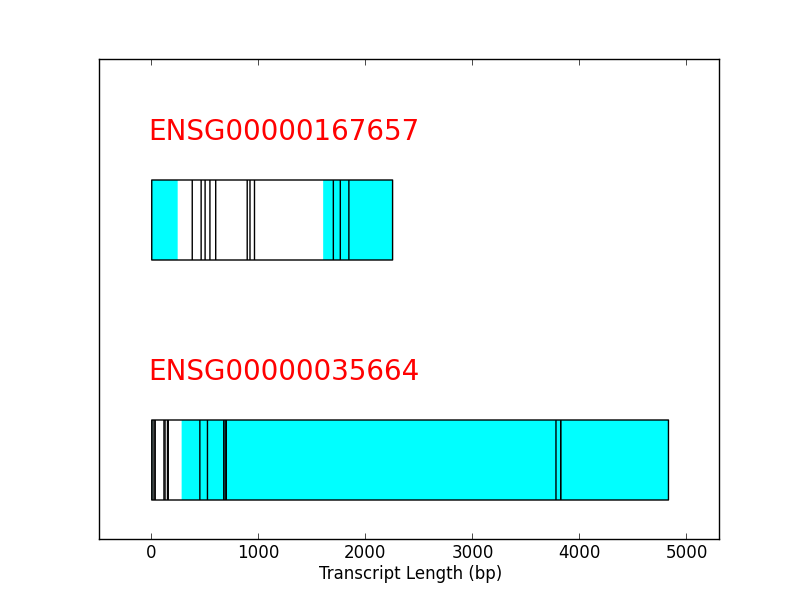

Supplement: Data file S2 [file rsob140029supp3.zip › rsob-14-0029-File010/Melanoma/ENSG00000035664_ENSG00000167657.png]

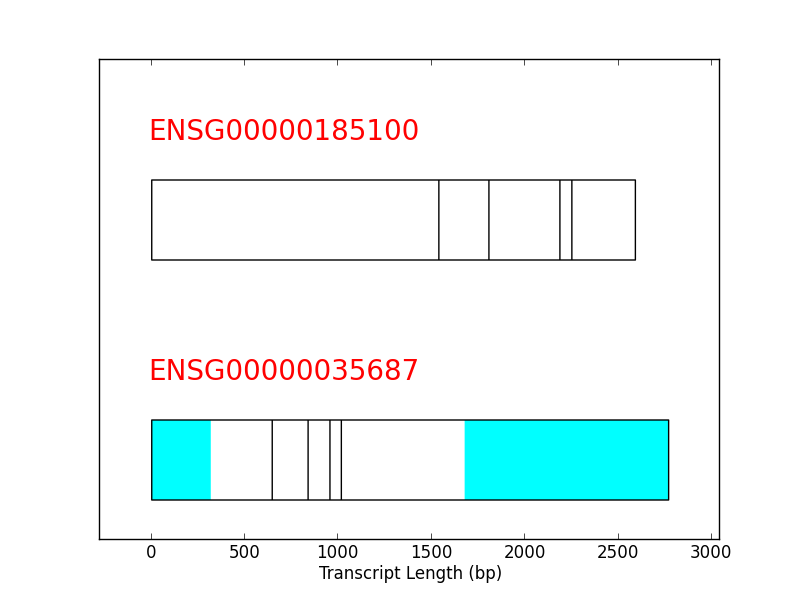

Supplement: Data file S2 [file rsob140029supp3.zip › rsob-14-0029-File010/Melanoma/ENSG00000035687_ENSG00000185100.png]

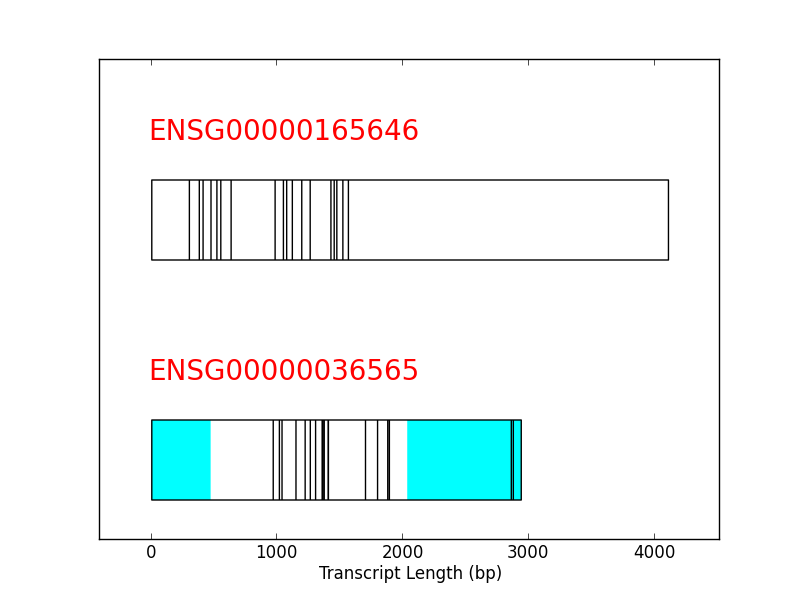

Supplement: Data file S2 [file rsob140029supp3.zip › rsob-14-0029-File010/Melanoma/ENSG00000036565_ENSG00000165646.png]

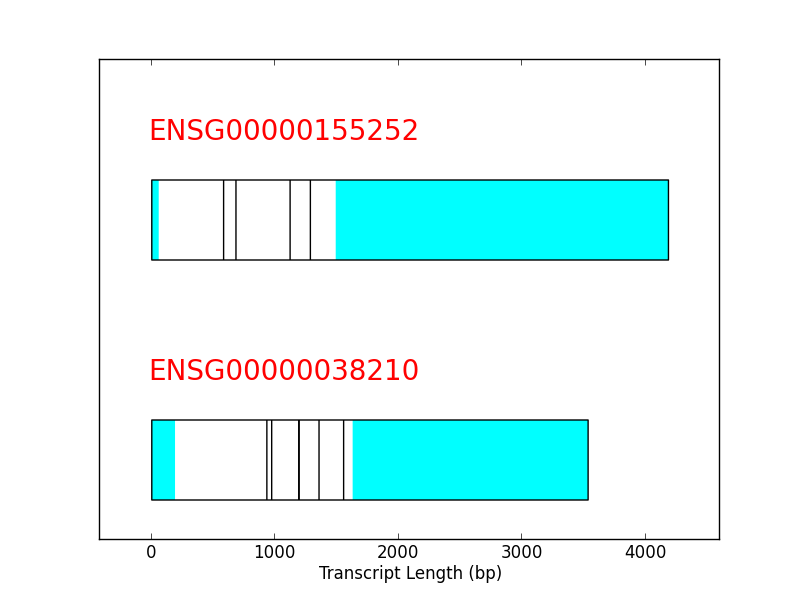

Supplement: Data file S2 [file rsob140029supp3.zip › rsob-14-0029-File010/Melanoma/ENSG00000038210_ENSG00000155252.png]

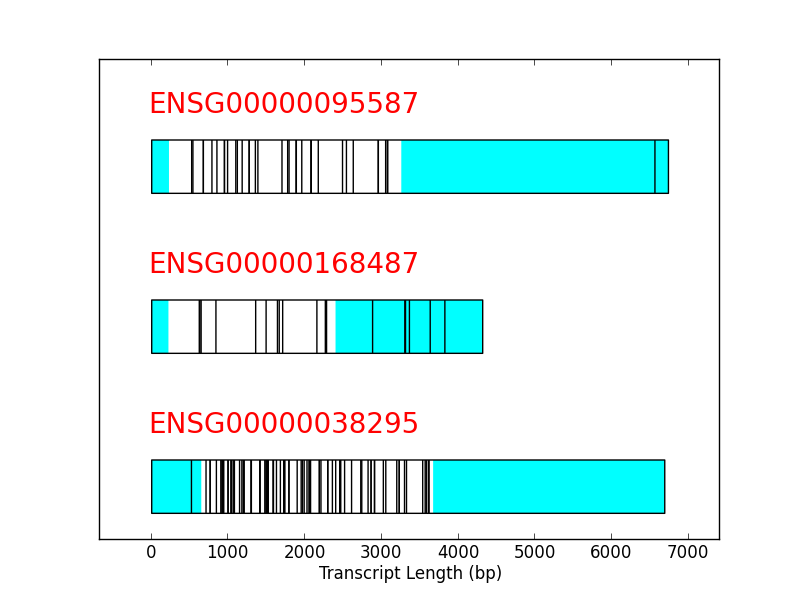

Supplement: Data file S2 [file rsob140029supp3.zip › rsob-14-0029-File010/Melanoma/ENSG00000038295_ENSG00000168487_ENSG00000095587.png]

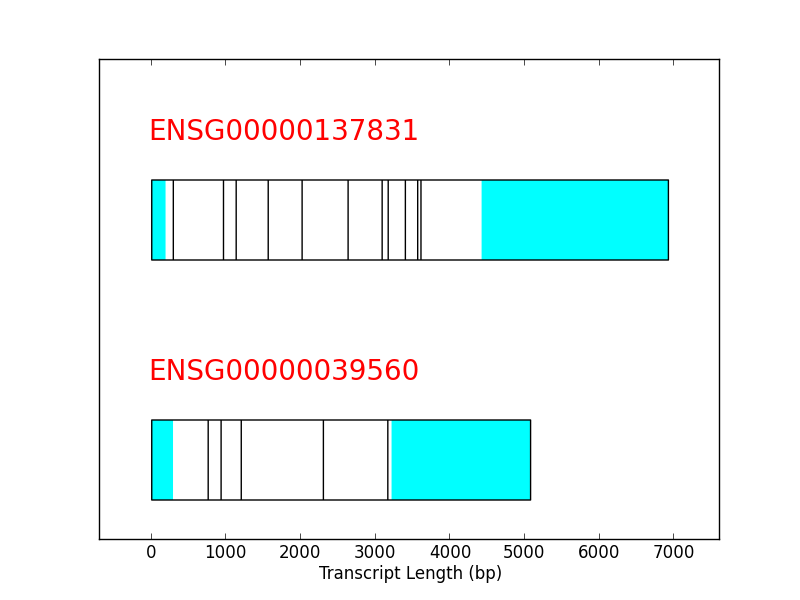

Supplement: Data file S2 [file rsob140029supp3.zip › rsob-14-0029-File010/Melanoma/ENSG00000039560_ENSG00000137831.png]

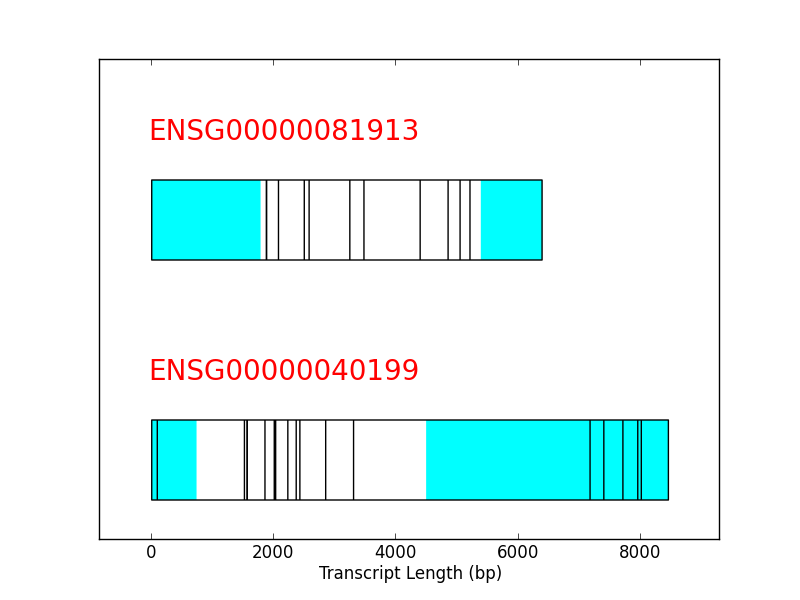

Supplement: Data file S2 [file rsob140029supp3.zip › rsob-14-0029-File010/Melanoma/ENSG00000040199_ENSG00000081913.png]

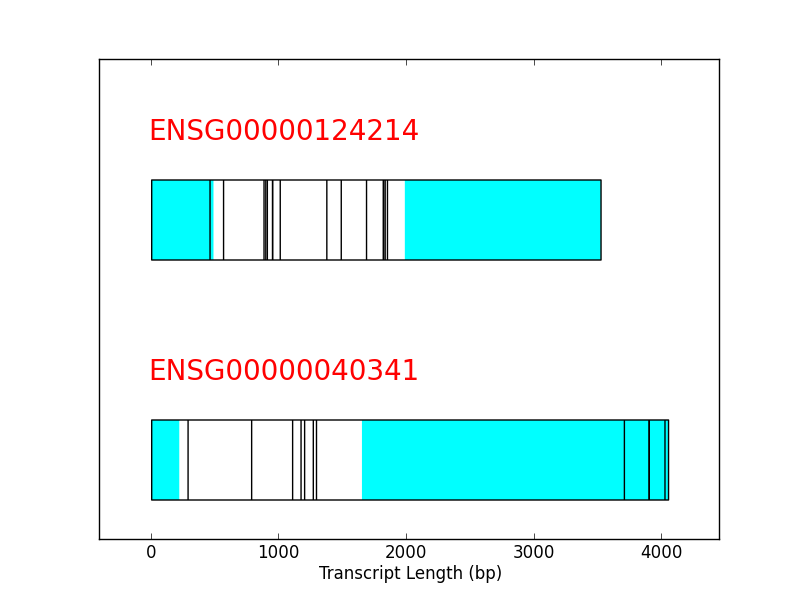

Supplement: Data file S2 [file rsob140029supp3.zip › rsob-14-0029-File010/Melanoma/ENSG00000040341_ENSG00000124214.png]

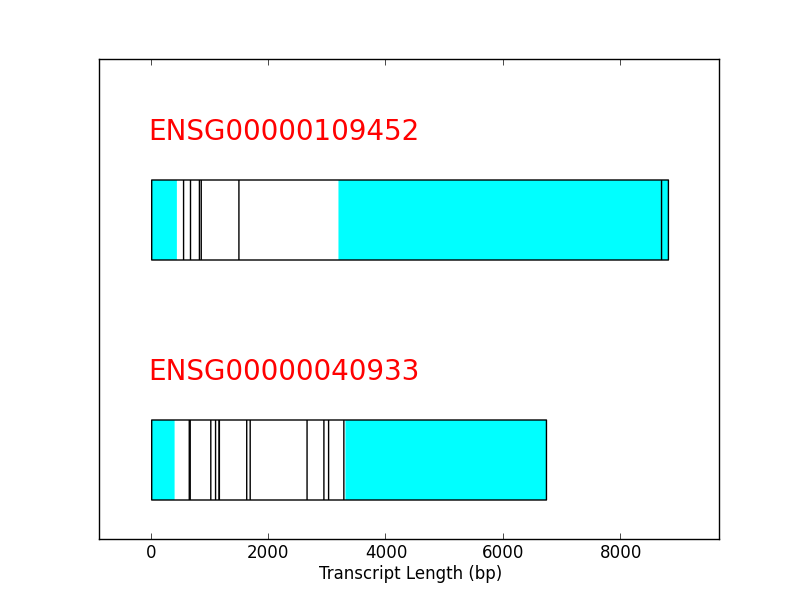

Supplement: Data file S2 [file rsob140029supp3.zip › rsob-14-0029-File010/Melanoma/ENSG00000040933_ENSG00000109452.png]

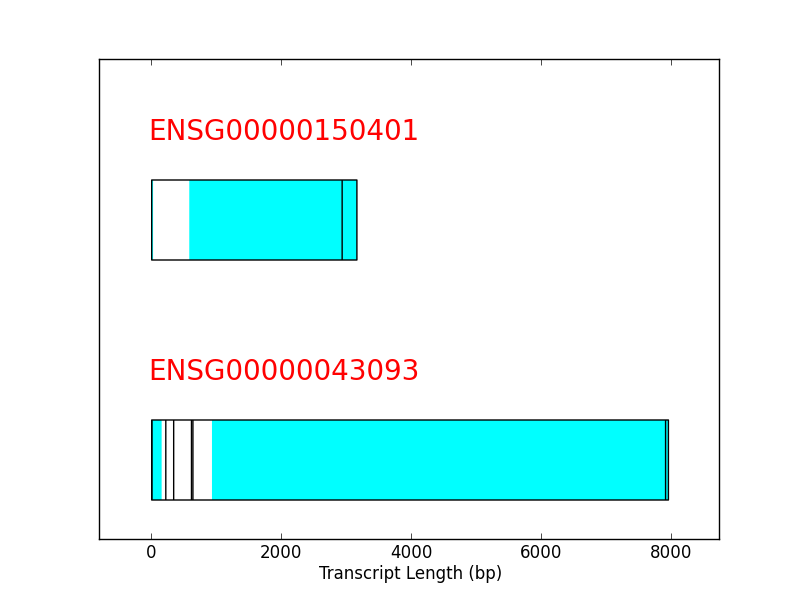

Supplement: Data file S2 [file rsob140029supp3.zip › rsob-14-0029-File010/Melanoma/ENSG00000043093_ENSG00000150401.png]

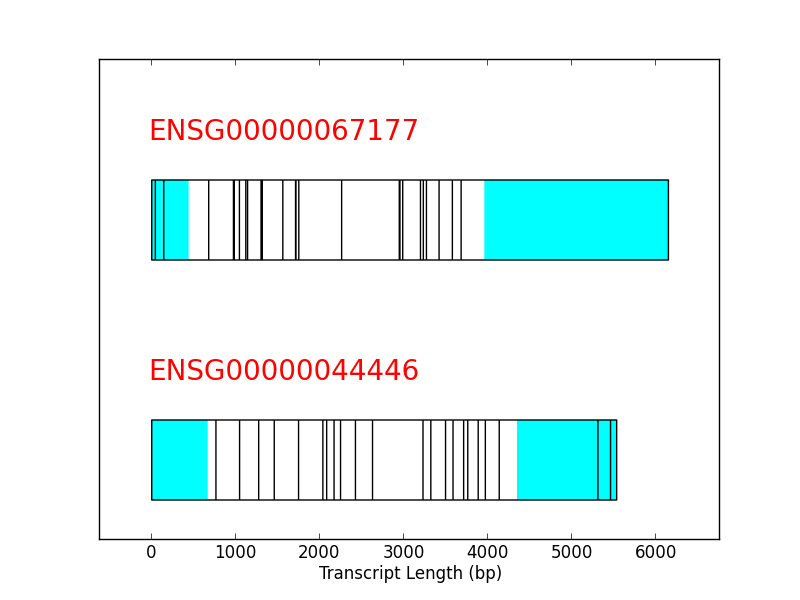

Supplement: Data file S2 [file rsob140029supp3.zip › rsob-14-0029-File010/Melanoma/ENSG00000044446_ENSG00000067177.png]

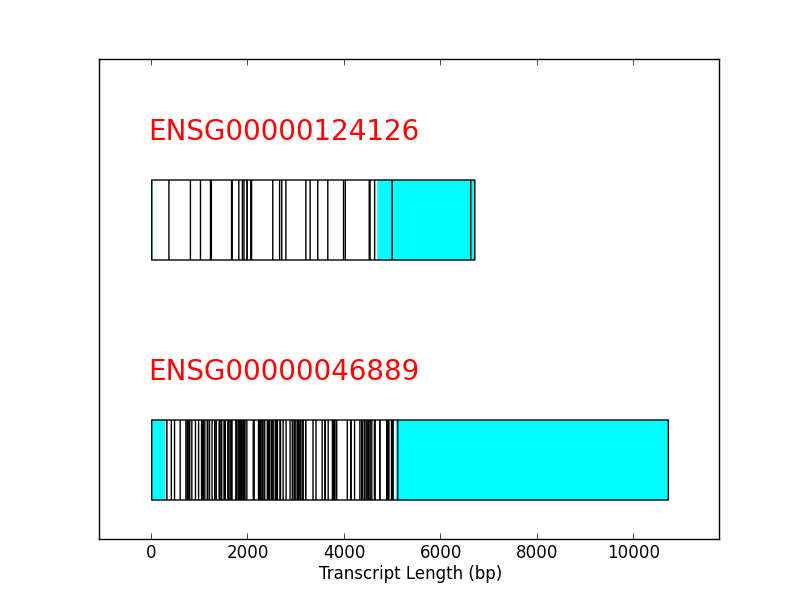

Supplement: Data file S2 [file rsob140029supp3.zip › rsob-14-0029-File010/Melanoma/ENSG00000046889_ENSG00000124126.png]

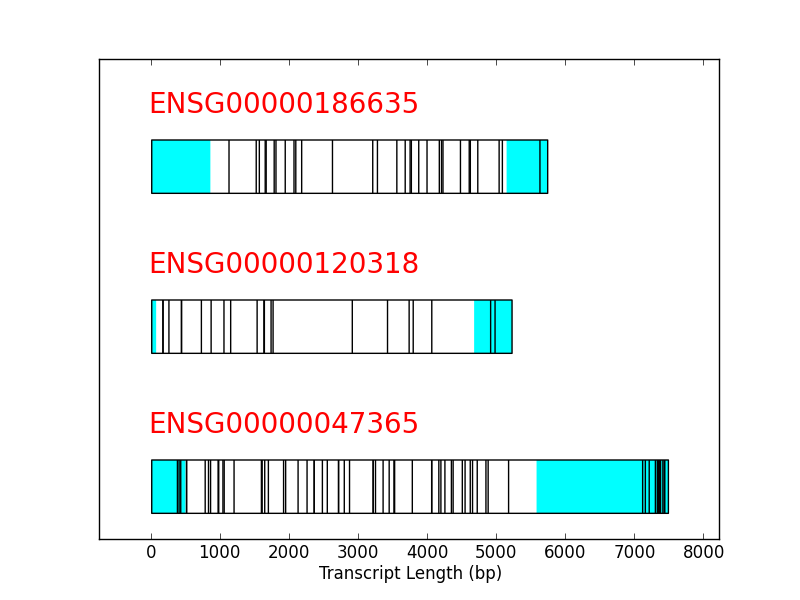

Supplement: Data file S2 [file rsob140029supp3.zip › rsob-14-0029-File010/Melanoma/ENSG00000047365_ENSG00000120318_ENSG00000186635.png]

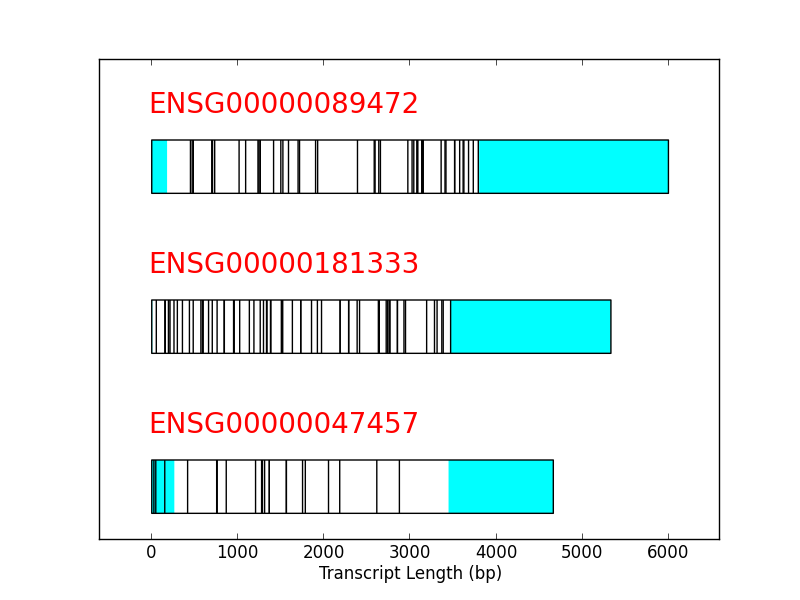

Supplement: Data file S2 [file rsob140029supp3.zip › rsob-14-0029-File010/Melanoma/ENSG00000047457_ENSG00000181333_ENSG00000089472.png]

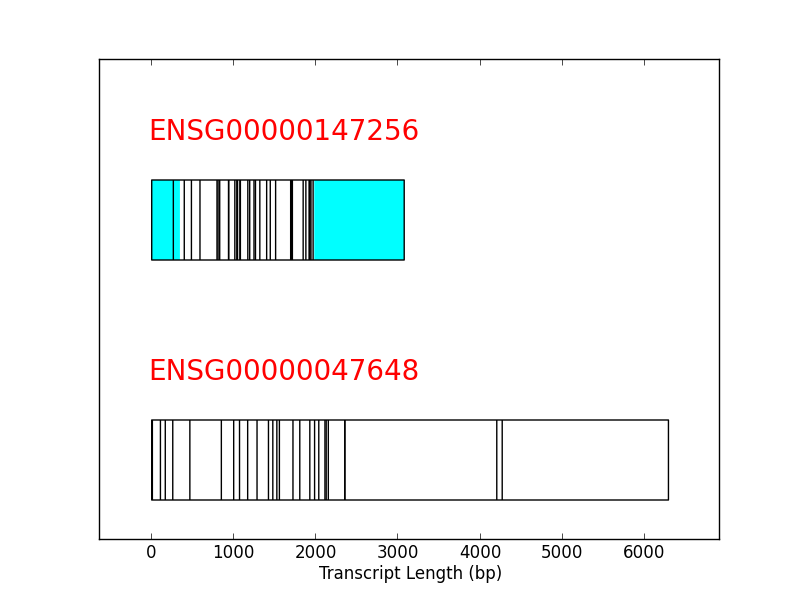

Supplement: Data file S2 [file rsob140029supp3.zip › rsob-14-0029-File010/Melanoma/ENSG00000047648_ENSG00000147256.png]

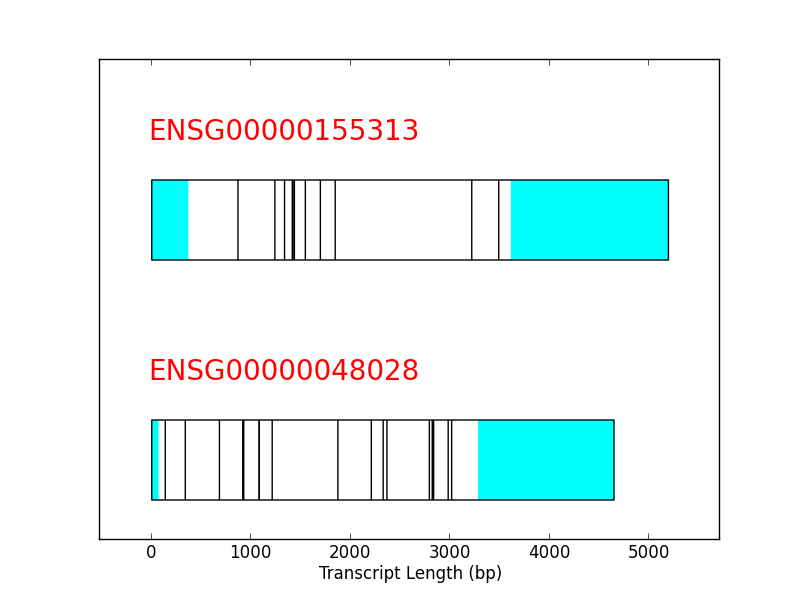

Supplement: Data file S2 [file rsob140029supp3.zip › rsob-14-0029-File010/Melanoma/ENSG00000048028_ENSG00000155313.png]

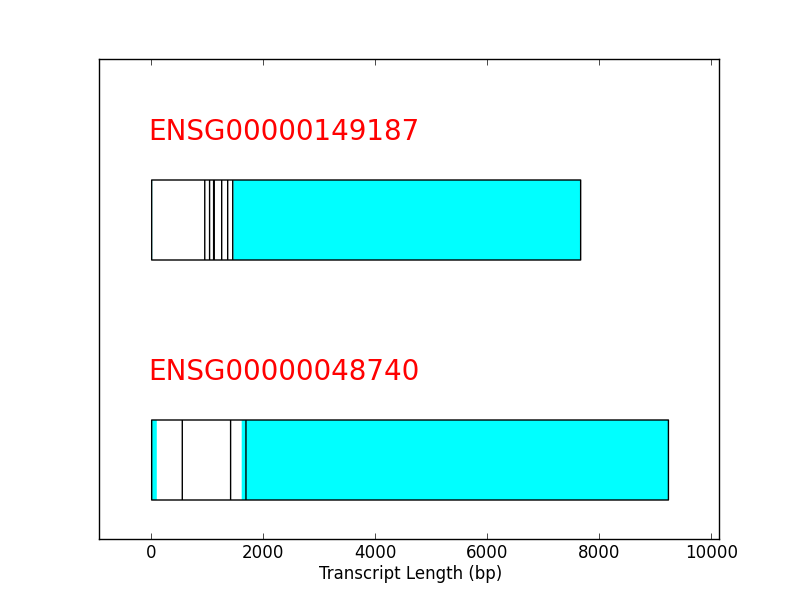

Supplement: Data file S2 [file rsob140029supp3.zip › rsob-14-0029-File010/Melanoma/ENSG00000048740_ENSG00000149187.png]

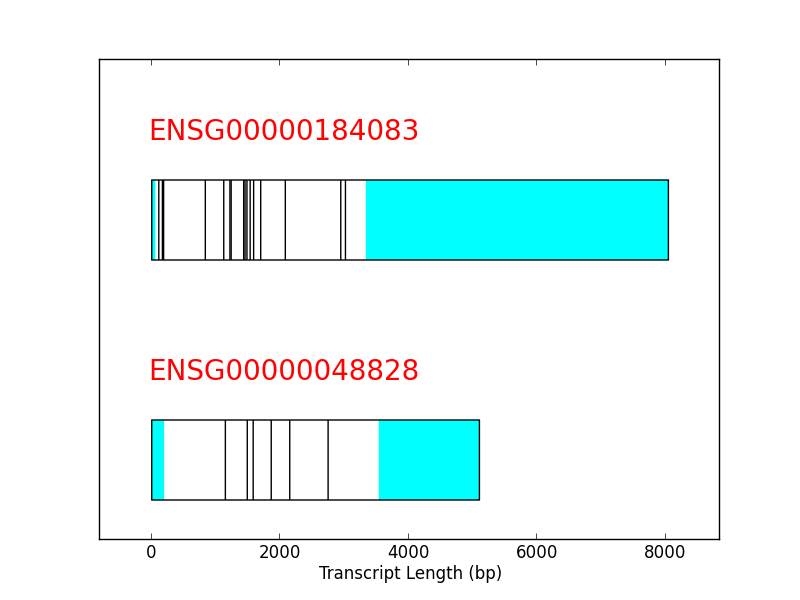

Supplement: Data file S2 [file rsob140029supp3.zip › rsob-14-0029-File010/Melanoma/ENSG00000048828_ENSG00000184083.png]

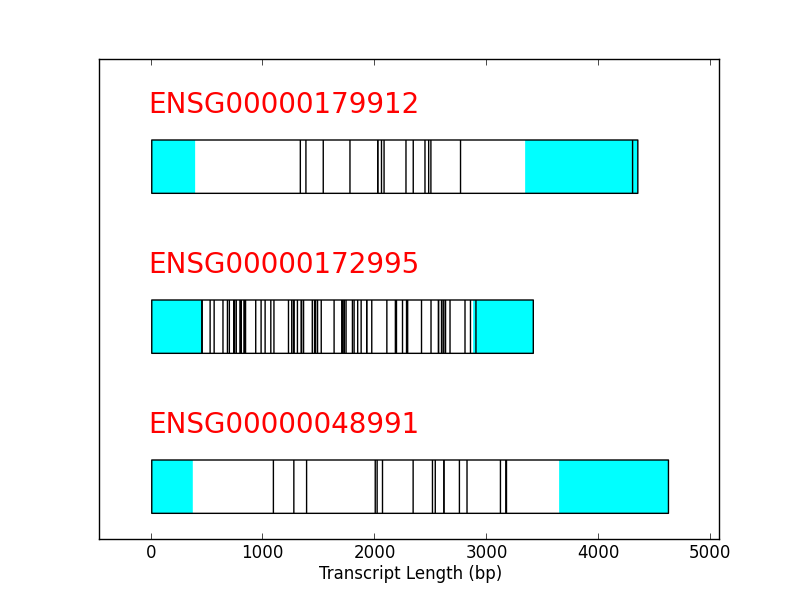

Supplement: Data file S2 [file rsob140029supp3.zip › rsob-14-0029-File010/Melanoma/ENSG00000048991_ENSG00000172995_ENSG00000179912.png]

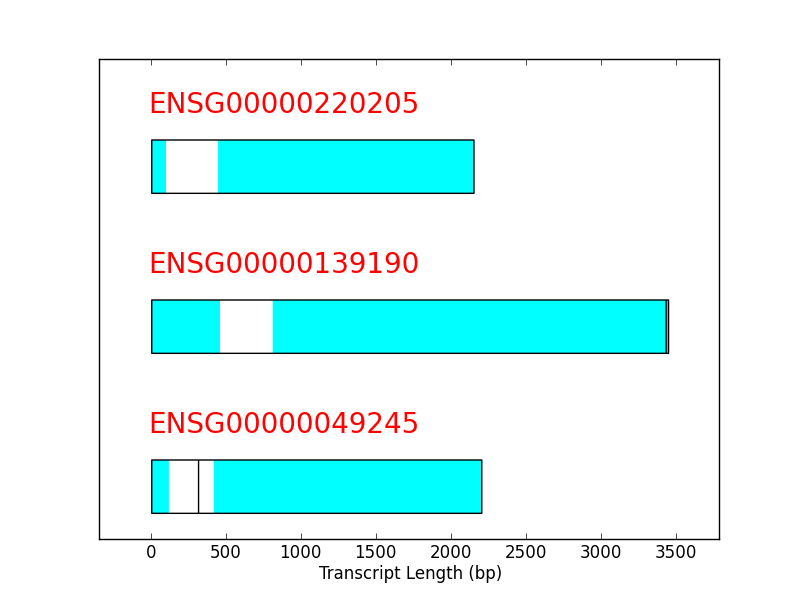

Supplement: Data file S2 [file rsob140029supp3.zip › rsob-14-0029-File010/Melanoma/ENSG00000049245_ENSG00000139190_ENSG00000220205.png]

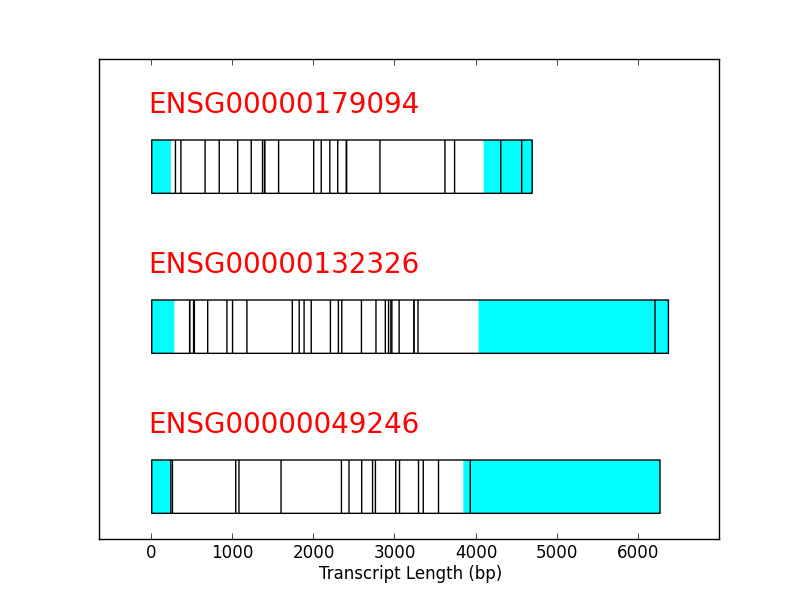

Supplement: Data file S2 [file rsob140029supp3.zip › rsob-14-0029-File010/Melanoma/ENSG00000049246_ENSG00000132326_ENSG00000179094.png]

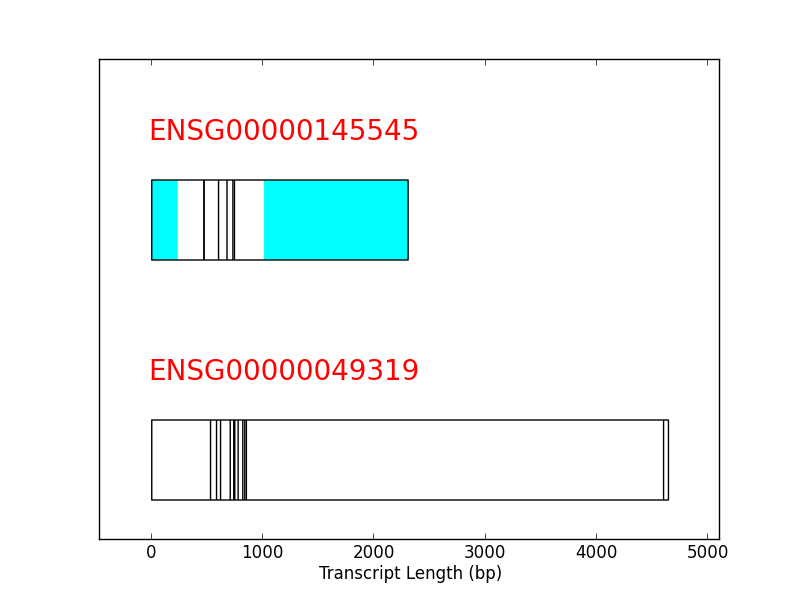

Supplement: Data file S2 [file rsob140029supp3.zip › rsob-14-0029-File010/Melanoma/ENSG00000049319_ENSG00000145545.png]

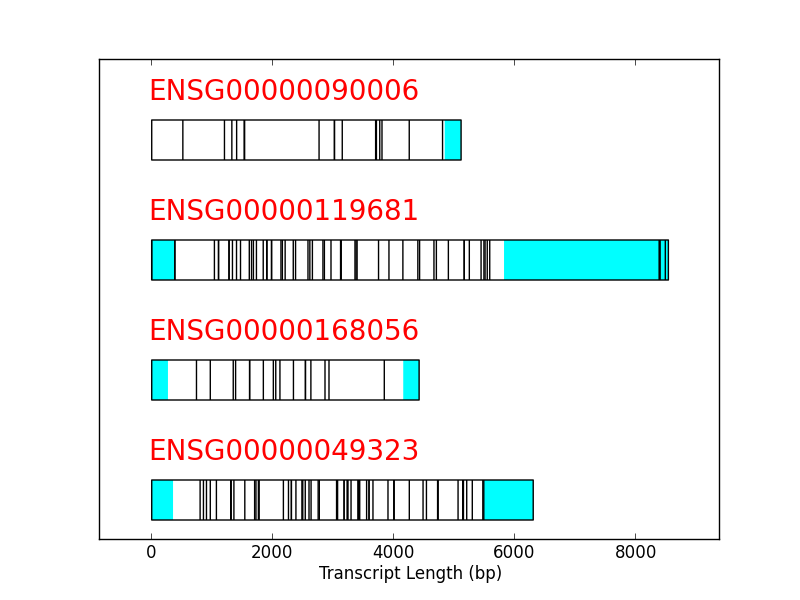

Supplement: Data file S2 [file rsob140029supp3.zip › rsob-14-0029-File010/Melanoma/ENSG00000049323_ENSG00000168056_ENSG00000119681_ENSG00000090006.png]

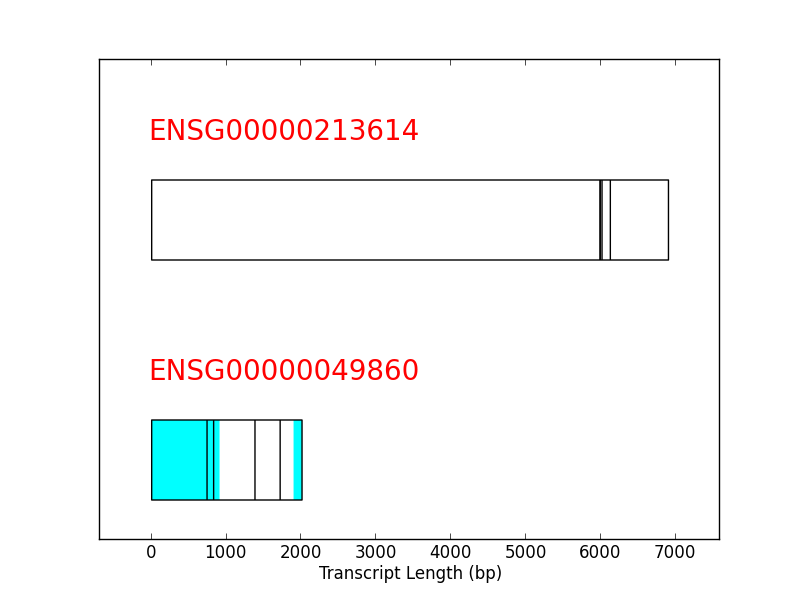

Supplement: Data file S2 [file rsob140029supp3.zip › rsob-14-0029-File010/Melanoma/ENSG00000049860_ENSG00000213614.png]

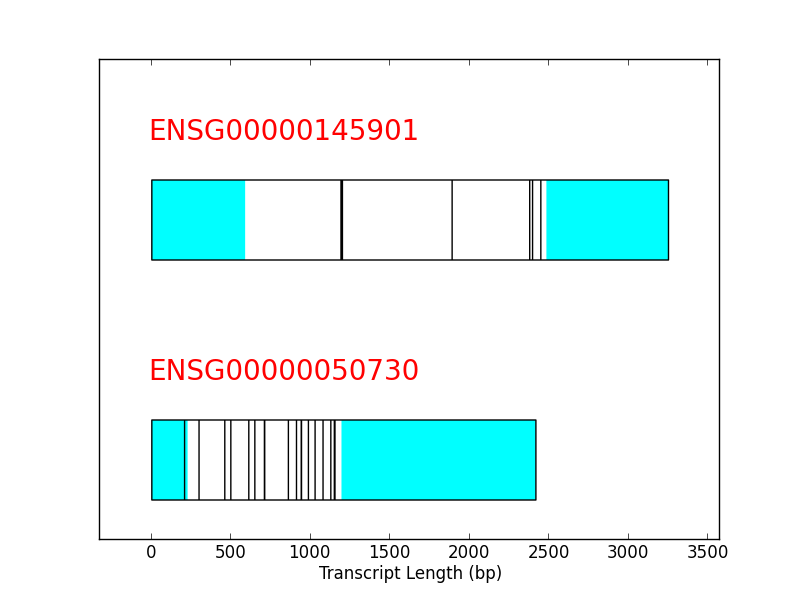

Supplement: Data file S2 [file rsob140029supp3.zip › rsob-14-0029-File010/Melanoma/ENSG00000050730_ENSG00000145901.png]

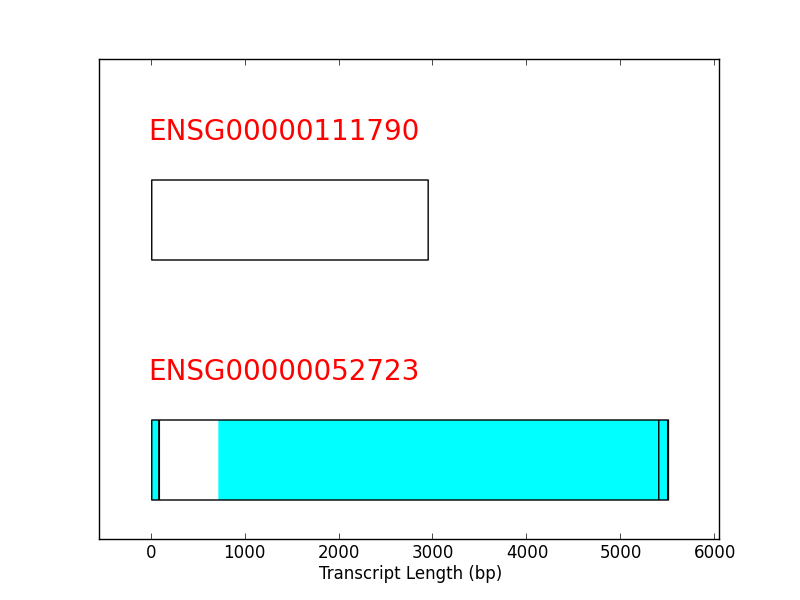

Supplement: Data file S2 [file rsob140029supp3.zip › rsob-14-0029-File010/Melanoma/ENSG00000052723_ENSG00000111790.png]

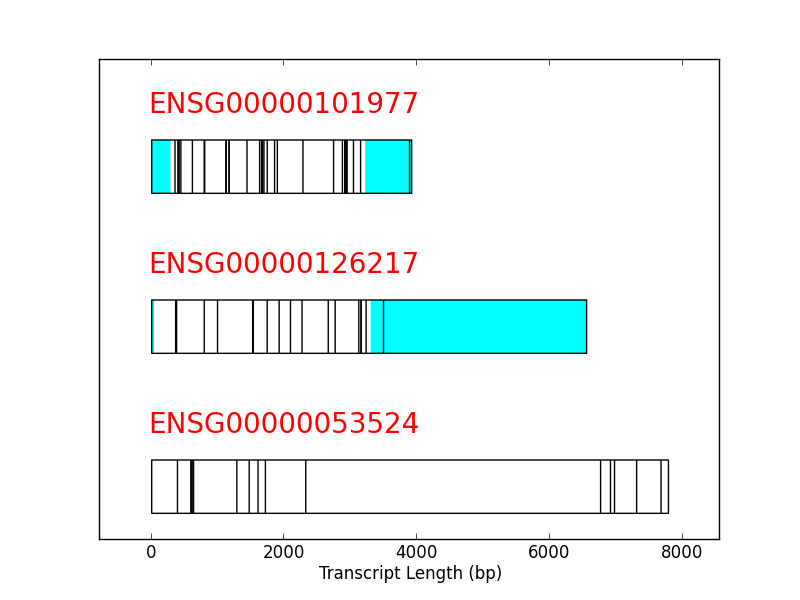

Supplement: Data file S2 [file rsob140029supp3.zip › rsob-14-0029-File010/Melanoma/ENSG00000053524_ENSG00000126217_ENSG00000101977.png]

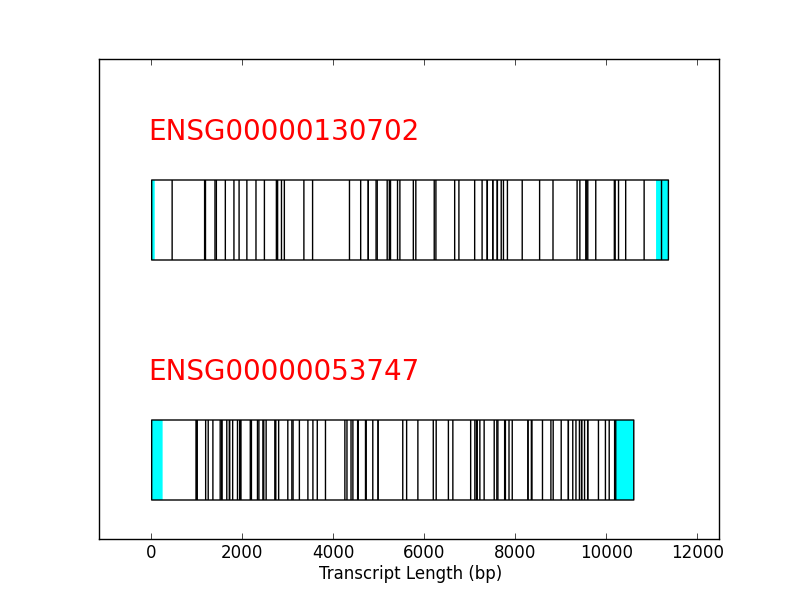

Supplement: Data file S2 [file rsob140029supp3.zip › rsob-14-0029-File010/Melanoma/ENSG00000053747_ENSG00000130702.png]

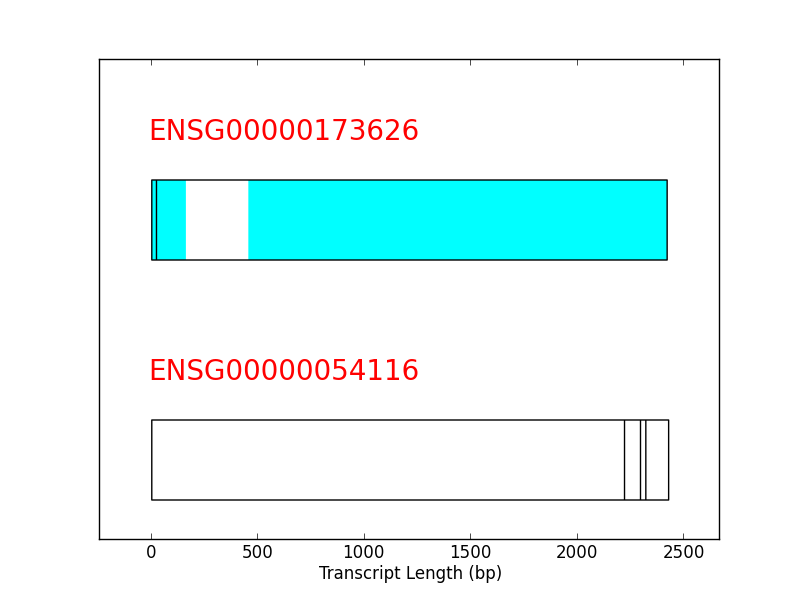

Supplement: Data file S2 [file rsob140029supp3.zip › rsob-14-0029-File010/Melanoma/ENSG00000054116_ENSG00000173626.png]

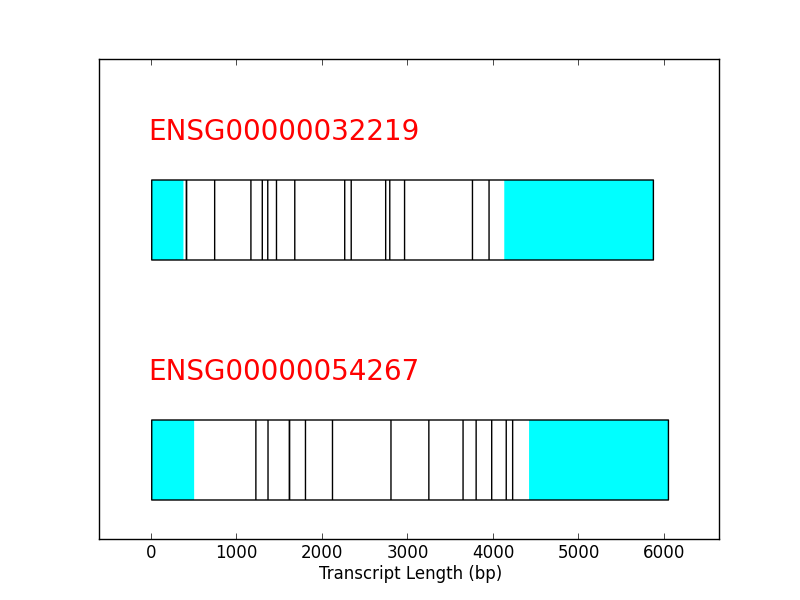

Supplement: Data file S2 [file rsob140029supp3.zip › rsob-14-0029-File010/Melanoma/ENSG00000054267_ENSG00000032219.png]

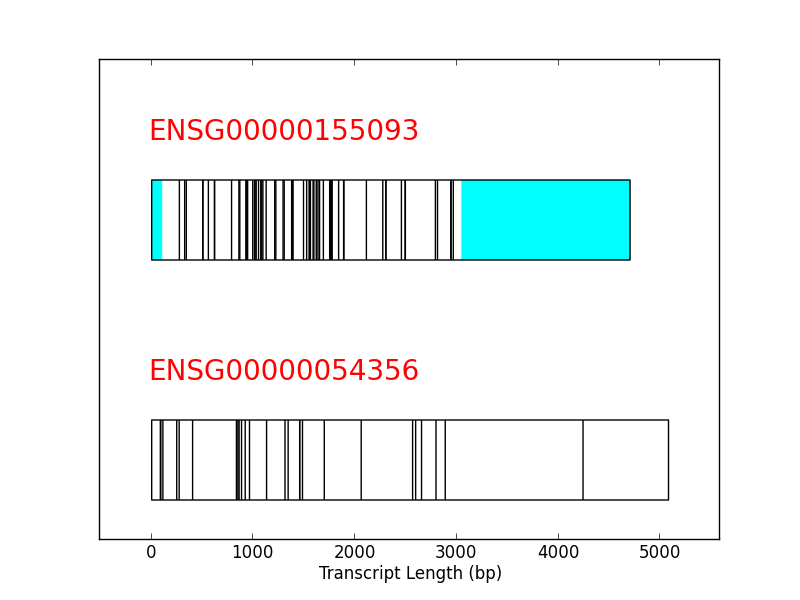

Supplement: Data file S2 [file rsob140029supp3.zip › rsob-14-0029-File010/Melanoma/ENSG00000054356_ENSG00000155093.png]

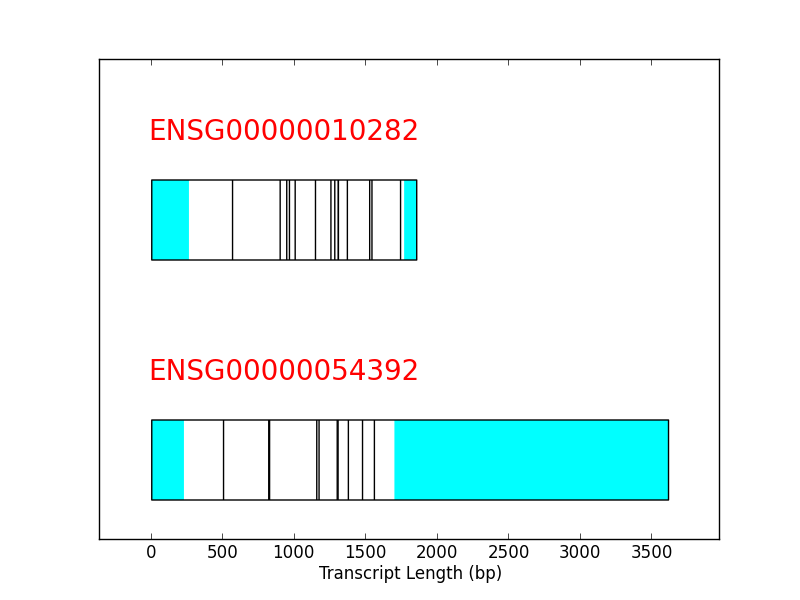

Supplement: Data file S2 [file rsob140029supp3.zip › rsob-14-0029-File010/Melanoma/ENSG00000054392_ENSG00000010282.png]

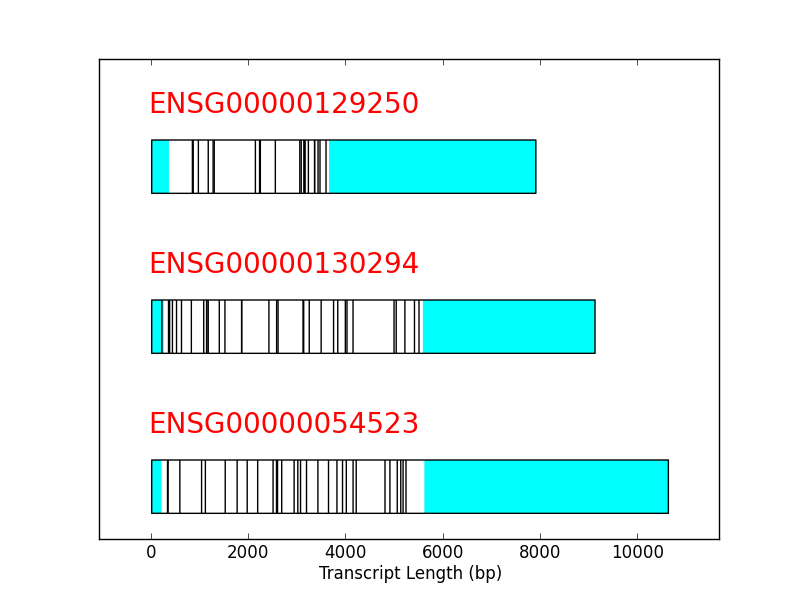

Supplement: Data file S2 [file rsob140029supp3.zip › rsob-14-0029-File010/Melanoma/ENSG00000054523_ENSG00000130294_ENSG00000129250.png]

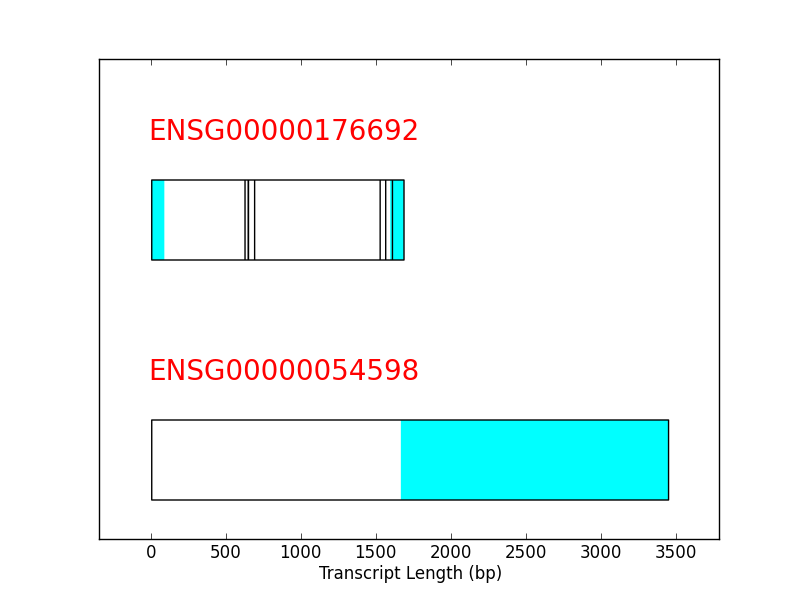

Supplement: Data file S2 [file rsob140029supp3.zip › rsob-14-0029-File010/Melanoma/ENSG00000054598_ENSG00000176692.png]

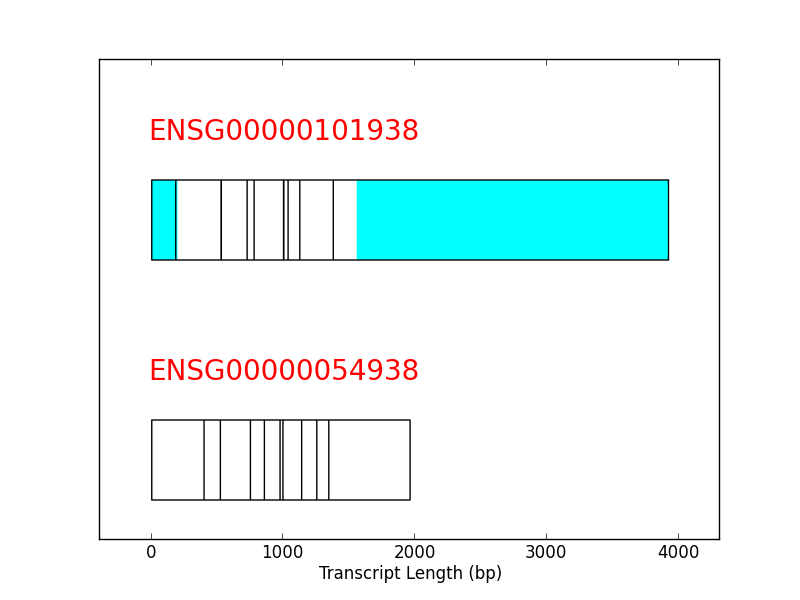

Supplement: Data file S2 [file rsob140029supp3.zip › rsob-14-0029-File010/Melanoma/ENSG00000054938_ENSG00000101938.png]

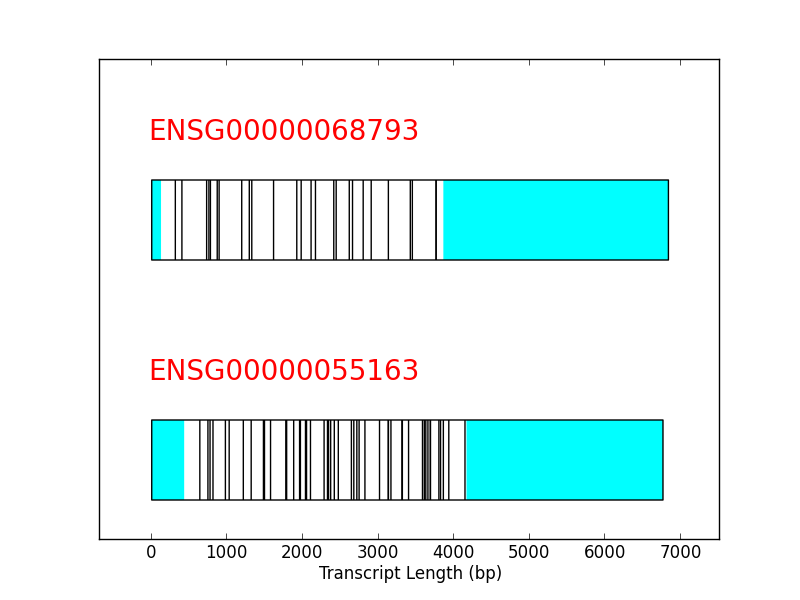

Supplement: Data file S2 [file rsob140029supp3.zip › rsob-14-0029-File010/Melanoma/ENSG00000055163_ENSG00000068793.png]

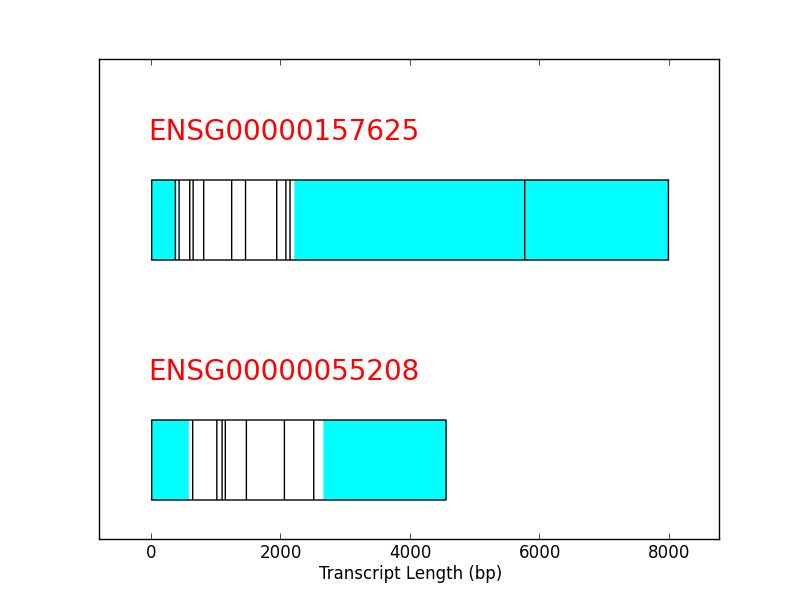

Supplement: Data file S2 [file rsob140029supp3.zip › rsob-14-0029-File010/Melanoma/ENSG00000055208_ENSG00000157625.png]

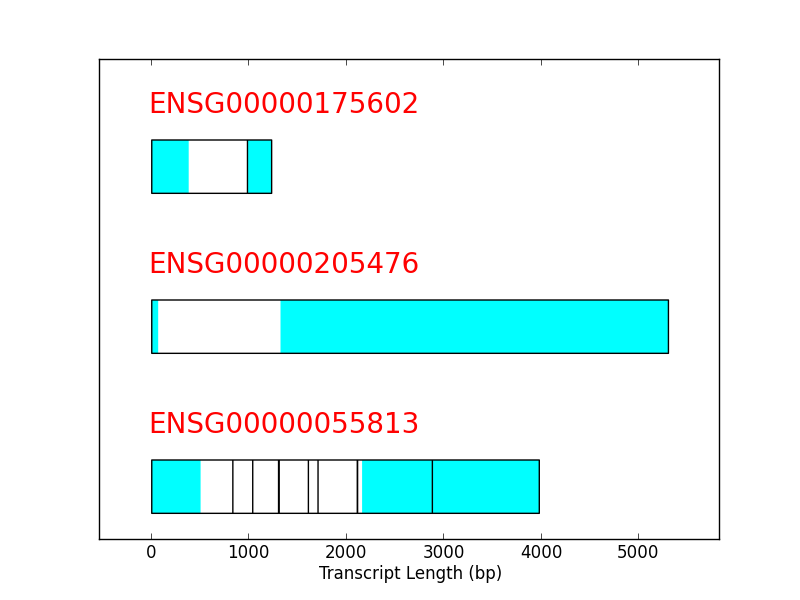

Supplement: Data file S2 [file rsob140029supp3.zip › rsob-14-0029-File010/Melanoma/ENSG00000055813_ENSG00000205476_ENSG00000175602.png]

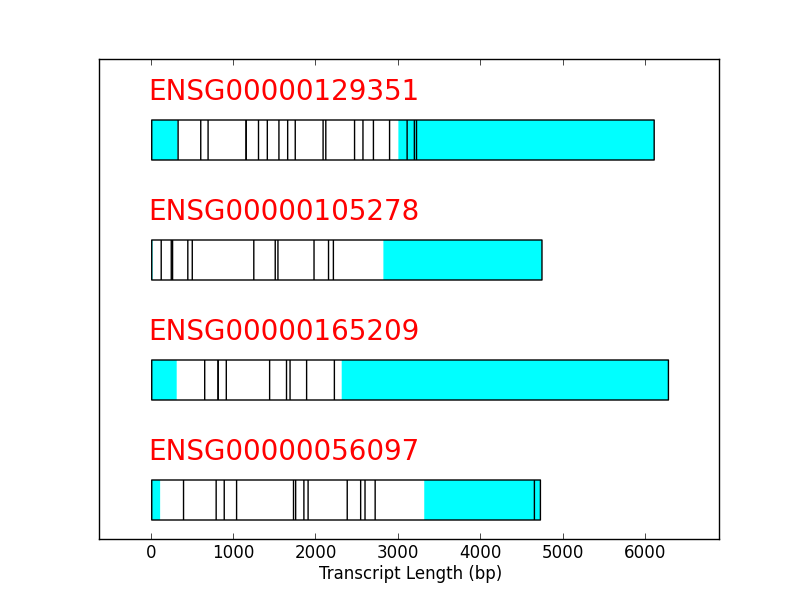

Supplement: Data file S2 [file rsob140029supp3.zip › rsob-14-0029-File010/Melanoma/ENSG00000056097_ENSG00000165209_ENSG00000105278_ENSG00000129351.png]

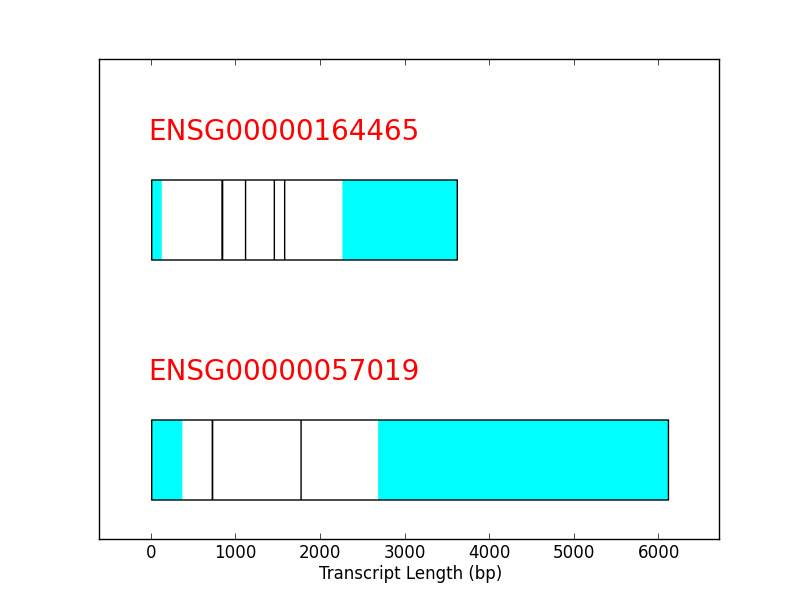

Supplement: Data file S2 [file rsob140029supp3.zip › rsob-14-0029-File010/Melanoma/ENSG00000057019_ENSG00000164465.png]

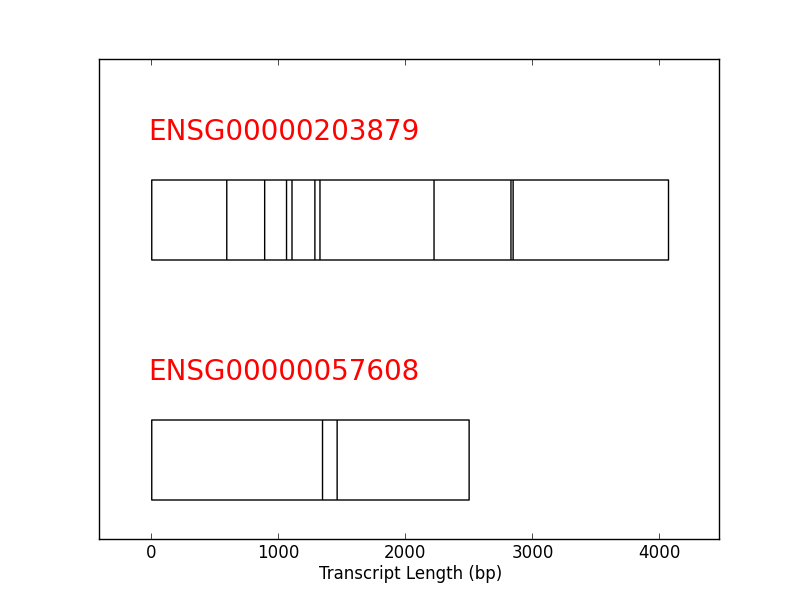

Supplement: Data file S2 [file rsob140029supp3.zip › rsob-14-0029-File010/Melanoma/ENSG00000057608_ENSG00000203879.png]

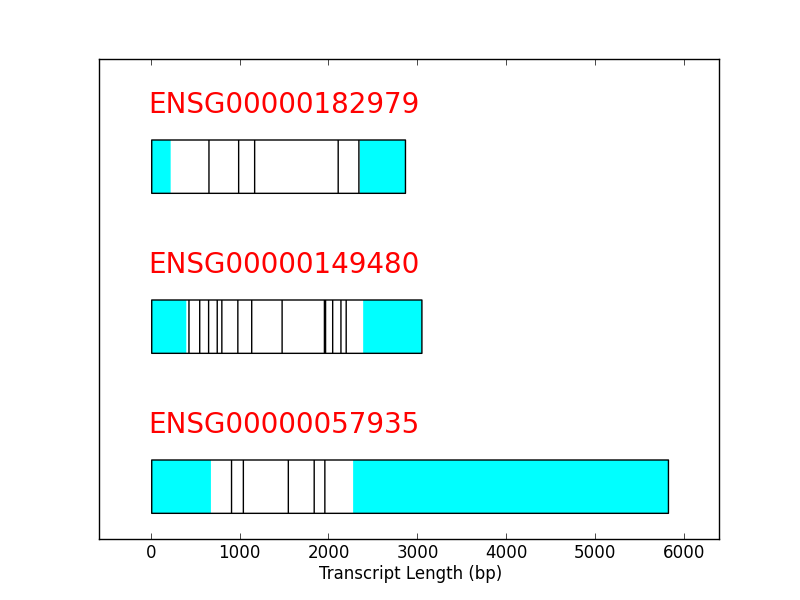

Supplement: Data file S2 [file rsob140029supp3.zip › rsob-14-0029-File010/Melanoma/ENSG00000057935_ENSG00000149480_ENSG00000182979.png]

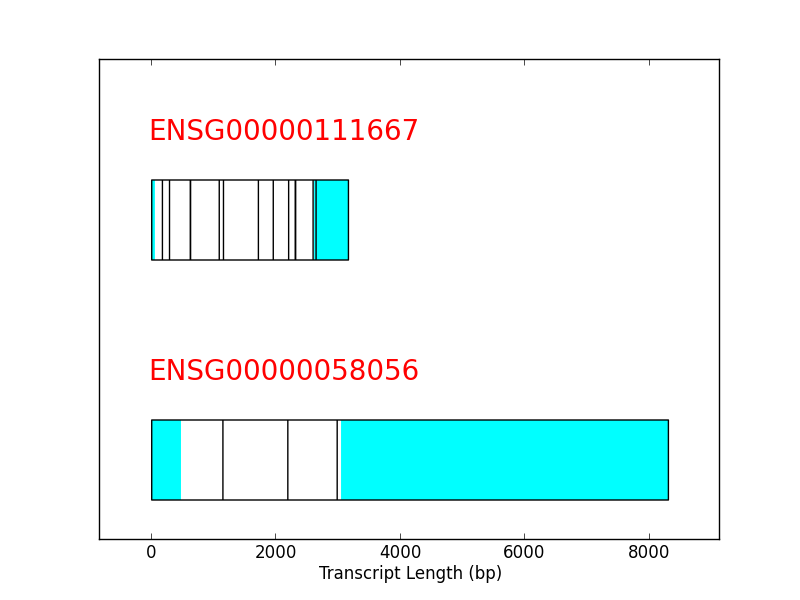

Supplement: Data file S2 [file rsob140029supp3.zip › rsob-14-0029-File010/Melanoma/ENSG00000058056_ENSG00000111667.png]

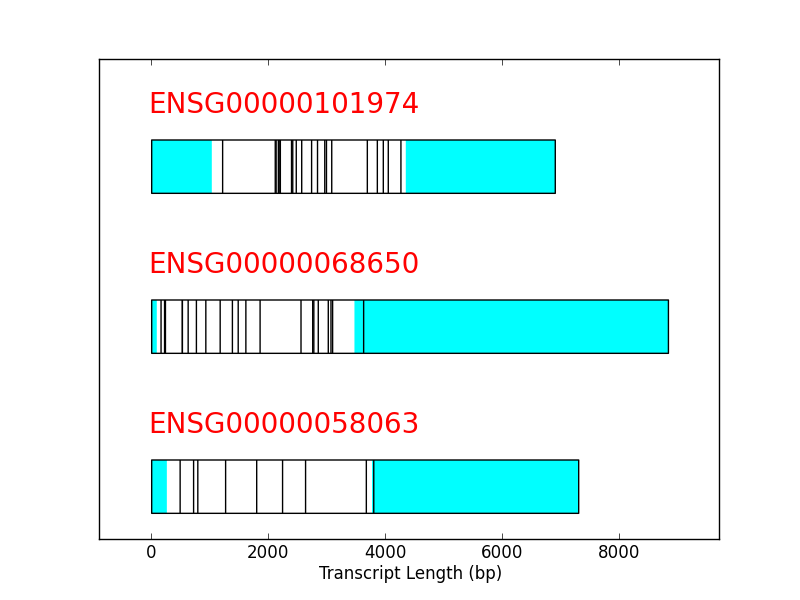

Supplement: Data file S2 [file rsob140029supp3.zip › rsob-14-0029-File010/Melanoma/ENSG00000058063_ENSG00000068650_ENSG00000101974.png]

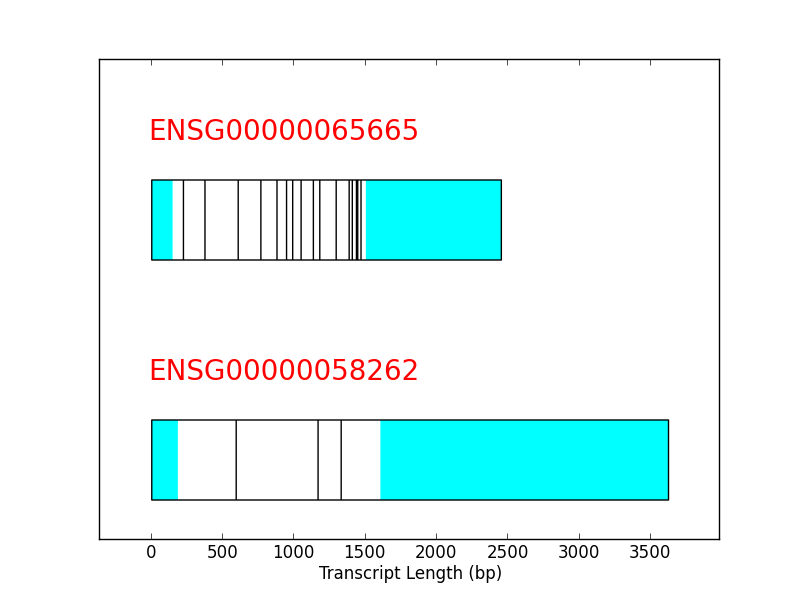

Supplement: Data file S2 [file rsob140029supp3.zip › rsob-14-0029-File010/Melanoma/ENSG00000058262_ENSG00000065665.png]

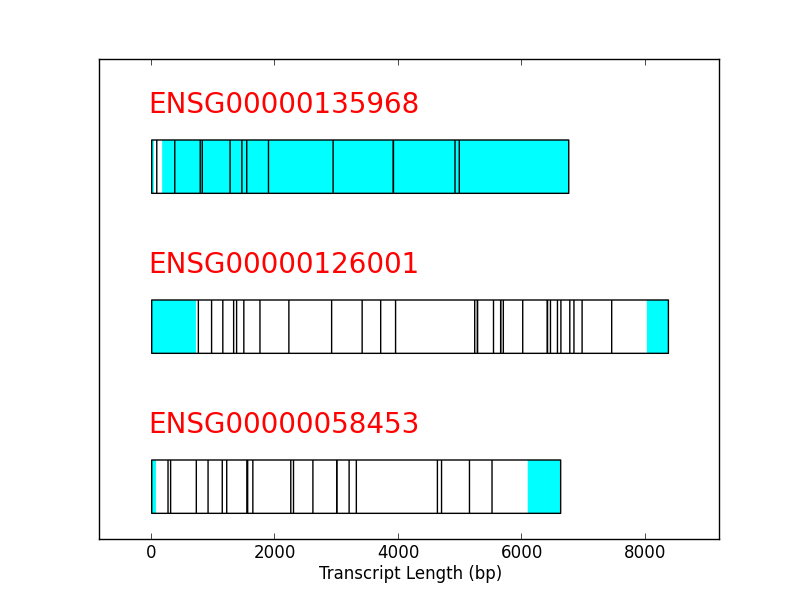

Supplement: Data file S2 [file rsob140029supp3.zip › rsob-14-0029-File010/Melanoma/ENSG00000058453_ENSG00000126001_ENSG00000135968.png]

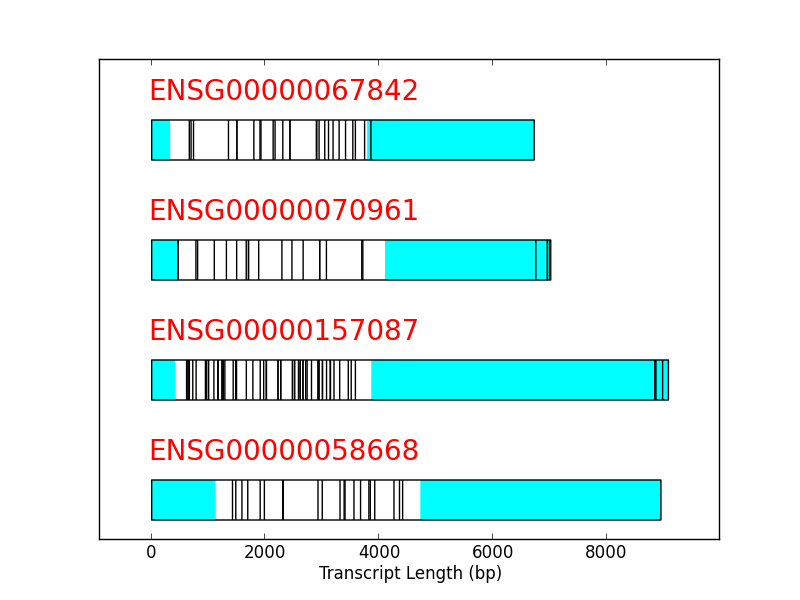

Supplement: Data file S2 [file rsob140029supp3.zip › rsob-14-0029-File010/Melanoma/ENSG00000058668_ENSG00000157087_ENSG00000070961_ENSG00000067842.png]

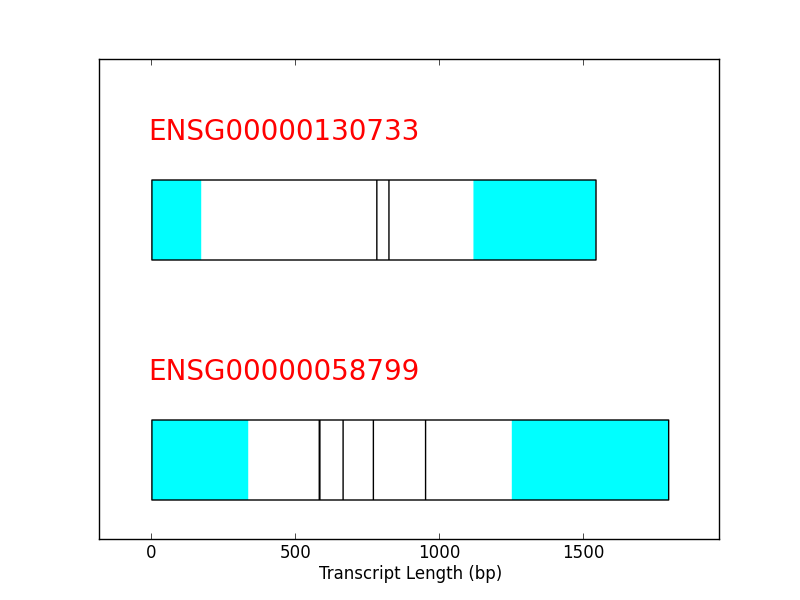

Supplement: Data file S2 [file rsob140029supp3.zip › rsob-14-0029-File010/Melanoma/ENSG00000058799_ENSG00000130733.png]

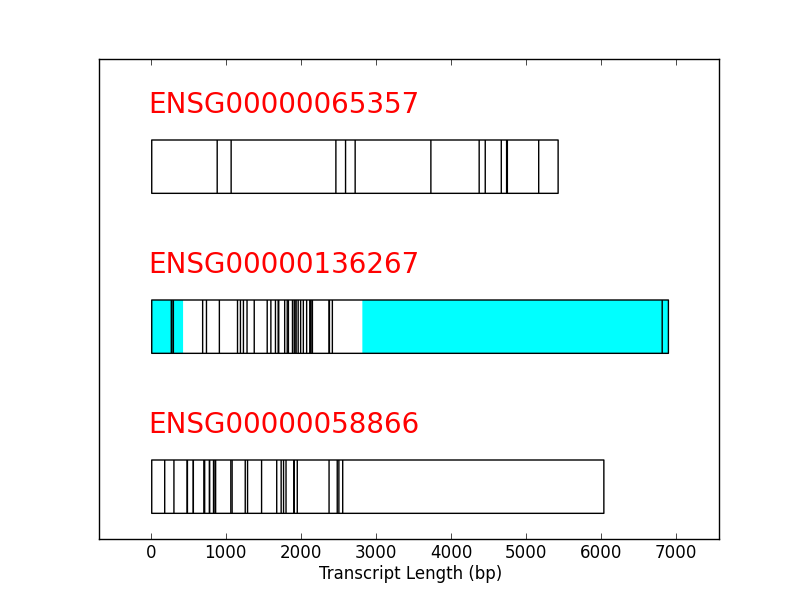

Supplement: Data file S2 [file rsob140029supp3.zip › rsob-14-0029-File010/Melanoma/ENSG00000058866_ENSG00000136267_ENSG00000065357.png]

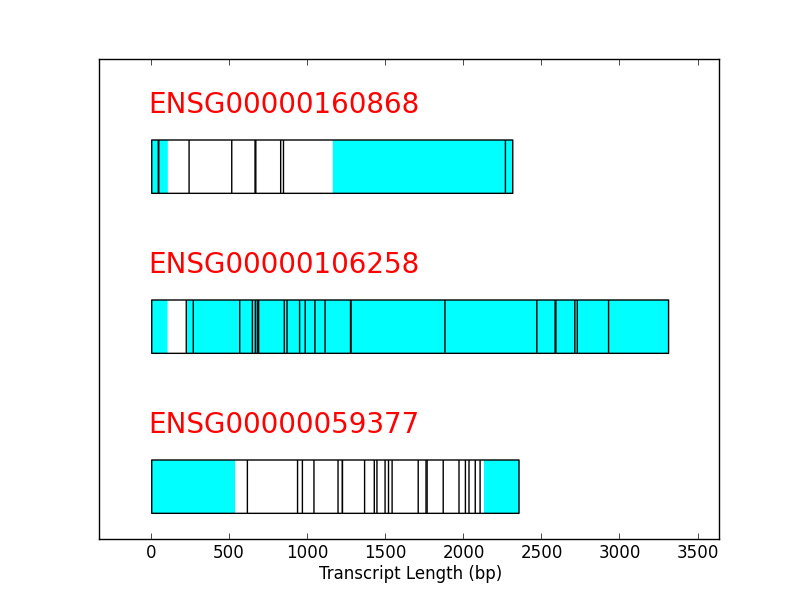

Supplement: Data file S2 [file rsob140029supp3.zip › rsob-14-0029-File010/Melanoma/ENSG00000059377_ENSG00000106258_ENSG00000160868.png]

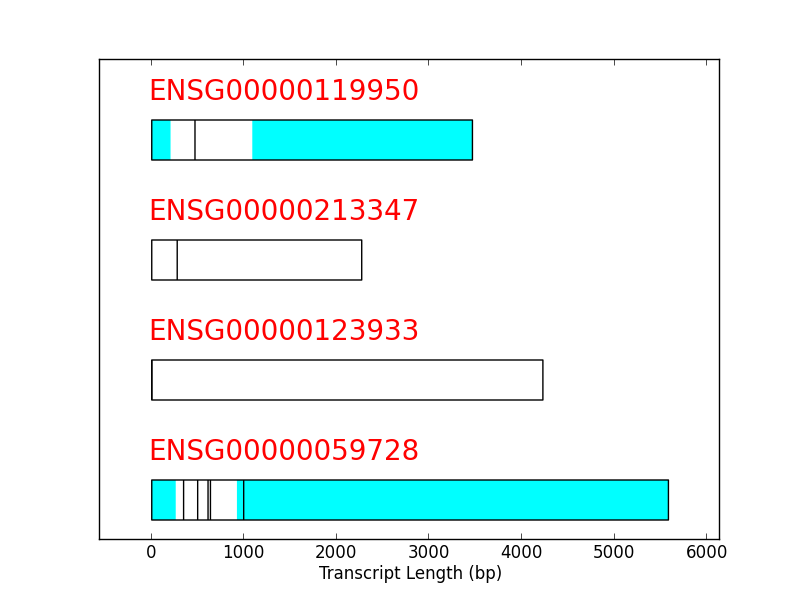

Supplement: Data file S2 [file rsob140029supp3.zip › rsob-14-0029-File010/Melanoma/ENSG00000059728_ENSG00000123933_ENSG00000213347_ENSG00000119950.png]

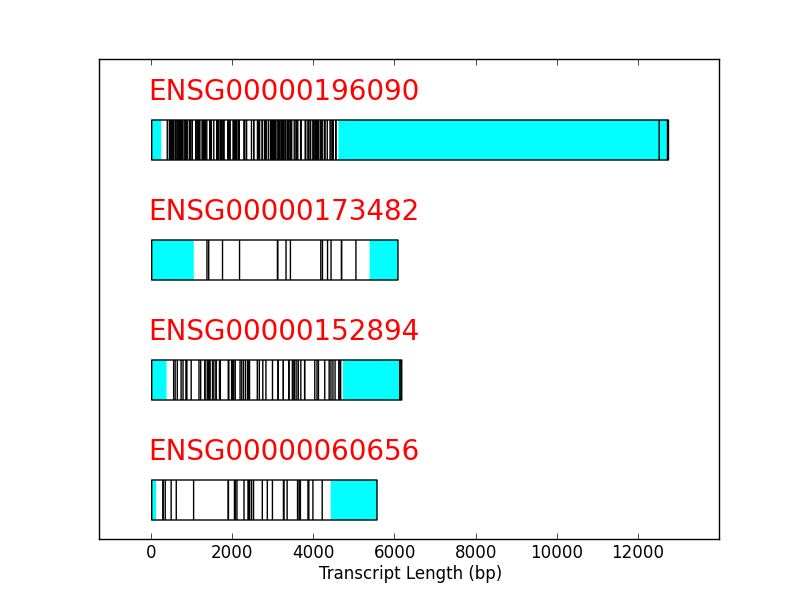

Supplement: Data file S2 [file rsob140029supp3.zip › rsob-14-0029-File010/Melanoma/ENSG00000060656_ENSG00000152894_ENSG00000173482_ENSG00000196090.png]

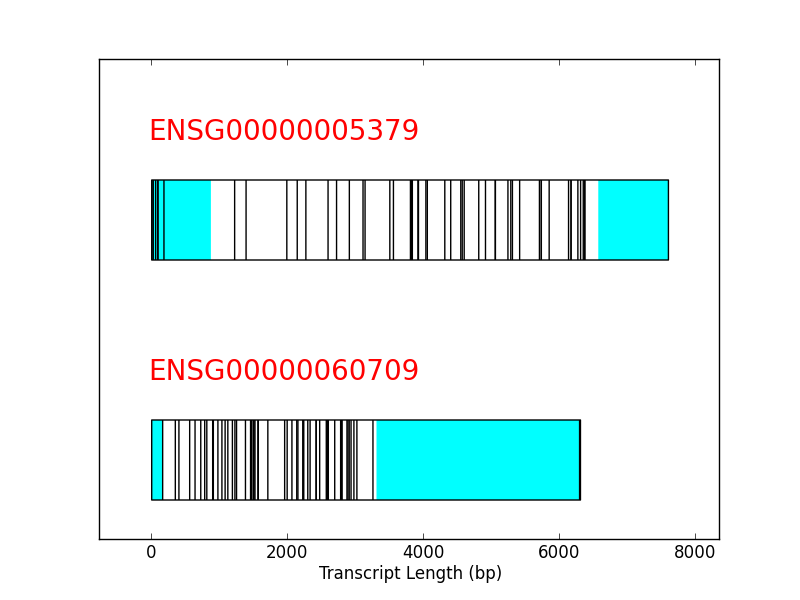

Supplement: Data file S2 [file rsob140029supp3.zip › rsob-14-0029-File010/Melanoma/ENSG00000060709_ENSG00000005379.png]

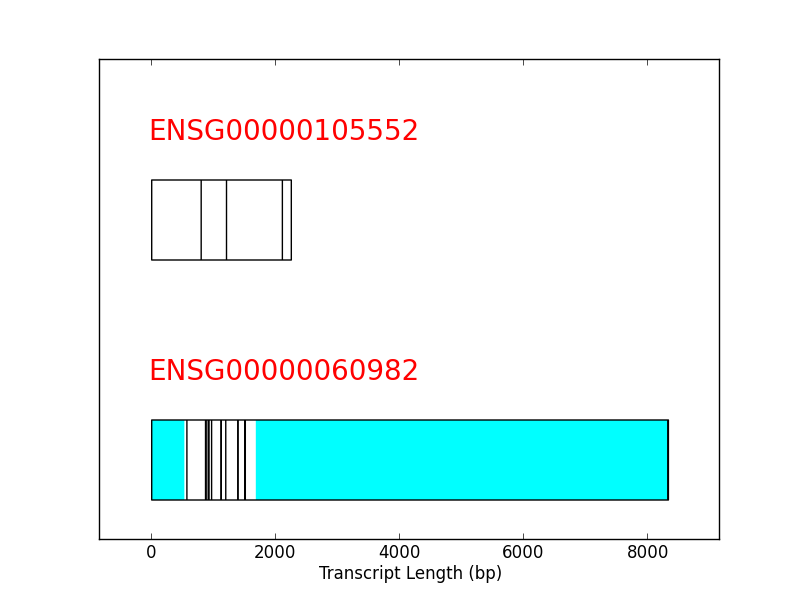

Supplement: Data file S2 [file rsob140029supp3.zip › rsob-14-0029-File010/Melanoma/ENSG00000060982_ENSG00000105552.png]

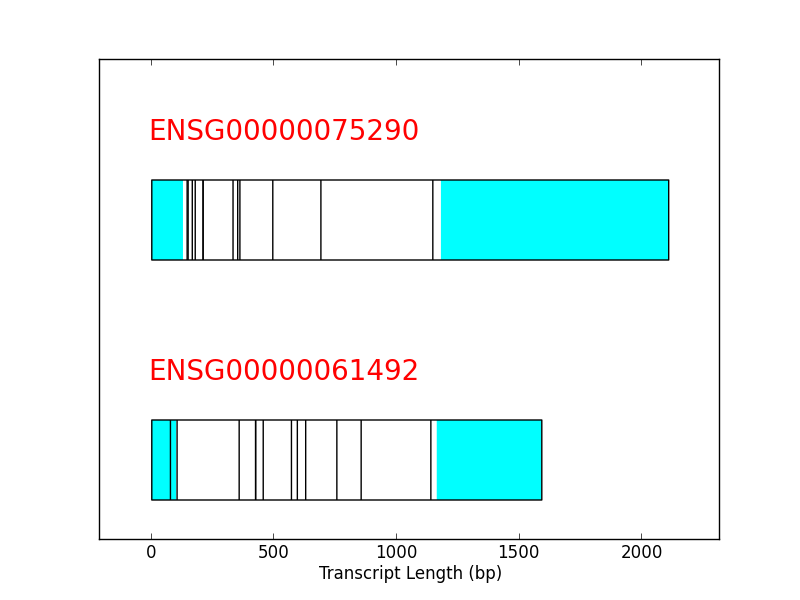

Supplement: Data file S2 [file rsob140029supp3.zip › rsob-14-0029-File010/Melanoma/ENSG00000061492_ENSG00000075290.png]

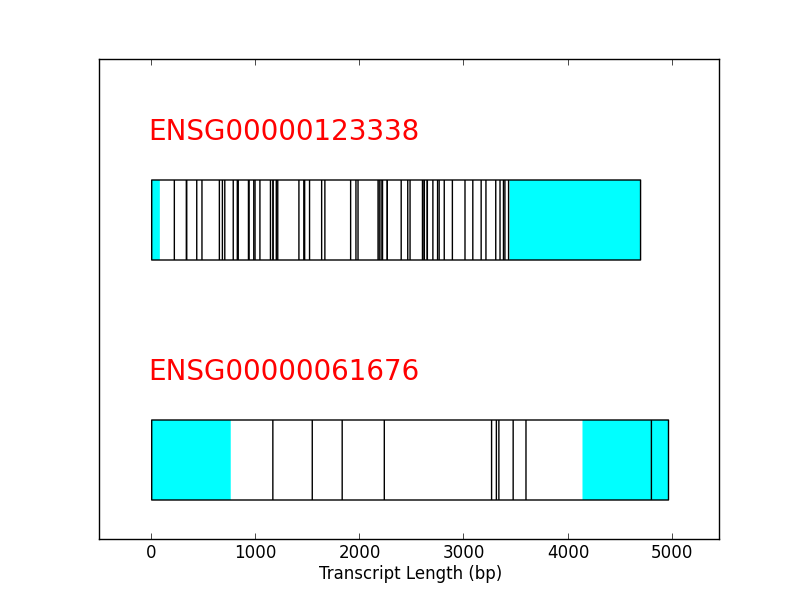

Supplement: Data file S2 [file rsob140029supp3.zip › rsob-14-0029-File010/Melanoma/ENSG00000061676_ENSG00000123338.png]

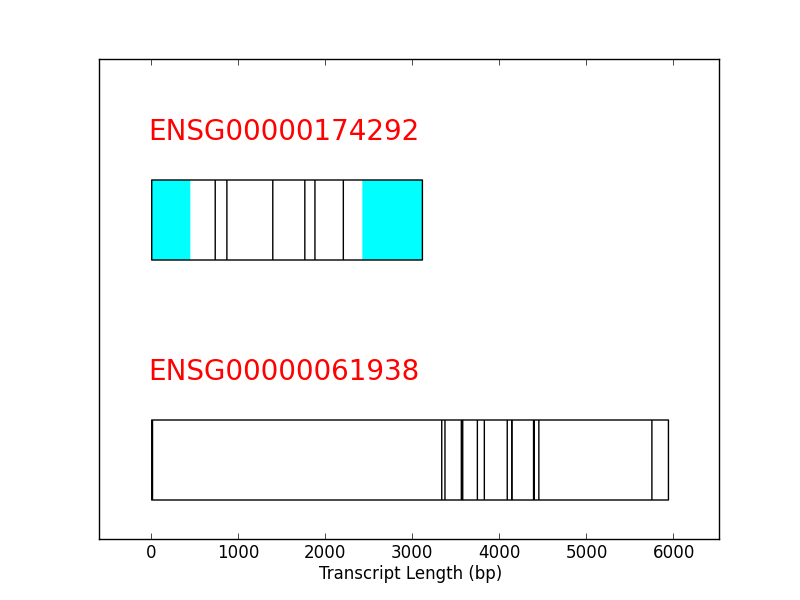

Supplement: Data file S2 [file rsob140029supp3.zip › rsob-14-0029-File010/Melanoma/ENSG00000061938_ENSG00000174292.png]

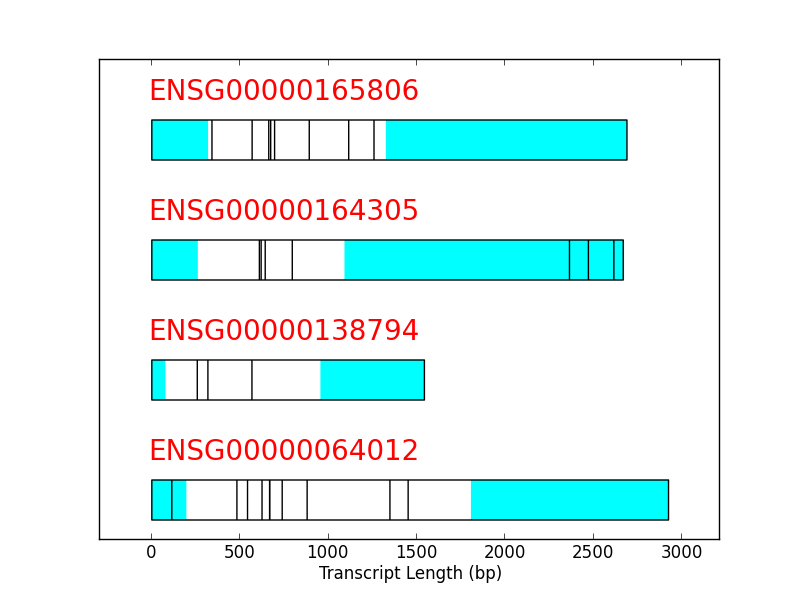

Supplement: Data file S2 [file rsob140029supp3.zip › rsob-14-0029-File010/Melanoma/ENSG00000064012_ENSG00000138794_ENSG00000164305_ENSG00000165806.png]

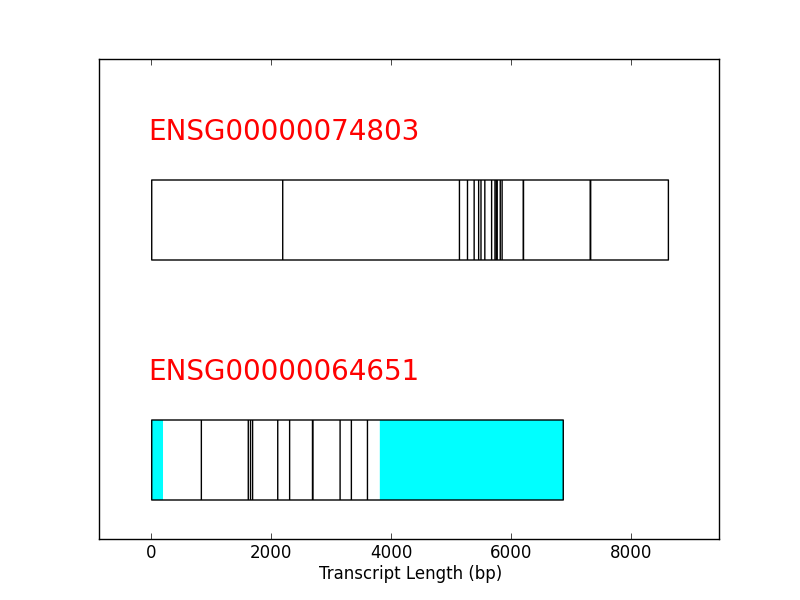

Supplement: Data file S2 [file rsob140029supp3.zip › rsob-14-0029-File010/Melanoma/ENSG00000064651_ENSG00000074803.png]

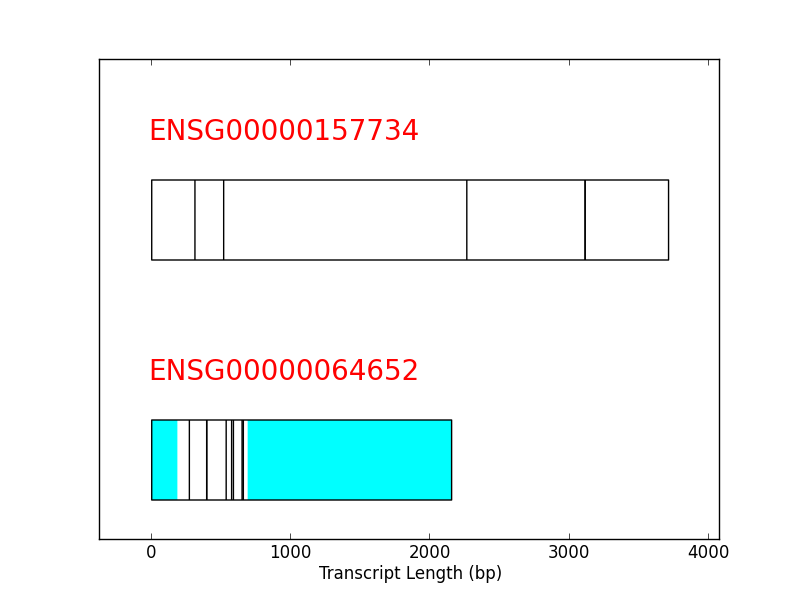

Supplement: Data file S2 [file rsob140029supp3.zip › rsob-14-0029-File010/Melanoma/ENSG00000064652_ENSG00000157734.png]

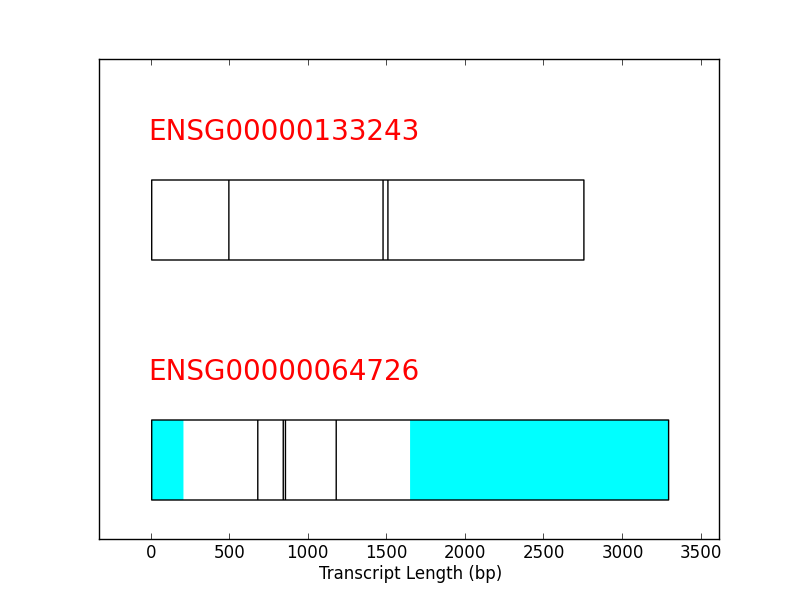

Supplement: Data file S2 [file rsob140029supp3.zip › rsob-14-0029-File010/Melanoma/ENSG00000064726_ENSG00000133243.png]

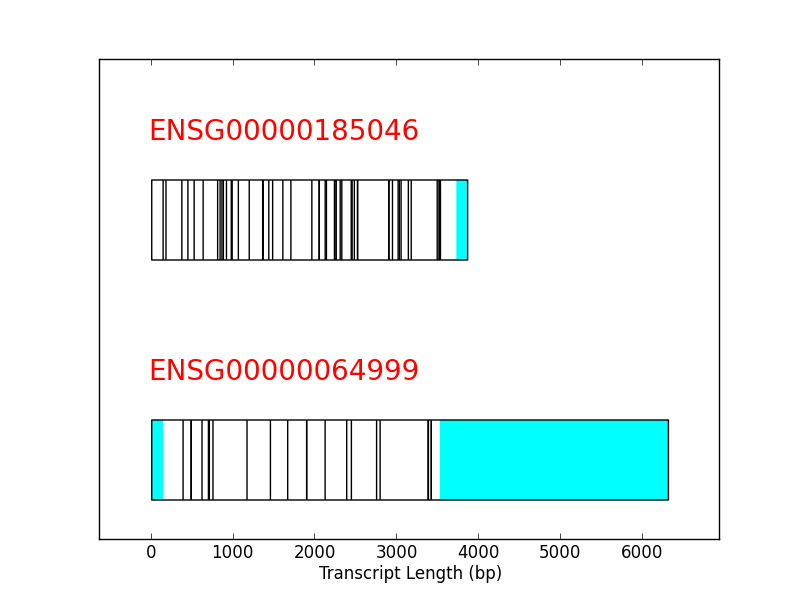

Supplement: Data file S2 [file rsob140029supp3.zip › rsob-14-0029-File010/Melanoma/ENSG00000064999_ENSG00000185046.png]

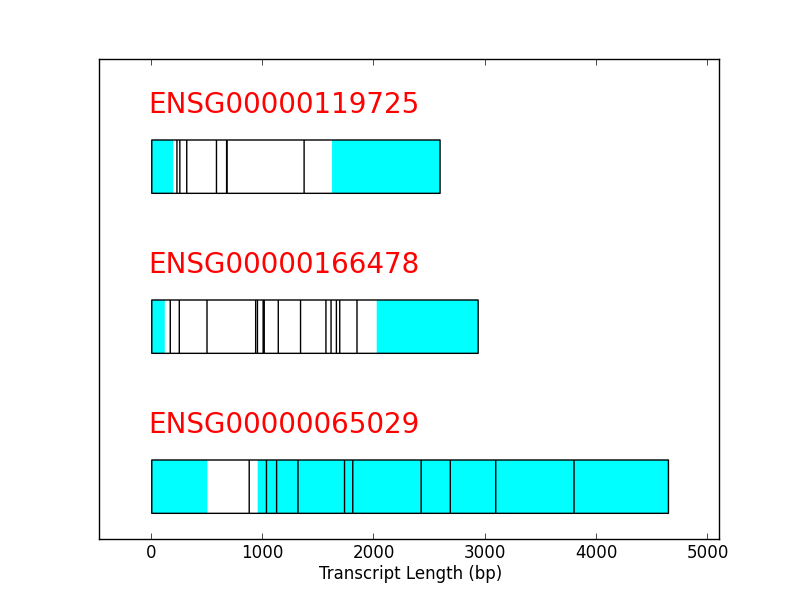

Supplement: Data file S2 [file rsob140029supp3.zip › rsob-14-0029-File010/Melanoma/ENSG00000065029_ENSG00000166478_ENSG00000119725.png]

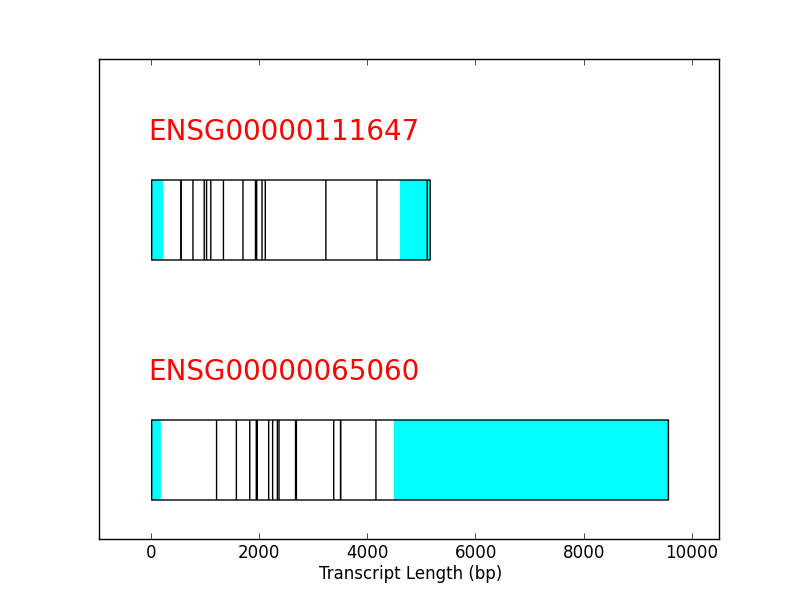

Supplement: Data file S2 [file rsob140029supp3.zip › rsob-14-0029-File010/Melanoma/ENSG00000065060_ENSG00000111647.png]

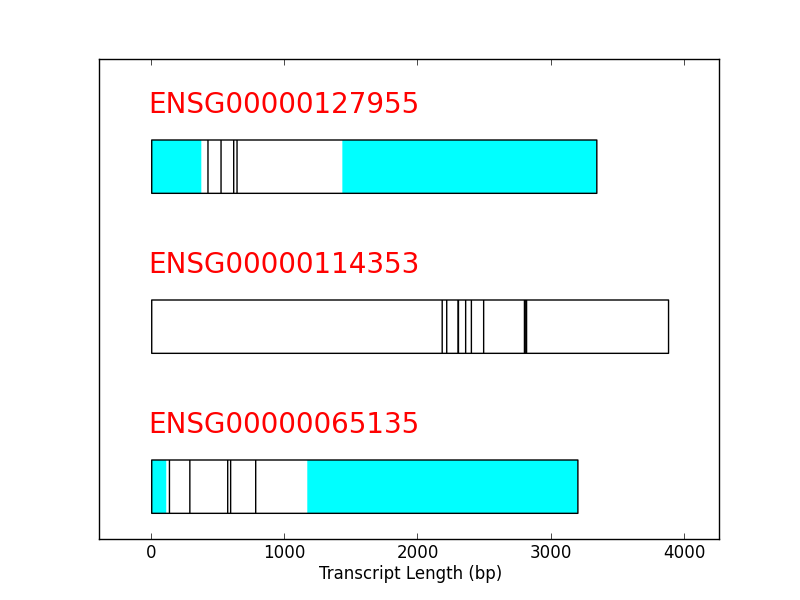

Supplement: Data file S2 [file rsob140029supp3.zip › rsob-14-0029-File010/Melanoma/ENSG00000065135_ENSG00000114353_ENSG00000127955.png]

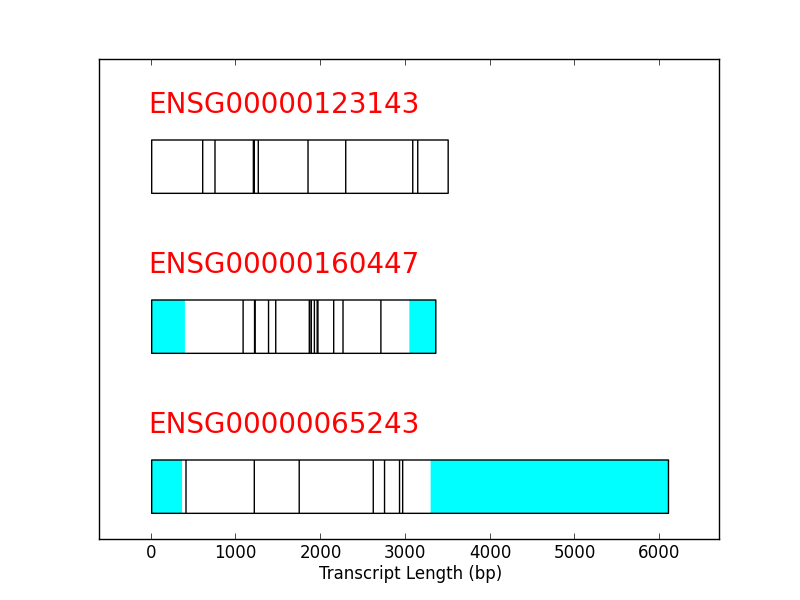

Supplement: Data file S2 [file rsob140029supp3.zip › rsob-14-0029-File010/Melanoma/ENSG00000065243_ENSG00000160447_ENSG00000123143.png]

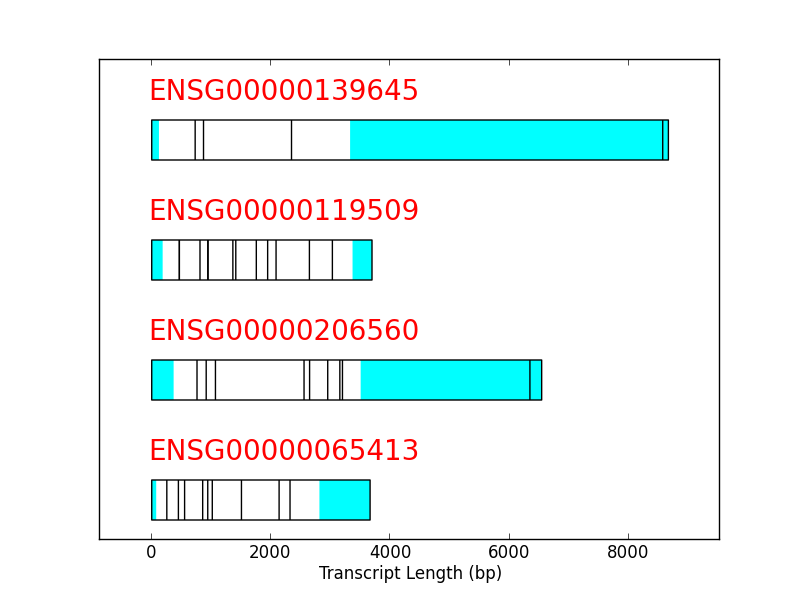

Supplement: Data file S2 [file rsob140029supp3.zip › rsob-14-0029-File010/Melanoma/ENSG00000065413_ENSG00000206560_ENSG00000119509_ENSG00000139645.png]

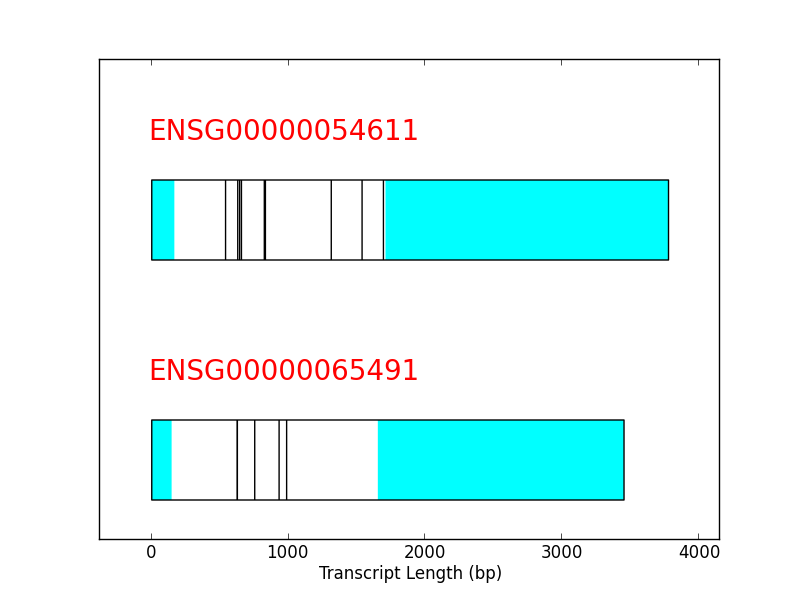

Supplement: Data file S2 [file rsob140029supp3.zip › rsob-14-0029-File010/Melanoma/ENSG00000065491_ENSG00000054611.png]

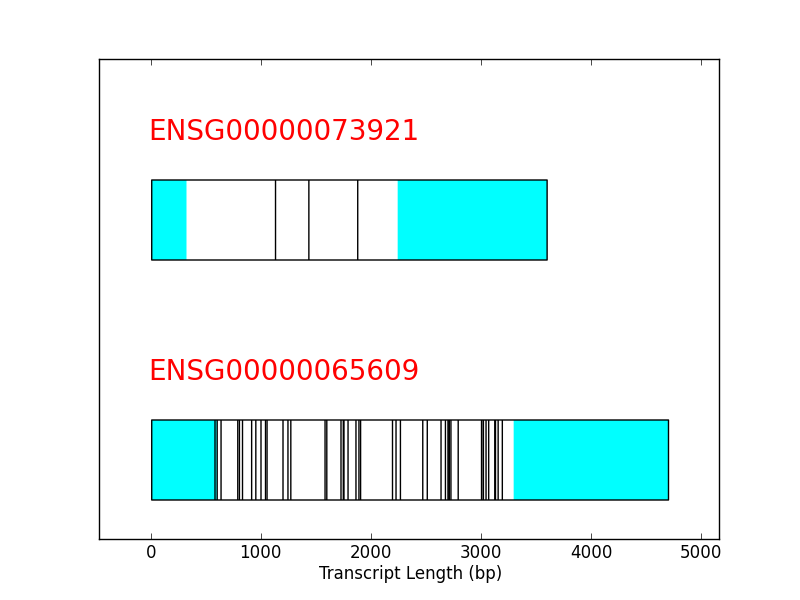

Supplement: Data file S2 [file rsob140029supp3.zip › rsob-14-0029-File010/Melanoma/ENSG00000065609_ENSG00000073921.png]

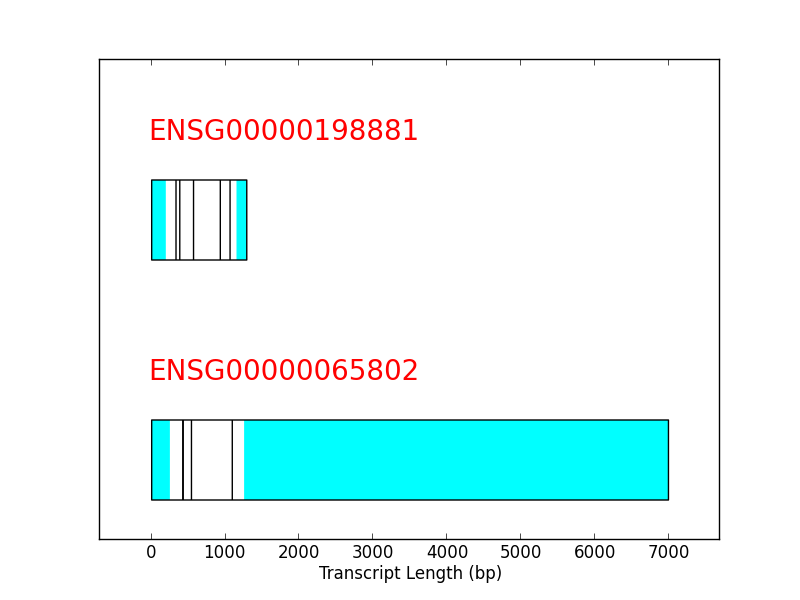

Supplement: Data file S2 [file rsob140029supp3.zip › rsob-14-0029-File010/Melanoma/ENSG00000065802_ENSG00000198881.png]

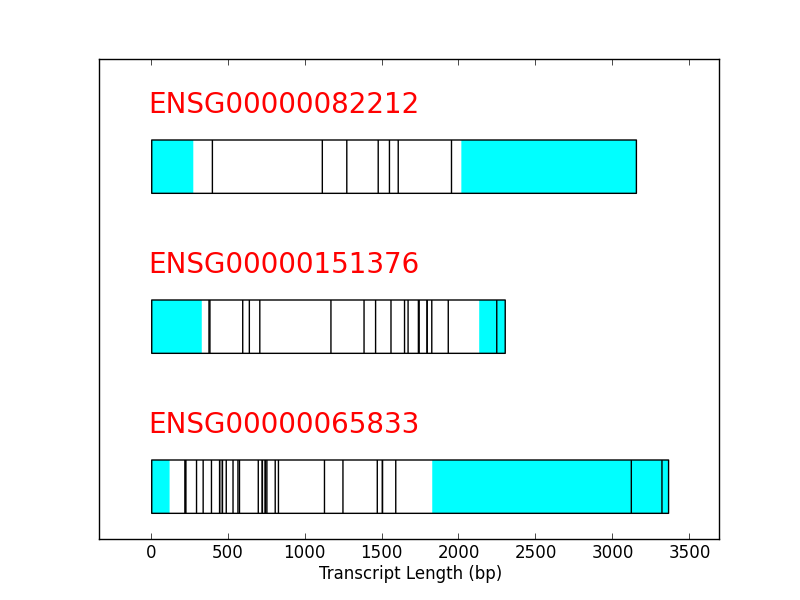

Supplement: Data file S2 [file rsob140029supp3.zip › rsob-14-0029-File010/Melanoma/ENSG00000065833_ENSG00000151376_ENSG00000082212.png]

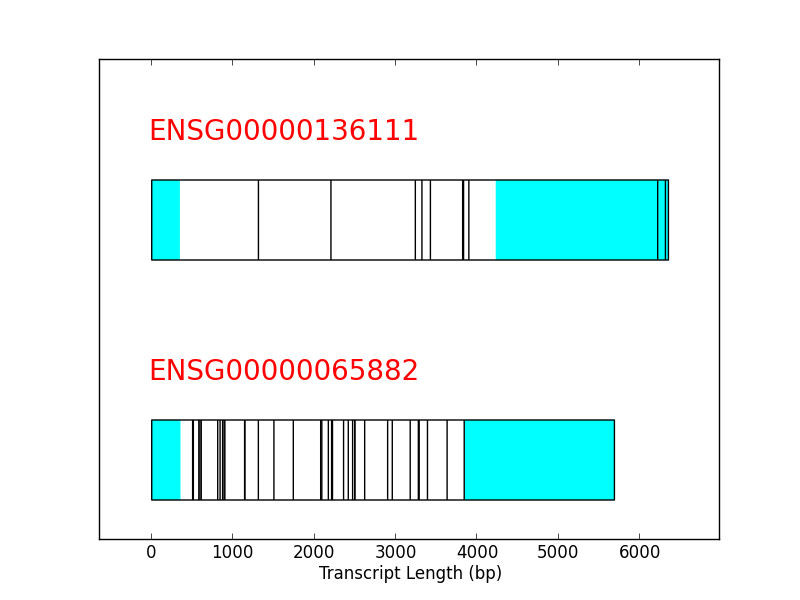

Supplement: Data file S2 [file rsob140029supp3.zip › rsob-14-0029-File010/Melanoma/ENSG00000065882_ENSG00000136111.png]

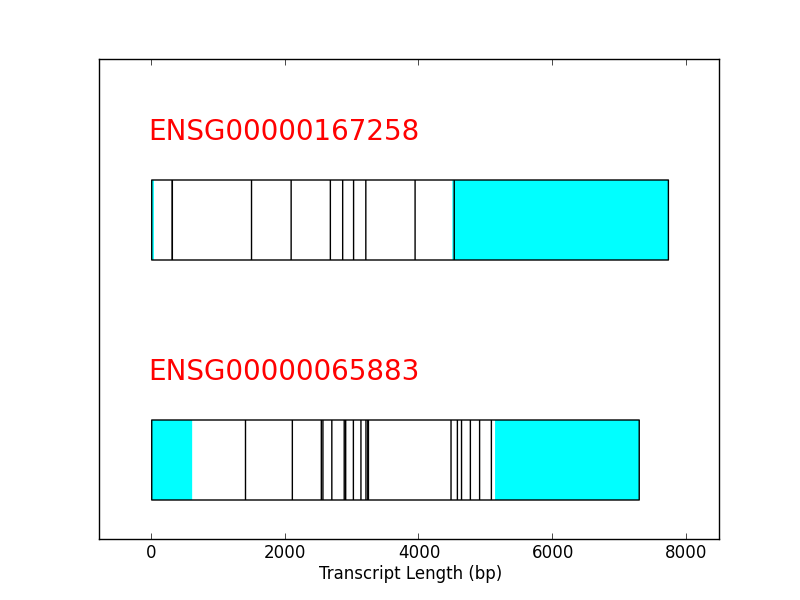

Supplement: Data file S2 [file rsob140029supp3.zip › rsob-14-0029-File010/Melanoma/ENSG00000065883_ENSG00000167258.png]

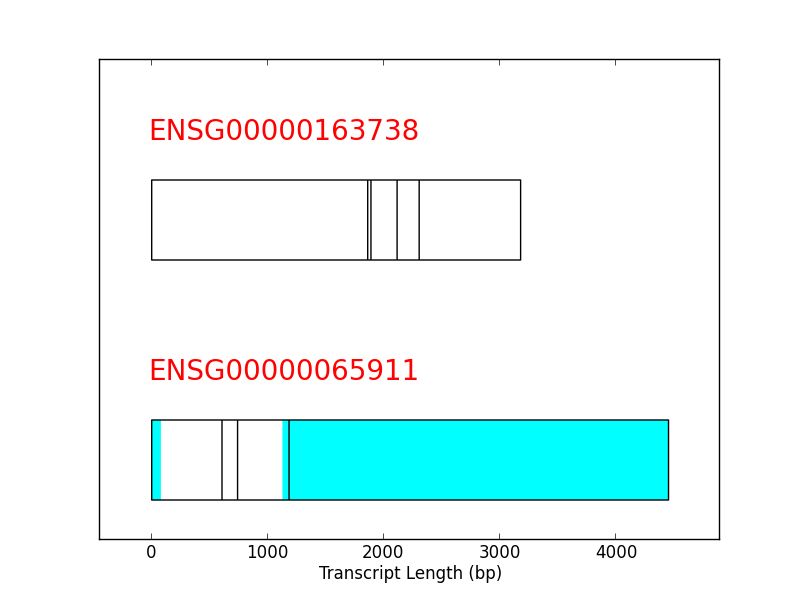

Supplement: Data file S2 [file rsob140029supp3.zip › rsob-14-0029-File010/Melanoma/ENSG00000065911_ENSG00000163738.png]

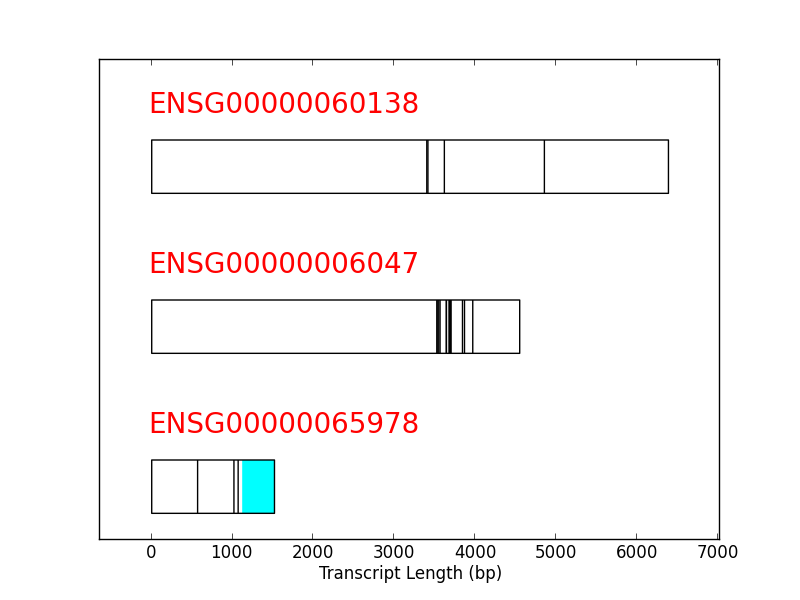

Supplement: Data file S2 [file rsob140029supp3.zip › rsob-14-0029-File010/Melanoma/ENSG00000065978_ENSG00000006047_ENSG00000060138.png]

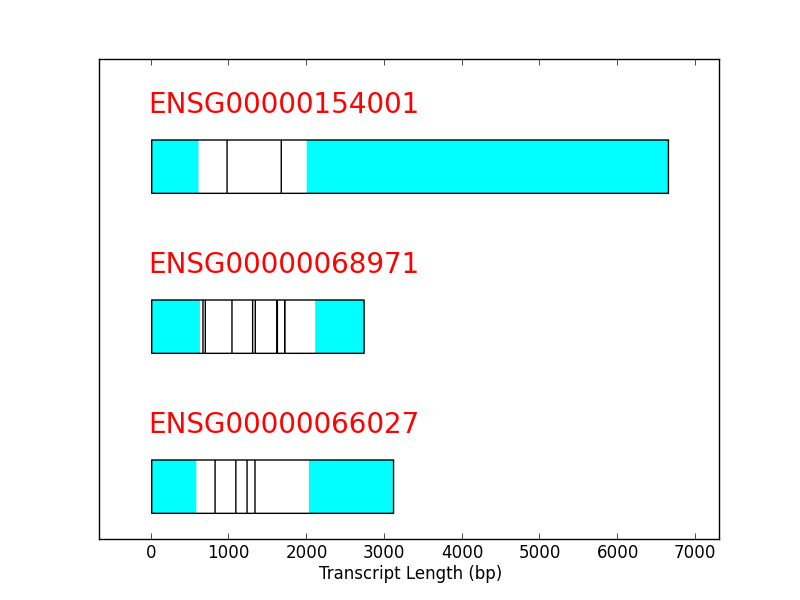

Supplement: Data file S2 [file rsob140029supp3.zip › rsob-14-0029-File010/Melanoma/ENSG00000066027_ENSG00000068971_ENSG00000154001.png]

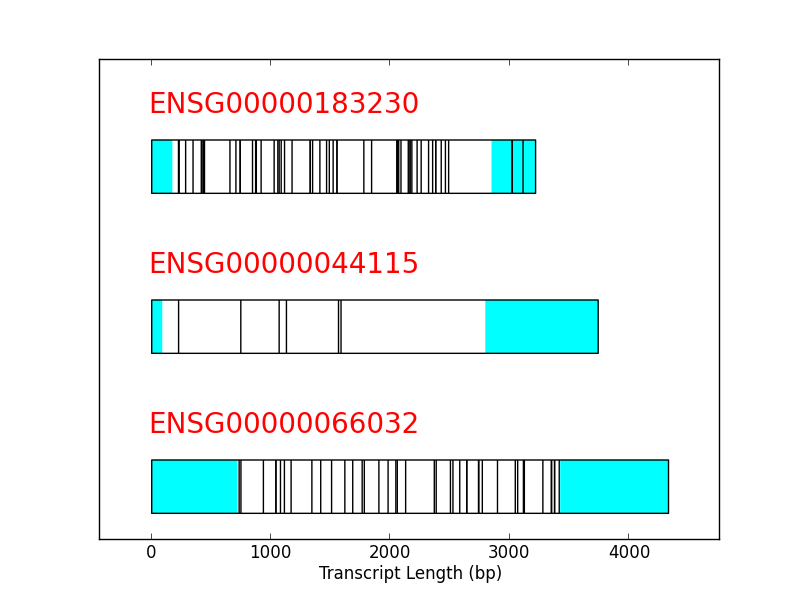

Supplement: Data file S2 [file rsob140029supp3.zip › rsob-14-0029-File010/Melanoma/ENSG00000066032_ENSG00000044115_ENSG00000183230.png]

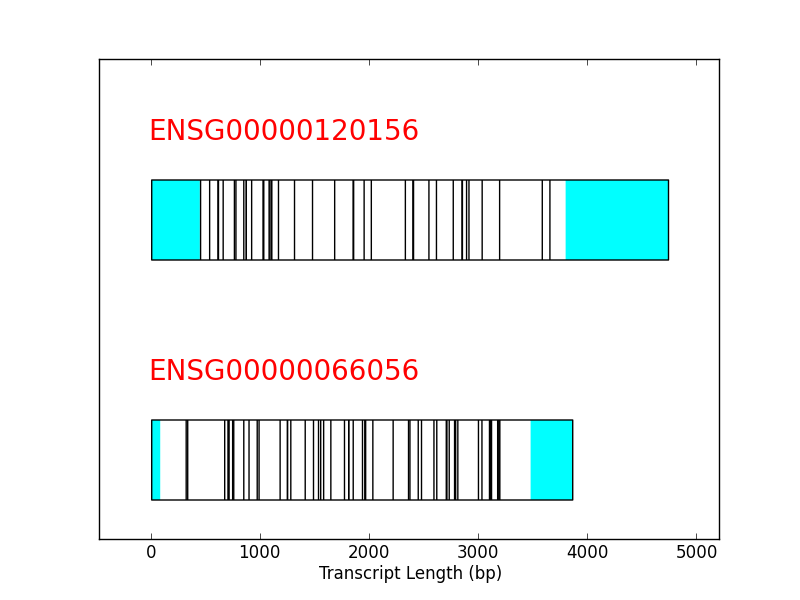

Supplement: Data file S2 [file rsob140029supp3.zip › rsob-14-0029-File010/Melanoma/ENSG00000066056_ENSG00000120156.png]

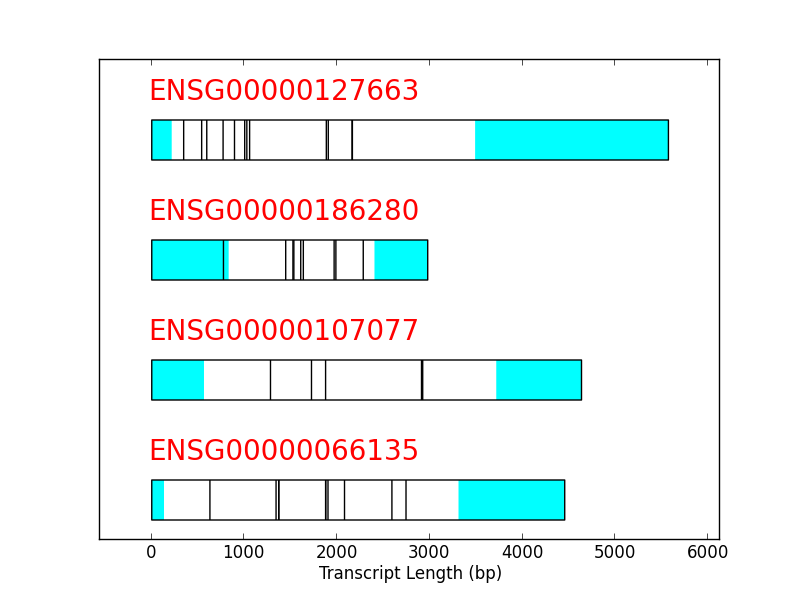

Supplement: Data file S2 [file rsob140029supp3.zip › rsob-14-0029-File010/Melanoma/ENSG00000066135_ENSG00000107077_ENSG00000186280_ENSG00000127663.png]

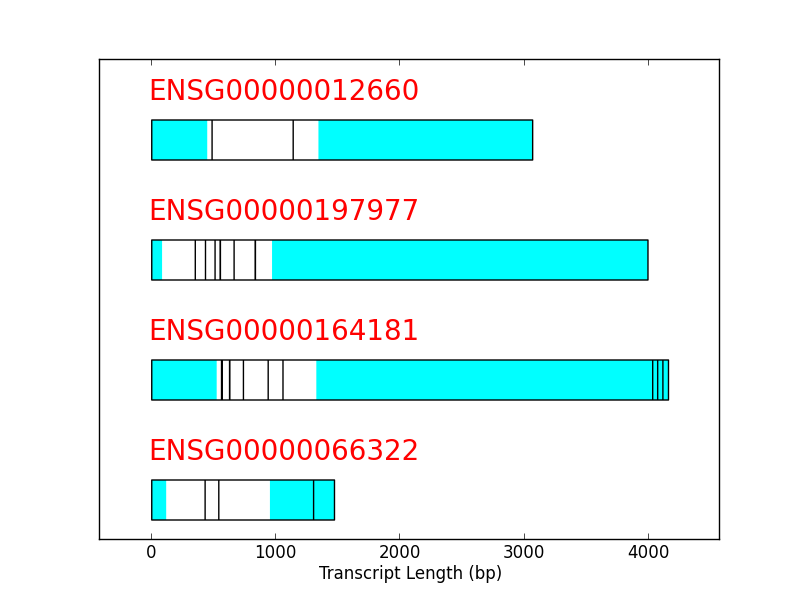

Supplement: Data file S2 [file rsob140029supp3.zip › rsob-14-0029-File010/Melanoma/ENSG00000066322_ENSG00000164181_ENSG00000197977_ENSG00000012660.png]

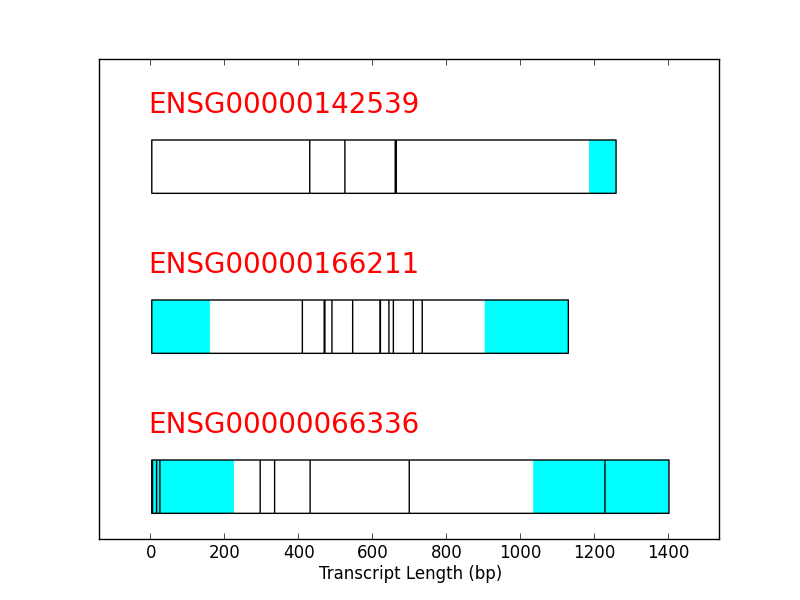

Supplement: Data file S2 [file rsob140029supp3.zip › rsob-14-0029-File010/Melanoma/ENSG00000066336_ENSG00000166211_ENSG00000142539.png]

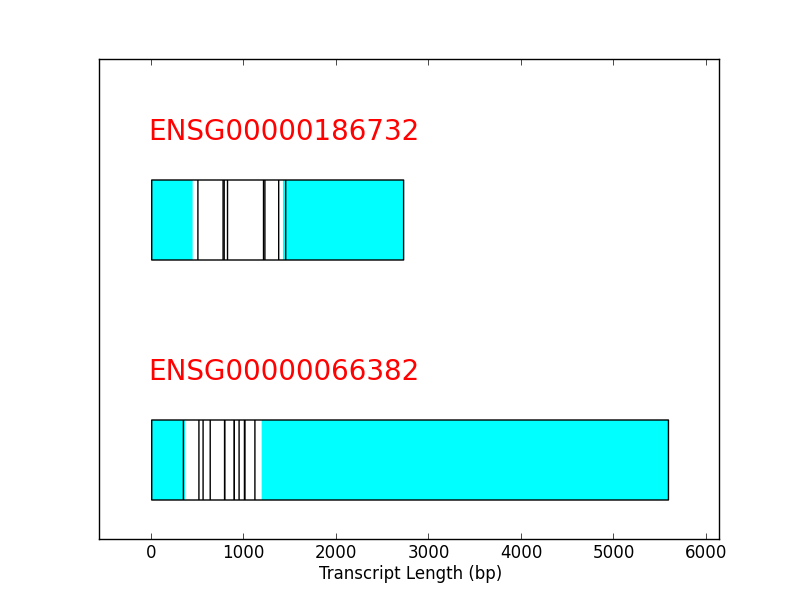

Supplement: Data file S2 [file rsob140029supp3.zip › rsob-14-0029-File010/Melanoma/ENSG00000066382_ENSG00000186732.png]

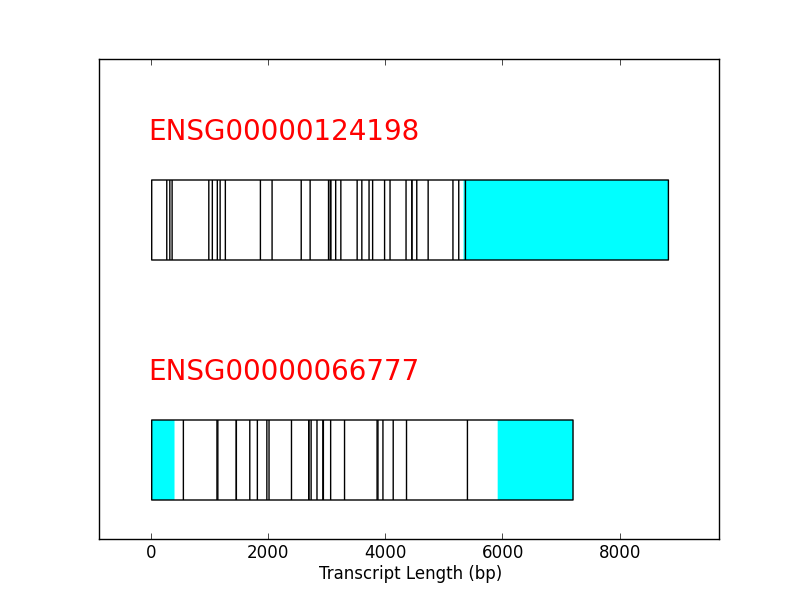

Supplement: Data file S2 [file rsob140029supp3.zip › rsob-14-0029-File010/Melanoma/ENSG00000066777_ENSG00000124198.png]

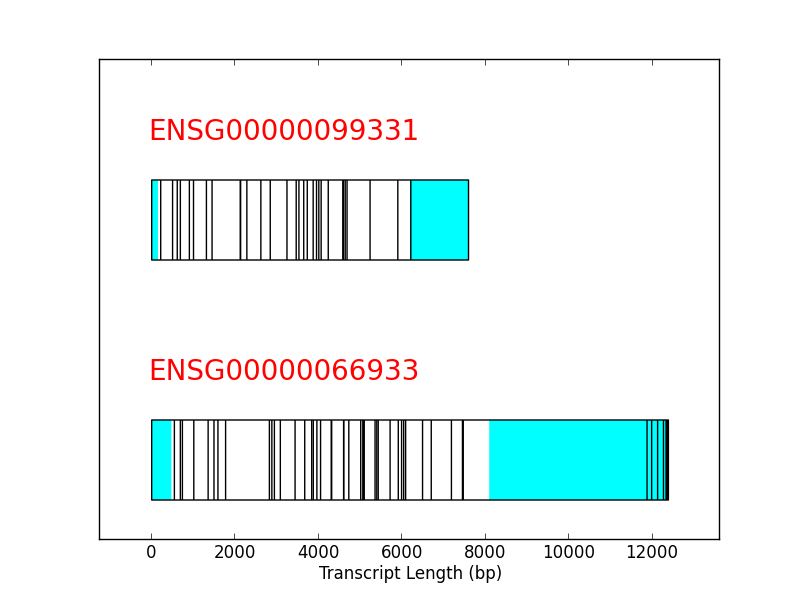

Supplement: Data file S2 [file rsob140029supp3.zip › rsob-14-0029-File010/Melanoma/ENSG00000066933_ENSG00000099331.png]

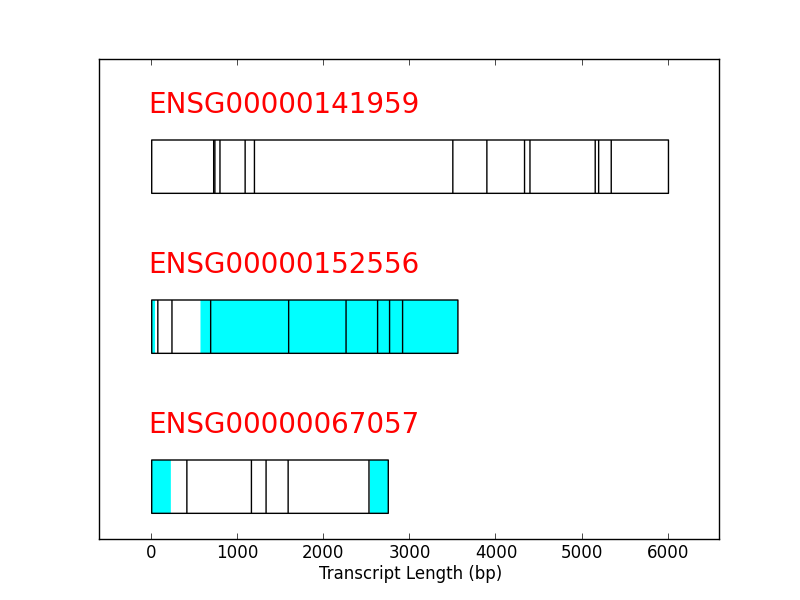

Supplement: Data file S2 [file rsob140029supp3.zip › rsob-14-0029-File010/Melanoma/ENSG00000067057_ENSG00000152556_ENSG00000141959.png]

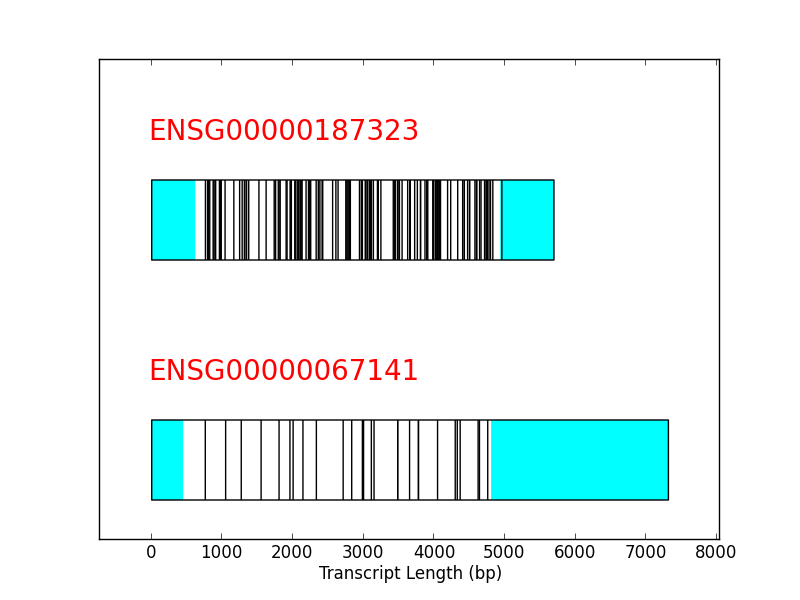

Supplement: Data file S2 [file rsob140029supp3.zip › rsob-14-0029-File010/Melanoma/ENSG00000067141_ENSG00000187323.png]

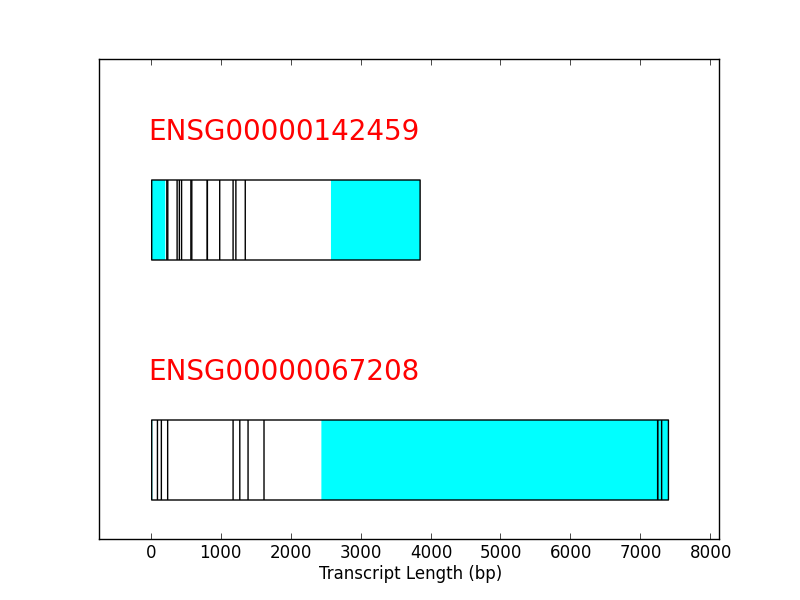

Supplement: Data file S2 [file rsob140029supp3.zip › rsob-14-0029-File010/Melanoma/ENSG00000067208_ENSG00000142459.png]

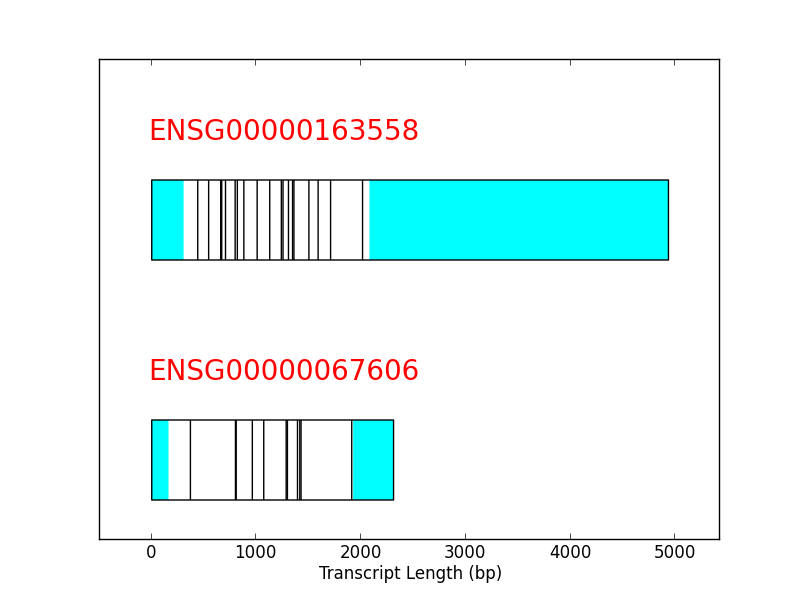

Supplement: Data file S2 [file rsob140029supp3.zip › rsob-14-0029-File010/Melanoma/ENSG00000067606_ENSG00000163558.png]

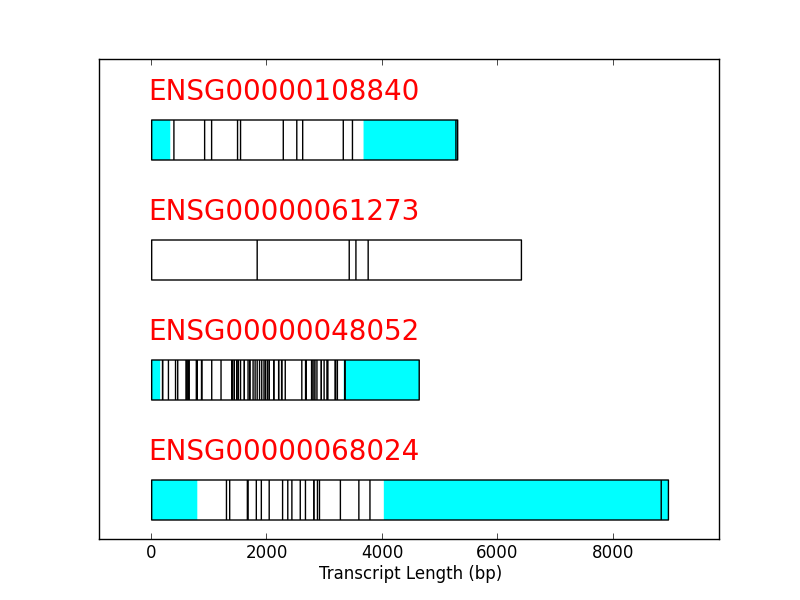

Supplement: Data file S2 [file rsob140029supp3.zip › rsob-14-0029-File010/Melanoma/ENSG00000068024_ENSG00000048052_ENSG00000061273_ENSG00000108840.png]

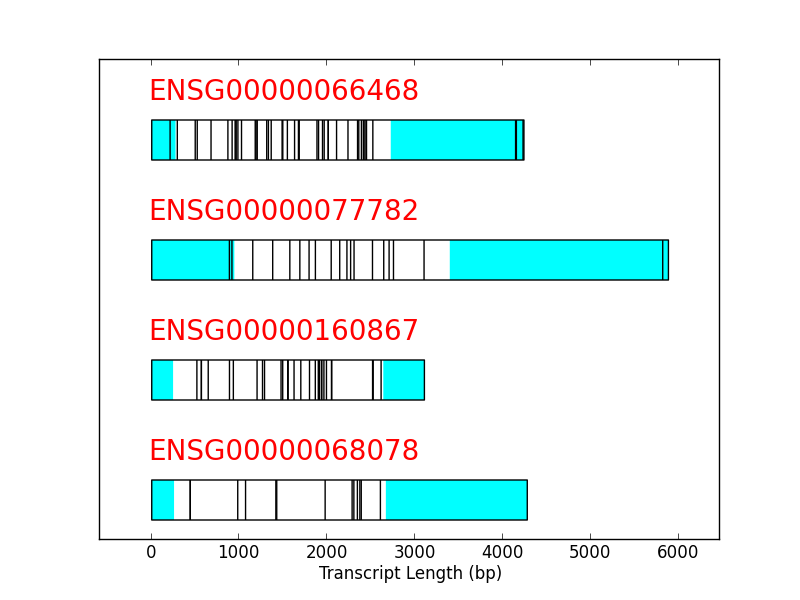

Supplement: Data file S2 [file rsob140029supp3.zip › rsob-14-0029-File010/Melanoma/ENSG00000068078_ENSG00000160867_ENSG00000077782_ENSG00000066468.png]

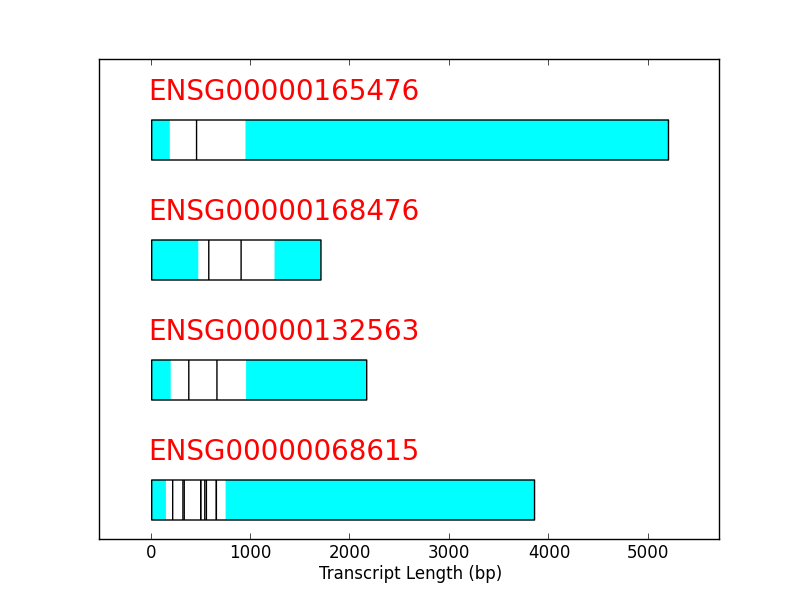

Supplement: Data file S2 [file rsob140029supp3.zip › rsob-14-0029-File010/Melanoma/ENSG00000068615_ENSG00000132563_ENSG00000168476_ENSG00000165476.png]

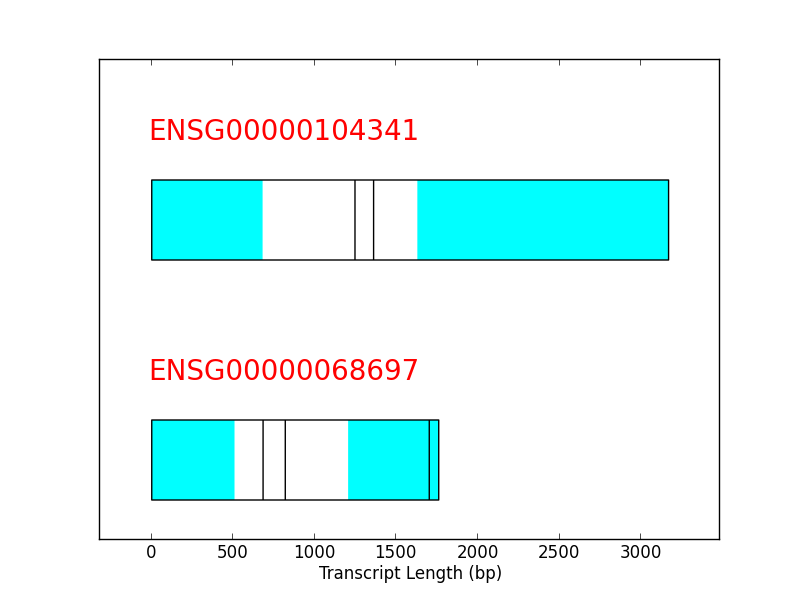

Supplement: Data file S2 [file rsob140029supp3.zip › rsob-14-0029-File010/Melanoma/ENSG00000068697_ENSG00000104341.png]

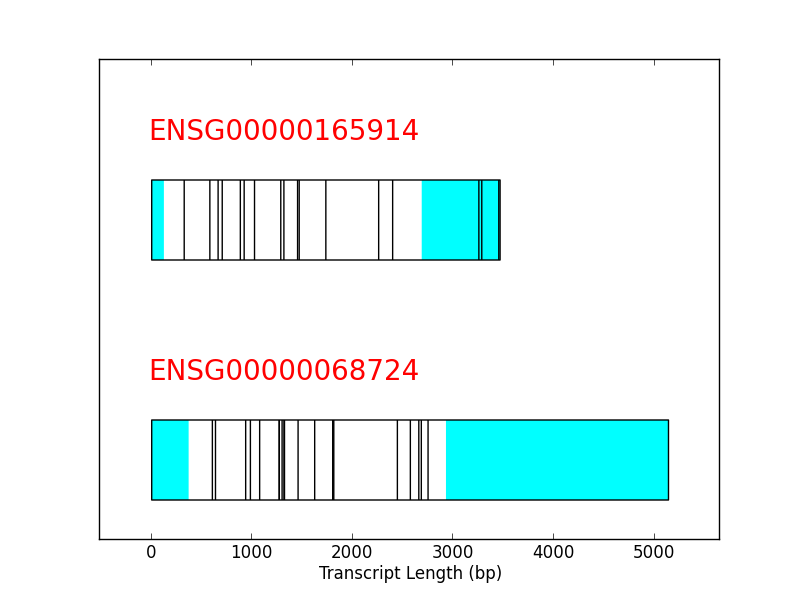

Supplement: Data file S2 [file rsob140029supp3.zip › rsob-14-0029-File010/Melanoma/ENSG00000068724_ENSG00000165914.png]

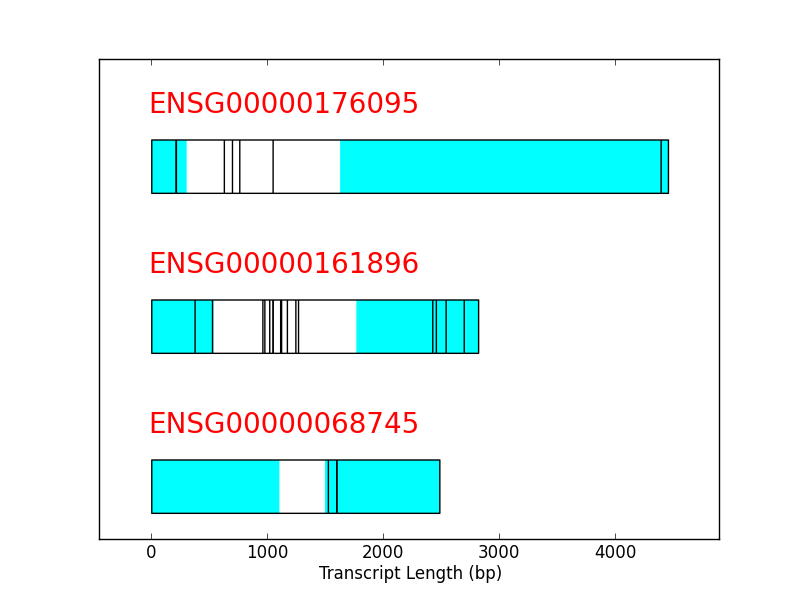

Supplement: Data file S2 [file rsob140029supp3.zip › rsob-14-0029-File010/Melanoma/ENSG00000068745_ENSG00000161896_ENSG00000176095.png]

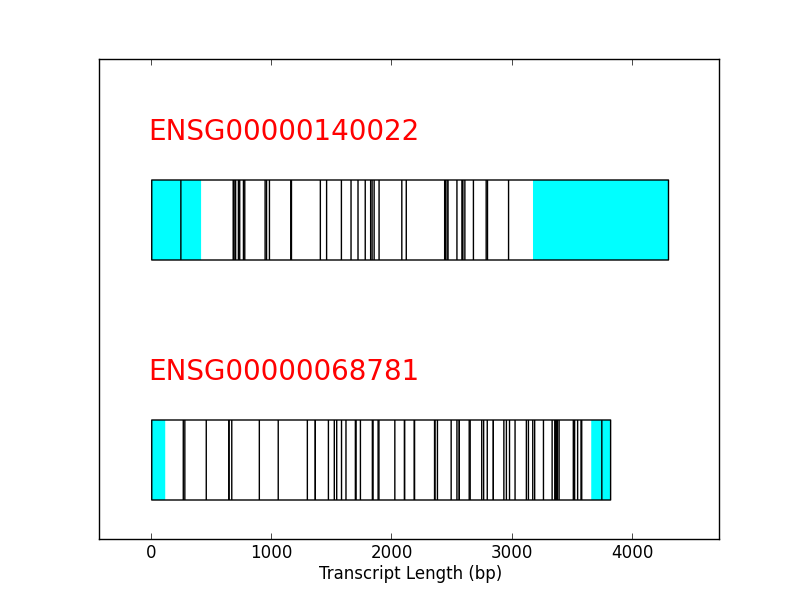

Supplement: Data file S2 [file rsob140029supp3.zip › rsob-14-0029-File010/Melanoma/ENSG00000068781_ENSG00000140022.png]
